# Gene Models, Expression Repertoire, and Immune Response of Plasmodium vivax Reticulocyte Binding Proteins

**DOI:** 10.1128/IAI.01117-15

**Published:** 2016-02-24

**Authors:** Jenni Hietanen, Anongruk Chim-ong, Thanprakorn Chiramanewong, Jakub Gruszczyk, Wanlapa Roobsoong, Wai-Hong Tham, Jetsumon Sattabongkot, Wang Nguitragool

**Affiliations:** a Department of Molecular Tropical Medicine, Faculty of Tropical Medicine, Mahidol University, Bangkok, Thailand; b The Walter and Eliza Hall Research Institute of Medical Research, Parkville, Victoria, Australia; c Mahidol Vivax Research Unit, Faculty of Tropical Medicine, Mahidol University, Bangkok, Thailand; d Department of Medical Biology, The University of Melbourne, Parkville, Victoria, Australia

## Abstract

Members of the Plasmodium vivax reticulocyte binding protein (PvRBP) family are believed to mediate specific invasion of reticulocytes by P. vivax. In this study, we performed molecular characterization of genes encoding members of this protein family. Through cDNA sequencing, we constructed full-length gene models and verified genes that are protein coding and those that are pseudogenes. We also used quantitative PCR to measure their *in vivo* transcript abundances in clinical P. vivax isolates. Like genes encoding related invasion ligands of P. falciparum, *Pvrbp* expression levels vary broadly across different parasite isolates. Through antibody measurements, we found that host immune pressure may be the driving force behind the distinctly high diversity of one of the family members, PvRBP2c. Mild yet significant negative correlation was found between parasitemia and the PvRBP2b antibody level, suggesting that antibodies to the protein may interfere with invasion.

## INTRODUCTION

The two major malaria parasites, Plasmodium falciparum and Plasmodium vivax, differ in their abilities to invade human erythrocytes. P. falciparum invades both mature erythrocytes and reticulocytes, but P. vivax can invade only the latter (1). Such a difference can have a profound influence on disease pathology and impose distinct metabolic requirements for the intracellular parasites (2). The molecular basis for this differential host trophism is not completely understood but is generally believed to result from the use of different ligand-receptor interactions for cellular recognition.

Two families of Plasmodium proteins have been implicated in host cell sensing. The first family is composed of proteins that contain the erythrocyte binding-like (EBL) domain, a protein domain conserved across the genus Plasmodium. These proteins include the Duffy binding protein of P. vivax (PvDBP) and the erythrocyte binding antigens PfEBA175, PfEBA181, and PfEBL-1 of P. falciparum. The second protein family is called the reticulocyte binding-like (RBL) family; its members, including P. vivax reticulocyte binding proteins (PvRBPs) and P. falciparum reticulocyte binding protein homologs (PfRHs). It has long been hypothesized that the members of these two protein families function at different steps during invasion (3). Consistent with this, PfRH1 was recently shown to have an initial erythrocyte-sensing function that triggers Ca^2+^-dependent discharge of PfEBA-175 from the micronemes (4). To date, specific human receptors have been identified for PfEBA140, PfEBA175, PfEBL-1, PfRH4, and PfRH5 of P. falciparum (5–10) and PvDBP of P. vivax (11, 12). None of these receptors are uniquely present on reticulocytes, and little is yet known about the PvRBP family.

PvRBPs were originally discovered by Galinski and coworkers through an effort to identify P. vivax invasion ligands that bind reticulocytes (13). From this study, two high-molecular-mass proteins of 250 kDa were discovered in the Belem strain of P. vivax and named PvRBP1 and PvRBP2. Available evidence suggests that genes encoding these proteins are transcribed in the schizont stage as 9.5-kb-long RNA and that the protein products form a high-molecular-mass complex at the apical tip of the merozoite. While direct evidence demonstrating the roles of these proteins in invasion is still lacking, their sequence similarity to the much better characterized PfRHs suggests that they may be involved in reticulocyte sensing (3) and could similarly hold potential as targets in malaria vaccine development. Interestingly, sequence analysis has demonstrated much higher sequence diversity in the *Pvrbp2* gene than in the *Pvrbp1* gene (14), pointing to a difference in protein function or selection pressure.

When the genome sequence of P. vivax became available, the family of *Pvrbp* genes grew to include many more members, several of which were predicted to be protein-coding genes (*Pvrbp1a*, *Pvrbp1b*, *Pvrbp2a*, *Pvrbp2b*, *Pvrbp2c*, *Pvrbp1p*, *Pvrbp2p1*, and *Pvrbp2p2*), while others were predicted to be pseudogenes (*Pvrbp2d*, *Pvrbp2e*, and *Pvrbp3*) (15, 16). The original *Pvrbp1* and *Pvrbp2* of the Belem strain identified by Galinski and coworkers correspond to *Pvrbp1a* and *Pvrbp2c* of the Sal-1 reference genome, respectively. Thus far, little effort has been made to validate the gene models, leaving uncertainties in the predictions of the start and stop codons, the number of introns, the positions of exon-intron junctions, and the presence of the signal peptide and the conserved C-terminal transmembrane domain. These issues are apparent in a recent review listing several missing elements (17).

In this study, we aimed to resolve these issues by completely sequencing the cDNAs of multiple members of the PvRBP family. We also developed sensitive quantitative-PCR (qPCR) assays to measure their transcript abundances and measured IgG responses to five of the PvRBPs. Using blood samples from malaria patients, we demonstrated high variations in PvRBP expression between different parasite isolates. PvRBP2c's distinctly high genetic diversity was also found to be associated with an elevated host antibody level in P. vivax-infected patients.

## MATERIALS AND METHODS

The use of human specimens in this study was approved by the Ethics Committee of the Faculty of Tropical Medicine, Mahidol University, Bangkok, Thailand, under certificate no. MUTM 2013-086-01.

### cDNA preparation.

Parasite samples were collected from P. vivax-infected patients by venous blood draw into heparin-containing tubes. RNA was extracted from the parasite pellet derived from 1 to 2 ml whole blood after leukocyte removal with Plasmodipur filters (EuroProxima) and erythrocyte lysis with 0.1% saponin, following the standard TRIzol reagent isolation method (Life Technologies). The extracted RNA was further purified using RNeasy minikits (Qiagen) with on-column DNase treatment. Lack of amplification by sensitive qPCR (qMAL) targeting Plasmodium 18S rRNA genes (18) was used to ensure that no genomic DNA (gDNA) contamination was present in all RNA samples used in this study. cDNA was generated using the Superscript III first-strand synthesis system (Life Technologies), following the manufacturer's protocol with the following exceptions: (i) cDNA synthesis was extended from 50 min to 1 h and (ii) a mixture of the supplied oligo(dT) (0.05 μg/μl) and a primer (TGTTGAGTCAAATTAAGCCGCAA; 200 nM) targeting Plasmodium 18S rRNA genes was used during reverse transcription. The 18S rRNA primer was included to allow sensitive verification by qMAL that cDNA was successfully produced during reverse transcription.

### cDNA sequencing.

To sequence the cDNA of each *Pvrbp* gene, we used PCR to amplify several overlapping fragments from one or more cDNA preparations to cover the entire length of the gene. The PCR products were then subjected to dye terminator sequencing (Macrogen). After sequence assembly, introns were identified as stretches of gDNA sequence in the Sal-1 reference strain that do not appear in the cDNA sequences. Transmembrane domains and signal peptides in translated protein sequences were predicted using the TMHMM and Phobius servers (19, 20).

### Diagnostic PCR to detect *Pvrbp* genes in gDNA.

DNA was extracted from 200 μl patient blood with a QIAamp DNA Blood minikit (Qiagen). The primers used in diagnostic PCR to detect each gene, as well as the predicted sizes of the amplified products, are listed in Table 1.

**TABLE 1:** Diagnostic PCR to determine presence of *Pvrbp* genes

Gene	Primer sequence^*a*^	Primer position^*b*^ (base)	Product size (bp)
*Pvrbp1a*	ACACGATGCAGGTGCAGA	8264	538
	ACCCATACGTATACCTACCAG	8801	
*Pvrbp1b*	CGGATGAAGCTGACAAGGA	4642	853
	GGGTCCGAATTCTGGGATA	5494	
*Pvrbp2a*	GGCAAGGCAAATTGTTCAGC	7321	122
	ACGGACATTTCCATTTTCATTTCT	7442	
*Pvrbp2b*	CACAACATGAGGATCAGTCACAAC	8468	94
	ATACCTGAAAAGACAGATAATCCCAAA	8561	
*Pvrbp2c*	GTGGGAAACTTTTCTTCCCTCT	5431	166
	CTGAAGTGTTTTCCGAAGCA	5596	
*Pvrbp3*	CTGGTGACAACCATTCTGATGAC	8413	187
	AAATCGGCTTCTTGTGATTCATG	8599	
*Pvrbp2e*	CGACTGCTACAAACATATTAGATAACACA	4336	111
	TCTGAGCTACCGCTTCTTCTAATAATT	4466	
	CGGAATTGCAAGACAAAGC	2299	844
	GCGATCGATTGTTCGTTTG	3142	
	TCGACAAAGGCATGGAAA	3367	1,314
	CGCTGTGCTTAACCGTTTT	4680	
	GCCGAATTAGACAAACATTCA	6333	1,494
	TTTACGCGTTCCTTTTCACC	7826	
*Pvrbp2p1*	TTGTTTCTACTTGTGAAGAAGCCAAA	1622	126
	CATCATAACTYTGTTTAACTGATGCAATT	1747	
*Pvrbp2d*	CAGAAGAGAAACTTTAGAAAGTGACCT	7402	86
	CTTCACTTAATGTATTCGCATTTAATT	7487	

a Primer sequences are shown from 5′ to 3′. Primer pairs are listed in the same order as in Fig. 2.

b The position of the 5′ end of each primer is relative to the start codon of each gene in the Sal-1 reference strain, except for *Pvrbp2e*, which is absent from the strain. The positions of *Pvrbp2e* primers are based on the sequence of the VTTY57 isolate in this study.

### Quantitative PCR.

Each target gene fragment was inserted into a T&A plasmid (RBC Bioscience) to generate qPCR standards. Each qPCR assay was optimized and performed on CFX96 real-time PCR (Bio-Rad) with iTaq Universal Probe Supermix (Bio-Rad). Efficiencies of amplification were determined from 10-fold serial dilution of the plasmid standards. The detection limits of each assay were defined as the lowest copy numbers of circular plasmids detected with a probability greater than 0.5.

cDNA was prepared from DNase-treated RNA as described above with the Superscript III first-strand synthesis system (Life Technologies) and diluted 10-fold in water before use. Four microliters of diluted cDNA was used in each 15-μl qPCR mixture, which contained 7.5 μl of iTaq Universal Probe Supermix (Bio-Rad), 400 nM each primer, and 400 to 600 nM probe. All probes were 5′ labeled with HEX (6-carboxy-2′,4,4′,5′,7,7′- hexachlorofluorescein) and 3′ labeled with black hole quencher 1 (BHQ1). Measurements were done in triplicate. Temperature cycling for all genes (*Pvdbp*, *Pvama1*, and *Pvrbp* genes) involved initial heating to 95°C for 5 min, followed by 50 cycles of heating at 95°C for 15 s and cooling at 60°C for 45 s. Amplification data were acquired and analyzed with CFX Manager software (Bio-Rad). Data normalization and plots were created in SigmaPlot 13 (Systat Software). Statistical analyses were performed with PASW Statistics 18 (IBM) after log transformation. GeneSpring 13.1 (Agilent Technologies) was used to generate the heat map and for hierarchical clustering of gene expression with centroid linkage and the Pearson correlation coefficient as the similarity measure.

### Protein expression and purification.

DNA sequences encoding PvRBP1a (amino acids 160 to 1170), PvRBP1b (amino acids 140 to 1275), PvRBP2a (amino acids 160 to 1135), PvRBP2b (amino acids 161 to 1454), and PvRBP2c (amino acids 501 to 1300) were codon optimized from the Sal-1 protein sequences for Escherichia coli expression and inserted into the pPROEX HTB expression vector (Life Technologies). Protein production involved growing plasmid-transformed E. coli (SHuffle T7; New England BioLabs) in 2 liters of terrific broth (TB) with 100 mg carbenicillin/liter at 37°C and 200 rpm until the optical density at 600 nm reached ∼1. Expression was then induced with 1 mM isopropyl-β-d-thiogalactopyranoside (Astral) at 16°C for 20 h. Cells were harvested by 6,000 × *g* centrifugation. After resuspension in 30 ml of lysis buffer (500 mM NaCl, 50 mM Tris-HCl, pH 7.5, 10% [vol/vol] glycerol), the cells were sonicated in the presence of 0.5 mg/ml DNase and 1.0 mg/ml lysozyme (Sigma) and clarified by centrifugation at 30,000 × *g* for 30 min at 4°C. The supernatant was subsequently filtered through a 0.22-μm filter before being loaded onto a 5-ml HisTrap column (GE Healthcare) preequilibrated with 50 mM Tris-HCl, 500 mM NaCl, 10% glycerol, and 20 mM imidazole. After column washing with >10 column volumes of the same buffer, bound protein was eluted in the same buffer supplemented with 300 mM imidazole. The His tag was removed by recombinant tobacco etch virus (TEV) protease produced in-house at 0.5 mg per 10 mg target proteins overnight at 4°C, while the sample was dialyzed against 5 liters of buffer containing 100 to 150 mM NaCl, 20 mM buffer (Bis-Tris-HCl, pH 6.5, for PvRBP1a; Tris-HCl, pH 7.5, for PvRBP1b and PvRBP2c; Tris-HCl, pH 8.5, for PvRBP2a; Tris-HCl, pH 8.0, for PvRBP2b). The cleaved protein was applied to Ni-nitrilotriacetic acid (NTA) again and collected as flowthrough before the final purification on an S200 Superdex size exclusion column (GE Healthcare) preequilibrated in 200 mM NaCl with 20 mM buffer at the same pH as mentioned above. The peak fraction containing pure protein was concentrated with a Vivaspin 15 Turbo centrifugal concentrator (Sartorius) with a molecular mass cutoff of 50 kDa. The purified proteins were analyzed by SDS-PAGE.

### Enzyme-linked immunosorbent assays (ELISA).

All 41 plasma samples used in this study were obtained from P. vivax malaria patients (aged 18 to 66) at the Malaria Clinic in Bongti village, Kanchanaburi Province, in western Thailand between August 2011 and June 2012. Plasma was stored at −80°C. Nunc MaxiSorp flat-bottom 96-well plates (eBioscience) were coated with recombinant PvRBPs at 0.88 pmol/well in phosphate-buffered saline (PBS) (pH 7.4) and incubated overnight at 4°C. All subsequent steps were carried out at room temperature. The plates were blocked with 200 μl/well of blocking solution (5% skim milk in PBS with 0.1% Tween 20) for 1 h. After removal of the blocking solution, 1 μl of patient plasma in 100 μl blocking solution was added and incubated for 2 h. The wells were then washed three times with 200 μl PBS with 0.1% Tween 20 (PBST), followed by addition of 100 μl of 100 ng/ml goat anti-human IgG(H+L) secondary antibody conjugated with horseradish peroxidase (Pierce) in blocking buffer. After 1 h incubation, the wells were washed three times with 200 μl PBST, and 100 μl 2,2′-azinobis(3-ethylbenzthiazolinesulfonic acid) (ABTS) (Merck Millipore) was added. Measurement of absorbance (abs) kinetics at 405 nm was initiated within 2 min on a microplate reader (Synergy H1 [Biotek] for PvRBP1b and PvRBP2c and Sunrise [Tecan] for PvRBP1a, PvRBP2a, and PvRBP2b), with a sampling frequency of >2 min^−1^. The final time course for each plasma sample was obtained by subtracting the absorbance of the mock-control well without protein coating from that of the test well with a recombinant PvRBP coating. Slopes (Δabs/min) were measured during the linear phase of absorbance increase (within the first 5 min 30 s after ABTS was added). Serial dilution of purified antibody standards suggested that the slopes vary approximately linearly with the log antibody concentration. Plasma from a Thai person with no past malaria exposure was used as the naive control on all plates. Statistical analyses were performed with PASW Statistics 18 (IBM). Spearman rank correlation coefficients (ρ) were used to determine the correlation between IgG responses to different PvRBPs. Pearson coefficients (*r*) were used to determine correlations between IgG responses and age or parasitemia.

### Nucleotide sequence accession numbers.

The GenBank accession numbers of the cDNA sequences from this study are listed in Table 2.

**TABLE 2:** *Pvrbp* gene model parameters

Gene	Gene ID^*a*^	Chromosome	Isolate^*b*^	Length (bases)	No. of introns	Intron (bases)	CDS^*c*^ length (bases)	Protein length (amino acids)	Protein size (kDa)	SP^*d*^	TM^*e*^
*Pvrbp1a*	PVX_098585 (KU158834)	7	VKBT95, VKBT99, VTTY57	8,704	1	53–254	8,502	2,833	326	Yes	1
*Pvrbp1b*	PVX_098582 (KU158835)	7	VTTY57	8,759	1	56–195	8,619	2,872	334	Yes	1
*Pvrbp2a*	PVX_121920 (KU158836)	14	VKBT95	7,643	1	56–234	7,464	2,487	286	Yes	1
*Pvrbp2b*	PVX_094255 (KU158837)	8	VTTY57	8,709	1	56–343	8,421	2,806	326	Yes	1
*Pvrbp2c*	PVX_090325 (KU158838)	5	VKBT95, VTTY57	8,658	1	56–256	8,457	2,818	325	Yes	1
*Pvrbp2p1*	PVX_090330 (KU158839)	5	VTTY57	2,070	1	56–256	1,869	622	72	Yes	0
*Pvrbp2d*	PVX_101585 (KU158840)	14	VKTS93, VTTY49, VTTY55, VTTY59, VTTY67	8,705	1	56–268	Pseudogene			Yes	1
*Pvrbp2d_tr_*	PVX_101585 (KU158841)	14	VTTY57	8,080	0		Pseudogene			No	1
*Pvrbp2e*	PVBG_04845 (KU158842)	7	VTTY57	8,445	1	56–300	Pseudogene			Yes	1
*Pvrbp3*	PVX_101495 (KU158843)	14	VTTY49, VTTY57	8,608	1	56–240	Pseudogene			Yes	1

a Gene identifiers (IDs) as used on public databases (http://www.plasmodb.org and http://www.broadinstitute.org/annotation/genome/plasmodium_vivax/MultiHome.html). In parentheses are the GenBank accession numbers of the cDNA sequences from this study.

b Isolate(s) contributing to each final gene model.

c CDS, coding sequence.

d Presence of signal peptide coding sequence.

e Number of transmembrane domain coding sequences.

## RESULTS

### *Pvrbp* gene models.

**FIG 1:**
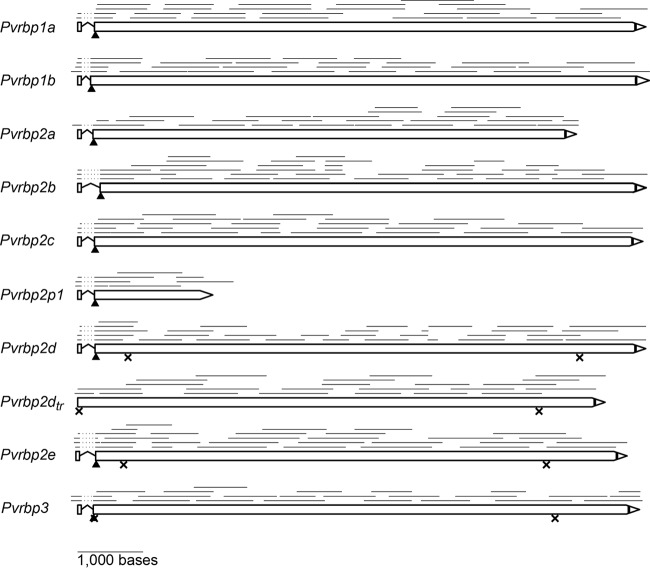
*Pvrbp* gene models. The models were constructed by aligning cDNA sequences with the genome of the Sal-1 reference strain. The lines above each gene model represent cDNA sequence reads; the dotted portion near the start codon of each gene denotes an intron. The arrowheads indicate the predicted signal peptide cleavage sites, while vertical black bars indicate the predicted transmembrane domains of the translated protein. The crosses indicate the positions of internal stop codons; for clarity, only the nearest internal stop codons in frame with the start and the final stop codons are shown.

Of the 8 previously annotated full-length *Pvrbp* genes (*Pvrbp1a*, *Pvrbp1b*, *Pvrbp2a*, *Pvrbp2b*, *Pvrbp2c*, *Pvrbp2d*, *Pvrbp2e*, and *Pvrbp3*) and 4 partial gene fragments (*Pvrbp1p*, *Pvrbp2p1*, *Pvrbp2p2*, and *Pvrbp2hb*), we chose to experimentally verify the gene models for 9 of them (Fig. 1). We did not work on *Pvrbp1p* (PVX_125738) because the gene fragment has a sequence mostly identical to that of an internal portion of *Pvrbp1b* and it has not been chromosomally located. We failed to amplify *Pvrbp2p2* (PVX_101590) from both cDNA and gDNA of all the P. vivax samples tested. The gene may thus be present in only a limited number of strains, including the Sal-1 reference strain. Finally, we did not study *Pvrbp2hb* (PVX_116930) because it has noticeably lower sequence similarity to other *Pvrbp* genes and is not located in the subtelomeric regions, like all the other genes.

Of the 9 chosen genes, several appear to lack the signal peptide (SP) coding sequence in a recent annotation (17). Because SP is conserved in RBL proteins with known functions, we *in silico* searched the genome sequence for a potential signal peptide coding sequence in all three reading frames, up to 2,000 bases upstream of the annotated start codon of these *Pvrbp* genes. Using this method, we could identify probable start codons for all *Pvrbp* genes. Putative stop codons were detected following identification of the acidic C-terminal domain downstream of a transmembrane domain (TM), also a conserved feature of RBL proteins.

To construct the gene models of the selected *Pvrbp* genes, we used PCR to amplify several overlapping fragments, using cDNA of P. vivax patient blood as the template. Dye terminator sequencing of these fragments revealed a single intron near the start codon for each gene (Fig. 1), as previously predicted (13). These data validate the two-exon structure and reveal the key features of RBL genes, such as SP and TM coding sequences. The first short exon spans SP almost entirely, while the second exon encodes the rest of the protein. All identified introns have the canonical eukaryotic 5′ GU/AG 3′ boundaries. We found two forms of *Pvrbp2d* in different P. vivax samples, one full length (*Pvrbp2d*) and the other with nucleotide deletion between bases 140 and 763 (*Pvrbp2d_tr_*). This truncation is present in gDNA (data not shown) and results in loss of splicing. Table 2 summarizes the model parameters of these *Pvrbp* genes. cDNA sequences, as well as their alignment with the Sal-1 reference sequences, can be found in the supplemental material.

Of the 9 genes, 5 (*Pvrbp1a*, *Pvrbp1b*, *Pvrbp2a*, *Pvrbp2b*, and *Pvrbp2c*) were confirmed to encode full-length proteins, with expected molecular masses of 286 to 326 kDa. One gene (*Pvrbp2p1*) appears to be a partial N-terminal fragment but is likely long enough to encode a functional protein based on its similarity to the known binding domain of PfRH5 (5). The other three full-length genes (*Pvrbp2d*, *Pvrbp2e*, and *Pvrbp3*) are presumably pseudogenes because they contain multiple internal stop codons due to indels.

### Presence of *Pvrbp* genes in natural P. vivax isolates.

**FIG 2:**
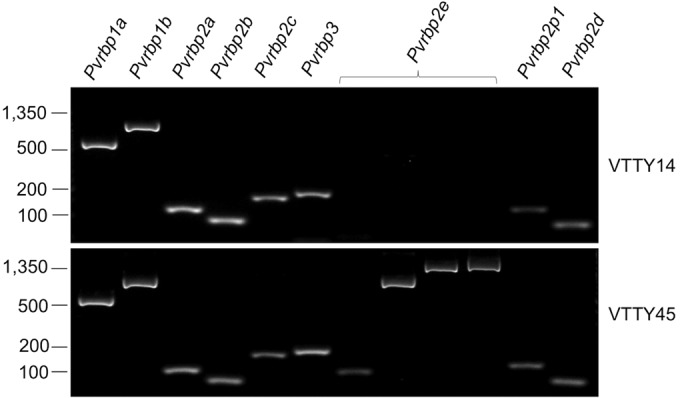
Absence of *Pvrbp2e* in VTTY14 gDNA. Diagnostic PCR was used to examine the presence of *Pvrbp* genes in P. vivax genomes. Four primer pairs were used to ensure loss of *Pvrbp2e* in VTTY14. VTTY45 is representative of 70 other samples examined, in which all the genes were present. Values at left indicate DNA sizes in base pairs.

Deletion of a gene from the genome is an indication that it is nonessential. We sought to identify such genes in the *Pvrbp* family by PCR. Of the 72 DNA samples prepared from different patient blood, we failed to amplify only one gene, *Pvrbp2e*, in one sample, VTTY14 (Fig. 2). Four separate primer pairs were used to ensure the absence of the gene. Every other *Pvrbp* gene was detected in all the samples. This gene was originally discovered after *de novo* sequencing of a field isolate from Cambodia (16). Interestingly, *Pvrbp2e* was also absent from the monkey-adapted Sal-1 strain as a result of genomic deletion in chromosome 7. Our result suggests that a loss of *Pvrbp2e* already occurs in the natural parasite population and is not necessarily due to simian adaptation of the Sal-1 strain.

### Expression repertoire of *Pvrbp* in patients.

**FIG 3:**
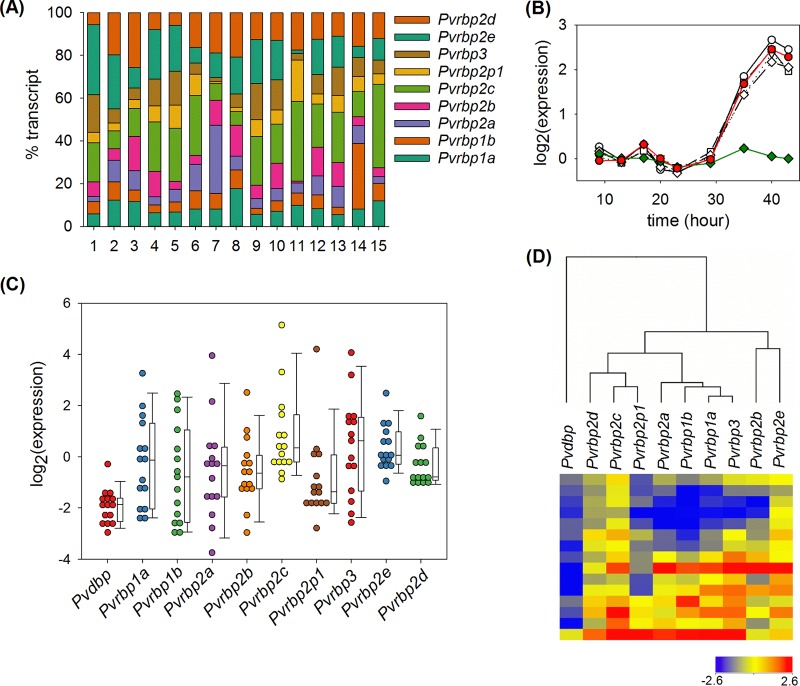
(A) Distribution of *Pvrbp* transcript abundances in 15 P. vivax malaria patient samples. Each stacked bar represents the transcript copy number normalized by the sum of all *Pvrbp* gene transcripts in each patient. Data for individual genes are arranged from top to bottom as indicated. (B) The average time courses of transcript abundance during the blood stage life cycle were constructed from published microarray data (22). Red circles, *Pvama1*; open circles, *Pvrbp1a*; open triangles, *Pvrbp2a*; open squares, *Pvrbp2b*; open diamonds, *Pvrbp2c*; green diamonds, a housekeeping gene encoding methionine tRNA ligase (PVX_110980). Each time course was baseline subtracted with the average log_2_(expression) during the first 30 h. (C) Distribution of *Pvama1*-normalized transcript copy numbers of *Pvdbp* and *Pvrbp* genes. Each circle represents data from a single patient. The whiskers on the box plots indicate the 10th and 90th percentiles. (D) Heat map and clustering of *Pvama-1*-normalized expression based on the Pearson correlation coefficient. Each row of the heat map represents data from a single patient.

The number of invasion pathways, or ligand-receptor interactions used for invasion, of P. vivax was once thought to be much more limited than that of P. falciparum. This idea originated from the restrictive ability of P. vivax to invade primarily reticulocytes (1), the almost universal importance of Duffy antigen receptor for chemokines (DARC) in invasion (21), and the early identification of only two *Pvrbp* genes (13). The expansion of the PvRBP family after whole-genome sequencing (15) challenged this idea. To date, only one study has been conducted to examine the expression of these proteins systematically, and it was done with three parasite isolates from western Thailand (22). To assess the expression repertoire of *Pvrbp* genes, we developed qPCR assays to measure their transcript abundances (Table 3). We applied these assays to cDNA preparations from 15 different P. vivax patient blood samples and found that the genes were expressed in all the samples (Fig. 3A; see Table S1 in the supplemental material). Because patient blood generally contained parasites at different maturation stages, we normalized the *Pvrbp* transcript numbers with that of *Pvama1*, a schizont-specific gene whose expression timing closely matches those of *Pvrbp* genes (Fig. 3B). After normalization, we found that *Pvrbp* genes exhibited high sample-to-sample variation compared to *Pvdbp* (Fig. 3C). Correlation analysis of gene expression across samples suggested a common transcription regulation mechanism among several members. Notably, the *Pvama1*-normalized expression of *Pvrbp1a* was highly correlated with those of *Pvrbp3* (*r* = 0.971; *P* = 2 × 10^−9^) and *Pvrbp1b* (*r* = 0.924; *P* = 8 × 10^−7^). Likewise, the expression levels of the neighboring genes *Pvrbp2c* and *Pvrbp2p1* were tightly correlated (*r* = 0.921; *P* = 1 × 10^−6^). In contrast, no significant correlation was found between *Pvrbp2b* and *Pvrbp2c*, *Pvrbp2p1*, or *Pvrbp2d* or between *Pvrbp2e* and *Pvrbp2c*, *Pvrbp2p1*, or *Pvrbp2d* (*r* < 0.378; *P* > 0.05). Clustering analysis of gene expression (Fig. 3D) revealed grouping of *Pvrbp1a*, *Pvrbp1b*, *Pvrbp2a*, and *Pvrbp3* and of *Pvrbp2c*, *Pvrbp2p1*, and *Pvrbp2d*. *Pvrbp2b* and *Pvrbp2e* are more distantly related to other genes. As expected, *Pvdbp* is placed distinctly outside the *Pvrbp* clade.

**TABLE 3:** qPCR assays to detect *Pvrbp*s

Target gene	Primers and probe^*a*^	Position^*b*^ (base)	*R*^2^	Eff^*c*^ (%)	Detection limit (copies/reaction)
*Pvama1*	CCTACTTCAGGAAGAAGGCTAACAAT	1514	0.999	107.5	<5
	GGTGGTGGGCTTCCCGTA	1590			
	HEX-CCCTCTGCCTGGTCCATCTTGTCATACTT-BHQ1	1571			
*Pvdbp*	TTAAAGACAAGACTTACTTTGCCGC	3146	0.999	105.8	<5
	TTTGATCATCTTCCTTGAAGCAATTAA	3330			
	HEX-AACAGCAGTATCAGCAACGCTCCCG-BHQ1	3302			
*Pvrbp1a*	GCATACAGAGAACACCCAGGATG	8417	0.999	98.7	<5
	GCTCCTCTTGGTCATCTTTCTTATGT	8598			
	HEX-CCTATCAGGATACGTCAAATTCCAGCGATG-BHQ1	8443			
*Pvrbp1b*	AGCAGGCTAAGACAGTTGCGAA	7408	0.999	100.0	<5
	AATGTCCTTCTTCTTCAGGTCAATTC	7529			
	HEX-TCCAGCATGGCTTCATTCTCCCTTATATG-BHQ1	7501			
*Pvrbp2a*	GGCAAGGCAAATTGTTCAGC	7321	0.999	102.5	<5
	ACGGACATTTCCATTTTCATTTCT	7442			
	HEX-CCTCTTGAATGGTTTCCACTTCCATAATGG-BHQ1	7341			
*Pvrbp2b*	CACAACATGAGGATCAGTCACAAC	8468	0.997	98.9	<5
	ATACCTGAAAAGACAGATAATCCCAAA	8561			
	HEX-AATTCCTYCTGCAAGGCGAACTCGAC-BHQ1	8532			
*Pvrbp2c*	CGCATGACGATACGCATGAC	8414	0.999	102.8	<5
	CCTGCTAAACGAGTCTTTCCAATT	8495			
	HEX-TTTGCCGTTGAATCCCTTCCASTTTGT-BHQ1	8465			
*Pvrbp2p1*	TTGTTTCTACTTGTGAAGAAGCCAAA	1622	0.998	100.0	<5
	CATCATAACTYTGTTTAACTGATGCAATT	1747			
	HEX-TAGAATCCTTGCGTACYTCGAGCACCG-BHQ1	1655			
*Pvrbp2d*	CAGAAGAGAAACTTTAGAAAGTGACCT	7402	0.999	100.8	<5
	CTTCACTTAATGTATTCGCATTTAATT	7487			
	HEX-TTTCACATTTGTGTCCTCTWCATGTCCTTT-BHQ1	7459			
*Pvrbp2e*	CGACTGCTACAAACATATTAGATAACACA	4336	0.999	101.1	<5
	TCTGAGCTACCGCTTCTTCTAATAATT	4446			
	HEX-TGATTTCTCAAGTTCCTTTGCCGCCTC-BHQ1	4415			
*Pvrbp3*	CTGGTGACAACCATTCTGATGAC	8413	0.999	97.3	<5
	AAATCGGCTTCTTGTGATTCATG	8599			
	HEX-TTAATGATTGCGAATCCAGCACTTGAGC-BHQ1	8566			

a The sequences of primers and probes are shown from 5′ to 3′.

b The position of the 5′ end of each oligonucleotide is relative to the start codon in the Sal-1 gene sequences, except for *Pvrbp2e*. Primer and probe locations of *Pvrbp2e* are based on the VTTY57 isolate.

c Eff, PCR amplification efficiency.

### PvRBP antibody levels in malaria patients.

**FIG 4:**
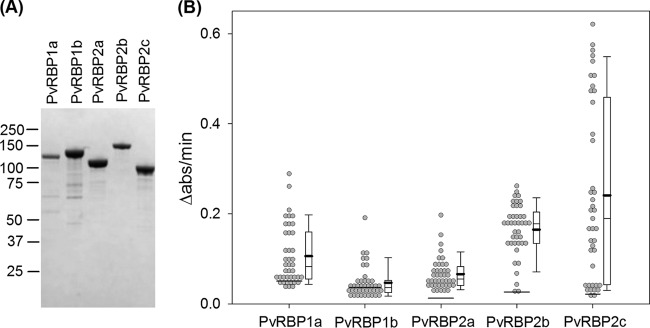
(A) Coomassie-stained SDS-PAGE of recombinant PvRBP used in ELISA of PvRBP1a (118 kDa), PvRBP1b (133 kDa), PvRBP2a (114 kDa), PvRBP2b (153 kDa), and PvRBP2c (94 kDa). Also indicated are the locations of protein molecular mass markers (kDa). (B) IgG responses to recombinant PvRBPs in plasma of 41 P. vivax patients. The horizontal lines across data points indicate the response of the naive control. The whiskers on the box plots indicate the 10th and 90th percentiles. The thick black lines mark the mean values.

We expressed recombinant fragments encoding the N-terminal domains of PvRBP1a, PvRBP1b, PvRBP2a, PvRBP2b, and PvRBP2c (Fig. 4A) in E. coli and used ELISA to measure their plasma IgG levels in 41 P. vivax patients (see Table S2 in the supplemental material). All the plasma samples were collected from a single malaria clinic in a village near the Thai-Myanmar border where malaria was endemic. The rate of absorbance change due to peroxidase conjugated to the secondary antibody (Δabs/min) was used as a proxy for the antibody level. We found that IgG levels against these five proteins were generally correlated (Table 4). The level of PvRBP1b antibodies was most weakly correlated with those of other antibodies. This is likely due to its low value; many patient plasma samples had a PvRBP1b level similar to that of the naive control (Fig. 4B). On the other hand, the distribution of anti-PvRBP2c levels was particularly broad and had some very high values. The median and mean of antibody to PvRBP2c were greater than those to PvRBP1a, PvRBP1b, and PvRBP2a (*P* < 0.015 [one-tailed signed test for medians]; *P* < 0.0024 [one-tailed paired *t* test for means]). Interestingly, although the distributions of IgG against PvRBP1a, PvRBP1b, PvRBP2a, and PvRBP2c were positively skewed, that of anti-PvRBP2b IgG was more symmetrical, with elevated mean and median.

**TABLE 4:** Correlation coefficients between IgG levels against different PvRBPs

Antibody	Correlation coefficient			
	Anti-PvRBP1a	Anti-PvRBP1b	Anti-PvRBP2a	Anti-PvRBP2b
Anti-PvRBP1b	0.357^*a*^			
Anti-PvRBP2a	0.632^*b*^	0.267		
Anti-PvRBP2b	0.426^*b*^	0.130	0.509^*b*^	
Anti-PvRBP2c	0.544^*b*^	0.397^*a*^	0.786^*b*^	0.524^*b*^

a Statistical significance at the 0.05 level.

b Statistical significance at the 0.01 level.

Anti-PvRBP IgG levels in patient plasma were positively correlated with age (Table 5) and likely reflected cumulative exposure to P. vivax. Negative trends were observed between antibody levels and parasitemia, but the relationship reached statistical significance only for anti-PvRBP2b antibodies. This negative correlation remained significant (*P* = 0.007) after controlling for age by multiple linear regression. No association was found between IgG levels and gender, body temperature, pulse rate, or hemoglobin content (data not shown).

**TABLE 5:** Correlation coefficients between IgG levels and age or parasitemia

Parameter	Correlation coefficient				
	Anti-PvRBP1a	Anti-PvRBP1b	Anti-PvRBP2a	Anti-PvRBP2b	Anti-PvRBP2c
Age	0.317^*a*^	0.376^*a*^	0.542^*b*^	0.314^*a*^	0.402^*b*^
Parasitemia	−0.012	−0.244	−0.205	−0.403^*b*^	−0.263

a Statistical significance at the 0.05 level.

b Statistical significance at the 0.01 level.

## DISCUSSION

Despite the importance of RBL proteins in Plasmodium invasion of red blood cells, PvRBPs have remained underexplored. This is likely due to limited access to live parasites to generate materials for molecular analyses and the absence of a long-term *in vitro* culture system. In this study, we used blood from P. vivax malaria patients in Thailand to systematically characterize the family in terms of gene models, expression, and immune responses.

Because the gene models of PvRBPs had been incompletely annotated, we completed the task by sequencing their cDNAs. These represent the first full-length sequences of any *Pvrbp* cDNA. The results confirm the predicted two-exon structure (13) and remove uncertainty regarding additional small introns that may have been missed by previous intron-searching algorithms. We confirmed the presence of one truncated gene, *Pvrbp2p1*, whose encoded protein is equivalent to the N-terminal portions of the full-length PvRBPs. While this gene retains the 5′ splice site, it lacks the transmembrane-encoding sequence. As such, the encoded protein has some similarity to PfRH5, an essential invasion ligand of P. falciparum. Whether the protein has a similar function remains to be demonstrated.

As an attempt to select essential *Pvrbp* genes, we examined the presence of these genes in multiple field isolates by PCR, hoping to be able to disregard those that were deleted in some isolates. However, all the genes, with the exception of *Pvrbp2e*, were maintained in all 72 lines tested. We were thus unable to use this criterion to downselect important genes.

To study gene expression, we developed new probe-based qPCR assays to quantify *Pvrbp* transcripts. These assays are sensitive, reaching the detection limit of less than 5 copies per reaction. Applying these assays to parasite samples from patients, we found that *Pvrbp* transcript copy numbers had high sample-to-sample variation, similar to those of *Pfrh* and *Pfebl* genes of P. falciparum field isolates (23–25). However, whether different PvRBPs play interchangeable roles and constitute multiple invasion pathways remains to be directly demonstrated by invasion inhibition assays (26). Our qPCR assays will be useful for future studies, as they enable researchers to explore invasion ligand transcription diversity and may help researchers tease apart the effects of interventions such as invasion-blocking antibodies.

Using plasma from adult malaria patients from western Thailand, we found a mild but statistically significant correlation between antibody levels of different PvRBPs. Because these antibody levels are associated with age, they likely reflect cumulative exposure to the parasite, similar to previous findings on different fragments of PvRBP1a from Brazil (27). Consistent with our findings, a recent study exploring potential immunodominant B-cell epitopes revealed that the N-terminal fragment of PvRBP2c is highly immunogenic, while that of PvRBP1b is the least immunogenic among PvRBP antigens tested (28).

Previous analyses of several *Pvrbp* sequences have revealed evidence of positive diversifying selection (14, 29). These studies found a strikingly high degree of polymorphism in *Pvrbp2c* relative to other *Pvrbp* genes. For example, Rayner and coworkers reported that *Pvrbp2c* is >25-fold more diverse than *Pvrbp1a* (14). Our data offer a plausible explanation. We found that the IgG responses against PvRBP2c have a greater mean and a broader distribution than those of other PvRBPs. This parallel between genetic diversity and antibody levels is consistent with host immunity being the diversifying force; the high antibody pressure may be driving PvRBP2c sequence diversity, which in turn induces variation in the host response. Interestingly, PvRBP2c was the only PvRBP detected in the previously published schizont proteomics of P. vivax (30). This protein may thus be a highly, if not the most, abundant member of the PvRBP family, another factor that may contribute to its heightened immunogenicity. The biological reason for PvRBP2c's extreme polymorphism is not known. It is possible that the protein has an important role in reticulocyte sensing (13) and needs sequence diversity for immune escape. Alternatively, it is possible that the protein is not directly involved in host cell sensing but functions as a decoy to divert the host immune system from other proteins.

In addition to PvRBP2c, PvRBP2b also appears to be unique among PvRBPs. Unlike other PvRBPs whose antibody distributions are positively skewed, the IgG response to PvRBP2b is more symmetrical, with a relatively high mean and median compared to PvRBP1a, PvRBP1b, and PvRBP2a. While we do not understand the reason for this, we suspect that the antigen is under unique selective and functional pressures. A mild but statistically significant negative correlation was observed between parasitemia and IgGs to the protein, suggesting that the antibodies may interfere with invasion. However, as we have analyzed only patient plasma, how much these antibodies contribute to naturally acquired immunity against P. vivax malaria remains unknown. Further investigation using a more powerful study design, such as one that utilizes a cohort (31, 32), would help address the role of anti-PvRBP antibodies in acquired immunity. Direct assessment of the invasion-blocking ability of antibodies to PvRBPs, alone or in conjunction with PvDBP antibodies (33, 34), will be an important step in determining their potentials as candidates for a P. vivax blood-stage vaccine.

## Supplementary Material

Supplemental material
