# Supplementary material for: Gene Models, Expression Repertoire, and Immune Response of Plasmodium vivax Reticulocyte Binding Proteins
Source: Infect Immun. 2016 Feb 24;84(3):677–85. doi: 10.1128/IAI.01117-15 (PMC4771344; doi:10.1128/IAI.01117-15)
Supplement: Supplemental material [file supp_84_3_677__index.html]

Gene Models, Expression Repertoire, and Immune Response of Plasmodium vivax Reticulocyte Binding Proteins — Supplemental material 

# Gene Models, Expression Repertoire, and Immune Response of Plasmodium vivax Reticulocyte Binding Proteins

## Supplemental material

- Supplemental file 1 -

  Table S1. Transcript abundance of *Pvdbp* and *Pvrbps* in 15 *P. vivax* patient blood samples. Table S2. Antibody levels against each recombinant PvRBP in 41 *P. vivax* patients. cDNA sequences of *Pvrbp* genes in the GenBank Flat File format. Alignment of *Pvrbp* cDNA sequences against those of the Sal-1 reference strain.

  PDF, 5.5M
