# Supplementary material for: Gene Models, Expression Repertoire, and Immune Response of Plasmodium vivax Reticulocyte Binding Proteins
Source: Infect Immun. 2016 Feb 24;84(3):677–85. doi: 10.1128/IAI.01117-15 (PMC4771344; doi:10.1128/IAI.01117-15)
Supplement: Supplemental material [file IAI.01117-15_zii999091596so1.pdf]

Supplemental Material for "Gene models, expression repertoire and immune response of *Plasmodium vivax* Reticulocyte Binding Proteins" (Hietanen, Chim-Ong, et al.)

This supplemental file is organized into 4 sections, including

1. Table S1. Transcript abundance of *Pvdbp* and *Pvrbps* in 15 *P. vivax* patient blood samples (page 2)

2. Table S2 Antibody levels against each recombinant PvRBP in 41 *P. vivax* patients (page 3)

3. cDNA sequences of *Pvrbp* genes in the GenBank Flat File format. ID of the parasite isolate from which a segment of sequence was derived is listed as misc_feature (page 4-33)

Pvrbp1a	(page 4-7)
Pvrbp1b	(page 8-10)
Pvrbp2a	(page 11-13)
Pvrbp2b	(page 14-16)
Pvrbp2c	(page 17-19)
Pvrbp2p1	(page 20)
Pvrbp2d	(page 21-24)
Pvrbp2d$_{tr}$	(page 25-27)
Pvrbp2e	(page 28-30)
Pvrbp3	(page 31-33)

4. Alignment of *Pvrbp* cDNA sequences above against those of the Sal-1 reference strain (page 34-169).

   For *Pvrbp2e*, which is absent from Sal-1, sequence alignment was made against the sequence of the Brazil-1 strain.

   Alignment was generated by ClustalW (www.genome.jp/tools/clustalw/). Predicted coding sequences was obtained from www.plasmodb.org/ for Sal-1 and www.broadinstitute.org for Brazil-1 on 16 November 2015.

TABLE S1 Transcript abundance of *Pvdbp* and *Pvrbps* in 15 *P. vivax* patient blood samples. Values were normalized by that of *Pvama1*.

Sample	Gene									
	Pvdbp	***Pvrbp 1a***	***Pvrbp 1b***	***Pvrbp 2a***	***Pvrbp 2b***	***Pvrbp 2c***	***Pvrbp 3***	***Pvrbp 2e***	***Pvrbp 2p1***	***Pvrbp 2d***
1	0.218	0.179	0.170	0.074	0.206	0.545	0.168	0.984	0.145	0.528
2	0.156	0.960	0.670	0.783	0.420	0.642	1.540	1.968	0.284	0.519
3	0.277	1.220	0.580	0.956	1.684	1.371	2.702	1.014	0.443	0.565
4	0.325	0.244	0.137	0.145	0.449	0.870	0.297	0.879	0.285	0.478
5	0.251	0.239	0.161	0.207	0.128	0.865	0.213	0.744	0.373	0.550
6	0.173	0.914	0.940	1.389	0.457	3.131	1.827	0.815	1.092	0.578
7	0.161	3.948	3.562	15.43	5.682	3.811	9.147	5.589	0.441	0.856
8	0.129	2.486	1.232	0.895	2.033	0.896	2.920	2.394	0.278	0.880
9	0.325	0.438	0.211	0.339	0.470	1.745	0.968	1.554	0.593	1.272
10	0.194	0.413	0.286	0.336	0.689	1.075	0.758	1.079	0.389	0.827
11	0.819	9.569	5.492	4.446	0.821	35.42	16.72	1.462	18.38	3.003
12	0.376	0.530	0.412	0.556	0.855	1.274	0.798	1.044	0.297	0.577
13	0.306	0.201	0.121	0.346	0.399	0.840	0.397	0.516	0.285	0.467
14	0.372	1.275	4.740	1.295	0.638	1.843	2.436	0.797	1.054	1.393
15	0.284	3.051	2.070	0.807	1.037	9.881	3.046	2.550	1.226	1.663

TABLE S2 Antibody levels against each recombinant PvRBP in 41 *P. vivax* patients.

Sample	Age (yr)	Parasitemia (%)	**Antibody level** (Δabs/min)				
			**PvRBP1a**	**PvRBP1b**	**PvRBP2a**	**PvRBP2b**	**PvRBP2c**
1	50	0.06	**0.208**	0.113	0.106	0.176	0.575
2	37	0.07	0.033	0.021	0.031	0.132	0.022
3	24	0.08	0.038	0.034	0.055	0.183	0.377
4	24	0.2	0.056	0.025	0.067	0.133	0.026
5	19	0.05	0.061	0.026	0.039	0.183	0.041
6	32	0.02	0.088	0.047	0.097	0.219	0.551
7	29	0.03	0.044	0.034	0.047	0.138	0.039
8	28	0.29	0.178	0.042	0.046	0.093	0.117
9	35	0.02	0.058	0.042	0.038	0.150	0.120
10	18	0.01	0.089	0.020	0.039	0.196	0.140
11	25	0.22	0.191	0.016	0.065	0.236	0.448
12	46	0.01	0.177	0.046	0.088	0.224	0.539
13	25	0.04	0.071	0.031	0.025	0.029	0.029
14	18	0.09	0.048	0.027	0.030	0.138	0.167
15	34	0.16	0.043	0.015	0.055	0.180	0.212
16	22	0.01	0.062	0.019	0.057	0.188	0.041
17	47	0.04	0.076	0.017	0.132	0.195	0.509
18	20	0.05	0.054	0.033	0.048	0.067	0.189
19	66	0.01	0.157	0.191	0.153	0.244	0.621
20	24	0.07	0.099	0.028	0.087	0.232	0.471
21	46	0.02	0.196	0.090	0.048	0.247	0.162
22	55	0.33	0.046	0.036	0.049	0.119	0.129
23	18	0.16	0.057	0.021	0.037	0.193	0.034
24	39	0.03	0.121	0.039	0.065	0.225	0.231
25	24	0.04	0.113	0.025	0.087	0.159	0.243
26	26	0.05	0.198	0.083	0.087	0.222	0.475
27	34	0.02	0.049	0.058	0.042	0.177	0.170
28	42	0.14	0.051	0.041	0.035	0.195	0.083
29	24	0.03	0.162	0.055	0.072	0.212	0.362
30	39	0.14	0.261	0.030	0.118	0.177	0.484
31	32	0.21	0.082	0.054	0.047	0.086	0.044
32	25	0.38	0.121	0.014	0.040	0.026	0.015
33	45	0.07	0.101	0.043	0.070	0.160	0.204
34	45	0.07	0.096	0.034	0.070	0.181	0.033
35	55	0.07	0.289	0.050	0.197	0.262	0.505
36	60	0.16	0.072	0.023	0.062	0.150	0.256
37	18	0.04	0.043	0.039	0.028	0.043	0.040
38	25	0.17	0.061	0.113	0.046	0.145	0.140
39	36	0.09	0.177	0.097	0.069	0.130	0.563
40	23	0.02	0.153	0.104	0.078	0.178	0.252
41	38	0.01	0.061	0.041	0.054	0.135	0.216
naive	35	-	0.050	0.036	0.012	0.026	0.022

```
LOCUS       Pvrbp1a                 8502 bp    mRNA    linear   INV 09-NOV-2015
DEFINITION  Reticulocyte binding protein 1a cDNA, full length CDS.
ACCESSION   Pvrbp1a
VERSION
KEYWORDS    .
SOURCE      Plasmodium vivax (malaria parasite P. vivax)
  ORGANISM  Plasmodium vivax
            Eukaryota; Alveolata; Apicomplexa; Aconoidasida; Haemosporida;
            Plasmodiidae; Plasmodium; Plasmodium (Plasmodium).

COMMENT
FEATURES             Location/Qualifiers
     source          1..8502
                     /organism="Plasmodium vivax"
                     /mol_type="mRNA"
                     /db_xref="taxon:5855"
     misc_feature    1..335
                /note="isolate VKBT95"
     misc_feature    336..1374
                /note="isolate VTTY57"
     misc_feature    1375..1841
                /note="isolate VKBT99"
     misc_feature    1842..3849
                /note="isolate VTTY57"
     misc_feature    3850..4809
                /note="isolate VKBT99"
     misc_feature    4810..6823
                /note="isolate VKBT95"
     misc_feature    6824..7827
                /note="isolate VKBT99"
     misc_feature    7828..8502
                /note="isolate VKBT95"
BASE COUNT     3556 a   1151 c   1866 g   1928 t      1 others
ORIGIN
        1 atgaaaaggg ggatttgcct agcagcgctt ctttgcctgt tcaactattt aggtgcaggg
       61 cacggagaaa atgcagaaga agacataagg aatagcgaag ggaaagtaaa cttcttttcg
      121 ctggattcta atttaaagaa aaataaaaag tcgaaacata atagggtgaa aagaaggaac
      181 gcgaaaattt cgaactttct aagccaaaaa gcgtatgtta aagaaacaga taacgcaagt
      241 ggaaaagacg ctgaaggatc acgtccgtct catgattcat cgtttgttaa cttaaacggt
      301 catgtcgatg gaaaaagttt atcatacagc gttcatgtaa aagaaagtac accacatagt
      361 actacgcggg agagcacgga aaagggaaaa gaaaatgaga aaatacaggg tgtcctaagc
      421 tcttttgtgc aaagccaaga aggggagaa tcagacgatt tggagggaaa gtacaattct
      481 atagttgata tgagcgagaa actgaaagaa aatgataagt ataacctagt gtttgacatg
      541 gagattgatt ttgttgactt acaatttttt aatctaattc tggagctgat tccaaaggac
      601 agtaaatacc atacctacta tgaggaaaca cttaaacagc aagttacaga atacacaaat
      661 tcgttgaaaa cgttaatgga ttcatgcata tctgaaaaag atcaaatgat catattagag
      721 tatgagatta attatgccaa gaggaagtcc atagaaacag agacgttggg tgataaggaa
      781 acaaaactct cagcagtatc tcaagcctat gcaatacatc tagaatcgta taaggagta
      841 ttaaagccca aattaaatga tataaaaaat aatgcctttt cagttttgaa ggattcctac
      901 tgtaaggata actgtgggga atatgtgcag aaatataata caatgcgcaa gaattttata
      961 tcaagttcag atcagtataa aatggaagca tatatgtaca ttccgaagag cattaacgat
     1021 tatacagtgc tagataaaat tttatcagaa tctaacgaac taggtataga cattcaagaa
     1081 aatgttaatt cgctgaagct gttaggagaa gaaattagtg aagtttccca tttgtatgtc
     1141 attaacagta cgttaattga cgatgcagcc aaaaaattag aatccataaa tgaagaggac
     1201 gaatctgcag aaatagattt acaaaaattt gaagataatt ccaaagcgct agcaaacaac
```

1261 tactgcattt ttcaatacat aaaaacgctt aacgaaccaa ttaagaaagc atatgaaagt
1321 aaggtaataa aatctaatga actactgtct accattatag acacgttagg aaaaagtgct
1381 actgcgctgc aggagtcgac atttaatcaa gaggagtgca ataaaattaa aaccgaagcg
1441 gaaaaagtaa aagatgacgc tgaagatata tacgaaaaaa atgaacagat atactacgaa
1501 attccagaaa gtgaggacga aacaatagat gataagataa acgatcttca gtaccttata
1561 gaccaaatga aagattataa agatgaaata gtaaacaatt cggagttcat ttctaataga
1621 tataaaaata tatacgaaaa cttaaaagaa acgtatgaaa cggaattaaa tgatatagga
1681 caactagaaa acgacacatc aaaagtaaat ttttatttga tgcaaataag aaaaattaac
1741 actgagaaaa ccaaaataga tgaaagttta caaacagtgg aaaaatttta caaagaaata
1801 ttagatttga aagaaaaaat atatgaactg aagatagagt ttgaaaaaag cgtcacagaa
1861 ataaatcgat tacaggatgg ggaatctgcg cgtgatttac atgaagaaca gatcaaagag
1921 atattagaca agatggcgaa aaaggtgcat tatttaaaag aacttctctc cttaaaaggg
1981 aaatcaagtg tgtacttcac cgaaatgaat gaattgctca acacagcatc gtatgacaat
2041 atggaggggt ttagcgctaa gaaggagaag gcggacaatg acattaatgc tctatataat
2101 tctgtgtata gggaagatat aaatgctctc atcgaagaag ttgaaaaatt tgtaaccgaa
2161 aataaagaaa gcactctgga gatgctaaaa gatgaagaaa tggaagagaa gctacaggat
2221 gccaaagaaa cttttgctaa attaaatttt gttagcgatg acaagctgac agatgtgtac
2281 accaaaatga gtgcagaagt gactaatgct gaagggatca aaaaagaaat tgcgcaaaaa
2341 caattcgaaa atgttcataa aaaaatgaaa gaattttcgg atgcgttttc cacaaaattt
2401 gaagcgttgc aaaacagcat gcagcagtac aatcaggagg gagacgcgat agaaaagcat
2461 aagcaaaata gatccgaaaa ggaggaggaa tattttaaaa atgaaagcgt agaagaagat
2521 ctatccaggg aagaaacaga agagcaagaa tacactaaac ataagaataa tttttccaga
2581 cggaaaggtg aaatatcagc agaaattacc aacatgaggg aagtcataaa taaaattgag
2641 agccaattaa actattacgg ggttattgaa aaatatttct ccctcattgg cgatcagaat
2701 gaagtgtcaa cggctaaagc gttaaaggaa aagattgtta gtgatagttt gagggacaaa
2761 atagaccagt acgagacaga attcaaagag aaaaccagtg cagtggaaaa taccgtctca
2821 actattcaaa gtttaagtaa ggcaatagat tcacttaaac gtttaaatgg tagcataaac
2881 aactgcaaaa aatataacac agatattgat cttttgagga gcaaaataaa aactctacga
2941 gaagaggtac aaaaggaaat tgccgaaacg gagggcgata aagtggtggg cgaaaatacc
3001 actgcgttac ttttaaaaag cttaagagac aaaatgggaa agataaacga gaaattgaac
3061 gatggtagac taaacagttt agacactaaa aaagaagatc tgctgaagtt ttactcagaa
3121 tcgaagtcga aaattcattt gagcaaagac caaaaggggc cacaagatcc gttgaataga
3181 attgacgaat gggaagacat caaaagggaa gtagacgaac taaatgttaa ttaccaggtg
3241 attagcgaaa ataaagttac gctatttaaa aataactctg tcacttatat agaggcaatg
3301 catagccata taaatactgt tgctcatgga ataacgagca ataagaatga aattttgaag
3361 tccgtaaagg aagtagaaga taaactaaac ctcgttgaac agaatgaaga ttacaaaaag
3421 gttaaaaatc cagaaaatga gaagcaatta gaagccataa ggggatccat gagcaagctc
3481 aaggaagtga tcaacaaaca cgtcagcgaa atgacccaac tggagagcac agcaaacact
3541 ttaaagagca atgccaaggg aaaggaaaac gaacacgatc tggaagagct aaataagaca
3601 aagggacaaa tgagggatat ttatgaaaag ttgaagaaga tagcggagga actaaaggaa
3661 ggaacagtaa acgaattgaa ggatgcaaat gaaaaagcaa acaaagtaga attagagttt
3721 gagaggaaca ttattgggca cgttttagaa cggattacag tagaaaagga caaagctgga
3781 aaggttgttg aagaaatgaa ttctctcaaa actaaaattg aaaaattgat acaggaaaca
3841 agtgatgatt cacaaaatga attagtcaca acgagtatta caaaacattt agagaatgca
3901 aaggggtatg aggatgtaat aaaacgaaat gaagaagatt caattcagtt gagggagaag
3961 gcgaaaagtc tggagacatt ggatgaaatg aaaaaactag ttcagcaggt taacatgaat
4021 ttgcaaagtg ctatacaagg caatgctggt ataagcaaag agctgaatga gcttaaaggc
4081 gttatcgaat tgttgatatc aacgaattat agcagcattt tagaatatgt aaagaaaaat
4141 tccagcgagt ctgtccgttt tagtcagcta gccaatgggg aatttacaaa ggctgaaggt
4201 gaagagaaaa acgcaagtgc cagatttgcg gaggcagaga agttaaagga acaaattgtc
4261 aaagatttag actacagtga catagatgat aaggtaaaaa aaattgaggg aatcaaaaga
4321 gaaattttaa agatgaaaga aagtgcacta acattttggg aagagtcaga gaagtttaaa
4381 caaatgtgct cttcacatat ggaaaatgct aaagaggggа agaaaaaaat tgagtattta
4441 aaaaataatg gggatggagg aaaggccaac ataacggata gccaaatgga ggaggtaggt
4501 aactatgtta gcaaagctga gcacgccttt cacacagtag aagcacaggt agacaaaact

4561 aaagcctttt acgaatccat cgtagcttat gtaacgaaga tggacaacct gtttaacgaa
4621 tcgttaatga aagaagtgaa agtgaagtgt gaaaaaaaga atgatgaagc ggagcaaata
4681 ttcggccaaa ttaaaaccgt agatggtaga attaaagcgc gagtgagtga gaatgaaaga
4741 aaaataagcg aattgaagga aaaagccaaa gttgagaaaa aggaatcctc gcaacttaac
4801 gatgtttcca cgaagtcgtt attacaaata gataattgca gacaacagct tgacagcgtt
4861 ttgtcaaaca ttggaagggt gaaacaaaat gcacttcaat atttcgattc ggctgataaa
4921 tcgatgaagt ccgttttgcc tataagcgaa ttgggtgccg aaaaatcgct agacaaagta
4981 aaagcggcta aggaaagtta tgagaaaaat ttggaaaccg ttcaaaatga aatgagtcgt
5041 attaatgtgg aagaaggaag tctgaccgac atagacaaaa aataaactga catagaaaat
5101 gaattgctaa aaatgaagaa acaatatgaa gaagggttac tacaaaagat taaggaaaat
5161 gcggataaga ggaagagtaa tttcgaatta gtaagaagcg aaataaacgc cttgctggat
5221 ccaagcacgt ctatttttat taaattaaaa ttaaaggaat atgacatgac cggcgattta
5281 aaaaattacg ctgttaaaat gaatgaaatt catggtgaat ttaccaaatc gtacaatttg
5341 atagaaaccc atttgtccaa tgctacagat tattctgtga cgtttgagaa ggcccaaagt
5401 ttaagggaac tagcagagaa ggaagaagaa catctcagaa gaagagagga ggaagcgatc
5461 aaactgctga atgatattaa aaaggtggaa tcgttaaaac tgctaaaaga aatgatgaaa
5521 aaggtgagtg ccgaatatga aggtatgaaa agagaccata cgagtgttag tcagcttgta
5581 caggatatga agacaattgt tgatgagctg aaaacactga atgatataag cgaatgttcg
5641 agcgtgctaa acaatgtagt tagtatagtt aaaaaggtta aagagtcgaa acatgcagac
5701 tataagagag acgcgaatag catgtatgaa agtatggtaa ctctggcaaa ttatttccta
5761 agcgatgagg ctaaaatttc atcaggaatg gaattcaatg cggaaatgaa atccaatttt
5821 aagacagact tagagttgga gatcttttcg gtaatatcca attcgaatga acttttaaaa
5881 aaaatagaac aggattcaaa tgatgtaata caaaaggaaa gagaaagtga acagctagct
5941 aaggatgcca ctgatattta taatgtgata aaactaaaaa atgaatttaa cgaaaagttg
6001 gaagaagcca agaacaaaga agaggtcgtt tcggaaaagg taagggaagc attgaagaga
6061 ctgagtcaag tggaaggaat aagatgccac tttgaaaatt tccataggtt actagacaat
6121 acagaggagc ttgaaaattt aaaaaaaatg gttaccatct accgggataa gaaaagcgaa
6181 gcgccgaaag aatcagggtt gcaagaaatg gagaacgaaa tgaacaccta tagtaactcc
6241 ataacacagt tagaaggaat agtcgtatca gccggggaat ccaaagaaga cattgaaaaa
6301 ttggaaagat ccaatgaaga aatgagaaat attagcgaaa agataagcac aatagattcg
6361 aaggttatcg aaatgaactc aactattgat gagttgtaca aattgggaaa aaactgtcag
6421 gcccactgga tttccctgat cagttatact gcgaatatga aaacgtccaa aaagttaata
6481 atgattaata aagaaaagga gaacacggaa aagtgtgtag actacattaa agacaattcg
6541 tcctccacag acggttatgt cgaaacgttg aaagggtttt acggtagtaa gcttacattt
6601 agcagcgcat ccgaaattgt gcaaaatgca gacacatatt ccgtgaattt tgcgaaacac
6661 gaaaaggagt ctttaaatgc gataagggat ataaaaaagg aattatattt attccaccaa
6721 aatagcgata ttagcattgt agagggaggc gtccaaaatg tgctggcact ttatgataag
6781 ctgaacgagg aaaaaagaga aatggatgaa ctgtacagaa atataagtga aactaagctg
6841 aagcaaatgg aacacagcac tgacgtgttt aagcccttga tagaattaca caaaggattg
6901 attgaaacga ataataagtc tttgctggag aaagaaanga acttaaaaag cgtgaacgac
6961 aatatgcaca gtatggaagc tgagatgatt aaaaatggcc ttaaatacac cccagaaagt
7021 gtgcaaaata ttaacaacat atacagcgtt attgaagctg aggtgaaaac gctggaagaa
7081 attgaccgtg attatggcga taattaccaa atcgtggaag agcacaagaa gcaattttcc
7141 attttaatcg acagaacgaa cgcgctaatg gatgacattg aaatttttaa aaaggaaaac
7201 aattacaatt taatggaagt aaacacagaa accatacaca gagtaaacga ttatatagaa
7261 aagatcacca ataagttagt acaagccaaa acggagtatg aacagatcct ggaaaatata
7321 aaacaaaacg atgatatgct acagaatatt tttctcaaaa aagtaagtat tatcgaatac
7381 tttgaaaacg taaagaagaa aaaagaatct atattgaacg atttatatga acaggaaagg
7441 ctgctcaaaa taggggaaca cttagatgag attaagcgta acgtgacaga gacactgagt
7501 agttatgaaa ttgatcaaaa aatggaaatg atgtctaaaa accttctaga gaaaaaaatc
7561 aaaatgatga attacacttc catttatgag ttggagagag aagcgaacga aataaacagg
7621 gatgctaaac aaataaaaga tgatgataca atacttaata gtgttttaga agctgccatt
7681 cagaagaggg gagatatgga cgcgatcttc agtcagatgt ctgcagatag aaatccaaat
7741 gaatataaat ctgcagagaa gtacatgaat gaagcgaatg aaataattcg ccaattagaa
7801 gtaaagcttc gagaaatagg ccaactagta caggatagtg aaagcatatt atcagaaatg

```
7861 aattctaaaa agagcgctat agaaaaggaa aagacagcta gggctttgag aactagtgaa
7921 aataatagaa gagaagagga agaaagggca cgtgtgcaag aaatgagcat gaataatgat
7981 cccacgcaaa gtgaaacaac tcattcggag ggatccatcg gtgaaggaaa agaatcagac
8041 tcggatgaaa caggtttaac acacgatgca ggtgcagatg aagattctac tagctcggca
8101 aaaggggcac atgaattaga agaggaggaa actacagcac ctatggaaga aaccgagatg
8161 aatgacaata cccttctagg gtatgacact accagaagtg atgaacctga tatgcataca
8221 gagaacaccc aggatggtac ctatcaggat acgtcaaatt ccagcgatga agcagatatt
8281 ttgaacggca agttcaattt taataatgtc aaatatgcag gagcattcgt tttactgtgt
8341 acgagcgcag ttataggggc tattatagca cataagaaag atgaccaaga ggagcttaat
8401 aatggcgtgg aagacgatag ggtgtttgaa gttaaaaaga gcatgaaccc agaaaacaaa
8461 gaagaaatca ttgatgtttc ttttgttgat attgagtatt aa
//
```

```
LOCUS       Pvrbp1b                 8619 bp    mRNA    linear   INV 09-NOV-2015
DEFINITION  Reticulocyte binding protein 1b cDNA, full length CDS.
ACCESSION   Pvrbp1b
VERSION
KEYWORDS    .
SOURCE      Plasmodium vivax (malaria parasite P. vivax)
  ORGANISM  Plasmodium vivax
            Eukaryota; Alveolata; Apicomplexa; Aconoidasida; Haemosporida;
            Plasmodiidae; Plasmodium; Plasmodium (Plasmodium).
COMMENT
FEATURES             Location/Qualifiers
     source          1..8619
                     /organism="Plasmodium vivax"
                     /mol_type="mRNA"
                     /db_xref="taxon:5855"
     misc_feature    1..8619
                     /note="isolate VTTY57"
BASE COUNT     3629 a   1146 c   1840 g   2004 t
ORIGIN
        1 atgaagaagc caatttatag gacagccctc tttggtctat ggcttgcatc tcttgattta
       61 aatcgaggga aaaacacgtt gcataatata aagaaacatt ttgaagactc gaaattttta
      121 tcattcgatc cgaatttgaa gagaaacaaa aagttcaagc ataaggacgt gaaaaagaag
      181 agctttaaaa gtctggaaac agatagtgta cagcagtttg ataaagaaat tagcaaagtt
      241 aatagggagg acccaacagg aataatcgaa tctgatagtg cttcatttat aagcttagga
      301 aatgctagtc tgaaaaatga accctctgac ggtggtggag atgaagtggt tttaagaacc
      361 gacggagggg gtagtgacac cgataatgat tcaggcaaat tggagaaaag tgaaatacct
      421 gatgtggaga acgcagaatc ggtatcgaaa tataaaatcg ttttagaaga ttatgtaaat
      481 aaactgaaag aatctaatcc atatttcttg atggaggttg attatgttaa tctgcagtct
      541 tttaaagaaa ttaggaacct cattccagag gggaaagaat accacaagtt ctacgatgag
      601 gagatggata aaaaagtcac agattacaca aatagattag acgcactgat gaaaaagttc
      661 attggagcga aaaatgaaat gataaaaatg gatgctatcg taaaaattca taaaaagggt
      721 tctaccgaat tgaagcaaga agctgaaaat gtaaaaaaat tagaattagc aaaggagcaa
      781 tatgaaaagt acatgaaaga gtataatgat gatataaaac ctattgtaca tgaaattaga
      841 aacaaggcat atgagcattt gaagcaatcc agctgcacgc accagtgtaa tgcttacatt
      901 atgaattatg ctcgtcttct taacaaatat ataaagaaca tttcagagac tacggacgag
      961 tcagttgtca ctatcgttaa aagtattaac gattataatt ctctggacaa aatattagaa
     1021 cttgcagaaa gggagaatat agacattgca gaaaatgtct accttttgaa gttactagga
     1081 gaagaagtaa atgaccttaa ctatgtgtat gtgataaaca gatcgctaat aaatgacgcg
     1141 aataaagttc tgatggacat aaaggaaaat gggattttat ttcatacgaa ggaggataaa
     1201 attgtaaaca gtacaaagga actaatcaac aactattgtg tgtttcacca tataatgatt
     1261 ttgaatgttc caattaaaaa aatgtacgaa gaaaggataa aaaggtctaa cgatttattt
     1321 tctacaatta tggaaaagtt aaaagatgaa gctcataaat taatcaattc tgtatttgtc
     1381 gaggaggaaa gtaataaaat tcttaagtcg tcagaaggaa ttgtccaaaa tgcagaaaaa
     1441 atacttgagg aaaataaaaa aaaaatagac ttttttaaaa ggtatccaga aataaaaacg
     1501 aagcctccta tggaagaatt acaaactgag tttagtcaac aagaagaagc gagggggggaa
     1561 atattaaaca tggcacagct aatcgatcac ttatataata atttgtttca tgtgaacaaa
     1621 actaatcatt taaataaatt aaacgaaata gctcagtacg gggatagcat aacaaaggga
     1681 aatttactgt tggaagaaat ttctgaaatt aataaagagc ggaatgttct aaaggtaaat
     1741 attgctactt tgaaaaatgt gcatgggaat ttggtacgtt cgaaaaataa cattgttgaa
     1801 attgtaataa atgtggacaa cacttctgag gtggacgaat tgaaggaaaa ggaagagaag
     1861 cttgcaaagt acatggaaga aattaacgaa aaaattaaag acatgataaa gaaaatgcac
     1921 aaattaaagg aagtcatcgt tttaagagat aaagcgaatc aagatgtcat cacgattaac
     1981 gagttactga atggagggtc gaatgccaat ttggaagaat tcataagtaa aaagaacaaa
     2041 gccaatgatg atattaattc gatatttaaa acattgtaca gcggagacgt atatgatctt
     2101 atcgaaacgg tgggcacgtt tgttcgtgaa aagagaaaaa ttgtaggcga aacgttttcc
```

2161 gctaaacaaa ttgaccaagt ggaacaatat ctcagcgaag taagagtggc tcatgccaat
2221 ttagacaccc tgactcatga aaaattgata aatgcctatg atcagttaag cgcagaagag
2281 aacaatacag aaaaacttaa aaatgatctt gttaagaaga agagcgaaga tctatacaaa
2341 aaaatggcag actctttgaa aacatttaaa actgaatatg ctgatttggt gagaaatatg
2401 gaggaatacg aaagggaaga aaaaatatta gaacagtgta tgggaaaaac tttgaaaaag
2461 gaggaagaac acttcaacac gttacaagtg gatggagaag ctctgaaaga cacgtacaat
2521 tgcgcaggat tcgaaaaact gaggggcact ttttcgcaga ggaaattcga attgtcacaa
2581 aaaatagacc acgttaaggg tgtggtgact aatatggaga aacttttaaa atcttacagt
2641 gctttggaga agcattttct tttacttgat gatagcgatt acgtaaaacg agtggaaaca
2701 ttaagacaaa ctgttcagcg cgaaatgatg aataaaaaaa tgagtgttca agaggacagc
2761 ttcaagagaa ggactgaatc tgtacttaag accatcaaaa tattcgaaag tttaaacaaa
2821 accatacatt ttcataacca cttaaacagg agcataaacg aatgtgaagg catcaatgta
2881 tcgatcaaca gtttaaaagg caaaacagtc gcgttaaagg gcgaactgca aaaagaaatt
2941 gatgacattg gagcagacca gctgataagt gaagctgtaa aagtggcact actagataag
3001 ttgaaagcag aactaggaag tgtaaacagc aaattagatg aaactcacat ggatgatttg
3061 ctcaaaaaat caacagattt gaataaattc tacagagatt caaaaagcga tttgcacgaa
3121 aagaggcaag aataccctgt gcactattct ctagaaaagg taaatgactg ggaagaaatt
3181 aaaacggaag tccacgaatt aaatgctcat tataatgcat tcaacgacaa taaggctaaa
3241 ctaattttag ccaattcgcg catgtacttg gaatcaatat ataaccttat aaaagagttg
3301 gttcataaca caagggagga gaaagacaaa atggagagat ctctaaaggg aatagaaaaa
3361 agcgtagaaa ctatcgaact gaaccaggat tataaaaacg ctaaagttac ggacaatgag
3421 aatgaaataa aaaatataaa tgaagacact attccaaaaa ttaaggaacg aattgatgag
3481 tgtagcagaa aactgactaa attcgaggaa caatcggata gtttaaacaa caaagacacg
3541 aataaaggaa gtgacgttac tctcttgctt gatgtgataa gacaaatgaa aggtacttat
3601 caagaattaa aaaagatacc tactgagatg ttcaaagaga gggacgaaat aaggaacact
3661 gaagaaaatg taaacaaggt tcaattcgca tacgagagaa aattaattga gcaattttta
3721 aaaagaatta ctcagaagag gaatgaggcg gaagagcaaa cacaacagat cagcgcaatt
3781 gttaaacaaa ttgaagggat aagaaaagaa acgggagatc ctatcgacaa agaattaacc
3841 accacgaact gcgaaaaaca tcttcacgat gctgaagaaa aaggggagga aataaagcaa
3901 attgaagagt tgtccaccaa actgagagga gaagctgcca ctactgatgt cataagtgaa
3961 gtcataaaaa ttaaagaaca ggtttacgca catttggaaa aagccacaag caatagtaac
4021 cacgtaagcg aggcactgaa cacaattaaa gaaatgaaag aactaatact gtcagagagt
4081 tataacagtg ttattaactt tatcttaagg aatgtaaatg atgctcataa atatgtcgaa
4141 ttggtaaaaa tggaattgct aaaaattgaa gatgccactg accatgtgaa gatacgattt
4201 gaggaagcaa agaagttaaa ggaaaaaatc aagacagatg tggatgacga gggggcagat
4261 catatcatca aagaaattga agaaataaga aaagtaattt tgaatgaaat aaaagacaca
4321 agcacatttt taacaaatgc agaaaagggc aaagaaaatt gtcttctaca tttcgagaat
4381 gcaaaggggg gaaaagcaaa atttgattac ttaaaagaac atggtgacgg tgagcacaaa
4441 agaatatcac acagtgagat aaatatggtg gaagaaaata tgacaaaagt gaaacaacac
4501 acggatgaag ctgacaagga tgtggaacaa acgaaaaaat tttattactc tattttgggg
4561 tatgaaaaac taatcaatga tctgctaaac gaatcgctac ttagaaaagt caaattaaaa
4621 tgcgagaaat tgagaacaga agtagacgga ataatggacg aaatggaacg tgtagatgtt
4681 aaggccaagg aagagtccat cgaacaagaa caaaaggtaa aacaaatgaa ggagcaatcc
4741 attgttgagg gtgatcagcc taaagaactt aacgaagagt ctatgaacgc attgctacaa
4801 ataaaaaatt acagacagaa attggacgac gttatgttaa gtataaaaag tgtggaagag
4861 aatatggagc agtttttaag gaatgctggc agttacgtga aacaacgct agacatctct
4921 caactgcgaa atgaaaattc gttcgataat ttgacagcgg cggaggaaaa ctacaaaata
4981 gcattgagaa atgttcagaa tgaaaaacag cgtatgataa gtgaggaaaa aaggctagct
5041 gaaatgtata gggatatcat tagtgtagga aaagaattgg aagaacaaaa aaaaagatac
5101 gaagtgggat tattgaaagg gataaaagag aatgctgaca gaagattgac cgcattggaa
5161 ttaaccaaaa aggaagttaa ctctttggtg gatccgtcca aatccatatt ttttaaattc
5221 aaattggaca acttagacga aacagatata atgaaatatc tatatcgcta tgctgatcga
5281 atttaccata tttttgacga atttgtgaaa ttgtacaaaa tgatagaagg atatttatcc
5341 cagaattcgg acccctctgt aacatttaat gaagtcaaaa tggtgaggga gaaagcgcaa
5401 atggaagaaa tgagccttgg agaaaaggaa gaagagtcaa aagaagtact gagagacatg

```
5461 aaaaaaaagg agtccataag gttgttaaac gaaatgatgg aaatgctgaa cagtgcaaag
5521 gaaagagtta cggaagatca tgcaaaagtc agcaactatg tggagaatgt taaaaaagac
5581 gtcaccgatt taaaggtact ggacgatgta aataacggcg ccaatgtttt taataaggca
5641 atgaatagtg ttaacgaaat taaagacgca aagtatatga accataggaa ggaagcggaa
5701 aatatatacg aaaatatgat acaactggcg aactatttct tagatgacga tgttaggatg
5761 gagtccacgg gaaagttaag cgaagcggca tttgcaaagg cagaaccggg tatagtgtca
5821 gacatttttg gcaaaataaa ggaggcgaag gaaattgtag aaaaaataga agaagagtca
5881 ataggtatac aaaataaaca aatagaaggg gaaaggctct ccactgaggc gaaccacatt
5941 tacagcgtgg caaagctgaa aaatgaattt aaaaacaaaa agaatgaagc gaaacttaag
6001 gtcatattcg tgctagccga aatagaggaa gcattacata aattaaaaag tgtgaataaa
6061 gtaaaatgcc attatgataa ttatgataat tataatgata tactagaaaa taacgaagaa
6121 catgagaatt tcaagcaaat ttcttctacc tacaagttga agaaggccca aatagcgaaa
6181 gaagcagata taaatgaaat gaaaaatgat gcaaatatgt acaaagacaa gttagccagt
6241 ttggataaaa acggggagac ttttaacgat ggaagcgatg aaatttctac tgcacagaag
6301 tacaaaactg acgttgaagg tattatagac aaactaaacg ttatagatga aaccattaat
6361 ggaattaatt ccacgctaga cgaattgcta gagttaggaa ataattgtca gctgcatcgg
6421 accttttttaa tcagcagttc tttgaataat aaaattgcaa aattcttagt agagattagg
6481 gaacaaaaag agaacacaaa aaaatgcttc caatatgtaa aaagaaatca ccaacatttg
6541 gctaatttcg tttctgagtt acacaaaact cagggtggca tatttgagaa tgttaacctg
6601 gtggataaca caccggatgc agataagtat taccatgaat ttatggaaat agagcaggag
6661 gcaacgaaaa tcgtgaagga tataaaaaag gaaatatacc acttaaacga tgatgtagac
6721 gaacctgttt tggagaaacg gataaaagac gtgatcaaca cgtataacaa attgaagacg
6781 aaaaaggtcc aaatggacca gagttataaa aatatgtata tcactaaatt gagggaagtc
6841 gagggaagcc acgatctttt caaccaggta gcacaattga ttagaggcga aacggataaa
6901 aaagggaaag ccctgtcaga gagagaaaat aacttacaca gtatttacaa ttttgtaaaa
6961 ttgcacgaaa ctgagctgca taatctttac gccaaataca caccggagta tatggaaaaa
7021 ataaataaaa tattcgacga catcaatgct aggatgattg cggttgacct taatgatgat
7081 cacagcagtg aatatagcga cgtgaaaagg catgaacacg aggccatgct tttaatggac
7141 gcaacaaata atttgtcgaa ggaagtagaa atgatgcaga acgaaagtgg cggcaaaaac
7201 gatggcataa atggaggaaa aagccaactc gtggaagatt acacaaatac gatgagcgaa
7261 ttcacggagc aggctaagac agttgcgaaa aagatacacg actcaaaagg ggactacgca
7321 aatatgtttg atcatataag ggagaatgaa gccatgctgg aaagaattga cctgaagaag
7381 aaggacatta aagaaatact tgcgcattta aacagaatga aggaatattt attaaaaaaa
7441 ttatctgaag aggagaaatt acatcatatg agagaaaaat tagaagaagt taacacgagt
7501 actgacgaaa ttgtaaaaaa attcaggaca tatgatcaaa tggtagatat atcccaaaat
7561 attgacatca aaaatgtgca gtcaaaaagg tacgactctg tcgatgagat agataaagaa
7621 atgagttaca taaaaactca caacaaagat ttaatagaca gcaaatttat agtggaaagg
7681 gcactagaaa atgacaaaag gaaaaagagc gaaatggctc aaatattttc tacaatttcg
7741 agagataact cgagtatgta cgaatatgca aaaagctttt ttgatagcgt ccttaaagaa
7801 attgaaaaac taactcaaat gattagaaat atggataaat tgataaacga aaatgaagcc
7861 gtaatggaaa aattgaaaga tcaacgaagg gagttacaaa atgtagagaa tgcttctacc
7921 gatttgggaa aattagaaga agtagacaaa atggcacaaa ccaaatcgga aacagagcta
7981 tcagaaagaa acgatagtag gaatgcaaag gatggtgcaa cttattctac acttatggat
8041 gataaagaaa cagatagcgt gaacggagaa gagacgaaac aagaaaatgt tgtggtgaag
8101 aagggactac cccctcaaac agacatttac acaagtggag ttttaaaaaa tgacagaaac
8161 gatcaaaaat cagaaaaaat tggtgagaaa aagtcaaaca aaccagtagg caccgaggag
8221 aatatacaac acagcagtta tttaaacaat gataatagta acaacgatat tgacgtaggc
8281 accttgtata cgctaggtgg atataacgct ccaaatgata attataacac taatgaatct
8341 ggggatgata taaatgaaga agcgaaaaaa aagagaaatg ctgttttatt cgtctatgta
8401 gggggactgt ttagtgctct tttcatttgt attggtgctg tcttctattt gttacataga
8461 aaaattggca tagagggggt cggaaaaagc gatcacgaaa aaaaaccaac cattgaggac
8521 accaaaattg aggtatttga agaaacatat ggtttaaaac gtaatgtaaa agatgaggtt
8581 attgatgtac cctttgtcga tatggaagat aatttgtaa
//
```

```
LOCUS       Pvrbp2a                 7464 bp    mRNA    linear   INV 09-NOV-2015
DEFINITION  Reticulocyte binding protein 2a cDNA, full length CDS.
ACCESSION   Pvrbp2a
VERSION
KEYWORDS    .
SOURCE      Plasmodium vivax (malaria parasite P. vivax)
  ORGANISM  Plasmodium vivax
            Eukaryota; Alveolata; Apicomplexa; Aconoidasida; Haemosporida;
            Plasmodiidae; Plasmodium; Plasmodium (Plasmodium).
COMMENT
FEATURES             Location/Qualifiers
     source          1..7464
                     /organism="Plasmodium vivax"
                     /mol_type="mRNA"
                     /db_xref="taxon:5855"
     misc_feature    1..7464
                     /note="isolate VKBT95"
BASE COUNT     3322 a   1180 c   1255 g   1704 t      3 others
ORIGIN
        1 atggaaaata aggtgctctg ggcagttttt tacaatctag tgctgttcct tctggcatca
       61 agcaaagaaa gcaatcgaat taaagcatat aaacttaaga aggaacctaa gctatggcca
      121 ctacaagata gccttaacga aagcgataaa ttcgagtata ccaatggagg gaaggaaaat
      181 cccccaaatt tcttcagctc aaatgtaaag gcgcataata aaaaagaggg caaaaagtat
      241 gaacaaaatt tgtctttacc tgacaacaca tctttcgtaa cggtaaaaaa ttataactac
      301 acaagaacac catcacacca tgcttacata agaagagaca atacacacac caacagtaca
      361 aataataatc aaattaggaa tgtacctaaa gaattaaatc ctcgggaatt tctcttcaca
      421 ccaaagaatc aaataagcgc atctctcatc caaactaacg gaccggtggc tccaatggat
      481 atactacgtt acctagattt ctcaaacagt tcaggacaaa ttatttctac agtgtatccg
      541 ttctatgttc agatgagtta ttttgctgaa attaaatatt acataacata tcattacgag
      601 gcgaaaaaaa attatgatga agcatataat caaagcgtaa atccacttat gtcaagtata
      661 caaaatcaaa taaattcttg tgtaccgaaa aaagcagcat tggaaaaaac aatattcgta
      721 ttagaatacc cagagaatca caatataaac ctaagcaatt acgaagcaaa gcataatgag
      781 tacaagcagc aattagatgc atacaaaaac tgtgtacagg caaatatgaa atcatacaca
      841 gataggatgt caaaatttaa tgaaaaaatc tattcaattt taaacagtgt gaaatgcaca
      901 gatgcatgtg aaactgatac gtacgaaatt atgcttgaaa tatatgtaga aagagtaaag
      961 gaagttaatc ataataatta tgtcaattat ttgagtactt tgaaagcatc tctccaattg
     1021 ggggttaccc tgatgctcaa agtaaaacaa gaaatagaca ataatgtcac cattagtgca
     1081 ataaattttt tacaagaaga aatgttagat atcatcacaa taggtgaagc tcacacaggt
     1141 aaaattatac atggtaagga aaacgtacta aaacttcaaa ataataatat tccaccacaa
     1201 gtccctttaa gtactcttaa aaaattatat tttgattccg ccaatttcta tgccacttat
     1261 aagtttagct taaaaagagc cgataccacg acagctgcgc taaaggaaaa aggaaaatta
     1321 cttgctaacc tttataataa attgataaca tatgtaagcg aaaaaataga taaaaatttg
     1381 gattctttat acttcatttc aaaaagtgaa tcaatgattt ccgaatttga agatacottt
     1441 aaagcgggaa cagaagcaag caaagaaaac tccagcataa cagcagatta cgcacgctat
     1501 tcaaatttgc aaattataga tatcaaatca aagtacgatt acaaaataac cgagttaaag
     1561 aaatacttaa atcaattaaa ggtactcatt tctcctatga aagatttata taatctaaat
     1621 tctctacaaa aagaccaaag ggacagtgta agaagtatac gctcatctga tagtagtcca
     1681 caagaaaagg ttagcgcact tttaaacttc ttatcaaaaa taaaggaaga gaatggcacc
     1741 gtgttggaga attttaaaaa tatagaaaga tattatgaac aaggcaagcc attaaaaggt
     1801 acactcgagg gattaataaa aatgatcaaa acctacgaac aagatatagt aaatttaatt
     1861 gataacgaaa agcacaacag atcgtacaaa gatgacataa aaaacaaaat aatatatata
     1921 accgaaaaaa cagatgctat aaaagacatt atccccttaa atgaggaagt agacaatatc
     1981 atcaaacaaa ttgagaaact attcaatgaa gacctattcc atgaaggatt accaaatatg
     2041 gaaaaactta aaaaagagaa agaaaaaata aaagctacta taaaatcgct caacactaat
     2101 ttctacgacg gagacttaaa agaattagtc gacgatatgt taaattttgt ctacaaacac
```

2161 agcgatttct acgatcaagc atacgctaaa ggagatgttg taaatctaca aaaggaagca
2221 gaacaagaat atgaaaaaat tacaaaacta aaatcgaata acattcctca aatgttaaaa
2281 gatttgaaat cagaatcgaa aaatctttca gagcttaaag acaccatgat ggatgaaatg
2341 ttgaacaatg ttcatcaaga catatcaaac gttttcgaac agataaggaa caaatataaa
2401 gacataacaa cctcgctcag tgcctacaaa gaggacaaac agaaattcga gaaatttaaa
2461 gtcgatatga tacagacgaa aaacgaactc ctggggagtc ttcaacaaaa tgataaaaaa
2521 ctctcagaag caaataatat gtatgaagaa ttcttggcgc acatagatac tactaccaat
2581 agagaaaagc aaatgtataa ccaagccaac gcgttaaagg aaaatttagc aaatgttcaa
2641 agtaaaatat catcgtacca gggaaaaata gaaaaattga aagcgcatac taggaaaaat
2701 tatgaaaaaa ttaagacatt actgcaaact tttggcaatg aaaatataaa cgcaaaactg
2761 gcagaatacg aaaaagaatt taatgaaaat aagnaagtag tgaataccat cataaaccaa
2821 attcaagaat caaacaaaaa tatagagaat attaaaatat taaataattc tatcgaccgt
2881 tctgaagtga acaaacagct gatagaagaa ttggccacca ataagcaaca attaaaggaa
2941 aaaatagata gtcacattaa gcaactaaac agttacaata tcattgcaaa agatgataag
3001 ctaatttttg agaaaacgtt aaatgaggag aaggcaaata ttaacacgca actaagcgac
3061 acgtcaatag ataatttaaa agcacaaata caagaaacgc tagattatta caaaacagca
3121 aaggcaaaca cgaatgataa agatggaacc catttagaaa aattagataa aaaaaaaatg
3181 gattggaaag acagcaaagt taaaatagat naattaagcc ccagctatca agtgctagat
3241 aaaaaaatta atgatttgat taaaaagcaa cccgacgaaa ttgtagctct cattgataaa
3301 cttataacag aaaagggcaa tgaaataagg gagaaaaccg acgaaaaaat aaagcacctt
3361 agcgaaataa aaacaaaatt gtcctctttc actgtctccg ataatgttaa gaatacccaa
3421 aatgttgcaa taaaaggggа aataagtgga ttcgaaaaaa aaatggaagc cctattaaga
3481 agaatagaca ataataacgc aaaattaact gaactgagag ggaaggcaga ccaacatgta
3541 accaccctac gtggggaaaa aaataaaaca gctgaattca atgcgaaaaa agatagcctg
3601 gaaaaaatac accaacaaat ggaaaatacg cataaagaat tggagagcat ggaaaatgaa
3661 aaaattccca taaacgacat taaccaaatg gaaatagaat atgcacgaat tttaattcat
3721 catatggttc aacaaattgg tgaaaaaaag gaaaggtcta aaatcatctt agaagagatt
3781 aaaacaacta tagcaagcat cgatcaagca aagaaaaata tacctcctga gcaacaagat
3841 atccttcgcg aattcgagta tagcggatat cgcgacaaag ctactgccag tagtaccgaa
3901 atagaacaaa ttgataaaag tgcaaaagca gaggagggta aagccaacag tagcaccaac
3961 ttaagcgaca ttgatgtgat taaaaaacag gtactgtcct acttcagaaa agccaccact
4021 gaaagtaaca ccatggaaaa tgcgttaaga gaaattaaaa atgtgaacac tttcttaaca
4081 tcaaacattt cagaagaaat tttagctaac atactaaaga gtgcagcgag gacccaagaa
4141 ttaaatcaac aagcggtagg cgaatttaac aaaacagatc gtctaattaa agaagtagaa
4201 gcgaagctat cccaggcaaa cgaacataaa tcagcaattt caggaagtgt agaatacaaa
4261 caaatcgaac agaaaataaa cctaattaaa caaattcaga aagaaattac ggcggggaaa
4321 gaagaaataa acaactgttt aagtaatacc aaagaatata aagaaaaatg cgagtctgaa
4381 gtgaattcag taaatagagg aaaagctaaa gtagattttc tgcaaaaaag ggaagcgcta
4441 gaaaaaaaaa tgagtcaaga aaatttgggt aaaattacag atagcattga tcaatgcaaa
4501 aaagatttag cagacattac tagtttagaa gtaaaggtta aagctaatta cgattcaatc
4561 attaagtatg aagaatccat taataccatt ttaaattatt cctccatttt agaatacaaa
4621 accaaattgg aaatacgcaa aaaagaaaaa acagacctca tgacttatat taacacagaa
4681 aattctgcca ttcaggagaa gctgctaaac ttgcaaaaaa aattgaacca attgaatgaa
4741 aacacggact atactaaagt gggaaacgat ctcaacaacg caaagtctac aaaagcaaat
4801 gtaattatcc agtataactt aggacgagtc aaacatcaac tggaaaatgt aagtgtcatc
4861 aaacaggagc tagaaaaagt cctaagtgca gctaccgatt tagagaggga catctcaaaa
4921 atagccgacg tcacagaatc taataacttg gagagcctaa atgggaagga agcagattac
4981 acgaaacgta taagaagttt taacaaattg aagcagctcg tgcaagagaa agcagcaaaa
5041 gtagaagaaa tttcctccga tacggataac atagaaaaag aactgacaga acataaaata
5101 atttttgaag tgggacttac cgaaaggtta atcgaaatag ttaaaaatag aaaatcatac
5161 gtagacacaa caaaggaatt gctaaattca tcattaaaca atttgcttc cctctttaat
5221 ggattggact taaaaggata caatccaaaa gcaaacctag aaatgtacac acaaaaactg
5281 aacacaatac ataacgaatt tatggcatct cacaaaatat ttgatgaaaa atcaaagaag
5341 gtcttagaca aagatgtgaa cttcacggaa gctaaaacac tcagggagga ggcgcaaaaa
5401 gaagatgtta ctcttaaaaa taaagaggaa gaagcaaaat cgtacttgag tgatatcaaa

```
5461 aaaaaagaat ctttcgaatt tatacttcac atgaaggaaa aattaaacca aattagtaaa
5521 atgtgtgaac agcaatatga acaagctgat aaaggctata gtgaagtcaa aacaagcatt
5581 gatcgcattg caaatttaaa tgatgaaaat agtatagcgg atgtactcaa agaagcaaac
5641 gataaaaatg agcaagtaca aaatctgaca cattatacat ataaaaatga agcacaaaat
5701 gtgctaaggc atatggcaaa gtcggcaaat tttataggca ttaacctagt aacaggaata
5761 caaccgacgg aattatcatc acaagcaagc gaatcgacaa cgccagagtt aaaatttgaa
5821 tcagaagggg aaatgaaatt ggaaattcta acattatcag gaaatactac aaaattggac
5881 tattataaaa atatgaaaga cgcctaccag agcgtccttt ccatatttaa atactctcat
5941 ggtatagacg aaaaacaaag agaaagccaa aaaataaccg aaagtgcaaa tggctcatat
6001 cttaataata aggaaataaa tgaattcaaa ggaagactga acaatgtgaa gagtaaacaa
6061 acagccattt caaacaaaat tgataacgct actacgctgc tgcacaattt gaacaaaatt
6121 aaaacggatg ataagaatta cgacacaatt ttagaaaaag acgcttctga agaattgaag
6181 aggcgacgtg attcatttaa tcaagaaata aaaaacaccg tcgatggctt aaaattgaag
6241 gaaatccaag aaaagtttaa tgagcaagta aaactcctac aaaatttaga aacaaaagtg
6301 agcacactaa atgtgcatga gggaaacgct accgaaactg tgaaaaaaga aaacactgcc
6361 gttgatgcaa ttcaagccgc aatggaaggc atagaaaaag atgtcgaata tatcaactac
6421 tcatatgatg aactattaaa gaaaggacaa aaaattgaaa atcagagata cacatccata
6481 agggaaaacc tcaccaataa aatagcgaat gactcctcag caatcaacaa aataaagaaa
6541 aaagctcaag agtatttagc atacattaag aataactaca actctattta taatgatact
6601 ggcactttaa atgaatactt cgacaccaaa cggttaagta accacgattt aacaaatgtt
6661 caggaagcga ccagattaca cattgaaatg tccgcagccg ttgaagcatc agaagagata
6721 attgcagata tgaaaaatga attcataacg aacacagaag cggatataag cgctttacaa
6781 aatagcgcag acagattaan gtcgctttat agtaatctaa aacgcaaaca aattagtata
6841 aaccaaattt acaaaaagat taacttaatt aagttgcaag aaattaaaac aagcgctaac
6901 aaatatatgg acatagccaa actgtttaac aatgtgctag aggcccaaca taaagaattg
6961 gcacaagata taagtaaaat attgcaagct aaggaaaact ccattaatat gcagcaactg
7021 ttagaatctc acattgataa acttcgagcc ttgataacca atattgataa agaactgata
7081 gaattaacaa atggtaaaat taaagcatca aatcgacgta ttagtcaaat ttccccaatg
7141 gggcaaggca aattgttcag caccccagaa ggacaagctt ataataattt acacaacaca
7201 ggatataacc attatggaag tggaaaccat tcaagaggaa gaaatgaaaa tggaaatgtc
7261 cgttttgcag cagggattgt tgtgggattt ggtgtttgtt ccttttttgc ttcggcattg
7321 tttaaaggca aaggggaaaa cgaaacatat ggaagagatc taaattcacg tgatgaagaa
7381 tttgaagcca aaaacaatgg aaatcttcaa gataaggaag aaattattga agtgtccttt
7441 cacgaaagtg aaaatgcgta ttaa
//
```

```
LOCUS       Pvrbp2b                 8421 bp    mRNA    linear   INV 09-NOV-2015
DEFINITION  Reticulocyte binding protein 2b cDNA, full length CDS.
ACCESSION   Pvrbp2b
VERSION
KEYWORDS    .
SOURCE      Plasmodium vivax (malaria parasite P. vivax)
  ORGANISM  Plasmodium vivax
            Eukaryota; Alveolata; Apicomplexa; Aconoidasida; Haemosporida;
            Plasmodiidae; Plasmodium; Plasmodium (Plasmodium).
COMMENT
FEATURES             Location/Qualifiers
     source          1..8421
                     /organism="Plasmodium vivax"
                     /mol_type="mRNA"
                     /db_xref="taxon:5855"
     misc_feature    1..8421
                     /note="isolate VTTY57"
BASE COUNT     3774 a   1288 c   1438 g   1921 t
ORIGIN
        1 atggaaataa acgcgctttg gacgattttg tgcaatcttc tatttgtcct tttagcgtca
       61 agtagagatg tcaacaggag taaaccgaac agatttaaaa aggaactcac gttatcaccc
      121 atatatcaaa atttaattcg aagcagtcaa gtacaacatg ataaaaacta tgaagttgat
      181 gatggcaaaa ttaacgatgg aggtgacgaa aaagcgcatt cccctgatag ctcattctcg
      241 ggggattcag aaaggtatag cgctaaagga aaaccatcgt actattctta cataaaaaga
      301 ggggacatat atacacacag ttcaaacaca aataaaagct taaatgatag taacaagttg
      361 aataggaata cgattccaaa cgaatttatt caaactagta ataagaaaaa acctgagaag
      421 aaaactacca aaagtgaacc agctccaaaa aacggtcaaa cgtctccttc accaaaagag
      481 ccttcccccg aatctacaca aattaccaat acaactgata acatagacta cttcgacatt
      541 tcagatgaga gcaattacta tttaatttcc cagttaagac cacatttttc taatatatac
      601 tttttcgatg aatttaaacg ttacgcatca tatcacacag aaatcaaacg ttatgaggac
      661 atacataaga aaaaagttaa ttccctttttg aacgaagcat ccagagcaat cggcatttgt
      721 aacagggcaa aaaatacagt gaaggggtta ataaatattt tagagaatcc ccaaaagttt
      781 aaaacgcaaa gagaatcata tgatgtaaaa cttcgccaat atgaagaaaa aaaggaagca
      841 tttaggggat gcttactaaa taaaaatcgg aagaatttgg atcaaataaa aaaaataaat
      901 aacgaaattc gtgatctgct agaacaactg aaatgctcac agtactgtca aactaatgta
      961 tattttgaca tgattaaaat atacttggta gattttaaaa aaatgcctta cgaaaattat
     1021 gacactttta taaaacagta taaaaattct tacctttctg gtgttgatat gataaggaaa
     1081 attgaaaaac aaatagacaa cccagttacc ataaatgcga taaaattcac tcaaaaggag
     1141 atgggataca ttatagacag atttgaatat cacctgcaaa aagtgaaaca ttccattgat
     1201 caagtgacag cattgtccga cggggttaaa ccaaaacagg tcactaaaaa tcgtcttaaa
     1261 gagtactact ttaatatagg taactactac tccattttta aattcggcaa agatagtctc
     1321 aatatgttga ataaagcatt aatacataaa gaaaaaatag tccacaactt acttggtgaa
     1381 ttatttggcc atttagaaga gagaatatct aaacttatag attcagagta cttcataaca
     1441 gaaagtaata acatcatttc acaatccgaa gaaactttaa aacttgcgaa agacgtatat
     1501 gataaaaaca ccaaactgat agaggacctt acactgtatc cacacttaga aattaacgaa
     1561 ttcaaaaaag actacgacaa caatgtggaa gatttgagag aatctatcat ctacatacaa
     1621 tcatacgtct catctattaa aagtgcgtat agatataatg tgttagaaaa ggactctgta
     1681 gaaagtaaac gaaagaacat accagctaac agtaatgccc aggagaaagt cgatgagctg
     1741 ttgagcatta tagatagcat aagttatagc aatttttccg tagccgagaa ttttcaaaaa
     1801 atgaaagatt actataaaga aatcgaaaaa ttaaaaatca aaatactgca attaatagaa
     1861 gccattaaaa aataccaaca acatgtggaa gaattaatca acaaggaaaa agcagttgcg
     1921 attctcaaag aagatataaa taaaataata gaatatataa aaggaataat agaaaaattg
     1981 aaacaactaa tttctgcgaa taaagatttt gataaaattt tccagcaagt tgaacaatta
     2041 attaacgaag cgctttttaa caaagaccaa tttgaacata ataaaaatga cttacacacc
     2101 aaaatgaaag aaataatgca tacatttcac gagcgtgatt tacaacaatt tttggacaat
```

2161 atgtcgaaat ttttaaagga ccaagaagct tcctaccaaa atgcagacag taaggaaaag
2221 cttgaccaac tgctaacaac agtaaaggca aaacaagatg aacttaaaga gatgaaatgc
2281 gatgacattc cagacataat cgacaatttg aaaaaagaat cgcaaaatgt tttaaatcta
2341 aaagatgagg tcataaataa acagtttgaa aatatgcgca cagaaatgtc aagttcattg
2401 gatcaaatga ccaaagagta taacgctctc aaaagcagca tagaggaata cgaagcggaa
2461 aaaaaaggaa tagaaaacca taaacaaaat ataattaaaa gaaaaaatac attcatcgtt
2521 gctgaacatg aaaatgatga agatgtgccg gaagggaaaa atacctacaa cgagtttata
2581 agcaataagg acacaatttt gcaaaaagaa agcgcaatta gcaaccagat gaacacatta
2641 gaggaaaaaa aaagaaatag aaaaactacg ttacaaacat atggtgacgc aatacaaaaa
2701 ttagaaacgt acactgaaaa gaaggatgaa gaaactaaag tgctgctaga aaagtttaac
2761 accgaagtgg aaaacttcaa actagatgaa gatgaaaaat catttaacga tgccaaaagt
2821 attgtcagta acacaataaa tgaagtcgaa aatgaaaaca aaaatataga ctccattaag
2881 aaagtgaata ttgcaatgaa acgctccaga agtaaccaac aatccgttga acagctaatc
2941 agaaataagg ataatcttct aaaagaaaaa atggaaaaac actttcaaaa aataaacaca
3001 gatgaaataa taggacaaag tgccaaggca aacttgctaa acaatctaga gagcgaaaaa
3061 gctatcataa acgaacagtt aaatgacgct catttgaatg acctaaaatt gcagatagaa
3121 aaaactctaa gttattgtga aaaatcaagg gaaaatatta aaggaaataa tggaacacat
3181 ccagaagaat taaacgcaag cgaaattgag tgggaagcta cgaaaggaaa aatagaaaca
3241 ctaaacagta attatcaagc gttaaacaaa aacatggatg atataatcaa taagcagcac
3301 gatgaaatga tagtgctcat tgatagagat ataaaggaga agggaaaaga catcgagctt
3361 aaagttgagc aacgtataca ggcgctcgaa aaaatgaaag caaaattgtc ctcatttgat
3421 accaatgagg atgttaaaaa ataccaaagt gacataaata aagaagaact cataaaatta
3481 aaagaaatgt ccactgcagt cctaaacaaa attgatcaaa ataaaaatca attgattaac
3541 gtaaagacaa aatccagtgg ctacgtcgca gaatcgaatg gaataaaaaa taaacaaacc
3601 gaaccgagtc aagagaaaga caagcaaatt gtgtacaaca caaatagggg tgacatcgaa
3661 aaagtttata aaaacattag agacatgtac aacgaactgg atgatgtcga aaagggagaa
3721 accacctcaa acgaagtaga caacatagaa atgcagtatg acagactttt aatcaatttg
3781 cttgttcaaa agatcaacga agaaaacaca aaggcgaagg gactcatgga agaaattaaa
3841 tcatacatag aaaatattga aagtgtacag gccaaaatgt cagatgaaac gcgagaaggc
3901 gcaactgagt ttgagtttaa tcaatatcag cagagcagtt tagccaatga taaaaaaatt
3961 accgaaattg ttcaacatgc tataaataag aagggagaag cagaaagaag taaaaaaacg
4021 aatgaagtaa aatccatcaa aaatgacgtt aacacttaca tgcaacaagt tatagatgaa
4081 tataacgcta tgcaaaaggc gttaggggat attaaaaatg tgaacgattt attaatatca
4141 accagtggta gaagaatcgc tgaagaaata tcgaagagca ctaacgaagc tgagaatttt
4201 gaaaagcaag caaaaggaga attaggaaaa ataaatgata taattgaaaa aatggaaaat
4261 caaatggttc aggcaaagga acacgaaaat aaaattcata taaacttaga ggataaacaa
4321 atagatgaag aagtgcaaaa aattagacaa attcatgaag aacttataaa tacgaaaatt
4381 gcaatgaatg aacatttaag taagacaaaa gccgctaaag aaaattgcct ttcccaagtc
4441 cgtaatgccg agaggggggaa aattaaaata gattttctca ataataacaa acaaagtagc
4501 aatttgaaag acgtcaatat ggatcatgta actaaaaata ttgccgactg tgtggaacac
4561 tcaaaagaag ttaataaaac tgaaaaagaa gctgaaaagg aacatgacct attcctagaa
4621 tatgaaaaag atattaccag catattaaac agatcctcaa ttttaggaat agaaacaaaa
4681 tcgaaaaaaa gaaaaaacga agctaccaaa ataatgggcc aaattgaggg ggaacattct
4741 ataattaaaa cggaacttca acaatcttca gaaaatttta aacaactgaa tgaaaaatat
4801 aaccttagcg aagaagaaag tatgcttgcc aatcccacat ccaccaaagc aaatgagtcc
4861 atacagtata acataggcaa ggtgaaacaa aatttgttat acatagacaa tatagcgaaa
4921 gaagtagaca ctattttaac taacgcacaa gattcaatta gatcaatatc gcagatttcc
4981 aaactagacg gaaggaatac actacaaatg gtggaaaagg aagcagcaga ttatgtaaaa
5041 tatttagaca aaataaccaa agagaaggaa ctaatgaatg ataaacacaa agacctcaat
5101 cccataaagt ccgatattgc aaatatagaa acagaattga aaaagcatag gaaacttttc
5161 gaaataggac ttttgaataa ggttatcgag atagctaaaa cgagaaaatt gtacatggat
5221 tcagtggaac agctcctaaa ttcctccatc aataattttg ttaccctctt taatgggttc
5281 aatttaaaaa aatataaaat tgaaaccagt ttagacgttt ataaaggaaa aataagtgaa
5341 ttccacactg aattccttca gccatacgaa tcaattcaaa ataaggaaaa acgcgtactg
5401 gacccttcgg tagattttag tgcagcaaaa aatatgcgag aagaagctca aaaggaggaa

```
5461 aaaaatatcg aaaatgtaga aaaaaagaca aaaaaatatc taagcgatat taacaaaatg
5521 gaatccttta cattcatacg taatatgaga caaactttgg atgaactaag caaaacatgc
5581 aaacaggaat acgcagaagt cgagggaggt cacaaagaca ttgaaaaaat catagaagat
5641 attaagagcc taaacgatga aagtggctcg tcagctaaat tacaacaagc agaggataaa
5701 aatagccaaa tgaaagccaa agtgaatcat tattcactta agaaagaggc acacactata
5761 ttagaacaaa ttgtcaaatc agccaatttt gtaggcatta caatagtgac agcgttacag
5821 ccatcagatt tagctgctaa agcgagtttg caaaaagcac cagaattgca gtttgaatcc
5881 caatccaaag taacactaga aagcggacaa ttcttagaaa aaaaaaacca attggatgtc
5941 cacgaaaatg tgcaagccgc ctacaatacc gttcttcaga tatacaaata ttcagatgat
6001 atagtaagga agcaggaaaa atgcgaacaa ttagtaaaag acggaaaaga tatatgcctt
6061 aaattcaaat caataaacga aattaaagtt atgattcaaa atagtaaggg aaaagaaagc
6121 accctttcag ccaaagtgag tcactccttt aacaagctaa gtgagctaaa caaaattaag
6181 tgcaatgatg aaagttacga tgccattttg gaaacaccca gtagggaaga actaaacaaa
6241 ctaaggagca catttaaaca agagaaagac accatcgcaa accaagcaaa attgagtggc
6301 tataaaacag atttcgaaac tcacattgga aaattaaatg acttagccaa aatagtggac
6361 aacttaaaag ccagcgaaac tcttccaaaa aatatagaag aaaaaaaaac gtccatcaac
6421 ctaatttcaa ccaaattgga aaccatcgaa aaggaaattg aaagtattaa ttcctccttt
6481 gatcagttgc tcgagaaagg aaagaagtgc gaaatgacga agtacaaatt ggtaagggat
6541 agcctaagca ctaaaataaa tgaccattct gcaatcatta aggacaacca aaaaaaagcc
6601 actgaatatt taacgtacat tcaaaataat cacatttcca tatttaaaga cattgatatg
6661 ataaatgaaa acctgggtga aaaatccgta agtaggtatg ccatagccaa aatcgaagaa
6721 gcaaatgatc tttccgcaca gctaaccgct gcagttagtg aatacgaagc gatagctaat
6781 tttattagaa aaaaatttac aaatataagt gaccacaccg aaatggacac tcttgaaaat
6841 gaggcaaaaa tgttgaaaga acattatgac aatctaataa ataaaaaaaa catcataaca
6901 gaattacata acaaaattaa tttaattaaa ctgcttgaaa taagagccac ctttgataaa
6961 tacgtcgata tagctgaact gcttggcgaa gtagtaaagg atcaaaaaaa aaagttacaa
7021 gaagcaaaaa ataaactgga cacgcttaaa gaccatatag cagttaaaga aaaggagtta
7081 atcaaccatg acagctcctt caccctagtg tccattaaag cgttcgacga aatatatgat
7141 gacattaaat ataatgtagg ccaattgcac acattagaag taactaactt tgatgaactt
7201 aaaaaaggta aaacgtatga agaaaatgtt acccatttat taaacagaag ggaaacctta
7261 cagaatgatc tccacaatta tgaagaaaaa gacaaactga aaaatacaaa tatagaaatg
7321 tcaaatgaag aaaacaacca aataagacaa accagtgaag taataaaaaa attagaaagc
7381 gaatttcaaa acttgctaaa aattattcaa caaagtaaca cgctatgcag taatgataat
7441 ataaaacagt tcatttctga tattctaaaa aaagtggaaa ctattagaga aaggtttgtc
7501 aagaatttcc ccgagagaga gaagtaccat caaatagaaa tcaactacaa cgaaattaaa
7561 ggcattgtta aagaagtaga tacaaatcca gaaataagca tatttactga aaaaataaat
7621 acatatatac ggcaaaaaat tagaagcgct caccatttgg aagatgccca aaaaataaaa
7681 gacataatag aagatgttac aagtaactat agaaaaataa aaagtaaact ctctcaagtt
7741 aataacgctt tagatagaat taaaataaag aaaagtgaga tggataccct atttgaatca
7801 ttgtccaaag aaaatgccaa taattataat tccgcaaaat attttttagt cgattcggat
7861 aaaataatta agcatttaga agaccaagtt agcaaaatgt caagcttaat aagttacgcc
7921 gaaagggaaa taaaagaatt agaagaaaaa gtgtatagca tattaaatcg acctgttatt
7981 gataactccg cttcggagca aatcaaactg ttaagtgata cccaagagaa cgatacagac
8041 gaagctcaaa aacgggaatt tgctgaagaa caaattctga atttacctaa acaacagata
8101 cattcagatg agggcaacaa taatgatatg agcactacag atagtgcaaa tgaagagaaa
8161 caaactggag aggaagaatc acaacatgag gatcagtcac aaccaagggg gatacatagt
8221 cgagttcgcc ttgcagaagg aattattttg ggattatctg tcttttcagg tattattttt
8281 atctcattca ggcataagga tgaggaaaag aaagaccctg aatccgtcgg ggaacaattt
8341 gaaggtaata acaattttaa tgaagacgat aaggaagaag tcatagacgt ttgcttcgac
8401 aatagcgatt attctgaata g
//
```

```
LOCUS       Pvrbp2c                 8457 bp    mRNA    linear   INV 09-NOV-2015
DEFINITION  Reticulocyte binding protein 2c cDNA, full length CDS.
ACCESSION   Pvrbp2c
VERSION
KEYWORDS    .
SOURCE      Plasmodium vivax (malaria parasite P. vivax)
  ORGANISM  Plasmodium vivax
            Eukaryota; Alveolata; Apicomplexa; Aconoidasida; Haemosporida;
            Plasmodiidae; Plasmodium; Plasmodium (Plasmodium).
COMMENT
FEATURES             Location/Qualifiers
     source          1..8457
                     /organism="Plasmodium vivax"
                     /mol_type="mRNA"
                     /db_xref="taxon:5855"
     misc_feature    1..4567
                /note="isolate VKBT95"
     misc_feature    4568..4721
                /note="isolate VTTY57"
     misc_feature    4722..8457
                /note="isolate VKBT95"
BASE COUNT     3927 a   1074 c   1376 g   2080 t
ORIGIN
        1 atggaaaaaa atgttctttg ggtaatcttt tacaatttcc tagttattct tttggcatca
       61 tgtaatgatt ctaatcggag caaatctaac agtctaaaaa gtgaaagtaa gtcgttaccg
      121 acatatgcta atttaatgag gaatggtcaa gacaaataca acaatgctaa aacggaagat
      181 aatataggta atcagatcaa caatgacaac aaccataacg gatataatga taacagaatt
      241 aatagtgaat atcccaaaac atcacatcta cagcatagtc cgtctttggt acatttaaat
      301 gatcataaat ttacgacaaa accatctcga cattcttacg tacaaagaaa cagcatatat
      361 aaacgtaaca caaataataa tatggaaaat acaaataacg aattgcatgt tgttccaaat
      421 ttttttattc aagaaaaaaa agcagccccc caaaagccat ctgtccaaaa tacaccaacg
      481 gccactcaaa ttgtatataa taatttggac tacctcaatg ccttcgatga cactaataat
      541 ataatttctg cgtttaaacc tcaccaccca attatatatt acttcaagga attggaacat
      601 tttgcaaata gttactatga cctgaggagt aaaattaagg attattttgc tcttccgatg
      661 aaacaggctt ccgatgtggt agaaaaaaat gtaaaagact gtttgcagaa cataaatgaa
      721 tcgagaatat taatgactca gttagaaaat ccccaaaact ataatgatat aagtgataaa
      781 tatgatgaaa aagtaaaaga atacaaaaag aaaattgagg atatgcaaat ttgtttaaaa
      841 gacagttata taaaaaattt taaagctatc atgtctgcaa atttaaaaat gaatctagct
      901 ttaaacggaa tatatatcca ctggtggtac ctaacatgtt cgacgaaaac ttacgatgat
      961 atagttaaag aatatgcaat agagattaac gattttgatg aaaaaaaatc gatttctttt
     1021 atggataata tgaagaaaat acataagtct gctattgata cattgaagaa aatgaaagca
     1081 gaattaaata caagtctgga ttctaaaaga acagaattta taataggaga aattggacat
     1141 atgatagaaa agtttaatct tcatttgact aaaattcgtt atgcatctgc ttttatcaaa
     1201 agtattcctt tacaaaaggt agaaagtgac atttatcgag ttgaactaaa aactcttttt
     1261 tatgtggcag ctaaacacta cgctgatttt aaatttagtt tggaacattt aaaaatgttt
     1321 gaaaatttat ccaaaagcaa agaaaaaatg ctttatagca cttttgaaaa attagagggt
     1381 gatttactaa ataaaataaa cacccttatg ggttctgaac agtctacctc tgatcttacc
     1441 tctattattg cggattctga aaaaatcata aaatctgcag aatcattaat taattcgagt
     1501 tctgaagaga tagcaaaata tgcacttgat tcaaatgaga aaattaatga gataaaaaaa
     1561 aattatgacc aaaagatact cagtgtaaaa gaatttataa ataaatcaaa tgggttaatt
     1621 acaactgtaa aaggtacatc taaactaagt gaatcagata agaaacagat agataaaaaa
     1681 atagagaaag taaaaaaaaa tacaaacact ctagagaggg gaaatgagtt tataaaaata
     1741 atgaatgaag taaaaacaaa aataagcaat tcttctaata gcgctacaaa ttcaaaggca
     1801 ttttctgata aattgaaaga attacaaact gaatttgaag gattaaataa aactatcgaa
     1861 gacgatttgc aaaaaataaa tgagataaaa ataaaagaaa atgaggatcg ttctatgaaa
```

1921 gatcaaatag aacaacatct gaaagatgcg tctgataaca gtgaaaaagt aaaaaggtta
1981 atttccaaaa ataatgaaat acaaaaatat attcagataa ttgaaaattt gattaaagat
2041 gcaccttctg gcaaagacaa atttacatct gagaaaacag atttgcaaaa caaggtaaag
2101 gaaataatca atgagtttca tacgggagat ttacaattat tattaaatag tttatcaaca
2161 ttttacgaga aacaccaaaa gttatacagc ggagcaagta ctatagaaaa cattaaagag
2221 ttgcacccaa aaataaaaga agaatatgaa aacttgaaa aaaagaatta tagtaatatt
2281 gatcaaacat tacataattt ggatgcagaa ctagataaac tgaaagctct agagaaaaat
2341 attgtagaag agcaaactaa taatatcaat aaagatatga cagattcgtt gactaagtta
2401 aataccgaag tcgacagttt gagaaatgcc ttggatggct ataaggcgga tgaaagtgag
2461 ctcaaaacct ataaaaaccg aatgaatgaa aggaaagaaa attttatcag tactttaaag
2521 gaacaagagg atgatattcc agatggaaaa aatatttatg aagagtgtaa taaacataaa
2581 ggtgttatgg ataacaaaga acataaaata actagtgata taaatcaatg caaagaaaat
2641 attgtaaatg ccgaaaaaaa tatagagaca ttcaatactc tagtgaaaac attagaagca
2701 cacacaggta aaacatatcc agacgctcat acttcattgc aaaagcttaa aagtaatcta
2761 gaaaatttaa atttgagtga actcgaaaat gaatttaaat ctctcttcga ttcagctagt
2821 acaaccaata aacagattga aaatataaga aaaaatatag acacaattaa atcattaaat
2881 tttacgaaaa atagttcgga cagcagcaaa ttgtcattgg aaaagataaa agaaaataaa
2941 gcagatttaa taaaaaaact agaacaacac actcaagaaa tagaaaaata cacattcata
3001 gaaaaagaag aaacgtcacc cctttaagc gatttacgag aagaaaaaaa ccgcgtacag
3061 cgtgacatgt ctgaagagtt gataaaccag ttaaacacaa aaattaatga tatactggaa
3121 tattacgata aatcaaagga tagtttcaat ggggatgatg aaaaaaaatt ggaacaatta
3181 gatgacttta aaagaaagtg ccaagatgtt caacaaggaa tagaaaagtt aacaactaac
3241 tataaagtat tagaaaatag gataaatgac ataattaaaa aacaacatga gaaagttata
3301 acaatgactg aaaatcatat aacagggaaa gacaaaaaga taaatgaaaa gattcaacaa
3361 aatgtaaatt cgctaaatga aatgaaagcg aaattgggtt tactcgaaat taacgaagat
3421 atcaaaaata gcaaagatgt tacaattaaa aataaaatac aggaatttga aaacaaagtt
3481 aaaactatac ttggaaatat aggtaatgct aataataaaa tagatgcaat taaaagagtg
3541 catgatgcga ataaagttga attcgataga gaaaaagtta aaaaaacaag tttcgatgaa
3601 aaaaaggaaa tcataggaaa agtttatcaa caaatggaaa aaactcttaa agaattagag
3661 gaaatggatg acgaaagtag cataacaaaa gatgtagacg tagctcaaat actatacaga
3721 agaattttta ttaatcatgc tgttaatttg atggatgatg aagttgaaaa gtccaaaact
3781 gtgatggcag aaatcgaatt atataaaaaa gaaattgacg aaactaaaca gaaaacgatt
3841 gaatataagc aaggtgatac atctaatttt gattatacaa aagaatacaa caatgctaca
3901 cagagtaaag ctaaaataga aaattttatt gaaaatgtta agacaaaaaa aggaacgtct
3961 gacacaagcc aaaatataaa agaattagaa agtattaaag aagaggtgca tgataattta
4021 caacaagtca aactagaaaa tgtttctatg gaggaaattc gaaaacaaat tcttaatatg
4081 aaaaatttac taatttcgaa caattctgaa accatagcaa aagaaatatc aaataatact
4141 caaaacgcat taggttttag ggataatgca aaaacagaac ttaataaaac aaatgaacta
4201 ttacaaagtg tggaagctaa gatagctgag gcagaggcac ataaaaaaaa aattggcata
4261 gctttagaag atggacaaat agacacggag gtaagcgaaa ttgaacaaat taagcgagaa
4321 attataaata aaaaaaatga aattgaatcc tatttaaatg aaataaaaga atataaagac
4381 aaatgcacaa ccgaaatcag taattcaaaa agaggaaaag ataaaattga gttcttggaa
4441 aaatttaagc ctaatgagga aagcaatcgg aataaggtta acattaatga aataaatgaa
4501 aatataagaa attctgaaca atacttaaaa gatatagaag acgcagaaaa acaagctagt
4561 acaaaagtag aactattcca taaacatgaa acaactatca gtaatatttt caaggaatct
4621 gaaattttag gagtggaaac taaatcccaa aaaaaaatta ataaagcaga agacataatg
4681 aaagaaattg agcgtcacaa ttctgaaatt caaacacagg tgaaaggttt ccaagaaaat
4741 ctaaataaac tgaacgagcc ccataattat gacaacgcag aagatgaact taataatgat
4801 aaatctacga atgcaaaggt acttatagaa actaacctag aaagtgtaaa acataattta
4861 tcagaaatta ctaatattaa acagggagga gaaaaaatat acagtaaagc taaagatatc
4921 atgcaaaaaa taaaagcaac ttcagaaaat actgcagaga aaactttaga gaaggtgaaa
4981 gacgaccaat ctaattatgt taattattta aatcaaataa ccacagaaag aaatcttatc
5041 gttacggaaa aaaatagact aaatggtata gattccacta ttacaaatat agaaggggca
5101 cttaaagaat ccaaaaagaa ttatgaaatt ggatttttgg aaaagttaga agaaataggt
5161 aaaaatagaa aattaaaggt tgacataacc aaaaaatcaa taaattcaac agtgggaaac

5221 ttttcttccc tcttcaacaa ttttgattta aatcaatatg actttaataa aaatataaat
5281 gattatgaaa ataaaatggg agaaatatat aacgaatttg aaggatcatt aaataaaatt
5341 agtgaaaatt taagaaatgc ttcggaaaac acttcagact ataactcagc aaaaacactg
5401 aggctagagg cacagaaaga aaaagttaat ctattaaata aagaagaaga ggcaaataaa
5461 tatttaagag atgttaaaaa agtggaatca ttcagattta tatttaatat gaaagaaagc
5521 ttagataaga ttaatgagat gattaaaaaa gaacaactaa cagtcaatga aggacaaggt
5581 aacgttaaac aactagttga aaatattaaa gagttagttg atgaaaacaa cttatcagat
5641 atattaaaac aagcgacggg caaaaatgag gaaatacaga aaataacgca ctctacgctt
5701 aaaaataaag caaaaactat tttaggacac gtagatactt ctgcaaaata tgtaggcatt
5761 aaaataacac ctgagttggc actaacagaa ttgttaggag atgcaaaatt gaaaactgca
5821 caggaattaa aatttgagtc aaaaaataat gtagtactag aaacagaaaa tatgtcaaag
5881 aatacaaacg aattggatgt tcataaaaat atacaggatg cttacaaggt tgcactggaa
5941 atacttgccc actcagacga aatagataca aaacaaaaag acagttctaa attaatagaa
6001 atgggaaacg aaatatatct taaagttgtg ctaataaatc aatacaaaaa taaaataagc
6061 tctataaaaa ttaaggaaga agctgtttca ggcaaaatag acaatgtttc caaaaaacat
6121 agtgagttaa gcaaaattac atgcagcgat aaaacttacg ataacatcat agcgttagag
6181 aaacaaactg aattacaaga tctacgcaat tctttcacgc aagaaaagac cgaaacgaat
6241 agcgattcga agttggaaac aattacaaaa gatttcgaaa gtttgaaaaa tgcattaaaa
6301 acactggaag gagaagttaa tgctctaaaa gcaagctcga acgatcatga atatgtaaaa
6361 ggtaaaagtg caccaataaa tgctgtgcaa accgaaatgg aaaagacaga aacaggcata
6421 gatagccttg atacggccct tgatgaatta ttacaaaaag gaaggaaatg cgaagtatct
6481 aggtacacat tgataaagga taccgttgtc aaagaaataa gtgatgacac cgaattaatc
6541 aacactatag agaagaatgt taaagcatac ttggcatatg ttaaaaaaaa ttatgaagac
6601 acagtgcagg atgttctcac attaaatgaa catttcaaag aaaaacaggt aagtaatcac
6661 gagccaacta attttgataa atcaaataag tcatccgaag agttaactaa agctgttact
6721 gactcaaaaa caataataag taaactaaaa ggtgtaatta tagaagttaa cgaaaacact
6781 gaaatgaaca ctatagaaag cagtgcaaaa gaaattgaag ctctctataa cgaattaaaa
6841 aataaaaaaa catcattaaa cgaaatttat caaacatcaa atgaagttaa attgcaagaa
6901 atgaaaacaa atgctgataa atacatcgat gtatctaaaa tatttaacac tgtattagac
6961 actcaaaagt caaaaatagt ggctaatcaa aatagtataa acagtgttaa aggccaatta
7021 aacggaaagc taaaggaatt aattcaggct gacagctcat ttacattaga gtccattaaa
7081 aagtttaacg aaatatatag tcatattaag actaatatag gtgaactaga acagttacaa
7141 caaactaata aaagtgaaca tgataatgtc gcaaagcaca aagaaaaaat tgtacattta
7201 ataaacaggg tagaaagttt gaaaggtgat gtgaaaaatc atgatgatga ccaatatatg
7261 aaaaaattaa atgctagtct attaaatgat aatattaaaa atacaacgaa ttctataaac
7321 atatcagatg aagaacttaa gaaactattg aaaaaagttg aagagaatga tcagctatgt
7381 aaaaataaca atacgcagaa cttcatttcg gacattatga aacgtgtaga agacttgaat
7441 agacgattta cagaaaattt accggaaaaa gaaaaacttc atcaaattga aaacaactat
7501 aatgaaatta gttcaatatt tagtgaaata aacttacaag acgtcgatga atttgttgca
7561 aaaattcaca aacaaattga tgctgaaaag gcaagcgtaa ataatgtaag agaggctgaa
7621 aaaataagga ccgcaatcca aaatgttaca agttatgata cagaaataat aagtagacta
7681 tctgaaatga ataatgtact agaaaggatt actacacgga aaacaaaaat ggatcaatta
7741 ttaaaatcat tgtccccaga taatacaagt ctaaatttaa atgcaagaac acacgtaaag
7801 aactcagaag acttaattaa taggctaaat agccatatag aaaaaataac aaaactaaat
7861 acacatgccc acgaagtaat gacatactta gaaaatgaac taagtaaatt attaaaacaa
7921 cttgaaattg aaagtgcaaa agttgagcct gaagctttgc caagtgatac ggaagtaaaa
7981 gaagaaaaag ttccaccaac ggtaacagaa aatggtccac aagagaattt gacaagcgtt
8041 ccccaagaga catcagaaga taacacacca cagatccaag aaaatgttgt acaagaggat
8101 tcagtaatcg ctcctcagga gcaagtagaa tatagtacac tagcggtacc agaaaatgat
8161 gaaacaacag aagaagaaag tgaacatgac gatgcgcatg acgatacgca tgacgaatca
8221 caaactggaa gggattcaac ggcaaaagaa gcaattggaa agactcgttt agcaggagct
8281 gttattattg ccatgtctgt tttatccgga tttattataa tagtatttaa agataaagat
8341 gaagaagaaa aggatcacaa cgaacacgga tataacgaag catttggaga acatgacgaa
8401 tataatatgc atgataaaga agaagttatc gaagtttgct ttaatgaaga agattaa
//

```
LOCUS       Pvrbp2p1                1869 bp    mRNA    linear   INV 09-NOV-2015
DEFINITION  Reticulocyte binding protein 2p1 cDNA, full length CDS.
ACCESSION   Pvrbp2p1
VERSION
KEYWORDS    .
SOURCE      Plasmodium vivax (malaria parasite P. vivax)
  ORGANISM  Plasmodium vivax
            Eukaryota; Alveolata; Apicomplexa; Aconoidasida; Haemosporida;
            Plasmodiidae; Plasmodium; Plasmodium (Plasmodium).
COMMENT
FEATURES             Location/Qualifiers
     source          1..1869
                     /organism="Plasmodium vivax"
                     /mol_type="mRNA"
                     /db_xref="taxon:5855"
     misc_feature    1..1869
                     /note="isolate VTTY57"
BASE COUNT      813 a    264 c    276 g    516 t
ORIGIN
        1 atggaaaaaa atatcctgtg ggtaatcttt tacaatttcc tgtttattct tttggcatca
       61 tgtaaggaca cacatcgaag caaacctagc agactaaaac atgaccataa tttattgcca
      121 aattacgtta atttaatgaa ggatgatcaa aacggacaaa acagtgaaaa cagaggagac
      181 aacataaaca atcacaataa aaaccataac gatcagaaca accataatgg taataatgat
      241 aacagcatta atagtgaata cctaaaaact tcacatctac agaatagttc ggccatggta
      301 cacctaaatg atcataaaat tacaacaaag ccagctcgct attcttacat acaaagaagc
      361 aaaatatatg cacttaaccc aaataataaa aaaatagaaa atattaataa cgaattgcat
      421 agtgttccaa attcatttgt tcagtattat gtatacacca atctaaatgc tacagtatat
      481 aagatgcgtt acattagtat cttcgatgag tataataata tagtttcttt gcatctgcct
      541 tatcatgcta gagtaaatta cttaatggaa ataaaaaatt attcagtggc ttatagagaa
      601 ttacagagtg atgtttatag ttattatgat ccacaaatag acaaagtttt caaggttgca
      661 gagaaaaatg caattgtctg tgaaaagtac ataaacaatt tatataatac agtaactcgt
      721 ttagaaaatc cagaacaatt taaaaataac agagacaatt attccaaaga aataaaagaa
      781 taccaaaaac aacttacgtc ccttcatgat tgcttatttg aaaatcatag accaaattac
      841 aaagacataa tatatgctga tacaacaatt ttcgattctt tacaaaatat atactgttat
      901 tcggaagata aatgtctatc aaaattgtat cgtgatatgc ttggtttatc tatgaaaaaa
      961 attaatgaat atgaacagaa aaaaaaaaat gaagatataa ataaaattat taatatacac
     1021 gatactgctg tagatgtaat gaggaaaata aaaagggaat taaatccaag cttaaattct
     1081 gatgtggcag atttcgtaat aggtgaaatt aaatatatta tagagagact taaggcacac
     1141 tcagaaaaaa ttaaaagtgc atccgatttg gtaaaatata taaatcaaga aactgtgccg
     1201 aacgaaatct caaaaaatga gattaaaagt aattatattg tgttagctat tcacaccggt
     1261 tcctttcttt ttagtactga acatgtaaaa atgttagagg aaatattcaa aagtaaagaa
     1321 caaatacttt acaaaatttg tagtaaattt ttgaatgact tgaaaaacag aataactact
     1381 ctgataaatt cggaatattc ttcatcaaac tgcactccta ttgtttctac ttgtgaagaa
     1441 gccaaaaaat ccttagaatc cttgcgtact tcgagcaccg gaaaactagg taagcatgat
     1501 ctaaattcaa agccggaaat tgcatcagtt aaacagagtt atgatgggaa aatgattaaa
     1561 ttagcagaag ctataaaaag agccgaagaa ataatcaatt ctgtaaagga catcgttcag
     1621 tttaacacaa ccgaaacaga agcaaagaaa aaggaaacag atagcatacc ccaaaatata
     1681 aatgcactag agaaggataa aaaactttta gaagtaatag atgcaataaa aaaacaaaaa
     1741 caaaaaatat cggaaaattc taacaaaata aaagaatttt ctggtgcagc tgatacttta
     1801 aaagctgaag tcgaagaatt aaagaaaggt attgatgaag atgttaataa aatattaaag
     1861 ccattctaa
//
```

```
LOCUS       Pvrbp2d                 8492 bp    mRNA    linear   INV 09-NOV-2015
DEFINITION  Reticulocyte binding protein 2d cDNA, pseudo gene.
ACCESSION   Pvrbp2d
VERSION
KEYWORDS    .
SOURCE      Plasmodium vivax (malaria parasite P. vivax)
  ORGANISM  Plasmodium vivax
            Eukaryota; Alveolata; Apicomplexa; Aconoidasida; Haemosporida;
            Plasmodiidae; Plasmodium; Plasmodium (Plasmodium).
COMMENT
FEATURES             Location/Qualifiers
     source          1..8492
                     /organism="Plasmodium vivax"
                     /mol_type="mRNA"
                     /db_xref="taxon:5855"
     misc_feature    1..638
                     /note="isolate VTTY67"
     misc_feature    639..1346
                /note="isolate VTTY59"
     misc_feature    1347..2419
                /note="isolate VKTS93"
     misc_feature    2420..3261
                /note="isolate VTTY55"
     misc_feature    3262..3940
                  /note="isolate VTTY49"
     misc_feature    3941..4392
                     /note="isolate VTTY67"
     misc_feature    4393..4839
                     /note="isolate VTTY49"
     misc_feature    4840..5827
                     /note="isolate VTTY67"
     misc_feature    5828..7073
                     /note="isolate VTTY49"
     misc_feature    7074..7487
                     /note="isolate VTTY55"
     misc_feature    7488..8492
                     /note="isolate VTTY67"
BASE COUNT     4032 a   1003 c   1338 g   2119 t
ORIGIN
        1 atggaaaaaa aagccctttg gatcatcttt tacaatttca tacttattct tttcgcatca
       61 tgtaaggatg tcaatcgaaa caaacccgga agactaaaat atcatcggaa gctattacca
      121 gcatatgtta acttaatgag ccctgataaa gacggatata agggtgccga aaaggatgat
      181 aaggcaaata atcataacga caacaataat ggcaacaata atgacaacaa taataatagt
      241 gacaataatg atgaaaacag taataatagt gaaaatctca gaacgtcaaa tttacaaaat
      301 agttcatcag tggggcattt aaacaaccac gaagatacaa cgaaaccatc ccattattct
      361 tacttgaaaa aaagcaacat atatgcaccg gatgcaaaga ataataaaat ggaagatgat
      421 aataaaaaat tgcatactgt ttcaaattcc ttcattcaaa atagtaaaca agcccaaaat
      481 ataattgcaa ctaataattt ggattatatt agtgccattg atgattataa caatataatt
      541 tcttcatttc aaccataaca tgctagtgta tattacttga atgaattgaa atactatgca
      601 acacattaca atgaattgaa ggttcttgtt caaaaagatg tcattcctca agtaacacac
      661 attaccaata ttgttgagaa aaacgttaag atctgtttgg agaaaacaca tgaattgaat
      721 acattaataa ctcaattaga aaatccacaa acatataata ttcaaagaag ccattatagt
      781 aatagagtaa aagattaccg taagaaaatt gaggatattc aaaattgttt aaaattaaat
      841 tataaaagaa atgttaaagc tacagtattt gccacaataa tatggttcca gacactaatt
      901 aaaatggaat gtagttggtg gaccaaatca tgttcaacga gaatctatta taatatgatt
```

961 aagatgtata cattaaaaat taaggagtat aaaatcaaaa aaccaactgc atatatggat
1021 aaaataaagg ctgtttataa gttagctaat gatacaatat ggagaataaa agtagaatta
1081 aactcaaatc ttgattcgga tacaaccgac tttattctag aagaatttaa atacattata
1141 gaaaagtata atggtcacat tgataaaatt aatttgggaa cttcttatat agatcatatt
1201 catgaacaag aaggaacttt gaataatact aaaatagaaa ttataacttt gtattctgtg
1261 atagctaatc gttacactgc ttttaaattt agtttagaac atataaatat gtttgaaaat
1321 acatccaaaa gtaaagaaca ggtactttat aactctttta gcaaatttga ggatagatta
1381 caaaagaaaa taaacgatct tattaattct gaattttcta tatcaaccgt taactctgtc
1441 attttggatt ccgaaaaaaa tatacaatta tcagaatcat tactcaactc gagttctaaa
1501 gaaatagcac aacatgaaat taaattaaat gcggaaattg aaaagaaaaa aaaagattat
1561 gatcaaaaaa ttataagagc aagagaaact ataaaaaaat caaaggaatt aattacatct
1621 gtaaaaaacg catttaagtt aagtttagaa gaaaaggaaa agatagaaaa gaaagtaaca
1681 gaagcaaaaa aattgccaaa cgctctagaa agagacaaag catatttaga cattatgagt
1741 gaaataagaa aaattaaaaa taagttatct gataatgcta gaaagacaac cgaatttact
1801 aaaaaatcaa atgctttaaa aaaggaagtt caagaattaa atacaagtgt taacaactat
1861 gtgcaagcaa taaaggaaca aaaagaaaaa gaaattcgga ataattcttt gaaagatgaa
1921 ataaaaaagt ttttggaata tatacctaat aacagagaaa aaataaaaga attaattacc
1981 aaaaagaatg aagtgcaaca atatattccc aaaattgaag aattaattaa agacgcacac
2041 tttggtgtag aagaatttac agccaaaaaa acagaattgc aaaatcaggt aaattcaata
2101 attgatgcat tttataaaga agacctacaa ttatttttag acaatttatc aaaatcttat
2161 gatgaaaatc aagttttaga gaaggaagct gatactacag aaaagattga agaacttcac
2221 aaaagaacca aaatggatta tgaaaaactt ctaaatatga aatgtgatga tatacccgaa
2281 ataataaaaa agttgaatac agaattaaat aatctcaaaa atttagaaaa aaatattgta
2341 gaagaacaga ctaaaaacat taataaatat gtttcagatt catttgataa cctaactgat
2401 gaaatgaacc gtttaagaaa tagcttgaat gaatataagc aggatgaagg taagctccaa
2461 acatataaag gtagtataaa tgaaagaaaa gacaaattcc tcaatacttc aagcgaaaaa
2521 gaggaagata ttccagaggg acaaaatatt tatgatgaat ataaaagtca taaagatctt
2581 atggttaaca aagaacttaa attatccagt gatattaatg aattcaagga aaatataaaa
2641 aaagtccaaa caaaaataga ggccttcagc agtgtaatgc aaaaattagg agcgaacgcc
2701 gatgaacaac atcaagaaac tataaattct ttgaagaact ttaatactaa aatcgaaggt
2761 ttaaaattaa gtgaacttga aggtgaattt aaaattaaca atgaatcagc tgccaaatta
2821 tataatcaaa ttgaaaatac aatgaaaaat atagacacaa ttaaatcctt aaattatact
2881 agaaacagcg ccaacaatag cagagaatta atggaaaaaa tagtaaaaga taaaaatgat
2941 ttaataaaaa aactagatca acagtctgaa gaaataaaac aatacacatt aatagaagaa
3001 gaagaaaagt caccccttct aagcgcttta aatgaagaac aaaatcatgt agaaagtgaa
3061 attcctaaag agtcgataga caaattaaac acagaaataa atgctatact ggaatattac
3121 aacagttcaa agggtaattt taatgaggac acggaaataa aattggaaaa gttagacaat
3181 tttaaaactc aatgtgataa tattaagcaa gaaatagaaa cattaaacac taaatataaa
3241 gtgttagaaa aaaggataga caccctaatt gatgaacaac atgctaaaat tgtgacgttg
3301 acagataagc acataacaac caaagataat atgattaatc aaaagattga acatagcaca
3361 aaatcgctag atgatatgaa aactaagttg aattctctca aatttaacga tgatatcaga
3421 aagaatgaaa atgtcgaaat tcaaggtaaa atacaggaat ttgaaaaaat ggttcaaact
3481 atagaggaaa gtataaataa aaataaaact aaaatatatg aaattaaagg agtatacgat
3541 gtgtacaagg gtgaattcaa tgaagaaaaa aataaaagca caaaaatcgt agaaaaaaaa
3601 aatagcatgg aaaaagttta taacaaaatg gaagatactc ttaaaaaatt agaagatata
3661 gataacgaaa aaaatatatt aaatgaaata gaaaaggctg aagtactata caggaaagtt
3721 ttcattcata atactgttca tatgatggat aatgaaacta aaaagtctaa aaatttgatg
3781 gaccaaatcg aattgtctaa aagagaaatt gaggaaacaa agaagcaaat gcttgaatac
3841 aaaaatgatg aggtatctaa ttatgattat atgaaatatt acaaccaggc tacagagagt
3901 aatgctaaaa taaaagaaac ttttaaaaat gctacagata agaaagaaaa ggctgaaaat
3961 atcgaaaaag taagtgaaat cgatgacatt aaaaaccaag ttaatcataa tttacatcag
4021 atccaatcag aaaatagttc tatagaggag atgttaaaac aaattaataa tatgaaagat
4081 ttattaaaaa cgaacagttc taaaggaata tcggatacag tattaagtag taccaaaaat
4141 gcagaagaat ttggcaaaca agcaataagt gaatttaata aaacgaatga tgtaatacaa
4201 gcaatcagag atttgataac taaggctgaa gcacataaaa atttaatcaa tataaattta

4261 gaagatgaaa aaataaatgc cgaagtgaaa gaaattgaaa aaattaagga acaaattgcc
4321 aataagaaag aagaaataga atcccatctg agcaaggcaa aagaatttaa agaaaaatgt
4381 tcctcagaaa caagtaacgc caaaagaggt aaaagtaaaa tagaattttt gcaaagtcgt
4441 gaaggcagtt cggaagaggt agatatgaag gagatagaag aaaatattac caaagcagaa
4501 ggatatttaa atcaagccat tgctgcagaa acagaagcta ataagaatgt agaattgttc
4561 ggtgaacttc aaaaaaatat ctgtgacatt ttcgatgaat catcaatttt aggaatagag
4621 actacatcaa agaaaaaaat taataaagca acagaaataa tggaagaaat taaaaggaaa
4681 aattttgaaa ttcaaggcga ggtgaaaatt ttccatgaaa cactcgttaa attgaaagaa
4741 acacaccctg ataacaacgt agatgctgaa cttaacaatg cgaaatcgac caatgcaaat
4801 gtacttatac aaactaacct agaaatggtg gaacataact tatccgtaat agctaaaatt
4861 aaacaggagg gagaaaatat atataataga gcctcatcta cgataaattc catgacagaa
4921 atctccaaaa atactgaaaa aaaaacatta gacaaggcaa aaagtgacga atctaaatat
4981 atatcctatt taaccaaaat aacttcagaa agagatctaa tcattgaaga aaggaataaa
5041 ctaaatggta taagccccaa tataataagt atagaaaagg aattgaataa agccagaaag
5101 gattacgaaa ttggactttt gcaaaagata gatgaaatag gcaaaaacaa aaaaaaaaaa
5161 tattgatttg acaaaagaat caataaaatt aacattgagc aacttttctt cccatttcaa
5221 aggatttgat ttaaaggaat atgtctttaa taaaaatata aatgactatg aacaaaagat
5281 gaaagaaata catgacaaat atgacaaatc gataaataaa attagtaaaa acttgaaaaa
5341 ggctgaagag gataatacaa actataccct agcgaatgag ctgaggaaag aagcgcaaca
5401 agaaaaagca gaacttacaa atatacaaga agaagtaaat aaatatttaa gggatataaa
5461 aaaatgggaa tcattcagat ttatacttga tatgaaagac aatttaagta atataaatac
5521 actgattaaa caagaagaat taaaggttaa tgatggatat gaatacatta aacaattagt
5581 tgacaatatt aaagagtcaa atgatgaaag cagtatatca gataacttac aaaaaggaaa
5641 agaaaaaaat gtagaaatac agaacaaaat gcaatctacg tataaaaaca aggcaaaagt
5701 tattttagga catataattg actccgcaaa atttatagat attaatataa ttacaaaatc
5761 accactaaac gaattgtctt cagaatcaca tttgatgaat gcaggagaat taaaatttca
5821 gcaagaaaat aaagtaacat tagaaacaga acatatgaca aacaataaga gcgaattaga
5881 tgtttataaa aatatacgcg atgcttacaa catcgttgtg gagataattg catattcaga
5941 taaaatagac acaaaacaaa gacaaagtac acaattattg gatgatggaa atgatatata
6001 tcttacgcta aagtcaataa acgagctaaa aaataaaata aactctgtaa aaagtaaaga
6061 aaatgctatt tcaggcaaaa tagataatat ttcaaacaaa cttaatgagt taaacaaaat
6121 tacatgtgat gataaaagtt acgataatat tttagacata ataaaacata cagaattaaa
6181 aactatacgt gattctttta aacaagaaaa aattaacaaa aagagcgatt ctaaattgga
6241 aaaaataaag gaagaatttg aaaattcaaa aaatacatta aaaaatatag aagaagaagt
6301 taatgctcta aaggctagtt tggtaaaaca tgataatata caaagcagaa acaagccaat
6361 tgatgatgtg ctaatcgaaa tagaaaaaac agaaaaaggt atagatagtt ttagtgcctc
6421 ccttgatgag atgttgaaga aaggaagaga atgcgaaata tttaggtata catccataaa
6481 agatggtgtt atcgcaaaaa taaatgatga cgcagaatta atcgataata tacacaataa
6541 tactaatgaa tatttgacat atgttcaaaa aaattatagt gaaacagcag aggatgttcg
6601 tacattaaat caacatttca tgacagaaac gataagcgat catgcgccaa ctaattacga
6661 aacatcaaat aaatcttatg aagagataac tgaagctgtt aataattcca aagcaataat
6721 agataatata caaaattcaa ttatacaagt taacgaaaaa acggaaatta gttctttaga
6781 aaatagcgca gaaaaaatag aaaaccttta taaagaatta caggataaaa aaaacaccat
6841 aaacgaaatt tataaaaagg ctaacatagt taaattgcaa gaaataaaat cagatgctga
6901 taaatatttg gatgtagcta acatatttaa taatgtattg gacggtcaaa aatcaagaat
6961 aataaataac ctaggtaaaa tagcccaagt taaagaaaca ataaacctaa aactgaagaa
7021 attggttgag actgacaata catttacgac agagtctatt aacaggtttg gagaaattta
7081 taatgacatt aagactagta tagatgaact ggaaattata gaacaaacaa attatagcga
7141 acaaaatgat gtaaaaaatc ataaagaaaa aattacatac ttaataagca gaagagaaac
7201 tttagaaagt gacctaaaag gacatgaaga ggacacaaat gtgaaaaaat taaatgcgaa
7261 tacattaagt gaagtaaata ttggcattgc aagtgcaaaa gaagctataa gcaactcaga
7321 ggaagtattt aagaaattat taagaaaaat agaagaaaat gaaagattat gtaataataa
7381 tgatgcaaag aattttattt cggatattat gcaaaatata gatgatctga ataaacgttt
7441 cacaaaaaat ataccagaaa gagaaaaact ttttgaaata gaaaaggatt atatataatg
7501 aaattaattc cattttcaat gaaataagaa cacttaacgt tgacgaattt gttgaaaaaa

7561 tgtataaaca aattgatagt gaaaaggaaa gcgtaaataa cataacagat actgataaga
7621 taagtgttgc aattcaaaat gttacaaatt acgatgcaga aataaaaggc ttattgtcta
7681 gaactattaa cgtattagaa agaataagta gaaggaaagc ggaaatggat actttattca
7741 atagtctatc atcgaataat aaaaacctaa atgaaaatgc aaaaagatac atgaatgatt
7801 cagctgaaat aattaatcaa ttaaatagcc atgttcaaaa aataacaaaa ctgaaaacat
7861 atgcaaatga ggtaattgaa caattacaaa aagaactaac aagattatta gatcaacgta
7921 aaattgaaat tccagttgat gatactcaaa cttctttaag taccgaggac gtaaaagaag
7981 aaactgctaa tcagaaggag ctagaaactg ataagcagaa ggagctagaa aaagccatag
8041 aaaaggaaca agaagaagct gtagaaggtg aactacaaaa aactatagaa gtggaaaaag
8101 aatctgctat aaaagaggaa caagaaactc ctgagcagga ggaacgagaa acatctataa
8161 atcaggaacc agaacatgtt acttatgaga cactaggata tgatacacca catgcccaag
8221 aaaacgagag cgatggtact caacacgacg attcagaaag agatgataaa tcaaaggcaa
8281 aagatgcaat gggaagaact cgtttggcag gagctattat tatcggttta tctttttttc
8341 ccggagttct tgtattagca tttagggaca cgcaaacaga agaagaggaa agccatgaac
8401 atgattatca tcaagcattt ggaggtactg atgattataa tatgcaagat aaagaagaag
8461 ttatcgaagt ctgttttaat gaggatgatt aa
//

```
LOCUS       Pvrbp2dtr               8080 bp    mRNA    linear   INV 09-NOV-2015
DEFINITION  Reticulocyte binding protein 2dtr cDNA, truncated pseudo gene.
ACCESSION   Pvrbp2dtr
VERSION
KEYWORDS    .
SOURCE      Plasmodium vivax (malaria parasite P. vivax)
  ORGANISM  Plasmodium vivax
            Eukaryota; Alveolata; Apicomplexa; Aconoidasida; Haemosporida;
            Plasmodiidae; Plasmodium; Plasmodium (Plasmodium).
COMMENT
FEATURES             Location/Qualifiers
     source          1..8080
                     /organism="Plasmodium vivax"
                     /mol_type="mRNA"
                     /db_xref="taxon:5855"
     misc_feature    1..8080
                     /note="isolate VTTY57"
BASE COUNT     3844 a    939 c   1273 g   2024 t
ORIGIN
        1 atggaaaaaa gagccttttg aatcatcttt tacaatttaa tatttatttt tttcggtaaa
       61 attaaatcat gcgcacatac acgtaaattt atttgaatat gttcgctcaa attattgtaa
      121 ccctaaaccc tgaaccctaa accatatcat gctagtgtat attacttgaa tgaattgaaa
      181 tactatgcaa cacattacaa tgaattgaag gttcttgttc aaaaagatgt cattcctcaa
      241 gtaacacaca ttaccaatat tgttgagaaa aacgttaaga tctgtttgga gaaaacacat
      301 gaattgaata cattaataac tcaattagaa aatccacaaa catataatat tcaaagaagc
      361 cattatagta atagagtaaa agattaccgt aagaaaattg aggatattca aaattgttta
      421 aaattaaatt ataaaagaaa tgttaaagct acagtatttg ccacaataat atggttccag
      481 acactaatta aaatggaatg tagttggtgg accaaatcat gttcaacgag aatctattat
      541 aatatgatta agatgtatac attaaaaatt aaggagtata aaatcaaaaa accaactgca
      601 tatatggata aaataaaggc tgtttataag ttagctaatg atacaatatg gagaataaaa
      661 gtagaattaa actcaaatct tgattcggat acaaccgact ttattctaga agaatttaaa
      721 tacattatag aaaagtataa tggtcacatt gataaaatta atttgggaac ttcttatata
      781 gatcatattc atgaacaaga aggaactttg aataatacta aaatagaaat tataactttg
      841 tattctgtga tagctaatcg ttacactgct tttaaattta gtttagaaca tataaatatg
      901 tttgaaaata catccaaaag taaagaacag gtactttata actcttttag caaatttgag
      961 gatagattac aaaagaaaat aaacgatctt attaattctg aattttctat atcaaccgtt
     1021 aactctgtca ttttggattc cgaaaaaaat atacaattat cagaatcatt actcaactcg
     1081 agttctaaag aaatagcaca acatgaaatt aaattaaatg cggaaattga aaagaaaaaa
     1141 aaagattatg atcaaaaaat tataagagca agagaaacta taaaaaaatc aaaggaatta
     1201 attacatctg taaaaaacgc atttaagtta agtttagaag aaaaggaaaa gatagaaaag
     1261 aaagtaacag aagcaaaaaa attgccaaac gctctagaaa gagacaaagc atatttagac
     1321 attatgagtg aaataagaaa aattaaaaat aagttatctg ataatgctag aaagacaacc
     1381 gaatttacta aaaaatcaaa tgctttaaaa aaggaagttc aagaattaaa tacaagtgtt
     1441 aacaactatg tgcaagcaat aaaggaacaa aaagaaaaag aaattcggaa taattctttg
     1501 aaagatgaaa taaaaaagtt tttggaatat atacctaata acagagaaaa aataaaagaa
     1561 ttaattacca aaaagaatga agtgcaacaa tatattccca aaattgaaga attaattaaa
     1621 gacgcacact ttggtgtaga agaatttaca gccaaaaaaa cagaattgca aaatcaggta
     1681 aattcaataa ttgatgcatt ttataaagaa gacctacaat tatttttaga caatttatca
     1741 aaatcttatg atgaaaatca agttttagag aaggaagctg atactacaga aaagattgaa
     1801 gaacttcaca aaagaaccaa aatggattat gaaaaacttc taaatatgaa atgtgatgat
     1861 atacccgaaa taataaaaaa gttgaataca gaattaaata atctcaaaaa tttagaaaaa
     1921 aatattgtag aagaacagac taaaaacatt aataaatatg tttcagattc atttgataac
     1981 ctaactgatg aaatgaaccg tttaagaaat agcttgaatg aatataagca ggatgaaggt
     2041 aagctccaaa catataaagg tagtataaat gaaagaaaag acaaattcct caatacttca
     2101 agcgaaaaag aggaagatat tccagaggga caaaatattt atgatgaata taaaagtcat
```

2161 aaagatctta tggttaacaa agaacttaaa ttatccagtg atattaatga attcaaggaa
2221 aatataaaaa aagtccaaac aaaaatagag gccttcagca gtgtaatgca aaaattagga
2281 gcgaacgccg atgaacaaca tcaagaaact ataaattctt tgaagaactt taatactaaa
2341 atcgaaggtt taaaattaag tgaacttgaa ggtgaattta aaattaacaa tgaatcagct
2401 gccaaattat ataatcaaat tgaaaataca atgaaaaata tagacacaat taaatcctta
2461 aattatacta gaaacagcgc caacaatagc agagaattaa tggaaaaaat agtaaaagat
2521 aaaaatgatt taataaaaaa actagatcaa cagtctgaag aaataaaaca atacacatta
2581 atagaagaag aagaaaagtc accccttcta agcgctttaa atgaagaaca aaatcatgta
2641 gaaagtgaaa ttcctaaaga gtcgatagac aaattaaaca cagaaataaa tgctatactg
2701 gaatattaca acagttcaaa gggtaatttt aatgaggaca cggaaataaa attggaaaag
2761 ttagacaatt ttaaaactca atgtgataat attaagcaat aaatagaaac attaaacact
2821 aaatataaag tgttagaaaa aaggatagac accctaattg atgaacaaca tgctaaaatt
2881 gtgacgttga cagataagca cataacaacc aaagataata tgattaatca aaagattgaa
2941 catagcacaa aatcgctaga tgatatgaaa actaagttga attctctcaa atttaacgat
3001 gatatcagaa agaatgaaaa tgtcgaaatt caaggtaaaa tacaggaatt tgaaaaaatg
3061 gttcaaacta tagaggaaag tataaataaa aataaaacta aaatatatga aattaaagga
3121 gtatacgatg tgtacaaggg tgaattcaat gaagaaaaaa ataaaagcac aaaaatcgta
3181 gaaaaaaaaa atagcatgga aaaagtttat aacaaaatgg aagatactct taaaaaatta
3241 gaagatatag ataacgaaaa aaatatatta aatgaaatag aaaaggctga agtactatac
3301 aggaaagttt tcattcataa tactgttcat atgatggata atgaaactaa aaagtctaaa
3361 aatttgatgg accaaatcga attgtctaaa aaagaaattg aggaaacaaa gaagcaaatg
3421 cttgaataca aaaatgatga ggtatctaat tatgattata tgaaatatta caaccaggct
3481 acagagagta atgctaaaat aaaagaaact tttaaaaatg ctacagatca gaaagaaaag
3541 gctgaaaata tcgaaaaagt aagtgaaatc gatgacatta aaaaccaagt taatcataat
3601 ttacatcaga tccaatcaga aaatagttct atagaggaga tgttaaaaca aattaataat
3661 atgaaagatt tattaaaaac gaacagttct aaaggaatat cggatacagt attaagtagt
3721 accaaaaatg cagaagaatt tggcaaacaa gcaataagtg aatttaataa aacgaatgat
3781 gtaatacaag caatcagaga tttgataact aaggctgaag cacataaaaa tttaatcaat
3841 ataaatttag aagatgaaaa aataaatgcc gaagtgaaag aaattgaaaa aattaaggaa
3901 caaattgcca ataagaaaga agaaatagaa tcccatctga gcaaggcaaa agaatttaaa
3961 gaaaaatgtt cctcagaaac aagtaacgcc aaaagaggta aaagtaaaat agaattttg
4021 caaagtcgtg aaggcagttc ggaagaggta gatatgaagg agatagaaga aaatattacc
4081 aaagcagaag gatatttaaa tcaagccatt gctgcagaaa cagaagctaa taagaatgta
4141 gaattgttcg gtgaacttca aaaaaatatc tgtgacattt tcgatgaatc atcaatttta
4201 ggaatagaga ctacatcaaa gaaaaaaatt aataaagcaa cagaaataat ggaagaaatt
4261 aaaaggaaaa attttgaaat tcaaggcgag gtgaaaattt tccatgaaac actcgttaaa
4321 ttgaaagaaa cacaccctga taacaacgta gatgctgaac ttaacaatgc gaaatcgacc
4381 aatgcaaatg tacttataca aactaaccta gaaatggtgg aacataactt atccgtaata
4441 gctaaaatta aacaggaggg agaaaatata tataatagag cctcatctac gataaattcc
4501 atgacagaaa tctccaaaaa tactgaaaaa aaaacattag acaaggcaaa aagtgacgaa
4561 tctaaatata tatcctattt aaccaaaata acttcagaaa gaaatctaat cattgaagaa
4621 aggaataaac taaatggtat aagccccaat ataataagta tagaaaagga attgaataaa
4681 gccagaaagg attacgaaat tggacttttg caaaagatag atgaaatagg caaaaacaga
4741 aaaaaaaata ttgatttgac aaaagaatca ataaaattaa cattgagcaa cttttcttcc
4801 catttcaaag gatttgattt aaaggaatat gtctttaata aaaatataaa tgactatgaa
4861 caaaagatga aagaaataca tgacaaatat gacaaatcga taaataaaat tagtaaaaac
4921 ttgaaaaagg ctgaagagga taatacaaac tataccctag cgaatgagct gaggaaagaa
4981 gcgcaacaag aaaaagcaga acttacaaat atacaagaag aagtaaataa atatttaagg
5041 gatataaaaa aatgggaatc attcagattt atacttgata tgaaagacaa tttaagtaat
5101 ataaatacac tgattaaaca agaagaatta aaggttaatg atggatatga atacattaaa
5161 caattagttg acaatattaa agagtcaaat gatgaaagca gtatatcaga taacttacaa
5221 aaaggaaaag aaaaaaatgt agaaatacag aacaaaatgc aatctacgta taaaaacaag
5281 gcaaaagtta ttttaggaca tataattgac tccgcaaaat ttatagatat taatataatt
5341 acaaaatcac cactaaacga attgtcttca gaatcacatt tgatgaatgc aggagaatta
5401 aaatttcagc aagaaaataa agtaacatta gaaacagaac atatgacaaa caataagagc

```
5461 gaattagatg tttataaaaa tatacgcgat gcttacaaca tcgttgtgga gataattgca
5521 tattcagata aaatagacac aaaacaaaga caaagtacac aattattgga tgatggaaat
5581 gatatatatc ttacgctaaa gtcaataaac gagctaaaaa ataaaataaa ctctgtaaaa
5641 agtaaagaaa atgctatttc aggcaaaata gataatattt caaacaaact taatgagtta
5701 aacaaaatta catgtgatga taaaagttac gataatattt tagacataat aaaacataca
5761 gaattaaaaa ctatacgtga ttcttttaaa caagaaaaaa ttaacaaaaa gagcgattct
5821 aaattggaaa aaataaagga agaatttgaa aattcaaaaa atacattaaa aaatatagaa
5881 gaagaagtta atgctctaaa ggctagtttg gtaaaacatg ataatataca aagcagaaac
5941 aagccaattg atgatgtgct aatcgaaata gaaaaaacag aaaaaggtat agatagtttt
6001 agtgcctccc ttgatgagat gttgaagaaa ggaagagaat gcgaaatatt taggtataca
6061 tccataaaag atggtgttat cgcaaaaata aatgatgacg cagaattaat cgataatata
6121 cacaataata ctaatgaata tttgacatat gttcaaaaaa attatagtga aacagcagag
6181 gatgttcgca cattaaatca acatttcatg acagaaacga taagcgatca tgcgccaact
6241 aattacgaaa catcaaataa atcttatgaa gagataactg aagctgttaa taattccaaa
6301 gcaataatag ataatataca aaattcaatt atacaagtta acgaaaaaac ggaaattagt
6361 tctttagaaa atagcgcaga aaaaatagaa aagctttata aagaattaca ggataaaaaa
6421 aacaccataa acgaaattta taaaaaggct aacatagtta aattgcaaga aataaaatca
6481 gatgctgata aatatttgga tgtagctaac atatttaata atgtattgga cggtcaaaaa
6541 tcaagaataa taaataacct aggtaaaata gcccaagtta aagaaacaat aaacctaaaa
6601 ctgaagaaat tggttgagac tgacaataca tttacgacag agtctattaa caggtttgga
6661 gaaatttata atgacattaa gactagtata gatgaactgg aaattataga acaaacaaat
6721 tatagcgaac aaaatgatgt aaaaaatcat aaagaaaaaa ttacatactt aataagcaga
6781 agagaaactt tagaaagtga cctaaaagga catgaagagg acacaaatgt gaaaaaatta
6841 aatgcgaata cattaagtga agtaaatatt ggcattgcaa gtgcaaaaga agctataagc
6901 aactcagagg aagtatttaa gaaattatta agaaaaatag aagaaaatga aagattatgt
6961 aataataatg atgcaaagaa ttttatttcg gatattatgc aaaatataga tgatctgaat
7021 aaacgtttca caaaaaatat accagaaaga gaaaaacttt ttgaaataga aaaggattat
7081 atataatgaa attaattcca ttttcaatga aataagaaca cttaacgttg acgaatttgt
7141 tgaaaaaatg tataaacaaa ttgatagtga aaaggaaagc gtaaataaca taacagatac
7201 tgataagata agtgttgcaa ttcaaaatgt tacaaattac gatgcagaaa taaaaggctt
7261 attgtctaga actattaacg tattagaaag aataagtaga aggaaagcgg aaatggatac
7321 tttattcaat agtctatcat cgaataataa aaacctaaat gaaaatgcaa aaagatacat
7381 gaatgattca gctgaaataa ttaatcaatt aaatagccat gttcaaaaaa taacaaaact
7441 gaaaacatat gcaaatgagg taattgaaca attacaaaaa gaactaacaa gattattaga
7501 tcaacgtaaa attgaaattc cagttgatga tactcaaact tctttaagta ccgaggacgt
7561 aaaagaagaa actgctaatc agaaggagct agaaactgat aagcagaagg agctagaaaa
7621 agccatagaa aaggaacaag aagaagctgt agaaggtgaa ctacaaaaaa ctatagaagt
7681 ggaaaaagaa tctgctataa aagaggaaca agaaactcct gagcaggagg aacgagaaac
7741 atctataaat caggaaccag aacatgttac ttatgagaca ctaggatatg atacaccaca
7801 tgcccaagaa aacgagagcg atggtactca acacgacgat tcagaaagag atgataaatc
7861 aaaggcaaaa gatgcaatgg gaagaactcg tttggcagga gctattatta tcggtttatc
7921 tttttttccc ggagttcttg tattagcatt tagggacacg caaacagaag aagaggaaag
7981 ccatgaacat gattatcatc aagcatttgg aggtactgat gattataata tgcaagataa
8041 agaagaagtt atcgaagtct gttttaatga ggatgattaa
//
```

```
LOCUS       Pvrbp2e                 8200 bp    mRNA    linear   INV 09-NOV-2015
DEFINITION  Reticulocyte binding protein 2e cDNA, pseudo gene.
ACCESSION   Pvrbp2e
VERSION
KEYWORDS    .
SOURCE      Plasmodium vivax (malaria parasite P. vivax)
  ORGANISM  Plasmodium vivax
            Eukaryota; Alveolata; Apicomplexa; Aconoidasida; Haemosporida;
            Plasmodiidae; Plasmodium; Plasmodium (Plasmodium).
COMMENT
FEATURES             Location/Qualifiers
     source          1..8200
                     /organism="Plasmodium vivax"
                     /mol_type="mRNA"
                     /db_xref="taxon:5855"
     misc_feature    1..8200
                     /note="isolate VTTY57"
BASE COUNT     3724 a   1203 c   1380 g   1892 t      1 others
ORIGIN
        1 atggaaaaga acctattttt tgtgatcttt tgtaacctct tgtttattct ttccgcagta
       61 tgtaaagaca ataacaaaaa ccaacctaac aggctcaaaa gggatttcaa gctatcacca
      121 atacatgcca cttccttggg aagtgaccag ttcgaacatg gcagtgaagc aaaggggaac
      181 gaacaaaata atcgtaacag tgacaatgat tacagtaatg agcaccctaa tgatgataac
      241 aaaataagag gagaaaacgt aaacacgact cacttacgaa ataattcatc ctttatatcc
      301 ttaaaaaatt ataaaattgc aagaaaacca tctcgctatt catacctaga taaggacaac
      361 acatatgcag ttactaccaa aaataatgat aaaaaaaact tacagaaaga gattaatact
      421 attccaagta cattcataca aaaaactcat tatgaacccc agagagtgag tgattcatgc
      481 gattaatctg gattatgttt gtatctacgg tgaccataaa agtataattt ctttattgta
      541 tccatataat cctgaaattt atgcttttag agaactgaga agttatgcac tacttgacga
      601 agaaatgaag agagcaatcg acgaaacgta taatgtcgaa atacctcgca ttgaaaacat
      661 tgtgtctcaa agtttgaatg aatgctcaga tcaaataaga caattgaata cattaatata
      721 tcatctagaa aatccagaag gacatggaat tcaagaacgt gtatacggta ctaagaagca
      781 agagtacaaa aacagacttc aatctttaca tcaatgcata aaagaaaaat ataagaaaaa
      841 tgagaaagaa attttactaa gacaaatatc tacgaaacta taaaggatat atcgtgcaac
      901 tggatacact tgtgttcaac agatatatat tataatatga ttaagatacg tgagataaat
      961 gttaaagaat atgacgttag atcaattact tcatacttta atgcaactaa gaaaatacat
     1021 aggtctggtg aagatacaat aaagagagta aaaaaacagt taaataatag tcttgcctat
     1081 gaaacagtag aatttatatt agaagaaatt aaatatatta cagaaaaaat gagtcaacac
     1141 ttagaaaaag ttaagcaaga aactgaaact ataaataagc tttctaaaca agaagtctgg
     1201 tcccacttct ttcgatttaa tcttcaaagg gaattattct ttttagccta cagctattcc
     1261 agttttaaat ttagtactga caatttaaga gtcttgaaaa atgtgtccga gagcaaaaca
     1321 ggaaaacttt atcaatattt tgcaaaattc gagggcagtc tcaaccacat aataaatact
     1381 cttatgtcct ctaaatattc cacgtcaagt attaattctg ttctctcgga ttctgaggaa
     1441 attttaaaat ctgtagaaac attacgcagt tctacgtccg agcagataaa agaacatgta
     1501 ctttattcga atgcccaaat agaagaggct aagaaaattt tagatgaaaa aatgcttaaa
     1561 ttagaagaat atgcacaaga aacaaaaggg gtaattaaaa cagtaaatga aacatttaaa
     1621 ttaaatataa tagcaagtga agaaatagaa aagaaaagac gcgaaactaa caaaaacaaa
     1681 acttctctag aaaaatgtaa gatattcgta gaagttatag acttaataaa atcaaataaa
     1741 tataaggcaa cccaaaataa taaaaaaata gaggaacttt caaaacagat tatcactata
     1801 aaaagcaatt ttcaacaatc agagacagct attaacgaag atgtgcaaaa aattaaagaa
     1861 ttaattgaaa atgaaaaaaa acgtcgttcc atgaaagatg aaataaaaaa acatttgaaa
     1921 tattttcctg agacggtgga taacataaaa aaaatcattt cccaaaggga agaaatagaa
     1981 aaatatattc taagcattga aaatttgatt aaagacgcgc cctctgctgt ggacgaattt
     2041 acaactgaaa aaacggaatt gcaagacaaa gcaaaatcaa tcgttgattc attttataag
     2101 gaaaatttac aaacatttgt agacgattta gcagaatttt atacaaaaca tcaagcctta
```

2161 gacaaggaag aaaatactaa agaagaaact gaagcattgt acgaaaaaac gaaagaaaaa
2221 tatcaacagc ttgtagatat gaagtgcgat ggtataccag aaatgttaaa caatttgagt
2281 gaagtgttaa atagtctcac acagctaaag aacaaaatta tagaggggaa tattcaaaat
2341 atcaaaaaag atgtgtcaag ttcgttaaag aatctaactg aagaaacaga ccgattaaaa
2401 aatagcctga aggactacga gcaaagtgca gataaactga aaacctacat aaatagcata
2461 aacgggataa aagacaaagt tcgaactact ttaatcgaaa aagatgaaga cattccagaa
2521 ttaaaaaacta cctatgaaga atatacaaag caaaagggcg aaggtactaa taaggaaagc
2581 acagtaacac acgatattga cgtactcaaa gaaaatataa gaaaggctaa cgaacaaatt
2641 taaacttaca aaaacgtaat gcaaagatta gaaacacaga atagtgagac atatcaggaa
2701 actaaaactt tgttagaagg ttttaacact aaaatcgaaa gtttagaatt tgaaaaactt
2761 caagatgaat taaggagtca caaagaatcc gctagtagta ccataaacca aatagaaaat
2821 acaaaaaaag ttatagacac gctgaagtct ttaaatactc tcacaaagaa ctccaacaca
2881 aacgaacaat cgatcgcaaa aataaaagag agaaagaatg atttagtaaa aaaaatagat
2941 aaacatattg aagcattaaa tagttacaac atagtagaag ataaagaaag aacatccctt
3001 ttgaacactt taaatgaaga aaaaaataat gtagaaagtg aaatacctga aacatcaata
3061 tctaaattgc aagtagatgt aaaagcttca caagattact gtaaaaatga aaagatcaat
3121 atcgacaaag gcatggaaac caattcggaa gaactgggaa aatataaaac tgagtgccat
3181 aacacagaac tggaaataaa caaattaaac gccaattgtc aagcattaga aaaaaaaata
3241 gaagagttgg tacaagatca acatggccaa attataatat ttgttgataa acttataaca
3301 acgagggaca cgcagataaa tggaaaaatc gagctgaacc taaattctct aaaggatatg
3361 aaaacaaaat tagacgcttt taattttgaa gaagctgtta acaataacgg aaacgttact
3421 gttcaaagca aaataagtgc ttttaaagaa aaagtgaaaa gtacattgca aaatatagaa
3481 aatgaaatcg gcaaatttaa cggaattaaa cagaactacg atgcatatat ggaaacattc
3541 aacaaaggaa aagatcagat caccgaattc aaagataaaa aagagaacat ggaaaaaatt
3601 tataaacaaa tgaaagaaac cacactcaac aaattagaag aggcagaaaa agaaagtagt
3661 atattaaacc aagtaaaaca aatcgaattg gaatacagaa gaattttaat tcatcatgct
3721 gttcacatga tcgatcagga aaacacaaaa gctaaatcgg ttatggcgga tatcgaaaca
3781 tctaaaagca aaattgatga aataaaactt caaactgaag aatacaaaca gaagaatatg
3841 actaattttg aatatgaaaa tcattacagc aaggctaaac agagcaacac caaaataaat
3901 gaattttcta tagacgccac aagcgataag gaaaaggctg acaaaagcga agatgtaaac
3961 gaaatagaac gtatcaaaga aaaaattaat gacaatctgc aaaaagtcaa agaagaatat
4021 acttctatgg aggaaataaa aaaacaaatg aatagtatga aagatttact agctttgaac
4081 aattctgaaa cgactgctac aaacatatta gataacacaa aaaacgcatt aggttttagc
4141 gaggaggcgg caaaggaact tgagaaatca aaacaattat tagaagaagc ggtagctcag
4201 atagctaagg caggggaaca taaaggtaaa attgagatag ccttagaaga tgaacaaata
4261 aatgcccacg taaaccagat tgaactaata aagaatgaaa ttgtgaataa aaaaaaagaa
4321 atggagtcct atttaagcaa gataaaagaa tataaagaga aatgcaccac ccaagtcaac
4381 aattcgaata gaggaaaaga taaaattgaa ttcttgaaaa cggttaagca cagcgtagaa
4441 agtagttcga ataaggttga catggatcag ataaatgaaa gtatagaaaa atccgaacaa
4501 tatttaaagg atgtagaagc tctggagaca caagctgata acaatctaaa gtcatacata
4561 caacatgaac aaacgatcaa tgatactttc cagcaatctg aaattctagg aatagaaact
4621 aaatcaaaaa agaagttcaa cgaagcaaca gcactaatgg aagaaattaa aaaacaaaat
4681 tctgaaattc aaacaaaggt gaatgatttc caagtaaaac tgaacaaatt aaaggaaccc
4741 caaaatgccg aagacgtaga agaggagctt aataatgaaa agtcttccaa tgcaaaagca
4801 cttatacaca cgaacctaga aagagtgcaa tctaatttaa cccaagttgc caatattaag
4861 caggaagtgg atggcatatt taagagagtc acagacacga tgaattccgc aacagaaacc
4921 tctcaaaata aagatggcaa aacattaggc acggtgaaaa gggacgaacc taaatatgaa
4981 gagtacttaa cccaaataac tgcagataaa aatctaatgg acgcagaatt gaataagcta
5041 aacggaataa gttccactat cgaaatctta gaaaaggaat tgaatgaatc cagaatgaat
5101 tacgaaattg gactcctgca aaagatagat gaagtaggca aaagtaagaa gacaaatatt
5161 gatttaaaaa aagagttaat aagttcaacg ctgaattcct tttcttccct gttcaatggt
5221 ctcgatttaa atcaatacga atttaagaaa aacatagatg gttacgaaca gaagatgaaa
5281 gaaatacaag gcaaatttga cgtatcgctg aacaaaattg gtgaacattt aaaaaaggct
5341 tcagaggagg gcgcaaatta taccttagcg aataaactca gacgagaagc tcaacaggaa
5401 aaagccaaac ttacaagtag cgaagatgaa gcaagcaaat acatagaaga tattaaaaag

```
5461 gtagaatcca taagatttat aaatcacatg aaagaaaatt tagacaaaat aagcacatcg
5521 atcaaagaag aaaaattaaa aattaatgaa ggacatgaat tcatcaaaca gatagttgaa
5581 ggcattaaaa acacagatga tgaacgcgat gtatcaaaca aattacaaca ggcggaagga
5641 aaaaatgaag aaatgcagaa aataatgcac tctaagcata aaaacgaggc aaaagaaatg
5701 ctaggacata tagctacctc tgcaaatttt ataaatatta aaataattcc agaattaact
5761 ctaaccgagt caaacgtaaa cgaagcagta gaactaaaat ttcaaccgga tggcaaagta
5821 accttaaaaa caagaaacat ctcgaaaagc gaagccgaat tggatgtgca taaaaatata
5881 aaggaagctt accatctcgc tttggagata caaaaatatg caaacgaaat agacgcacaa
5941 caaacgaata acacacagtt aatggtcaca gtaaacgacc tacatcataa aattaagttc
6001 ataaatgaat taaaaaataa agtaaaaatt gaaaagagta atgaaaattc cctatcgggt
6061 aaagtaatgg gcatttctaa caaaattgcc gaattagaca aacattcatg cagtgaaaaa
6121 agttatgata aatttttaga aaacacgaag aaaacagatc tacaaaatat acgtgattcc
6181 tttaatcaag aaaagagaaa cgcaaatatt gacttgaatt tgaaaaatat aaaagaagat
6241 tttgccaatt cacaaacatc cttaaaaatt gttgaacagg aaattgaagc tttaaaggca
6301 aatgaaatca acactgaaaa tctgcaaggc aaatctactc agatagaaac actacataac
6361 aaaataggaa gcatagaaca aaatattgca agtctaaata cgtctttgga caaattacta
6421 aacagtggaa aaaaatgcga aatagctaga tacacatcct tgagggatag tattaaaggt
6481 aaaataaatg cagatgaaga aacaatcgat aatctgcaga agcatgttag tcaatatttg
6541 acatatgttg aagataacta taatgccaca gtgaaggacg tccttacatt aaatgaacat
6601 ttnagcaaca aaatggtgac tgatcatgct gcgactaatt ttgaaaaatc aaataaaact
6661 tcggaagaat tatccacagc ggtcaatcaa tcaaaggcca taataaacga tataaaaaat
6721 gccttcatag aagttaacga aaaaacggaa tttactttgt tagaaaacag cgcacaagca
6781 atagaaaagc tttacaatac attacatgaa aaaaaaaaac gcagtggacg aaatttacaa
6841 aacatccatt ttagttaaat cgcaaaaaat gaaatcaaat gctgataaat ataccgatat
6901 ggctaaaata tttgaaaacg tgttagacac ccaaaaatcg agaattacaa gtaaccagag
6961 tagcgtatcc cacgttaaag atatgataaa cgtacagctt aagaagttag aagctacgga
7021 cagcgcattt aaattagaat ccataaataa gtttcacgaa ttaagcgata acatcaaaaa
7081 gagtatagat gaattggaaa agctagaaca agttaatcgc caagaacata atactgtgga
7141 aacgcataaa gaacgaatta cacatttaat gaacagaaga gacagcctca aaaatggagt
7201 gaaagaatac ggagaagatg caaatttaaa aaaattaaga ggggatatcc taaaccaggt
7261 aattagtgac ataagaaata ttaatggaga acttaacaac tcagatcagc tgtacgtcaa
7321 attaatgaaa aaagtggacg aaaataatga tttgtgtaaa aagaactata cagaaaatta
7381 cgtttctgaa gtactgcaaa aaatcgataa tctgaataaa cgcttcaaac aaaatttacc
7441 agaaaagaaa aaagttttac aaattgagaa caactttaat gaaattaaaa ccctatttgg
7501 tgaaataaaa accttttata aggtagaaga atttgtaacg aacatgtata gtcaagttaa
7561 tggtgaaaag gaacgcgtaa aagatcagac aaatattgaa aaaataaaat tggcaataca
7621 aaatattaca aataacaatg aagaagtaaa aacctattta tccaaattta ttagcaccct
7681 agaaagaatg aatatcatga aaaaagaaat ggacgattta tccagctcac tgcccacaca
7741 taatacaaat gcaacagaaa atgcaaaacg gtttgtaaac agttcggtag aaatcattaa
7801 tgagctaact agctatattt ctaaaataac cgaactaaag aattatgctg aaggtgtgat
7861 aaccgaatta gaagaagcat ctaagttaat acgcccagct gctaatgact caaatgagac
7921 gcaaatgttg ccaaccgaag ctgatagcac acatcaagac gattcacaga gtgataatcg
7981 ttcaagggca agagatgcag atggaagaac tcgttatgca gcagctatta ttggtttgtc
8041 tcttattacc ggagttgccc tattaagccg caggcataat cctgatgaag aagaggatca
8101 tcatgaacac ggctatcatg aggcatttga gggcaacgac gactacactg tgcacgataa
8161 ggaagaagtc atcgaagtct gtttcaatga cagtgattag
//
```

```
LOCUS       Pvrbp3                  8423 bp    mRNA    linear   INV 09-NOV-2015
DEFINITION  Reticulocyte binding protein 3 cDNA, pseudo gene.
ACCESSION   Pvrbp3
VERSION
KEYWORDS    .
SOURCE      Plasmodium vivax (malaria parasite P. vivax)
  ORGANISM  Plasmodium vivax
            Eukaryota; Alveolata; Apicomplexa; Aconoidasida; Haemosporida;
            Plasmodiidae; Plasmodium; Plasmodium (Plasmodium).
COMMENT
FEATURES             Location/Qualifiers
     source          1..8423
                     /organism="Plasmodium vivax"
                     /mol_type="mRNA"
                     /db_xref="taxon:5855"
     misc_feature    1..403
                /note="isolate VTTY49"
     misc_feature    404..8423
                /note="isolate VTTY57"
BASE COUNT     3548 a   1101 c   1765 g   2008 t      1 others
ORIGIN
        1 atgaagaaaa acggatggtg gggagtatgc ttcggggttc ttctctttct tatcgctggg
       61 gaaatgccat aaacgaatcg aataaacaca aaaaggagga aacgacttct tctgttaatt
      121 cgtattgggg tagacaacat gagtcagaat accctggaag gaagaagaaa aattttaaaa
      181 aaaatagctt gtccccagga gaggagggat gtaggaatga ttgccccccc agcggtagtg
      241 atggaaacga atcagggtta tttaagaaga aagcacttat acatttaaga gatagcaaat
      301 ttagaaggaa gccatcgcgg tatgcgtata ttaaaagaaa cggtgagaac aatttggata
      361 gacttccgag tagcactgag aaggaggaaa ataatgaccg taatggaggt gccagttcct
      421 ttttacaaaa tttgaacgtt cccaatgtag acgagatttt agattatttc gagattgtag
      481 gtgaacaggg agagttagtc ttttcattag aatcatacta tgtggagatc tacacgtgca
      541 aagaaattgt gcgttttata aaccgccaag cgttaaccaa ctttagcggg aactatgggg
      601 atgatgatgg tgaggaggaa gaagaagatg aggatagtga ctacaattct gggttaagaa
      661 aagattccag gatacaaaat ctgtatcatc atatgaaact ctccatgatt tcttgcattt
      721 ctgaaaaggt gaagctacta aacgaaataa atagtttgga ataccccccag gcagaggata
      781 gtgaagagga ctcaagagca gagaattcta ccacatacaa attactcata aatgagtatg
      841 tagattgctt aaagaagtat agcgaaaatg ttaaagggaa gatagcggat gctaagaaaa
      901 gcatcgaaac agaatatgga aataaatttt gtcctaatga gtgcaattcg aatgtgtatt
      961 ttgaaagtgt acatttgtac ctctcatatg tcaataataa tgcagacatt ttgtacaatt
     1021 ctaacattag cgatgcgaag gaatttttaa aatctgggac aagcattgtt agcattatag
     1081 agaaagaaat agggttaaag catgtcataa gttctataaa gttcctgcaa agtgaaataa
     1141 ataacatcat aaataactac aattatcaca taaagaatat aaaaactgct atcgataata
     1201 taaaggagca tgaaaaaaat gataactcta tcgtgtttga taaaaaaagc ttggtggaga
     1261 aaagtttcga cttggcaaaa aacatgtccg tttacaaatt taacaatgag atgttcaata
     1321 gaatgaaagt attgtacgat ttgaagagaa ctaaatttaa aaaactattt attttcttag
     1381 cagaggagtt agagaataag cttaactcct ttgcagagtc cgcggaattt cagaaaacgt
     1441 atgactcgat aattaacgac tggaaattta tcttggcata cgcgaaagat gtatataata
     1501 aaaatttgac caagataaaa aattacgaag ggaatgaaag tttagaagtt atcatagtca
     1561 gaaataaggt gaaggaaaag ctagcaacgt tggaaggtct ggtagacaag ttagaggacc
     1621 tatttaatat catcaaatcg aagtatgtaa ttatcatgtc agcgaaatcg ctcattgggg
     1681 aactgaaggg ggaattcaaa actggagaaa atgtcgagca gaaatttgat gacctcataa
     1741 gattaatgga aacgataagc agcaaaatta acacgatgaa tgaaggtatc gatactgtaa
     1801 ataagaccta ctccagcatt aagtatgtac aaatacaaat cgaaaactta agcacttcta
     1861 tcgatgggta catgaacgaa atagatgcct taaaagctaa ggggtcaacg aatgatcata
     1921 ttagagaaga aatggtgtcc aaaatgttgt tcattacgga aaatatcaac aatttgaaaa
     1981 aaattttttc cctggaggag aaggcaaacg aaaatattgc gcaaattgat gaattaatta
```

2041 tgggaacatc gttagatatg aacgaaatta tagataaaaa aagtgacgcg tataataaaa
2101 taaaaatgat tattaaagat gtttatgaag gcaatttgaa ggactttcta gacgaactgt
2161 ctcttaatgt agacaagaat agaaattcca tcaaccaaag tagttctacg aacgaactgg
2221 atggcgtgtt aaataatata aaaggtgatt atgagaaaat gaaatctatc aaatgtgata
2281 atatatcaga atctttgcag aacataacga atgaactcaa aattatttca gacgccaaag
2341 aggaaatttt aaaaatccaa ttcggagata tcaatgaaaa attaacaaac tcgttagagc
2401 aacttcagag tgtctacgac aaattacaag ttagcacaac agagtataca gcgggaaagg
2461 ggaagattga ggaatacaaa acggttatac taaaggggga aaaagaattt tttgacaaag
2521 cgtatgataa ggatgaagat atgttagaag tgaaagatgc ttacgcaaat tttctcaaac
2581 ataaggataa tctcctcaag aacagaagta aagtgtttga agaattccat atggcgaaag
2641 aggccctaaa aacggctagg atttacctcc tatcttatgt agatgtgccg gaaaaatata
2701 acattatcaa gggggaaaca aatcaaaaat ataaaaagat aatacaagaa attaatgaca
2761 aagatgtcga tatcaaattg gaagaatacg aagcagactt caacagggac aaccaattat
2821 ccgatagcat cataaaggaa gtggaccaat caaataaaaa tttgtctaac ctgaaaattt
2881 taaacaagtc tattaaagat tgctccacgg atagcaacat tatcgaagaa ttaattagca
2941 aaaataattc tctgaaggaa aaaattattt ccgaaaggaa agaaatagaa gaagataatt
3001 tcatggaaca acatgtgaag ataaatttaa aaatcaaatt gaacgatgct cgtattactc
3061 ttgaaaacaa attgtatgac aacaatttga cgaacttgaa gacacaaatt aagaatattc
3121 tcgattttca cacaagttcg aaggaaaaat ataaagattt gaacgaaaca gacctgaaga
3181 aaataaaaga aaatgaagag tggaaaagcg ctaaggaatt gatatacgct cttaatgtcg
3241 aatacgaaat attgaagaag caagcggatg gtctgatcag cagcaaaaat agtgaaatta
3301 taaaatggat aggcaacaga atagtggaaa agaataaaga aataaatgaa aaagtggatg
3361 aacatgtaaa ttcgttcgat gaaattgttg taaaatctaa gtcgcccagt tttgtcagag
3421 atgttaatca ctaccaaaat ggtgagaata aagaaaaggc aagacagttt aatgcagaag
3481 tggaagctct cgttcggaag atcgaaacag aaaaggaaaa gtttaacaaa attaaaacaa
3541 attctaatga atacttggta caagcaaatc gaaagaaaag cgaaaatgta gatttcaagg
3601 ccaaggaaaa ggccatgaaa gaagtgtacg aacaaattag aagcgcatca gaagaactga
3661 ataactcact gaaggaaatt cccacactga atgatttaca gaagatagaa ttaaaatttg
3721 aaaaaaacga aattcacgat atggttaatc agattaccac tgagcagaaa aagtgtgagc
3781 aggaaatggc agaaatcaat tcttgtaaac atgatataga tgaaatgaag agggcaaact
3841 cgggagaaga gactcccgat tcccctttat ttgtatatac cgaattttac gaaaaagcgg
3901 taagcagtca tgggactatc aaagcccgtt ttaacgaggc taccgggcta aatgggaagt
3961 gtgagagtag cgaaaatatt aacgaaataa agggaatgaa aaagcagatt gatggttatt
4021 tgagagaaat tttaacaaat tatggcacct tgcatgaagc gttgggcgat attaaaaata
4081 tgaaggaaat gctgatggat gtggatgttc aagaaattgt gcgtctggtg gagaaggatg
4141 agacagaagc gcagaaatat gccacgttgg tgaaaagtga gcaggccaaa tcagatgccc
4201 taattaaaga attagataaa catttcacag aagcaacgag gctaaaaacg gaaatgaata
4261 aaaatttatc catccaaaga atcgatgaaa ttgtcaatga aattatgaaa tataaggacg
4321 aaatagcaca ccggaaagaa gaaatgaaca attatttgaa gaactccaag gagtataagg
4381 ataaggcttt tctgcattac cgtaacgcag acaggagaaa gaaaaagcta caacatttga
4441 aggaaaaggg ggaagaatct gaaccgcaga ttgatatgga taaagtaagt ttgagtgtaa
4501 accaatgtat gactcaatca aatgccgctt cagcgagtga agaagaaata aaaaagcacg
4561 gtaatagcta cgcagaatat gaaggaaaaa tgaatactct cttaaatgac atttccgttt
4621 tgcaaataag gaccaagtat gcaaagggga aggaccaggc caccagcatc atgagagaaa
4681 tcgaacaatt gcatgcaggc atccnagaaa aacttaaaga atctgaaaga aaattagaaa
4741 tgtggaagag gaaatcccaa atgaatgaaa gaacagatat attgaataat gacaaatcga
4801 agacggcata cataaacgtg aaattaaatt tagaattagt cgagtctaat ttaggccaga
4861 taaaaagttt gaaagaaagt gtgaataata ttctatccaa atcgactaat ttgaatagtt
4921 ccatactagg ggcgtataaa attgaaagtg atagctcctt agatgtgctg aaaaaggaag
4981 aaatgcatta catgcagtat ttgaaaaata ttgaaaatga gaaaaagctc atgacggaag
5041 agaagagcaa catggatgtg atacacggaa atgtccttaa aatagaaaat gagatggaga
5101 aatgtaaaaa aaattacgaa gagagcattt tgaaaaaagc caaagaaacg gctgataaga
5161 agaaacaact tattgaggca actagggagt cgctaaactc cctggagcca tactttaccg
5221 agatgtttaa tgaatcatat ttgaaagaat acaacattaa ggaaaaattt gtcggttatc
5281 aaaagagcat gaaagggtta tatgacgaat ttggtgagtc ctacaaagta atcgaaacga

```
5341 acgctgcaaa ggttgcagag ggcacggtaa agtatgatga agcgagaaga ctgagggagg
5401 aagctcaaaa ggcagaaata aatattaaca ataaggaaga agctgcaaaa acaaatttga
5461 acaaaataaa gcagcatgaa tttatgaact ttctatttca tacaaaggaa catgtggata
5521 atattaagaa agcgtgtgaa caagaaaata ccaaaatgga cgaaggacat acccatatta
5581 agcaaatcat tataaacatt cgaaagttaa cagaccagaa gagcactttc gaaacgttaa
5641 aaatggcaaa ggagaaaaat aacgaaataa aaaagaacgc acagcagtgt aataaaaatg
5701 aggcacatag cgcctttggg aagatgataa aagcgtctaa ttttatgggg ataaaaatat
5761 taacaaactt aggatccgag ttgagccccg aaatgcacct agaaactact tcacgaacta
5821 attcagagtt aaaatttgaa tcggaggtgg aaataaagtc ggaaagtgat gccaaattag
5881 atgcctacaa aaatctacaa gtggcctatg gatatattca aaaaatattt aaagattcgg
5941 aggagacaga gaggaaacaa gaagaaatcg aagggtggat aaggaaaggg aacaataaat
6001 gtcacgatat taaatcatct aatgaattaa aaactaaatt gaagtacacg aagtataagg
6061 gaaacattac tttaactaaa attaatgacg ctttgaccaa gtctgcccaa ttgaaagcga
6121 tgacttgcag tattgtgaac gatagcaaaa ttatggagag ctcagaatat acaaaattga
6181 aggagatgag tgatacgtac aatcggaaga agaaggaaga agtatccgag acggaggtcg
6241 acggaataaa tgaagaattc caagggatta tgaaaaatat agaaagttta gaaaagcaat
6301 ttgaagcagt acaggaacaa gaatcaccca gcgaaatgat tatcgtggaa aactccctag
6361 ttacagaaat taataaaaaa atagacagca taaatagcag aattacaacg attatgacta
6421 gtcttaacga gttgctagta acaggaaagg catgtgagaa atcgtcttat gcttccctaa
6481 ttggtaatat aaatgcgaaa acgtcaagtg acttatgaat aatcagtaac caaaaggaaa
6541 acgcggaaaa gtacgtagaa tacattaaga aaaattcaaa cttgataaat gatgacattc
6601 gtgcattaaa taaatacttt gatactaata ggataaataa ttaccagttg aaaaatttgg
6661 aagaagtaat taaacacgct aatgatttta acgtaaagga aaaagaagca acgggaatag
6721 ttaacgaaat taaaaaagaa ttcctagacc taagtcggga ggtagaaatg aatttcttaa
6781 atagcagcaa agaaaaaatt atggaacatt acaaaaatct gaaagataaa ataaagagca
6841 ttaacgaagt ttataaaaat atcaacttgg taaaattgaa agaaatggaa agtagctcag
6901 ataagtactt agaaatagca gggaaattta aaaatgtgct agatactcaa ataacaaggc
6961 tgttggataa tcatatggtg ctacaagata tcgaaaaaaa tataacagaa aatgaagggg
7021 aactgaaagg tattagtaat acatacacac tacagtccat ccaaaagttt aacaatgtgt
7081 gtaagaatat tgagaccaat atgcagaagc tgcatgaagt agagcagtag aataacagtg
7141 aggaaaaaca ggtgaaagcc tgtatagaaa atgtgtccca tcttataaac agaggaaaca
7201 atttgttgac cgatttaaat gattacgatg tagtaagcca tagtgcggcg aaagaaataa
7261 ctgatgagag tacaaaagag tacattacaa agattaaagg aaaggtaaat aataccatag
7321 aggcatttca aaaggtgtta gaaaacattc aggaaaataa atcgcacaca cagaacaatg
7381 gagatttaaa taaaggcata tacgaaatat ggaaaagggc taacgaaatt aaggcaaact
7441 tcacggaaaa ttttccagaa aatgataact actttcaagt gggggattat ctaaaggata
7501 taaaaaacat tttgaacgaa actgttggag gtgccaacat agaggaatat atcaaaagcg
7561 tttctcaaaa tattgaagaa caaataaagg gtacacaaaa tcatcgtaat gtggaatcca
7621 ttttaaaagc caaaaaaaag attcaatctt ataatgaaga ggcaaacaaa acattacaat
7681 ctatgaacac tgctcaggat aaaattttgt taaagaaaaa agacatggat aatatattta
7741 gtattctatc cgtaaatatg aagaatagtg tgtacataaa tacgaaaaaa tatattaacg
7801 aggtggatga tttatttaac aagttaaaag tggatgtaca taagttggaa aattttataa
7861 atgatactga actgagaata aagcagttag aggaagagga agctaagctc aaaattaaga
7921 atgcagttaa tggtgatttg aatgagcatt ctgaggatgc ttctcgtgat gaggacgacg
7981 aaccattagg tgaggttacg caaaattcag attcggaagg gtcaagtgat aaccaaaata
8041 ggcgtaaacg aaatggtgca agtagccatt caaaaggaaa ttcttctaat aactctttga
8101 ataatgtgca tggattaaaa gaagataata aaactggtga caaccattct gatgacaatc
8161 agaaagaaga atctcataat ggaagtaata attcgggtgg aaaaggctca cgggagaggt
8221 tcagatatgc tggaggaatt acgctagcat tttttatttg ctcaagtgct ggattcgcaa
8281 tcattaaata taaaaatcat gaatcacaag aagccgattt cggaacagac aaggagcact
8341 ttgaggggga taaagatttt aataaacggg aaaaggagga aattattgaa gtttctttta
8401 acgaaaatga acactatgaa tga
//
```

Alignment: Pvrbp1a

```
             ....|....| ....|....| ....|....| ....|....| ....|....| ....|....|
                5         15         25         35         45         55
1a_SalI      ATGAAAAGGG GGATTTGCCT AGCAGCGCTT CTTTGCCTGT TCAACTATTT AGGTGCAGGG
1a_Thai      ATGAAAAGGG GGATTTGCCT AGCAGCGCTT CTTTGCCTGT TCAACTATTT AGGTGCAGGG
Conservation ********** ********** ********** ********** ********** **********

             ....|....| ....|....| ....|....| ....|....| ....|....| ....|....|
               65         75         85         95        105        115
1a_SalI      CACGGAGAAA ATGCAGAAGA AGACATAAGG AATAGCGAAG GGAAAGTAAA CTTCTTTTCG
1a_Thai      CACGGAGAAA ATGCAGAAGA AGACATAAGG AATAGCGAAG GGAAAGTAAA CTTCTTTTCG
Conservation ********** ********** ********** ********** ********** **********

             ....|....| ....|....| ....|....| ....|....| ....|....| ....|....|
              125        135        145        155        165        175
1a_SalI      CTGGATTCTA ATTTAAAGAA AAATAAAAAG TCGAAACATA ATAGGGTGAA AAGAAGGAAC
1a_Thai      CTGGATTCTA ATTTAAAGAA AAATAAAAAG TCGAAACATA ATAGGGTGAA AAGAAGGAAC
Conservation ********** ********** ********** ********** ********** **********

             ....|....| ....|....| ....|....| ....|....| ....|....| ....|....|
              185        195        205        215        225        235
1a_SalI      GCGAAAATTT CGAACTTTCT AAGCCAAAAA GCGTATGTTA AAGAAACAGA TAACGCAAGT
1a_Thai      GCGAAAATTT CGAACTTTCT AAGCCAAAAA GCGTATGTTA AAGAAACAGA TAACGCAAGT
Conservation ********** ********** ********** ********** ********** **********

             ....|....| ....|....| ....|....| ....|....| ....|....| ....|....|
              245        255        265        275        285        295
1a_SalI      GGAAAAGACG CTGAAGGATC ACGTCCGTCT CATGATTCAT CGTTTGTTAA CTTAAACGGT
1a_Thai      GGAAAAGACG CTGAAGGATC ACGTCCGTCT CATGATTCAT CGTTTGTTAA CTTAAACGGT
Conservation ********** ********** ********** ********** ********** **********

             ....|....| ....|....| ....|....| ....|....| ....|....| ....|....|
              305        315        325        335        345        355
1a_SalI      CATGTCGATG GAAAAAGTTT ATCATACAGC GTTCATGTAA AAGAAAGTAC ACCACATAGT
1a_Thai      CATGTCGATG GAAAAAGTTT ATCATACAGC GTTCATGTAA AAGAAAGTAC ACCACATAGT
Conservation ********** ********** ********** ********** ********** **********

             ....|....| ....|....| ....|....| ....|....| ....|....| ....|....|
              365        375        385        395        405        415
1a_SalI      ACTACGCGGG AGAGCACGGA AAAGGGAAAA GAAAATGAGA AAATACAGGG TGTCCTAAGC
1a_Thai      ACTACGCGGG AGAGCACGGA AAAGGGAAAA GAAAATGAGA AAATACAGGG TGTCCTAAGC
Conservation ********** ********** ********** ********** ********** **********

             ....|....| ....|....| ....|....| ....|....| ....|....| ....|....|
              425        435        445        455        465        475
1a_SalI      TCTTTTGTGC AAAGCCAAGA AGGGGGAGAA TCAGACGATT TGGAGGGAAT GTACAATTCT
1a_Thai      TCTTTTGTGC AAAGCCAAGA AGGGGGAGAA TCAGACGATT TGGAGGGAAA GTACAATTCT
Conservation ********** ********** ********** ********** *********: **********

             ....|....| ....|....| ....|....| ....|....| ....|....| ....|....|
              485        495        505        515        525        535
1a_SalI      ATAGTTGATA TGAGCGAGAA ACTGAAAGAA AATGATAAGT ATAACCTAGT GTTTGACATG
1a_Thai      ATAGTTGATA TGAGCGAGAA ACTGAAAGAA AATGATAAGT ATAACCTAGT GTTTGACATG
Conservation ********** ********** ********** ********** ********** **********

             ....|....| ....|....| ....|....| ....|....| ....|....| ....|....|
              545        555        565        575        585        595
1a_SalI      GAGATTGATT TTGTTGACTT ACAATTTTTT AATCTAATTC TGGAGCTGAT TCCAAAGGAC
1a_Thai      GAGATTGATT TTGTTGACTT ACAATTTTTT AATCTAATTC TGGAGCTGAT TCCAAAGGAC
Conservation ********** ********** ********** ********** ********** **********

             ....|....| ....|....| ....|....| ....|....| ....|....| ....|....|
              605        615        625        635        645        655
```

```
1a_SalI      AGTCAATACC ATACCTACTA TGAGGAAACA CTTAAACAGC AAGTTACAGA ATACACAAAT
1a_Thai      AGTAAATACC ATACCTACTA TGAGGAAACA CTTAAACAGC AAGTTACAGA ATACACAAAT
Conservation ***.****** ********** ********** ********** ********** **********

             ....|....| ....|....| ....|....| ....|....| ....|....| ....|....|
                665        675        685        695        705        715
1a_SalI      TCGTTGAAAA CGTTAATGGA TTCATGCATA TCTGAAAAAG ATCAAATGAT CATATTAGAG
1a_Thai      TCGTTGAAAA CGTTAATGGA TTCATGCATA TCTGAAAAAG ATCAAATGAT CATATTAGAG
Conservation ********** ********** ********** ********** ********** **********

             ....|....| ....|....| ....|....| ....|....| ....|....| ....|....|
                725        735        745        755        765        775
1a_SalI      TATGAGATTA ATTATGCCAA GAGGAAGTCC ATAGAAACAG AGACGTTGGG TGATAAGGAA
1a_Thai      TATGAGATTA ATTATGCCAA GAGGAAGTCC ATAGAAACAG AGACGTTGGG TGATAAGGAA
Conservation ********** ********** ********** ********** ********** **********

             ....|....| ....|....| ....|....| ....|....| ....|....| ....|....|
                785        795        805        815        825        835
1a_SalI      ACAAAACTCT CAGCAGTATC TCAAGCCTAT GCAAAACATC TAGAATCGTA TAAGGGAGTA
1a_Thai      ACAAAACTCT CAGCAGTATC TCAAGCCTAT GCAATACATC TAGAATCGTA TAAGGGAGTA
Conservation ********** ********** ********** ****:***** ********** **********

             ....|....| ....|....| ....|....| ....|....| ....|....| ....|....|
                845        855        865        875        885        895
1a_SalI      TTAAAGCCCA AATTAAATGA TATAAAAAAT AATGCCTTTT CAGTTTTGAA GGATTCCTAC
1a_Thai      TTAAAGCCCA AATTAAATGA TATAAAAAAT AATGCCTTTT CAGTTTTGAA GGATTCCTAC
Conservation ********** ********** ********** ********** ********** **********

             ....|....| ....|....| ....|....| ....|....| ....|....| ....|....|
                905        915        925        935        945        955
1a_SalI      TGTAAGGATA ACTGTGGGGA ATATGTGCAG AAATATAATA CAATGCGCAA GAATTTTATA
1a_Thai      TGTAAGGATA ACTGTGGGGA ATATGTGCAG AAATATAATA CAATGCGCAA GAATTTTATA
Conservation ********** ********** ********** ********** ********** **********

             ....|....| ....|....| ....|....| ....|....| ....|....| ....|....|
                965        975        985        995       1005       1015
1a_SalI      TCAAGTTCAG ATCAGTATAA AATGGAAGCA TATATGTACA TTCCGAAGAG CATTAACGAT
1a_Thai      TCAAGTTCAG ATCAGTATAA AATGGAAGCA TATATGTACA TTCCGAAGAG CATTAACGAT
Conservation ********** ********** ********** ********** ********** **********

             ....|....| ....|....| ....|....| ....|....| ....|....| ....|....|
               1025       1035       1045       1055       1065       1075
1a_SalI      TATACAGTGC TAGATAAAAT TTTATCAGAA TCTAACGAAC TAGGTATAGA CATTCAAGAA
1a_Thai      TATACAGTGC TAGATAAAAT TTTATCAGAA TCTAACGAAC TAGGTATAGA CATTCAAGAA
Conservation ********** ********** ********** ********** ********** **********

             ....|....| ....|....| ....|....| ....|....| ....|....| ....|....|
               1085       1095       1105       1115       1125       1135
1a_SalI      ACTGTTAATT CGCTGAAGCT GTTAGGAGAA GAAATTAGTG AAGTTTCCCA TTTGTATGTC
1a_Thai      AATGTTAATT CGCTGAAGCT GTTAGGAGAA GAAATTAGTG AAGTTTCCCA TTTGTATGTC
Conservation *.******** ********** ********** ********** ********** **********

             ....|....| ....|....| ....|....| ....|....| ....|....| ....|....|
               1145       1155       1165       1175       1185       1195
1a_SalI      ATTAACAGTA CGTTAATTGA CGATGCAGCC AAAAAATTAG AATCCATAAA TGAAGAGGAC
1a_Thai      ATTAACAGTA CGTTAATTGA CGATGCAGCC AAAAAATTAG AATCCATAAA TGAAGAGGAC
Conservation ********** ********** ********** ********** ********** **********

             ....|....| ....|....| ....|....| ....|....| ....|....| ....|....|
               1205       1215       1225       1235       1245       1255
1a_SalI      GAATCTGCAG AAATAGATTT ACAAAAATTT GAAGATAATT CCAAAGCGCT AGCAAACAAC
1a_Thai      GAATCTGCAG AAATAGATTT ACAAAAATTT GAAGATAATT CCAAAGCGCT AGCAAACAAC
Conservation ********** ********** ********** ********** ********** **********
```

```
             ....|....| ....|....| ....|....| ....|....| ....|....| ....|....|
                  1265       1275       1285       1295       1305       1315
1a_SalI      TACTGCATTT TTCAATACAT AAAAACGCTT AACGAACCAA TTAAGAAAGC ATATGAAAGT
1a_Thai      TACTGCATTT TTCAATACAT AAAAACGCTT AACGAACCAA TTAAGAAAGC ATATGAAAGT
Conservation ********** ********** ********** ********** ********** **********

             ....|....| ....|....| ....|....| ....|....| ....|....| ....|....|
                  1325       1335       1345       1355       1365       1375
1a_SalI      AAGGTAATAA AATCTAATGA ACTACTGTCT ACCATTATAG ACACGTTAGG AAAAAGTGCT
1a_Thai      AAGGTAATAA AATCTAATGA ACTACTGTCT ACCATTATAG ACACGTTAGG AAAAAGTGCT
Conservation ********** ********** ********** ********** ********** **********

             ....|....| ....|....| ....|....| ....|....| ....|....| ....|....|
                  1385       1395       1405       1415       1425       1435
1a_SalI      ACTGCGCTGC AGGAGTCGAC ATTTAATCAA GAGGAGTGCA ATAAAATTAA AACCGAAGCG
1a_Thai      ACTGCGCTGC AGGAGTCGAC ATTTAATCAA GAGGAGTGCA ATAAAATTAA AACCGAAGCG
Conservation ********** ********** ********** ********** ********** **********

             ....|....| ....|....| ....|....| ....|....| ....|....| ....|....|
                  1445       1455       1465       1475       1485       1495
1a_SalI      GAAAAAGTAA AAGATGACGC TGAAGATATA TACGAAAAAA ATGAACAGAT ATACTACGAA
1a_Thai      GAAAAAGTAA AAGATGACGC TGAAGATATA TACGAAAAAA ATGAACAGAT ATACTACGAA
Conservation ********** ********** ********** ********** ********** **********

             ....|....| ....|....| ....|....| ....|....| ....|....| ....|....|
                  1505       1515       1525       1535       1545       1555
1a_SalI      ATTCCAGAAA GTGAGGACGA AACAATAGCT GATAAGATAA ACGATCTTCA GTACCTTATA
1a_Thai      ATTCCAGAAA GTGAGGACGA AACAATAGAT GATAAGATAA ACGATCTTCA GTACCTTATA
Conservation ********** ********** ********.* ********** ********** **********

             ....|....| ....|....| ....|....| ....|....| ....|....| ....|....|
                  1565       1575       1585       1595       1605       1615
1a_SalI      GACCAAATGA ATGAATATAA AGATGAAATA GTAAACAATT CGGAGTTCAT TTCTAATAGA
1a_Thai      GACCAAATGA AAGATTATAA AGATGAAATA GTAAACAATT CGGAGTTCAT TTCTAATAGA
Conservation ********** *:**:***** ********** ********** ********** **********

             ....|....| ....|....| ....|....| ....|....| ....|....| ....|....|
                  1625       1635       1645       1655       1665       1675
1a_SalI      TATAAAAATA TATACGAAAA CTTAAAAGAA ACGTATGAAA CGGAATTAAA TGATATAGGA
1a_Thai      TATAAAAATA TATACGAAAA CTTAAAAGAA ACGTATGAAA CGGAATTAAA TGATATAGGA
Conservation ********** ********** ********** ********** ********** **********

             ....|....| ....|....| ....|....| ....|....| ....|....| ....|....|
                  1685       1695       1705       1715       1725       1735
1a_SalI      CAACTAGAAA ACGACACATC AAAAGTAAAT TTTTATTTGA TGCAAATAAG AAAAATTAAC
1a_Thai      CAACTAGAAA ACGACACATC AAAAGTAAAT TTTTATTTGA TGCAAATAAG AAAAATTAAC
Conservation ********** ********** ********** ********** ********** **********

             ....|....| ....|....| ....|....| ....|....| ....|....| ....|....|
                  1745       1755       1765       1775       1785       1795
1a_SalI      ACTGAGAAAA CCAAAATAGA TGAAAGTTTA CAAACAGTGG AAAAATTTTA CAAAGGAATA
1a_Thai      ACTGAGAAAA CCAAAATAGA TGAAAGTTTA CAAACAGTGG AAAAATTTTA CAAAGAAATA
Conservation ********** ********** ********** ********** ********** *****.****

             ....|....| ....|....| ....|....| ....|....| ....|....| ....|....|
                  1805       1815       1825       1835       1845       1855
1a_SalI      TTGAATTTGA AAGAAAAAAT ATATGAACTG AAGATAGAGT TTGAAAAAAG CGTCACAGAA
1a_Thai      TTAGATTTGA AAGAAAAAAT ATATGAACTG AAGATAGAGT TTGAAAAAAG CGTCACAGAA
Conservation **..****** ********** ********** ********** ********** **********

             ....|....| ....|....| ....|....| ....|....| ....|....| ....|....|
                  1865       1875       1885       1895       1905       1915
```

```
1a_SalI      ATAAATCGAT TACAGGATGG GGAATCTGCG CGTGATTTAC ATGAAGAACA GATCAAAGAG
1a_Thai      ATAAATCGAT TACAGGATGG GGAATCTGCG CGTGATTTAC ATGAAGAACA GATCAAAGAG
Conservation ********** ********** ********** ********** ********** **********

             ....|....| ....|....| ....|....| ....|....| ....|....| ....|....|
                 1925       1935       1945       1955       1965       1975
1a_SalI      ATATTAGACA AGATGGCGAA AAAGGTGCAT TATTTAAAAG AACTTCTCTC CTTAAAAGGG
1a_Thai      ATATTAGACA AGATGGCGAA AAAGGTGCAT TATTTAAAAG AACTTCTCTC CTTAAAAGGG
Conservation ********** ********** ********** ********** ********** **********

             ....|....| ....|....| ....|....| ....|....| ....|....| ....|....|
                 1985       1995       2005       2015       2025       2035
1a_SalI      AAATCAAGTG TGTACTTCAC CGAAATGAAT GAATTGCTCA ACACAGCATC GTATGACAAT
1a_Thai      AAATCAAGTG TGTACTTCAC CGAAATGAAT GAATTGCTCA ACACAGCATC GTATGACAAT
Conservation ********** ********** ********** ********** ********** **********

             ....|....| ....|....| ....|....| ....|....| ....|....| ....|....|
                 2045       2055       2065       2075       2085       2095
1a_SalI      ATGGAGGGGT TTAGCGCTAA GAAGGAGAAG GCGGACAATG ACATTAATGC TCTATATAAT
1a_Thai      ATGGAGGGGT TTAGCGCTAA GAAGGAGAAG GCGGACAATG ACATTAATGC TCTATATAAT
Conservation ********** ********** ********** ********** ********** **********

             ....|....| ....|....| ....|....| ....|....| ....|....| ....|....|
                 2105       2115       2125       2135       2145       2155
1a_SalI      TCTGTGTATA GGGAAGATAT AAATGCTCTC ATCGAAGAAG TTGAAAAATT TGTAACCGAA
1a_Thai      TCTGTGTATA GGGAAGATAT AAATGCTCTC ATCGAAGAAG TTGAAAAATT TGTAACCGAA
Conservation ********** ********** ********** ********** ********** **********

             ....|....| ....|....| ....|....| ....|....| ....|....| ....|....|
                 2165       2175       2185       2195       2205       2215
1a_SalI      AATAAAGAAA GCACTCTGGA GATGCTAAAA GATGAAGAAA TGGAAGAGAA GCTACAGGAT
1a_Thai      AATAAAGAAA GCACTCTGGA GATGCTAAAA GATGAAGAAA TGGAAGAGAA GCTACAGGAT
Conservation ********** ********** ********** ********** ********** **********

             ....|....| ....|....| ....|....| ....|....| ....|....| ....|....|
                 2225       2235       2245       2255       2265       2275
1a_SalI      GCCAAAGAAA CTTTTGCTAA ATTAAATTTT GTTAGCGATG ACAAGCTGAC AGATGTGTAC
1a_Thai      GCCAAAGAAA CTTTTGCTAA ATTAAATTTT GTTAGCGATG ACAAGCTGAC AGATGTGTAC
Conservation ********** ********** ********** ********** ********** **********

             ....|....| ....|....| ....|....| ....|....| ....|....| ....|....|
                 2285       2295       2305       2315       2325       2335
1a_SalI      ACCAAAATGA GTGCAGAAGT GACTAATGCT GAAGGGATCA AAAAAGAAAT TGCGCAAAAA
1a_Thai      ACCAAAATGA GTGCAGAAGT GACTAATGCT GAAGGGATCA AAAAAGAAAT TGCGCAAAAA
Conservation ********** ********** ********** ********** ********** **********

             ....|....| ....|....| ....|....| ....|....| ....|....| ....|....|
                 2345       2355       2365       2375       2385       2395
1a_SalI      CAATTCGAAA ATGTTCATAA AAAAATGAAA GAATTTTCGG ATGCGTTTTC CACAAAATTT
1a_Thai      CAATTCGAAA ATGTTCATAA AAAAATGAAA GAATTTTCGG ATGCGTTTTC CACAAAATTT
Conservation ********** ********** ********** ********** ********** **********

             ....|....| ....|....| ....|....| ....|....| ....|....| ....|....|
                 2405       2415       2425       2435       2445       2455
1a_SalI      GAAGCGTTGC AAAACAGCAT GCAGCAGTAC AATCAGGAGG GAGACGCGAT AGAAAAGCAT
1a_Thai      GAAGCGTTGC AAAACAGCAT GCAGCAGTAC AATCAGGAGG GAGACGCGAT AGAAAAGCAT
Conservation ********** ********** ********** ********** ********** **********

             ....|....| ....|....| ....|....| ....|....| ....|....| ....|....|
                 2465       2475       2485       2495       2505       2515
1a_SalI      AAGCAAAATA GATCCGAAAA GGAGGAGGAA TATTTTAAAA ATGAAAGCGT AGAAGAAGAT
1a_Thai      AAGCAAAATA GATCCGAAAA GGAGGAGGAA TATTTTAAAA ATGAAAGCGT AGAAGAAGAT
Conservation ********** ********** ********** ********** ********** **********
```

```
             ....|....| ....|....| ....|....| ....|....| ....|....| ....|....|
                  2525       2535       2545       2555       2565       2575
1a_SalI      CTATCCAGGG AAGAAACAGA AGAGCAAGAA TACACTAAAC ATAAGAATAA TTTTTCCAGA
1a_Thai      CTATCCAGGG AAGAAACAGA AGAGCAAGAA TACACTAAAC ATAAGAATAA TTTTTCCAGA
Conservation ********** ********** ********** ********** ********** **********

             ....|....| ....|....| ....|....| ....|....| ....|....| ....|....|
                  2585       2595       2605       2615       2625       2635
1a_SalI      CGGAAAGGTG AAATATCAGC AGAAATTACC AACATGAGGG AAGTCATAAA TAAAATTGAG
1a_Thai      CGGAAAGGTG AAATATCAGC AGAAATTACC AACATGAGGG AAGTCATAAA TAAAATTGAG
Conservation ********** ********** ********** ********** ********** **********

             ....|....| ....|....| ....|....| ....|....| ....|....| ....|....|
                  2645       2655       2665       2675       2685       2695
1a_SalI      AGCCAATTAA ACTATTACGG GGTTATTGAA AAATATTTCT CCCTCATTGG CGATCAGAAT
1a_Thai      AGCCAATTAA ACTATTACGG GGTTATTGAA AAATATTTCT CCCTCATTGG CGATCAGAAT
Conservation ********** ********** ********** ********** ********** **********

             ....|....| ....|....| ....|....| ....|....| ....|....| ....|....|
                  2705       2715       2725       2735       2745       2755
1a_SalI      GAAGTGTCAA CGGCTAAAGC GTTAAAGGAA AAGATTGTTA GTGATAGTTT GAGGGACAAA
1a_Thai      GAAGTGTCAA CGGCTAAAGC GTTAAAGGAA AAGATTGTTA GTGATAGTTT GAGGGACAAA
Conservation ********** ********** ********** ********** ********** **********

             ....|....| ....|....| ....|....| ....|....| ....|....| ....|....|
                  2765       2775       2785       2795       2805       2815
1a_SalI      ATAGACCAGT ACGAGACAGA ATTCAAAGAG AAAACCAGTG CAGTGGAAAA TACCGTCTCA
1a_Thai      ATAGACCAGT ACGAGACAGA ATTCAAAGAG AAAACCAGTG CAGTGGAAAA TACCGTCTCA
Conservation ********** ********** ********** ********** ********** **********

             ....|....| ....|....| ....|....| ....|....| ....|....| ....|....|
                  2825       2835       2845       2855       2865       2875
1a_SalI      ACTATTCAAA GTTTAAGTAA GGCAATAGAT TCACTTAAAC GTTTAAATGG TAGCATAAAC
1a_Thai      ACTATTCAAA GTTTAAGTAA GGCAATAGAT TCACTTAAAC GTTTAAATGG TAGCATAAAC
Conservation ********** ********** ********** ********** ********** **********

             ....|....| ....|....| ....|....| ....|....| ....|....| ....|....|
                  2885       2895       2905       2915       2925       2935
1a_SalI      AACTGCAAAA AATATAACAC AGATATTGAT CTTTTGAGGA GCAAAATAAA AACTCTACGA
1a_Thai      AACTGCAAAA AATATAACAC AGATATTGAT CTTTTGAGGA GCAAAATAAA AACTCTACGA
Conservation ********** ********** ********** ********** ********** **********

             ....|....| ....|....| ....|....| ....|....| ....|....| ....|....|
                  2945       2955       2965       2975       2985       2995
1a_SalI      GAAGAGGTAC AAAAGGAAAT TGCCGAAACG GAGGGCGATA AAGTGGTGGG CGAAAATACC
1a_Thai      GAAGAGGTAC AAAAGGAAAT TGCCGAAACG GAGGGCGATA AAGTGGTGGG CGAAAATACC
Conservation ********** ********** ********** ********** ********** **********

             ....|....| ....|....| ....|....| ....|....| ....|....| ....|....|
                  3005       3015       3025       3035       3045       3055
1a_SalI      ACTGCGTTAC TTTTAAAAAG CTTAAGAGAC AAAATGGGAA AGATAAACGA GAAATTGAAC
1a_Thai      ACTGCGTTAC TTTTAAAAAG CTTAAGAGAC AAAATGGGAA AGATAAACGA GAAATTGAAC
Conservation ********** ********** ********** ********** ********** **********

             ....|....| ....|....| ....|....| ....|....| ....|....| ....|....|
                  3065       3075       3085       3095       3105       3115
1a_SalI      GATGGTAGAC TAAACAGTTT AGACACTAAA AAAGAAGATC TGCTGAAGTT TTACTCAGAA
1a_Thai      GATGGTAGAC TAAACAGTTT AGACACTAAA AAAGAAGATC TGCTGAAGTT TTACTCAGAA
Conservation ********** ********** ********** ********** ********** **********
```

```
             ....|....| ....|....| ....|....| ....|....| ....|....| ....|....|
                  3125       3135       3145       3155       3165       3175
1a_SalI      TCGAAGTCGA AAATTCATTT GAGCAAAGAC CAAAAGGGGC CACAAGATCC GTTGAATAGA
1a_Thai      TCGAAGTCGA AAATTCATTT GAGCAAAGAC CAAAAGGGGC CACAAGATCC GTTGAATAGA
Conservation ********** ********** ********** ********** ********** **********

             ....|....| ....|....| ....|....| ....|....| ....|....| ....|....|
                  3185       3195       3205       3215       3225       3235
1a_SalI      ATTGACGAAT GGGAAGACAT CAAAAGGGAA GTAGACGAAC TAAATGTTAA TTACCAGGTG
1a_Thai      ATTGACGAAT GGGAAGACAT CAAAAGGGAA GTAGACGAAC TAAATGTTAA TTACCAGGTG
Conservation ********** ********** ********** ********** ********** **********

             ....|....| ....|....| ....|....| ....|....| ....|....| ....|....|
                  3245       3255       3265       3275       3285       3295
1a_SalI      ATTAGCGAAA ATAAAGTTAC GCTATTTAAA AATAACTCTG TCACTTATAT AGAGGCAATG
1a_Thai      ATTAGCGAAA ATAAAGTTAC GCTATTTAAA AATAACTCTG TCACTTATAT AGAGGCAATG
Conservation ********** ********** ********** ********** ********** **********

             ....|....| ....|....| ....|....| ....|....| ....|....| ....|....|
                  3305       3315       3325       3335       3345       3355
1a_SalI      CATAGCCATA TAAATACTGT TGCTCATGGA ATAACGAGCA ATAAGAATGA AATTTTGAAG
1a_Thai      CATAGCCATA TAAATACTGT TGCTCATGGA ATAACGAGCA ATAAGAATGA AATTTTGAAG
Conservation ********** ********** ********** ********** ********** **********

             ....|....| ....|....| ....|....| ....|....| ....|....| ....|....|
                  3365       3375       3385       3395       3405       3415
1a_SalI      TCCGTAAAGG AAGTAGAAGA TAAACTAAAC CTCGTTGAAC AGAATGAAGA TTACAAAAAG
1a_Thai      TCCGTAAAGG AAGTAGAAGA TAAACTAAAC CTCGTTGAAC AGAATGAAGA TTACAAAAAG
Conservation ********** ********** ********** ********** ********** **********

             ....|....| ....|....| ....|....| ....|....| ....|....| ....|....|
                  3425       3435       3445       3455       3465       3475
1a_SalI      GTTAAAAATC CAGAAAATGA GAAGCAATTA GAAGCCATAA GGGGATCCAT GAGCAAGCTC
1a_Thai      GTTAAAAATC CAGAAAATGA GAAGCAATTA GAAGCCATAA GGGGATCCAT GAGCAAGCTC
Conservation ********** ********** ********** ********** ********** **********

             ....|....| ....|....| ....|....| ....|....| ....|....| ....|....|
                  3485       3495       3505       3515       3525       3535
1a_SalI      AAGGAAGTGA TCAACAAACA CGTCAGCGAA ATGACCCAAC TGGAGAGCAC AGCAAACACT
1a_Thai      AAGGAAGTGA TCAACAAACA CGTCAGCGAA ATGACCCAAC TGGAGAGCAC AGCAAACACT
Conservation ********** ********** ********** ********** ********** **********

             ....|....| ....|....| ....|....| ....|....| ....|....| ....|....|
                  3545       3555       3565       3575       3585       3595
1a_SalI      TTAAAGAGCA ATGCCAAGGG AAAGGAAAAC GAACACGATC TGGAAGAGCT AAATAAGACA
1a_Thai      TTAAAGAGCA ATGCCAAGGG AAAGGAAAAC GAACACGATC TGGAAGAGCT AAATAAGACA
Conservation ********** ********** ********** ********** ********** **********

             ....|....| ....|....| ....|....| ....|....| ....|....| ....|....|
                  3605       3615       3625       3635       3645       3655
1a_SalI      AAGGGACAAA TGAGGGATAT TTATGAAAAG TTGAAGAAGA TAGCGGAGGA ACTAAAGGAA
1a_Thai      AAGGGACAAA TGAGGGATAT TTATGAAAAG TTGAAGAAGA TAGCGGAGGA ACTAAAGGAA
Conservation ********** ********** ********** ********** ********** **********

             ....|....| ....|....| ....|....| ....|....| ....|....| ....|....|
                  3665       3675       3685       3695       3705       3715
1a_SalI      GGAACAGTAA ACGAATTGAA GGATGCAAAT GAAAAAGCAA ACAAAGTAGA ATTAGAGTTT
1a_Thai      GGAACAGTAA ACGAATTGAA GGATGCAAAT GAAAAAGCAA ACAAAGTAGA ATTAGAGTTT
Conservation ********** ********** ********** ********** ********** **********
```

```
             ....|....| ....|....| ....|....| ....|....| ....|....| ....|....|
                 3725       3735       3745       3755       3765       3775
1a_SalI      GAGAGGAACA TTATTGGGCA CGTTTTAGAA CGGATTACAG TAGAAAAGGA CAAAGCTGGA
1a_Thai      GAGAGGAACA TTATTGGGCA CGTTTTAGAA CGGATTACAG TAGAAAAGGA CAAAGCTGGA
Conservation ********** ********** ********** ********** ********** **********

             ....|....| ....|....| ....|....| ....|....| ....|....| ....|....|
                 3785       3795       3805       3815       3825       3835
1a_SalI      AAGGTTGTTG AAGAAATGAA TTCTCTCAAA ACTAAAATTG AAAAATTGAT ACAGGAAACA
1a_Thai      AAGGTTGTTG AAGAAATGAA TTCTCTCAAA ACTAAAATTG AAAAATTGAT ACAGGAAACA
Conservation ********** ********** ********** ********** ********** **********

             ....|....| ....|....| ....|....| ....|....| ....|....| ....|....|
                 3845       3855       3865       3875       3885       3895
1a_SalI      AGTGATGATT CACAAAATGA ATTAGTCACA ACGAGTATTA CAAAACATTT AGAGAATGCA
1a_Thai      AGTGATGATT CACAAAATGA ATTAGTCACA ACGAGTATTA CAAAACATTT AGAGAATGCA
Conservation ********** ********** ********** ********** ********** **********

             ....|....| ....|....| ....|....| ....|....| ....|....| ....|....|
                 3905       3915       3925       3935       3945       3955
1a_SalI      AAGGGGTATG AGGATGTAAT AAAACGAAAT GAAGAAGATT CAATTCAGTT GAGGGAGAAG
1a_Thai      AAGGGGTATG AGGATGTAAT AAAACGAAAT GAAGAAGATT CAATTCAGTT GAGGGAGAAG
Conservation ********** ********** ********** ********** ********** **********

             ....|....| ....|....| ....|....| ....|....| ....|....| ....|....|
                 3965       3975       3985       3995       4005       4015
1a_SalI      GCGAAAAGTC TGGAGACATT GGATGAAATG AAAAAACTAG TTCAGCAGGT TAACATGAAT
1a_Thai      GCGAAAAGTC TGGAGACATT GGATGAAATG AAAAAACTAG TTCAGCAGGT TAACATGAAT
Conservation ********** ********** ********** ********** ********** **********

             ....|....| ....|....| ....|....| ....|....| ....|....| ....|....|
                 4025       4035       4045       4055       4065       4075
1a_SalI      TTGCAAAGTG CTATACAAGG CAATGCTGGT ATAAGCAAAG AGCTGAATGA GCTTAAAGGC
1a_Thai      TTGCAAAGTG CTATACAAGG CAATGCTGGT ATAAGCAAAG AGCTGAATGA GCTTAAAGGC
Conservation ********** ********** ********** ********** ********** **********

             ....|....| ....|....| ....|....| ....|....| ....|....| ....|....|
                 4085       4095       4105       4115       4125       4135
1a_SalI      GTTATCGAAT TGTTGATATC AACGAATTAT AGCAGCATTT TAGAATATGT AAAGAAAAAT
1a_Thai      GTTATCGAAT TGTTGATATC AACGAATTAT AGCAGCATTT TAGAATATGT AAAGAAAAAT
Conservation ********** ********** ********** ********** ********** **********

             ....|....| ....|....| ....|....| ....|....| ....|....| ....|....|
                 4145       4155       4165       4175       4185       4195
1a_SalI      TCCAGCGAGT CTGTCCGTTT TAGTCAGCTA GCCAATGGGG AATTTACAAA GGCTGAAGGT
1a_Thai      TCCAGCGAGT CTGTCCGTTT TAGTCAGCTA GCCAATGGGG AATTTACAAA GGCTGAAGGT
Conservation ********** ********** ********** ********** ********** **********

             ....|....| ....|....| ....|....| ....|....| ....|....| ....|....|
                 4205       4215       4225       4235       4245       4255
1a_SalI      GAAGAGAAAA ACGCAAGTGC CAGATTTGCG GAGGCAGAGA AGTTAAAGGA ACAAATTGTC
1a_Thai      GAAGAGAAAA ACGCAAGTGC CAGATTTGCG GAGGCAGAGA AGTTAAAGGA ACAAATTGTC
Conservation ********** ********** ********** ********** ********** **********

             ....|....| ....|....| ....|....| ....|....| ....|....| ....|....|
                 4265       4275       4285       4295       4305       4315
1a_SalI      AAAGATTTAG ACTACAGTGA CATAGATGAT AAGGTAAAAA AAATTGAGGG AATCAAAAGA
1a_Thai      AAAGATTTAG ACTACAGTGA CATAGATGAT AAGGTAAAAA AAATTGAGGG AATCAAAAGA
Conservation ********** ********** ********** ********** ********** **********
```

```
             ....|....| ....|....| ....|....| ....|....| ....|....| ....|....|
                  4325       4335       4345       4355       4365       4375
1a_SalI      GAAATTTTAA AGATGAAAGA AAGTGCACTA ACATTTTGGG AAGAGTCAGA GAAGTTTAAA
1a_Thai      GAAATTTTAA AGATGAAAGA AAGTGCACTA ACATTTTGGG AAGAGTCAGA GAAGTTTAAA
Conservation ********** ********** ********** ********** ********** **********

             ....|....| ....|....| ....|....| ....|....| ....|....| ....|....|
                  4385       4395       4405       4415       4425       4435
1a_SalI      CAAATGTGCT CTTCACATAT GGAAAATGCT AAAGAGGGGA AGAAAAAAAT TGAGTATTTA
1a_Thai      CAAATGTGCT CTTCACATAT GGAAAATGCT AAAGAGGGGA AGAAAAAAAT TGAGTATTTA
Conservation ********** ********** ********** ********** ********** **********

             ....|....| ....|....| ....|....| ....|....| ....|....| ....|....|
                  4445       4455       4465       4475       4485       4495
1a_SalI      AAAAATAATG GGGATGGAGG AAAGGCCAAC ATAACGGATA GCCAAATGGA GGAGGTAGGT
1a_Thai      AAAAATAATG GGGATGGAGG AAAGGCCAAC ATAACGGATA GCCAAATGGA GGAGGTAGGT
Conservation ********** ********** ********** ********** ********** **********

             ....|....| ....|....| ....|....| ....|....| ....|....| ....|....|
                  4505       4515       4525       4535       4545       4555
1a_SalI      AACTATGTTA GCAAAGCTGA GCACGCCTTT CACACAGTAG AAGCACAGGT AGACAAAACT
1a_Thai      AACTATGTTA GCAAAGCTGA GCACGCCTTT CACACAGTAG AAGCACAGGT AGACAAAACT
Conservation ********** ********** ********** ********** ********** **********

             ....|....| ....|....| ....|....| ....|....| ....|....| ....|....|
                  4565       4575       4585       4595       4605       4615
1a_SalI      AAAGCCTTTT ACGAATCCAT CGTAGCTTAT GTAACGAAGA TGGACAACCT GTTTAACGAA
1a_Thai      AAAGCCTTTT ACGAATCCAT CGTAGCTTAT GTAACGAAGA TGGACAACCT GTTTAACGAA
Conservation ********** ********** ********** ********** ********** **********

             ....|....| ....|....| ....|....| ....|....| ....|....| ....|....|
                  4625       4635       4645       4655       4665       4675
1a_SalI      TCGTTAATGA AAGAAGTGAA AGTGAAGTGT GAAAAAAAGA ATGATGAAGC GGAGCAAATA
1a_Thai      TCGTTAATGA AAGAAGTGAA AGTGAAGTGT GAAAAAAAGA ATGATGAAGC GGAGCAAATA
Conservation ********** ********** ********** ********** ********** **********

             ....|....| ....|....| ....|....| ....|....| ....|....| ....|....|
                  4685       4695       4705       4715       4725       4735
1a_SalI      TTCGGCCAAA TTAAAACCGT AGATGGTAGA ATTAAAGCGC GAGTGAGTGA GAATGAAAGA
1a_Thai      TTCGGCCAAA TTAAAACCGT AGATGGTAGA ATTAAAGCGC GAGTGAGTGA GAATGAAAGA
Conservation ********** ********** ********** ********** ********** **********

             ....|....| ....|....| ....|....| ....|....| ....|....| ....|....|
                  4745       4755       4765       4775       4785       4795
1a_SalI      AAAATAAGCG AATTGAAGGA AAAAGCCAAA GTTGAGAAAA AGGAATCCTC GCAACTTAAC
1a_Thai      AAAATAAGCG AATTGAAGGA AAAAGCCAAA GTTGAGAAAA AGGAATCCTC GCAACTTAAC
Conservation ********** ********** ********** ********** ********** **********

             ....|....| ....|....| ....|....| ....|....| ....|....| ....|....|
                  4805       4815       4825       4835       4845       4855
1a_SalI      GATGTTTCCA CGAAGTCGTT ATTACAAATA GATAATTGCA GACAACAGCT TGACAGCGTT
1a_Thai      GATGTTTCCA CGAAGTCGTT ATTACAAATA GATAATTGCA GACAACAGCT TGACAGCGTT
Conservation ********** ********** ********** ********** ********** **********

             ....|....| ....|....| ....|....| ....|....| ....|....| ....|....|
                  4865       4875       4885       4895       4905       4915
1a_SalI      TTGTCAAACA TTGGAAGGGT GAAACAAAAT GCACTTCAAT ATTTCGATTC GGCTGATAAA
1a_Thai      TTGTCAAACA TTGGAAGGGT GAAACAAAAT GCACTTCAAT ATTTCGATTC GGCTGATAAA
Conservation ********** ********** ********** ********** ********** **********
```

```
             ....|....| ....|....| ....|....| ....|....| ....|....| ....|....|
                 4925       4935       4945       4955       4965       4975
1a_SalI      TCGATGAAGT CCGTTTTGCC TATAAGCGAA TTGGGTGCCG AAAAATCGCT AGACAAAGTA
1a_Thai      TCGATGAAGT CCGTTTTGCC TATAAGCGAA TTGGGTGCCG AAAAATCGCT AGACAAAGTA
Conservation ********** ********** ********** ********** ********** **********

             ....|....| ....|....| ....|....| ....|....| ....|....| ....|....|
                 4985       4995       5005       5015       5025       5035
1a_SalI      AAAGCGGCTA AGGAAAGTTA TGAGAAAAAT TTGGAAACCG TTCAAAATGA AATGAGTCGT
1a_Thai      AAAGCGGCTA AGGAAAGTTA TGAGAAAAAT TTGGAAACCG TTCAAAATGA AATGAGTCGT
Conservation ********** ********** ********** ********** ********** **********

             ....|....| ....|....| ....|....| ....|....| ....|....| ....|....|
                 5045       5055       5065       5075       5085       5095
1a_SalI      ATTAATGTGG AAGAAGGAAG TCTGACCGAC ATAGACAAAA AAATAACTGA CATAGAAAAT
1a_Thai      ATTAATGTGG AAGAAGGAAG TCTGACCGAC ATAGACAAAA AAATAACTGA CATAGAAAAT
Conservation ********** ********** ********** ********** ********** **********

             ....|....| ....|....| ....|....| ....|....| ....|....| ....|....|
                 5105       5115       5125       5135       5145       5155
1a_SalI      GACTTGCTAA AAATGAAGAA ACAATATGAA GAAGGGTTAC TACAAAAGAT TAAAGAAAAT
1a_Thai      GAATTGCTAA AAATGAAGAA ACAATATGAA GAAGGGTTAC TACAAAAGAT TAAGGAAAAT
Conservation **.******* ********** ********** ********** ********** ***.******

             ....|....| ....|....| ....|....| ....|....| ....|....| ....|....|
                 5165       5175       5185       5195       5205       5215
1a_SalI      GCGGATAAGA GGAAGAGTAA TTTCGAATTA GTAAGAAGCG AAATAAACGC CTTGCTGGAT
1a_Thai      GCGGATAAGA GGAAGAGTAA TTTCGAATTA GTAAGAAGCG AAATAAACGC CTTGCTGGAT
Conservation ********** ********** ********** ********** ********** **********

             ....|....| ....|....| ....|....| ....|....| ....|....| ....|....|
                 5225       5235       5245       5255       5265       5275
1a_SalI      CCAAGCACGT CTATTTTTAT TAAATTAAAA TTAAAGGAAT ATGACATGAC CGGCGATTTA
1a_Thai      CCAAGCACGT CTATTTTTAT TAAATTAAAA TTAAAGGAAT ATGACATGAC CGGCGATTTA
Conservation ********** ********** ********** ********** ********** **********

             ....|....| ....|....| ....|....| ....|....| ....|....| ....|....|
                 5285       5295       5305       5315       5325       5335
1a_SalI      AAAAATTACG CTGTTAAAAT GAATGAAATT CATGGTGAAT TTACCAAATC GTACAATTTG
1a_Thai      AAAAATTACG CTGTTAAAAT GAATGAAATT CATGGTGAAT TTACCAAATC GTACAATTTG
Conservation ********** ********** ********** ********** ********** **********

             ....|....| ....|....| ....|....| ....|....| ....|....| ....|....|
                 5345       5355       5365       5375       5385       5395
1a_SalI      ATAGAAACCC ATTTGTCCAA TGCTACAGAT TATTCTGTGA CGTTTGAGAA GGCCCAAAGT
1a_Thai      ATAGAAACCC ATTTGTCCAA TGCTACAGAT TATTCTGTGA CGTTTGAGAA GGCCCAAAGT
Conservation ********** ********** ********** ********** ********** **********

             ....|....| ....|....| ....|....| ....|....| ....|....| ....|....|
                 5405       5415       5425       5435       5445       5455
1a_SalI      TTAAGGGAAC TAGCAGAGAA GGAAGAAGAA CATCTCAGAA GAAGAGAGGA GGAAGCGATC
1a_Thai      TTAAGGGAAC TAGCAGAGAA GGAAGAAGAA CATCTCAGAA GAAGAGAGGA GGAAGCGATC
Conservation ********** ********** ********** ********** ********** **********

             ....|....| ....|....| ....|....| ....|....| ....|....| ....|....|
                 5465       5475       5485       5495       5505       5515
1a_SalI      AAACTGCTGA ATGATATTAA AAAGGTGGAA TCGTTAAAAC TGCTAAAAGA AATGATGAAA
1a_Thai      AAACTGCTGA ATGATATTAA AAAGGTGGAA TCGTTAAAAC TGCTAAAAGA AATGATGAAA
Conservation ********** ********** ********** ********** ********** **********
```

```
             ....|....| ....|....| ....|....| ....|....| ....|....| ....|....|
                  5525       5535       5545       5555       5565       5575
1a_SalI      AAGGTGAGTG CCGAATATGA AGGTATGAAA AGAGACCATA CGAGTGTTAG TCAGCTTGTA
1a_Thai      AAGGTGAGTG CCGAATATGA AGGTATGAAA AGAGACCATA CGAGTGTTAG TCAGCTTGTA
Conservation ********** ********** ********** ********** ********** **********

             ....|....| ....|....| ....|....| ....|....| ....|....| ....|....|
                  5585       5595       5605       5615       5625       5635
1a_SalI      CAGGATATGA AGACAATTGT TGATGAGCTG AAAACACTGA ATGATATAAG CGAATGTTCG
1a_Thai      CAGGATATGA AGACAATTGT TGATGAGCTG AAAACACTGA ATGATATAAG CGAATGTTCG
Conservation ********** ********** ********** ********** ********** **********

             ....|....| ....|....| ....|....| ....|....| ....|....| ....|....|
                  5645       5655       5665       5675       5685       5695
1a_SalI      AGCGTGCTAA ACAATGTAGT TAGTATAGTT AAAAAGGTTA AAGAGTCGAA ACATGCAGAC
1a_Thai      AGCGTGCTAA ACAATGTAGT TAGTATAGTT AAAAAGGTTA AAGAGTCGAA ACATGCAGAC
Conservation ********** ********** ********** ********** ********** **********

             ....|....| ....|....| ....|....| ....|....| ....|....| ....|....|
                  5705       5715       5725       5735       5745       5755
1a_SalI      TATAAGAGAG ACGCGAATAG CATGTATGAA AGTATGGTAA CTCTGGCAAA TTATTTCCTA
1a_Thai      TATAAGAGAG ACGCGAATAG CATGTATGAA AGTATGGTAA CTCTGGCAAA TTATTTCCTA
Conservation ********** ********** ********** ********** ********** **********

             ....|....| ....|....| ....|....| ....|....| ....|....| ....|....|
                  5765       5775       5785       5795       5805       5815
1a_SalI      AGCGATGAGG CTAAAATTTC ATCAGGAATG GAATTCAATG CGGAAATGAA ATCCAATTTT
1a_Thai      AGCGATGAGG CTAAAATTTC ATCAGGAATG GAATTCAATG CGGAAATGAA ATCCAATTTT
Conservation ********** ********** ********** ********** ********** **********

             ....|....| ....|....| ....|....| ....|....| ....|....| ....|....|
                  5825       5835       5845       5855       5865       5875
1a_SalI      AAGACAGACT TAGAGTTGGA GATCTTTTCG GTAATATCCA ATTCGAATGA ACTTTTAAAA
1a_Thai      AAGACAGACT TAGAGTTGGA GATCTTTTCG GTAATATCCA ATTCGAATGA ACTTTTAAAA
Conservation ********** ********** ********** ********** ********** **********

             ....|....| ....|....| ....|....| ....|....| ....|....| ....|....|
                  5885       5895       5905       5915       5925       5935
1a_SalI      AAAATAGAAC AGGATTCAAA TGATGTAATA CAAAAGGAAA GAGAAAGTGA ACAGCTAGCT
1a_Thai      AAAATAGAAC AGGATTCAAA TGATGTAATA CAAAAGGAAA GAGAAAGTGA ACAGCTAGCT
Conservation ********** ********** ********** ********** ********** **********

             ....|....| ....|....| ....|....| ....|....| ....|....| ....|....|
                  5945       5955       5965       5975       5985       5995
1a_SalI      AAGGATGCCA CTGATATTTA TAATGTGATA AAACTAAAAA ATGAATTTAA CGAAAAGTTG
1a_Thai      AAGGATGCCA CTGATATTTA TAATGTGATA AAACTAAAAA ATGAATTTAA CGAAAAGTTG
Conservation ********** ********** ********** ********** ********** **********

             ....|....| ....|....| ....|....| ....|....| ....|....| ....|....|
                  6005       6015       6025       6035       6045       6055
1a_SalI      GAAGAAGCCA AGAACAAAGA AGAGGTCGTT TCGGAAAAGG TAAGGGAAGC ATTGAAGAGA
1a_Thai      GAAGAAGCCA AGAACAAAGA AGAGGTCGTT TCGGAAAAGG TAAGGGAAGC ATTGAAGAGA
Conservation ********** ********** ********** ********** ********** **********

             ....|....| ....|....| ....|....| ....|....| ....|....| ....|....|
                  6065       6075       6085       6095       6105       6115
1a_SalI      CTGAGTCAAG TGGAAGGAAT AAGATGCCAC TTTGAAAATT TCCATAGGTT ACTAGACAAT
1a_Thai      CTGAGTCAAG TGGAAGGAAT AAGATGCCAC TTTGAAAATT TCCATAGGTT ACTAGACAAT
Conservation ********** ********** ********** ********** ********** **********
```

```
             ....|....| ....|....| ....|....| ....|....| ....|....| ....|....|
                  6125       6135       6145       6155       6165       6175
1a_SalI      ACAGAGGAGC TTGAAAATTT AAAAAAAATG GTTACCATCT ACCGGGATAA GAAAAGCGAA
1a_Thai      ACAGAGGAGC TTGAAAATTT AAAAAAAATG GTTACCATCT ACCGGGATAA GAAAAGCGAA
Conservation ********** ********** ********** ********** ********** **********

             ....|....| ....|....| ....|....| ....|....| ....|....| ....|....|
                  6185       6195       6205       6215       6225       6235
1a_SalI      GCGCCGAAAG AATCAGGGTT GCAAGAAATG GAGAACGAAA TGAACACCTA TAGTAACTCC
1a_Thai      GCGCCGAAAG AATCAGGGTT GCAAGAAATG GAGAACGAAA TGAACACCTA TAGTAACTCC
Conservation ********** ********** ********** ********** ********** **********

             ....|....| ....|....| ....|....| ....|....| ....|....| ....|....|
                  6245       6255       6265       6275       6285       6295
1a_SalI      ATAACACAGT TAGAAGGAAT AGTCGTATCA GCCGGGGAAT CCAAAGAAGA CATTGAAAAA
1a_Thai      ATAACACAGT TAGAAGGAAT AGTCGTATCA GCCGGGGAAT CCAAAGAAGA CATTGAAAAA
Conservation ********** ********** ********** ********** ********** **********

             ....|....| ....|....| ....|....| ....|....| ....|....| ....|....|
                  6305       6315       6325       6335       6345       6355
1a_SalI      TTGGAAAGAT CCAATGAAGA AATGAGAAAT ATTAGCGAAA AGATAAGCAC AATAGATTCG
1a_Thai      TTGGAAAGAT CCAATGAAGA AATGAGAAAT ATTAGCGAAA AGATAAGCAC AATAGATTCG
Conservation ********** ********** ********** ********** ********** **********

             ....|....| ....|....| ....|....| ....|....| ....|....| ....|....|
                  6365       6375       6385       6395       6405       6415
1a_SalI      AAGGTTATCG AAATGAACTC AACTATTGAT GAGTTGTACA AATTGGGAAA AAACTGTCAG
1a_Thai      AAGGTTATCG AAATGAACTC AACTATTGAT GAGTTGTACA AATTGGGAAA AAACTGTCAG
Conservation ********** ********** ********** ********** ********** **********

             ....|....| ....|....| ....|....| ....|....| ....|....| ....|....|
                  6425       6435       6445       6455       6465       6475
1a_SalI      GCCCACTGGA TTTCCCTGAT CAGTTATACT GCGAATATGA AAACGTCCAA AAAGTTAATA
1a_Thai      GCCCACTGGA TTTCCCTGAT CAGTTATACT GCGAATATGA AAACGTCCAA AAAGTTAATA
Conservation ********** ********** ********** ********** ********** **********

             ....|....| ....|....| ....|....| ....|....| ....|....| ....|....|
                  6485       6495       6505       6515       6525       6535
1a_SalI      ATGATTAATA AAGAAAAGGA GAACACGGAA AAGTGTGTAG ACTACATTAA AGACAATTCG
1a_Thai      ATGATTAATA AAGAAAAGGA GAACACGGAA AAGTGTGTAG ACTACATTAA AGACAATTCG
Conservation ********** ********** ********** ********** ********** **********

             ....|....| ....|....| ....|....| ....|....| ....|....| ....|....|
                  6545       6555       6565       6575       6585       6595
1a_SalI      TCCTCCACAG ACGGTTATGT CGAAACGTTG AAAGGGTTTT ACGGTAGTAA GCTTACATTT
1a_Thai      TCCTCCACAG ACGGTTATGT CGAAACGTTG AAAGGGTTTT ACGGTAGTAA GCTTACATTT
Conservation ********** ********** ********** ********** ********** **********

             ....|....| ....|....| ....|....| ....|....| ....|....| ....|....|
                  6605       6615       6625       6635       6645       6655
1a_SalI      AGCAGCGCAT CCGAAATTGT GCAAAATGCA GACACATATT CCGTGAATTT TGCGAAACAC
1a_Thai      AGCAGCGCAT CCGAAATTGT GCAAAATGCA GACACATATT CCGTGAATTT TGCGAAACAC
Conservation ********** ********** ********** ********** ********** **********

             ....|....| ....|....| ....|....| ....|....| ....|....| ....|....|
                  6665       6675       6685       6695       6705       6715
1a_SalI      GAAAAGGAGT CTTTAAATGC GATAAGGGAT ATAAAAAAGG AATTATATTT ATTCCACCAA
1a_Thai      GAAAAGGAGT CTTTAAATGC GATAAGGGAT ATAAAAAAGG AATTATATTT ATTCCACCAA
Conservation ********** ********** ********** ********** ********** **********
```

```
                ....|....| ....|....| ....|....| ....|....| ....|....| ....|....|
                    6725       6735       6745       6755       6765       6775
1a_SalI         AATAGCGATA TTAGCATTGT AGAGGGAGGC GTCCAAAATG TGCTGGCACT TTATGATAAG
1a_Thai         AATAGCGATA TTAGCATTGT AGAGGGAGGC GTCCAAAATG TGCTGGCACT TTATGATAAG
Conservation    ********** ********** ********** ********** ********** **********

                ....|....| ....|....| ....|....| ....|....| ....|....| ....|....|
                    6785       6795       6805       6815       6825       6835
1a_SalI         CTGAACGAGG AAAAAAGAGA AATGGATGAA CTGTACAGAA ATATAAGTGA AACTAAGCTG
1a_Thai         CTGAACGAGG AAAAAAGAGA AATGGATGAA CTGTACAGAA ATATAAGTGA AACTAAGCTG
Conservation    ********** ********** ********** ********** ********** **********

                ....|....| ....|....| ....|....| ....|....| ....|....| ....|....|
                    6845       6855       6865       6875       6885       6895
1a_SalI         AAGCAAATGG AACACAGCAC TGACGTGTTT AAGCCCATGA TAGAATTACA CAAAGGAATG
1a_Thai         AAGCAAATGG AACACAGCAC TGACGTGTTT AAGCCCTTGA TAGAATTACA CAAAGGATTG
Conservation    ********** ********** ********** ******:*** ********** *******:**

                ....|....| ....|....| ....|....| ....|....| ....|....| ....|....|
                    6905       6915       6925       6935       6945       6955
1a_SalI         AATGAAACGA ATAATAAGTC TTTGCTGGAG AAAGAAAAGA AACTAAAAAG CGTGAACGAC
1a_Thai         ATTGAAACGA ATAATAAGTC TTTGCTGGAG AAAGAAANGA ACTTAAAAAG CGTGAACGAC
Conservation    *:******** ********** ********** ******* ** *. ******* **********

                ....|....| ....|....| ....|....| ....|....| ....|....| ....|....|
                    6965       6975       6985       6995       7005       7015
1a_SalI         AATATGCACA GTATGGAAGC TGAGATGATT AAAAATGGCC TTAAATACAC CCCAGAAAGT
1a_Thai         AATATGCACA GTATGGAAGC TGAGATGATT AAAAATGGCC TTAAATACAC CCCAGAAAGT
Conservation    ********** ********** ********** ********** ********** **********

                ....|....| ....|....| ....|....| ....|....| ....|....| ....|....|
                    7025       7035       7045       7055       7065       7075
1a_SalI         GTGCAAAATA TTAACAACAT ATACAGCGTT ATTGAAGCTG AGGTGAAAAC GCTGGAAGAA
1a_Thai         GTGCAAAATA TTAACAACAT ATACAGCGTT ATTGAAGCTG AGGTGAAAAC GCTGGAAGAA
Conservation    ********** ********** ********** ********** ********** **********

                ....|....| ....|....| ....|....| ....|....| ....|....| ....|....|
                    7085       7095       7105       7115       7125       7135
1a_SalI         ATTGACCGTG ATTATGGCGA TAATTACCAA ATCGTGGAAG AGCACAAGAA GCAATTTTCC
1a_Thai         ATTGACCGTG ATTATGGCGA TAATTACCAA ATCGTGGAAG AGCACAAGAA GCAATTTTCC
Conservation    ********** ********** ********** ********** ********** **********

                ....|....| ....|....| ....|....| ....|....| ....|....| ....|....|
                    7145       7155       7165       7175       7185       7195
1a_SalI         ATTTTAATCG ACAGAACGAA CGCGCTAATG GATGACATTG AAATTTTTAA AAAGGAAAAC
1a_Thai         ATTTTAATCG ACAGAACGAA CGCGCTAATG GATGACATTG AAATTTTTAA AAAGGAAAAC
Conservation    ********** ********** ********** ********** ********** **********

                ....|....| ....|....| ....|....| ....|....| ....|....| ....|....|
                    7205       7215       7225       7235       7245       7255
1a_SalI         AATTACAATT TAATGGAAGT AAACACAGAA ACGATACACA GAGTAAACGA TTATATAGAA
1a_Thai         AATTACAATT TAATGGAAGT AAACACAGAA ACCATACACA GAGTAAACGA TTATATAGAA
Conservation    ********** ********** ********** ** ******* ********** **********

                ....|....| ....|....| ....|....| ....|....| ....|....| ....|....|
                    7265       7275       7285       7295       7305       7315
1a_SalI         AAGATCACCA ATAAGTTAGT ACAAGCCAAA ACGGAGTATG AACAGATCCT GGAAAATATA
1a_Thai         AAGATCACCA ATAAGTTAGT ACAAGCCAAA ACGGAGTATG AACAGATCCT GGAAAATATA
Conservation    ********** ********** ********** ********** ********** **********
```

```
               ....|....| ....|....| ....|....| ....|....| ....|....| ....|....|
                   7325       7335       7345       7355       7365       7375
1a_SalI        AAACAAAACG ATGATATGCT ACAGAATATT TTTCTCAAAA AAGTAAGTAT TATCGAATAC
1a_Thai        AAACAAAACG ATGATATGCT ACAGAATATT TTTCTCAAAA AAGTAAGTAT TATCGAATAC
Conservation   ********** ********** ********** ********** ********** **********

               ....|....| ....|....| ....|....| ....|....| ....|....| ....|....|
                   7385       7395       7405       7415       7425       7435
1a_SalI        TTTGAAAACG TAAAGAAGAA AAAAGAATCT ATATTGAACG ATTTATATGA ACAGGAAAGG
1a_Thai        TTTGAAAACG TAAAGAAGAA AAAAGAATCT ATATTGAACG ATTTATATGA ACAGGAAAGG
Conservation   ********** ********** ********** ********** ********** **********


               ....|....| ....|....| ....|....| ....|....| ....|....| ....|....|
                   7445       7455       7465       7475       7485       7495
1a_SalI        CTGCTCAAAA TAGGGGAACA CTTAGATGAG ATTAAGCGTA ACGTGACAGA GACACTGAGT
1a_Thai        CTGCTCAAAA TAGGGGAACA CTTAGATGAG ATTAAGCGTA ACGTGACAGA GACACTGAGT
Conservation   ********** ********** ********** ********** ********** **********

               ....|....| ....|....| ....|....| ....|....| ....|....| ....|....|
                   7505       7515       7525       7535       7545       7555
1a_SalI        AGTTATGAAA TTGATCAAAA AATGGAAATG ATGTCTAAAA ACCTTCTAGA GAAAAAAAGC
1a_Thai        AGTTATGAAA TTGATCAAAA AATGGAAATG ATGTCTAAAA ACCTTCTAGA GAAAAAAATC
Conservation   ********** ********** ********** ********** ********** ******** *

               ....|....| ....|....| ....|....| ....|....| ....|....| ....|....|
                   7565       7575       7585       7595       7605       7615
1a_SalI        AAAATGATGA ATTACACTTC CATTTATGAG TTGGAGAGAG AAGCGAACGA AATAAACAGG
1a_Thai        AAAATGATGA ATTACACTTC CATTTATGAG TTGGAGAGAG AAGCGAACGA AATAAACAGG
Conservation   ********** ********** ********** ********** ********** **********

               ....|....| ....|....| ....|....| ....|....| ....|....| ....|....|
                   7625       7635       7645       7655       7665       7675
1a_SalI        GATGCTAAAC AAATAAAAGA TGATGATACA ATACTTAATA GTGTTTTAGA AGCTGCCATT
1a_Thai        GATGCTAAAC AAATAAAAGA TGATGATACA ATACTTAATA GTGTTTTAGA AGCTGCCATT
Conservation   ********** ********** ********** ********** ********** **********

               ....|....| ....|....| ....|....| ....|....| ....|....| ....|....|
                   7685       7695       7705       7715       7725       7735
1a_SalI        CAGAAGAGGG GAGATATGGA CGCGATCTTC AGTCAGATGT CTGCAGATAG AAATCCAAAT
1a_Thai        CAGAAGAGGG GAGATATGGA CGCGATCTTC AGTCAGATGT CTGCAGATAG AAATCCAAAT
Conservation   ********** ********** ********** ********** ********** **********

               ....|....| ....|....| ....|....| ....|....| ....|....| ....|....|
                   7745       7755       7765       7775       7785       7795
1a_SalI        GAATATAAAT CTGCAGAGAA GTACATGAAT GAAGCGAATG AAATAATTCG CCAATTAGAA
1a_Thai        GAATATAAAT CTGCAGAGAA GTACATGAAT GAAGCGAATG AAATAATTCG CCAATTAGAA
Conservation   ********** ********** ********** ********** ********** **********

               ....|....| ....|....| ....|....| ....|....| ....|....| ....|....|
                   7805       7815       7825       7835       7845       7855
1a_SalI        GTAAAGCTTC GAGAAATAGG CCAACTAGTA CAGGATAGTG AAAGCATATT ATCAGAAATG
1a_Thai        GTAAAGCTTC GAGAAATAGG CCAACTAGTA CAGGATAGTG AAAGCATATT ATCAGAAATG
Conservation   ********** ********** ********** ********** ********** **********

               ....|....| ....|....| ....|....| ....|....| ....|....| ....|....|
                   7865       7875       7885       7895       7905       7915
1a_SalI        AATTCTAAAA AGAGCGCTAT AGAAAAGGAA AAGACAGCTA GGGCTTTGAG AACTAGTGAA
1a_Thai        AATTCTAAAA AGAGCGCTAT AGAAAAGGAA AAGACAGCTA GGGCTTTGAG AACTAGTGAA
Conservation   ********** ********** ********** ********** ********** **********
```

```
             ....|....| ....|....| ....|....| ....|....| ....|....| ....|....|
                  7925       7935       7945       7955       7965       7975
1a_SalI      AATAATAGAA GAGAAGAGGA AGAAAGGGCA CGTGTGCAAG AAATGAGCAT GAATAATGAT
1a_Thai      AATAATAGAA GAGAAGAGGA AGAAAGGGCA CGTGTGCAAG AAATGAGCAT GAATAATGAT
Conservation ********** ********** ********** ********** ********** **********

             ....|....| ....|....| ....|....| ....|....| ....|....| ....|....|
                  7985       7995       8005       8015       8025       8035
1a_SalI      CCCACGCAAA GTGAAACAAC TCATTCGGAG GGATCCATCG GTGAAGGAAA AGAATCAGAC
1a_Thai      CCCACGCAAA GTGAAACAAC TCATTCGGAG GGATCCATCG GTGAAGGAAA AGAATCAGAC
Conservation ********** ********** ********** ********** ********** **********

             ....|....| ....|....| ....|....| ....|....| ....|....| ....|....|
                  8045       8055       8065       8075       8085       8095
1a_SalI      TCGGATGAAA CAGGTTTAAC ACACGATGCA GGTGCAGATG AAGATTCTAC TAGCTCGGCA
1a_Thai      TCGGATGAAA CAGGTTTAAC ACACGATGCA GGTGCAGATG AAGATTCTAC TAGCTCGGCA
Conservation ********** ********** ********** ********** ********** **********

             ....|....| ....|....| ....|....| ....|....| ....|....| ....|....|
                  8105       8115       8125       8135       8145       8155
1a_SalI      AAAGGGGCAC ATGAATTAGA AGAGGAGGAA ACTACAGCAC CTATGGAAGA AACCGAGATG
1a_Thai      AAAGGGGCAC ATGAATTAGA AGAGGAGGAA ACTACAGCAC CTATGGAAGA AACCGAGATG
Conservation ********** ********** ********** ********** ********** **********

             ....|....| ....|....| ....|....| ....|....| ....|....| ....|....|
                  8165       8175       8185       8195       8205       8215
1a_SalI      AATGACAATA CCCTTCTAGG GTATGACACT ACCAGAAGTG ATGAACCTGA TATGCATACA
1a_Thai      AATGACAATA CCCTTCTAGG GTATGACACT ACCAGAAGTG ATGAACCTGA TATGCATACA
Conservation ********** ********** ********** ********** ********** **********

             ....|....| ....|....| ....|....| ....|....| ....|....| ....|....|
                  8225       8235       8245       8255       8265       8275
1a_SalI      GAGAACACCC AGGATGGTAC CTATCAGGAT ACGTCAAATT CCAGCGATGA AGCAGATATT
1a_Thai      GAGAACACCC AGGATGGTAC CTATCAGGAT ACGTCAAATT CCAGCGATGA AGCAGATATT
Conservation ********** ********** ********** ********** ********** **********

             ....|....| ....|....| ....|....| ....|....| ....|....| ....|....|
                  8285       8295       8305       8315       8325       8335
1a_SalI      TTGAACGGCA AGTTCAATTT TAATAATGTC AAATATGCAG GAGCATTCGT TTTACTGTGT
1a_Thai      TTGAACGGCA AGTTCAATTT TAATAATGTC AAATATGCAG GAGCATTCGT TTTACTGTGT
Conservation ********** ********** ********** ********** ********** **********

             ....|....| ....|....| ....|....| ....|....| ....|....| ....|....|
                  8345       8355       8365       8375       8385       8395
1a_SalI      ACGAGCGCAG TTATAGGGGC TATTATAGCA CATAAGAAAG ATGACCAAGA GGAGCTTAAT
1a_Thai      ACGAGCGCAG TTATAGGGGC TATTATAGCA CATAAGAAAG ATGACCAAGA GGAGCTTAAT
Conservation ********** ********** ********** ********** ********** **********

             ....|....| ....|....| ....|....| ....|....| ....|....| ....|....|
                  8405       8415       8425       8435       8445       8455
1a_SalI      AATGGCGTGG AAGACGATAG GGTGTTTGAA GTTAAAAAGA GCATGAACCC AGAAAACAAA
1a_Thai      AATGGCGTGG AAGACGATAG GGTGTTTGAA GTTAAAAAGA GCATGAACCC AGAAAACAAA
Conservation ********** ********** ********** ********** ********** **********

             ....|....| ....|....| ....|....| ....|....| ..
                  8465       8475       8485       8495
1a_SalI      GAAGAAATCA TTGATGTTTC TTTTGTTGAT ATTGAGTATT AA
1a_Thai      GAAGAAATCA TTGATGTTTC TTTTGTTGAT ATTGAGTATT AA
Conservation ********** ********** ********** ********** **
```

Alignment: Pvrbp1b

```
             ....|....| ....|....| ....|....| ....|....| ....|....| ....|....|
                 5         15         25         35         45         55
1b_SalI      ATGAAGAAGC CAATTTATTG GACAGCCCTC TTTGGTCTAT GGCTTGCATC TCTTGATTTA
1b_Thai      ATGAAGAAGC CAATTTATAG GACAGCCCTC TTTGGTCTAT GGCTTGCATC TCTTGATTTA
Conservation ********** ********:* ********** ********** ********** **********

             ....|....| ....|....| ....|....| ....|....| ....|....| ....|....|
                65         75         85         95        105        115
1b_SalI      AATCGAGGGA AAAACACGTT GCATAATATA AAGAAACATT TTGAAGACTC GAAATTTTTA
1b_Thai      AATCGAGGGA AAAACACGTT GCATAATATA AAGAAACATT TTGAAGACTC GAAATTTTTA
Conservation ********** ********** ********** ********** ********** **********

             ....|....| ....|....| ....|....| ....|....| ....|....| ....|....|
                125        135        145        155        165        175
1b_SalI      TCATTCGATC CGAATTTGAA GAGAAACAAA AAGTTCAAGC ATAAGGACGT GAAAAAGAAG
1b_Thai      TCATTCGATC CGAATTTGAA GAGAAACAAA AAGTTCAAGC ATAAGGACGT GAAAAAGAAG
Conservation ********** ********** ********** ********** ********** **********

             ....|....| ....|....| ....|....| ....|....| ....|....| ....|....|
                185        195        205        215        225        235
1b_SalI      AGCTTTAAAA GTCTGGAAAC AGATAGTGTA CAGCAGTTTG ATAAAGAAAT TAGCAAAGTT
1b_Thai      AGCTTTAAAA GTCTGGAAAC AGATAGTGTA CAGCAGTTTG ATAAAGAAAT TAGCAAAGTT
Conservation ********** ********** ********** ********** ********** **********

             ....|....| ....|....| ....|....| ....|....| ....|....| ....|....|
                245        255        265        275        285        295
1b_SalI      AATAGGGAGG ACCCAACAGG AATAATCGAA TCTGATAGTG CTTCATTTAT AAGCTTAGGA
1b_Thai      AATAGGGAGG ACCCAACAGG AATAATCGAA TCTGATAGTG CTTCATTTAT AAGCTTAGGA
Conservation ********** ********** ********** ********** ********** **********

             ....|....| ....|....| ....|....| ....|....| ....|....| ....|....|
                305        315        325        335        345        355
1b_SalI      AATGCTAGTC TGAAAAATGA ACCCTCTGAC GGTGGTGGAG ATGAAGTGGT TTTAAGAACC
1b_Thai      AATGCTAGTC TGAAAAATGA ACCCTCTGAC GGTGGTGGAG ATGAAGTGGT TTTAAGAACC
Conservation ********** ********** ********** ********** ********** **********

             ....|....| ....|....| ....|....| ....|....| ....|....| ....|....|
                365        375        385        395        405        415
1b_SalI      GACGGAGGGG GTAGTGACAC CGATAAAGAT TCAGGCAAAT TGGAGAAAAG TGAAATACCT
1b_Thai      GACGGAGGGG GTAGTGACAC CGATAATGAT TCAGGCAAAT TGGAGAAAAG TGAAATACCT
Conservation ********** ********** ******.*** ********** ********** **********

             ....|....| ....|....| ....|....| ....|....| ....|....| ....|....|
                425        435        445        455        465        475
1b_SalI      GATGTGGAGA ACGCAGAATC GGTATCGAAA TATAAAATCG TTTTAGAAGA TTATGTAAAT
1b_Thai      GATGTGGAGA ACGCAGAATC GGTATCGAAA TATAAAATCG TTTTAGAAGA TTATGTAAAT
Conservation ********** ********** ********** ********** ********** **********

             ....|....| ....|....| ....|....| ....|....| ....|....| ....|....|
                485        495        505        515        525        535
1b_SalI      AAACTGAAAG AATCTAATCC ATATTTCTTG ATGGAGGTTG ATTATGTTAA TCTGCAGTCT
1b_Thai      AAACTGAAAG AATCTAATCC ATATTTCTTG ATGGAGGTTG ATTATGTTAA TCTGCAGTCT
Conservation ********** ********** ********** ********** ********** **********

             ....|....| ....|....| ....|....| ....|....| ....|....| ....|....|
                545        555        565        575        585        595
1b_SalI      TTTAAAGAAA TTAGGAACCT CATTCCAGAG GGGAAAGAAT ACCACAAGTT CTACGATGAG
1b_Thai      TTTAAAGAAA TTAGGAACCT CATTCCAGAG GGGAAAGAAT ACCACAAGTT CTACGATGAG
Conservation ********** ********** ********** ********** ********** **********

             ....|....| ....|....| ....|....| ....|....| ....|....| ....|....|
```

```
                605        615        625        635        645        655
1b_SalI      GAGATGGATA AAAAAGTCAC AGATTACACA AATAGATTAG ACGCACTGAT GAAAAAGTTC
1b_Thai      GAGATGGATA AAAAAGTCAC AGATTACACA AATAGATTAG ACGCACTGAT GAAAAAGTTC
Conservation ********** ********** ********** ********** ********** **********

             ....|....| ....|....| ....|....| ....|....| ....|....| ....|....|
                665        675        685        695        705        715
1b_SalI      ATTGGAGCGA AAAATGAAAT GATAAAAATG GATGCTATCG TAAAAATTCA TAAAAAGGGT
1b_Thai      ATTGGAGCGA AAAATGAAAT GATAAAAATG GATGCTATCG TAAAAATTCA TAAAAAGGGT
Conservation ********** ********** ********** ********** ********** **********

             ....|....| ....|....| ....|....| ....|....| ....|....| ....|....|
                725        735        745        755        765        775
1b_SalI      TCTACCGAAT TGAAGCAAGA AGCTGAAAAT GTAAAAAAAT TAGAATTAGC AAAGGAGCAA
1b_Thai      TCTACCGAAT TGAAGCAAGA AGCTGAAAAT GTAAAAAAAT TAGAATTAGC AAAGGAGCAA
Conservation ********** ********** ********** ********** ********** **********

             ....|....| ....|....| ....|....| ....|....| ....|....| ....|....|
                785        795        805        815        825        835
1b_SalI      TATGAAAAGT ACATGAAAGA GTATAATGAT GATATAAAAC CTATTGTACA TGAAATTAGA
1b_Thai      TATGAAAAGT ACATGAAAGA GTATAATGAT GATATAAAAC CTATTGTACA TGAAATTAGA
Conservation ********** ********** ********** ********** ********** **********

             ....|....| ....|....| ....|....| ....|....| ....|....| ....|....|
                845        855        865        875        885        895
1b_SalI      AACAAGGCAT ATGAGCATTT GAAGCAATCC AGCTGCACGC ACCAGTGTAA TGCTTACATT
1b_Thai      AACAAGGCAT ATGAGCATTT GAAGCAATCC AGCTGCACGC ACCAGTGTAA TGCTTACATT
Conservation ********** ********** ********** ********** ********** **********

             ....|....| ....|....| ....|....| ....|....| ....|....| ....|....|
                905        915        925        935        945        955
1b_SalI      ATGAATTATG CTCGTCTTCT TAACAAATAT ATAAAGAACA TTTCAGAGAC TACGGACGAG
1b_Thai      ATGAATTATG CTCGTCTTCT TAACAAATAT ATAAAGAACA TTTCAGAGAC TACGGACGAG
Conservation ********** ********** ********** ********** ********** **********

             ....|....| ....|....| ....|....| ....|....| ....|....| ....|....|
                965        975        985        995       1005       1015
1b_SalI      TCAGTTGTCA CTATCGTTAA AAGTATTAAC GATTATAATT CTCTGGACAA AATATTAGAA
1b_Thai      TCAGTTGTCA CTATCGTTAA AAGTATTAAC GATTATAATT CTCTGGACAA AATATTAGAA
Conservation ********** ********** ********** ********** ********** **********

             ....|....| ....|....| ....|....| ....|....| ....|....| ....|....|
               1025       1035       1045       1055       1065       1075
1b_SalI      CTTGCAGAAA GGGAGAATAT AGACATTGCA GAAAATGTCT ACCTTTTGAA GTTACTAGGA
1b_Thai      CTTGCAGAAA GGGAGAATAT AGACATTGCA GAAAATGTCT ACCTTTTGAA GTTACTAGGA
Conservation ********** ********** ********** ********** ********** **********

             ....|....| ....|....| ....|....| ....|....| ....|....| ....|....|
               1085       1095       1105       1115       1125       1135
1b_SalI      GAAGAAGTAA ATGACCTTAA CTATGTGTAT GTGATAAACA GATCGCTAAT AAATGACGCG
1b_Thai      GAAGAAGTAA ATGACCTTAA CTATGTGTAT GTGATAAACA GATCGCTAAT AAATGACGCG
Conservation ********** ********** ********** ********** ********** **********

             ....|....| ....|....| ....|....| ....|....| ....|....| ....|....|
               1145       1155       1165       1175       1185       1195
1b_SalI      AATAAAGTTC TGATGGACAT AAAGGAAAAT GGGATTTTAT TTCATACGAA GGAGGATAAA
1b_Thai      AATAAAGTTC TGATGGACAT AAAGGAAAAT GGGATTTTAT TTCATACGAA GGAGGATAAA
Conservation ********** ********** ********** ********** ********** **********

             ....|....| ....|....| ....|....| ....|....| ....|....| ....|....|
               1205       1215       1225       1235       1245       1255
1b_SalI      ATTGTAAACA GTACAAAGGA ACTAATCAAC AACTATTGTG TGTTTCACCA TATAATGATT
1b_Thai      ATTGTAAACA GTACAAAGGA ACTAATCAAC AACTATTGTG TGTTTCACCA TATAATGATT
```

```
Conservation ********** ********** ********** ********** ********** **********

             ....|....| ....|....| ....|....| ....|....| ....|....| ....|....|
                 1265       1275       1285       1295       1305       1315
1b_SalI      TTGAATGTTC CAATTAAAAA AATGTACGAA GAAAGGATAA AAAGGTCTAA CGATTTATTT
1b_Thai      TTGAATGTTC CAATTAAAAA AATGTACGAA GAAAGGATAA AAAGGTCTAA CGATTTATTT
Conservation ********** ********** ********** ********** ********** **********


             ....|....| ....|....| ....|....| ....|....| ....|....| ....|....|
                 1325       1335       1345       1355       1365       1375
1b_SalI      TCTACAATTA TGGAAAAGTT AAAAGATGAA GCTCATAAAT TAATCAATTC TGTATTTGTC
1b_Thai      TCTACAATTA TGGAAAAGTT AAAAGATGAA GCTCATAAAT TAATCAATTC TGTATTTGTC
Conservation ********** ********** ********** ********** ********** **********

             ....|....| ....|....| ....|....| ....|....| ....|....| ....|....|
                 1385       1395       1405       1415       1425       1435
1b_SalI      GAGGAGGAAA GTAATAAAAT TCTTAAGTCG TCAGAAGGAA TTGTCCAAAA TGCAGAAAAA
1b_Thai      GAGGAGGAAA GTAATAAAAT TCTTAAGTCG TCAGAAGGAA TTGTCCAAAA TGCAGAAAAA
Conservation ********** ********** ********** ********** ********** **********

             ....|....| ....|....| ....|....| ....|....| ....|....| ....|....|
                 1445       1455       1465       1475       1485       1495
1b_SalI      ATACTTGAGG AAAATAAAAA AAAAATAGAC TTTTTTAAAA GGTATCCAGA AATAAAAACG
1b_Thai      ATACTTGAGG AAAATAAAAA AAAAATAGAC TTTTTTAAAA GGTATCCAGA AATAAAAACG
Conservation ********** ********** ********** ********** ********** **********

             ....|....| ....|....| ....|....| ....|....| ....|....| ....|....|
                 1505       1515       1525       1535       1545       1555
1b_SalI      AAGCCTCCTA TGGAAGAATT ACAAACTGAG TTTAGTCAAC AAGAAGAAGC GAGGGGGGAA
1b_Thai      AAGCCTCCTA TGGAAGAATT ACAAACTGAG TTTAGTCAAC AAGAAGAAGC GAGGGGGGAA
Conservation ********** ********** ********** ********** ********** **********

             ....|....| ....|....| ....|....| ....|....| ....|....| ....|....|
                 1565       1575       1585       1595       1605       1615
1b_SalI      ATATTAAACA TGGCACAGCT AATCGATCAC TTATATAATA ATTTGTTTCA TGTGAACAAA
1b_Thai      ATATTAAACA TGGCACAGCT AATCGATCAC TTATATAATA ATTTGTTTCA TGTGAACAAA
Conservation ********** ********** ********** ********** ********** **********

             ....|....| ....|....| ....|....| ....|....| ....|....| ....|....|
                 1625       1635       1645       1655       1665       1675
1b_SalI      ACTAATCATT TAAATAAATT AAACGAAATA GCTCAGTACG GGGATAGCAT AACAAAGGGA
1b_Thai      ACTAATCATT TAAATAAATT AAACGAAATA GCTCAGTACG GGGATAGCAT AACAAAGGGA
Conservation ********** ********** ********** ********** ********** **********

             ....|....| ....|....| ....|....| ....|....| ....|....| ....|....|
                 1685       1695       1705       1715       1725       1735
1b_SalI      AATTTACTGT TGGAAGAAAT TTCTGAAATT AATAAAGAGC GGAATGTTCT AAAGGTAAAT
1b_Thai      AATTTACTGT TGGAAGAAAT TTCTGAAATT AATAAAGAGC GGAATGTTCT AAAGGTAAAT
Conservation ********** ********** ********** ********** ********** **********

             ....|....| ....|....| ....|....| ....|....| ....|....| ....|....|
                 1745       1755       1765       1775       1785       1795
1b_SalI      ATTGCTACTT TGAAAAATGT GCATGGGAAT TTGGTACGTT CGAAAAATAA CATTGTTGAA
1b_Thai      ATTGCTACTT TGAAAAATGT GCATGGGAAT TTGGTACGTT CGAAAAATAA CATTGTTGAA
Conservation ********** ********** ********** ********** ********** **********

             ....|....| ....|....| ....|....| ....|....| ....|....| ....|....|
                 1805       1815       1825       1835       1845       1855
1b_SalI      ATTGTAATAA ATGTGGACAA CACTTCTGAG GTGGACGAAT TGAAGGAAAA GGAAGAGAAG
1b_Thai      ATTGTAATAA ATGTGGACAA CACTTCTGAG GTGGACGAAT TGAAGGAAAA GGAAGAGAAG
Conservation ********** ********** ********** ********** ********** **********
```

```
             ....|....| ....|....| ....|....| ....|....| ....|....| ....|....|
                  1865       1875       1885       1895       1905       1915
1b_SalI      CTTGCAAAGT ACATGGAAGA AATTAACGAA AAAATTAAAG ACATGATAAA GAAAATGCAC
1b_Thai      CTTGCAAAGT ACATGGAAGA AATTAACGAA AAAATTAAAG ACATGATAAA GAAAATGCAC
Conservation ********** ********** ********** ********** ********** **********

             ....|....| ....|....| ....|....| ....|....| ....|....| ....|....|
                  1925       1935       1945       1955       1965       1975
1b_SalI      AAATTAAAGG AAGTCATCGT TTTAAGAGAT AAAGCGAATC AAGATGTCAT CACGATTAAC
1b_Thai      AAATTAAAGG AAGTCATCGT TTTAAGAGAT AAAGCGAATC AAGATGTCAT CACGATTAAC
Conservation ********** ********** ********** ********** ********** **********


             ....|....| ....|....| ....|....| ....|....| ....|....| ....|....|
                  1985       1995       2005       2015       2025       2035
1b_SalI      GAGTTACTGA ATGGAGGGTC GAATGCCAAT TTGGAAGAAT TCATAAGTAA AAAGAACAAA
1b_Thai      GAGTTACTGA ATGGAGGGTC GAATGCCAAT TTGGAAGAAT TCATAAGTAA AAAGAACAAA
Conservation ********** ********** ********** ********** ********** **********

             ....|....| ....|....| ....|....| ....|....| ....|....| ....|....|
                  2045       2055       2065       2075       2085       2095
1b_SalI      GCCAATGATG ATATTAATTC GATATTTAAA ACATTGTACA GCGGAGACGT ATATGATCTT
1b_Thai      GCCAATGATG ATATTAATTC GATATTTAAA ACATTGTACA GCGGAGACGT ATATGATCTT
Conservation ********** ********** ********** ********** ********** **********

             ....|....| ....|....| ....|....| ....|....| ....|....| ....|....|
                  2105       2115       2125       2135       2145       2155
1b_SalI      ATCGAAACGG TGGGCACGTT TGTTCGTGAA AAGAGAAAAA TTGTAGGCGA AACGTTTTCC
1b_Thai      ATCGAAACGG TGGGCACGTT TGTTCGTGAA AAGAGAAAAA TTGTAGGCGA AACGTTTTCC
Conservation ********** ********** ********** ********** ********** **********

             ....|....| ....|....| ....|....| ....|....| ....|....| ....|....|
                  2165       2175       2185       2195       2205       2215
1b_SalI      GCTAAACAAA TTGACCAAGT GGAACAATAT CTCAGCGAAG TAAGAGTGGC TCATGCCAAT
1b_Thai      GCTAAACAAA TTGACCAAGT GGAACAATAT CTCAGCGAAG TAAGAGTGGC TCATGCCAAT
Conservation ********** ********** ********** ********** ********** **********

             ....|....| ....|....| ....|....| ....|....| ....|....| ....|....|
                  2225       2235       2245       2255       2265       2275
1b_SalI      TTAGACACCC TGACTCATGA AAAATTGATA AATGCCTATG ATCAGTTAAG CGCAGAAGAG
1b_Thai      TTAGACACCC TGACTCATGA AAAATTGATA AATGCCTATG ATCAGTTAAG CGCAGAAGAG
Conservation ********** ********** ********** ********** ********** **********

             ....|....| ....|....| ....|....| ....|....| ....|....| ....|....|
                  2285       2295       2305       2315       2325       2335
1b_SalI      AACAATACAG AAAAACTTAA AAATGATCTT GTTAAGAAGA AGAGCGAAGA TCTATACAAA
1b_Thai      AACAATACAG AAAAACTTAA AAATGATCTT GTTAAGAAGA AGAGCGAAGA TCTATACAAA
Conservation ********** ********** ********** ********** ********** **********

             ....|....| ....|....| ....|....| ....|....| ....|....| ....|....|
                  2345       2355       2365       2375       2385       2395
1b_SalI      AAAATGGCAG ACTCTTTGAA AACATTTAAA ACTGAATATG CTGATTTGGT GAGAAATATG
1b_Thai      AAAATGGCAG ACTCTTTGAA AACATTTAAA ACTGAATATG CTGATTTGGT GAGAAATATG
Conservation ********** ********** ********** ********** ********** **********

             ....|....| ....|....| ....|....| ....|....| ....|....| ....|....|
                  2405       2415       2425       2435       2445       2455
1b_SalI      GAGGAATACG AAAGGGAAGA AAAAATATTA GAACAGTGTA TGGGAAAAAC TTTGAAAAAG
1b_Thai      GAGGAATACG AAAGGGAAGA AAAAATATTA GAACAGTGTA TGGGAAAAAC TTTGAAAAAG
Conservation ********** ********** ********** ********** ********** **********
```

```
              ....|....| ....|....| ....|....| ....|....| ....|....| ....|....|
                 2465       2475       2485       2495       2505       2515
1b_SalI       GAGGAAGAAC ACTTCAACAC GTTACAAGTG GATGGAGAAG CTCTGAAAGA CACGTACAAT
1b_Thai       GAGGAAGAAC ACTTCAACAC GTTACAAGTG GATGGAGAAG CTCTGAAAGA CACGTACAAT
Conservation  ********** ********** ********** ********** ********** **********

              ....|....| ....|....| ....|....| ....|....| ....|....| ....|....|
                 2525       2535       2545       2555       2565       2575
1b_SalI       TGCGCAGGAT TCGAAAAACT GAGGGGCACT TTTTCGCAGA GGAAATTCGA ATTGTCACAA
1b_Thai       TGCGCAGGAT TCGAAAAACT GAGGGGCACT TTTTCGCAGA GGAAATTCGA ATTGTCACAA
Conservation  ********** ********** ********** ********** ********** **********

              ....|....| ....|....| ....|....| ....|....| ....|....| ....|....|
                 2585       2595       2605       2615       2625       2635
1b_SalI       AAAATAGACC ACGTTAAGGG TGTGGTGACT AATATGGAGA AACTTTTAAA ATCTTACAGT
1b_Thai       AAAATAGACC ACGTTAAGGG TGTGGTGACT AATATGGAGA AACTTTTAAA ATCTTACAGT
Conservation  ********** ********** ********** ********** ********** **********


              ....|....| ....|....| ....|....| ....|....| ....|....| ....|....|
                 2645       2655       2665       2675       2685       2695
1b_SalI       GATTTGGAGA AGCATTTTCT TTTACTTGAT GATAGCGATT ACGTAAAACG AGTGGAAACA
1b_Thai       GCTTTGGAGA AGCATTTTCT TTTACTTGAT GATAGCGATT ACGTAAAACG AGTGGAAACA
Conservation  *.******** ********** ********** ********** ********** **********

              ....|....| ....|....| ....|....| ....|....| ....|....| ....|....|
                 2705       2715       2725       2735       2745       2755
1b_SalI       TTAAGACAAA CTGTTCAGCG CGAAATGATG AATAAAAAAA TGAGTGTTCA AGAGGACAGC
1b_Thai       TTAAGACAAA CTGTTCAGCG CGAAATGATG AATAAAAAAA TGAGTGTTCA AGAGGACAGC
Conservation  ********** ********** ********** ********** ********** **********

              ....|....| ....|....| ....|....| ....|....| ....|....| ....|....|
                 2765       2775       2785       2795       2805       2815
1b_SalI       TTCAAGAGAA GGACTGAATC TGTACTTAAG ACCATCAAAA TATTCGAAAG TTTAAACAAA
1b_Thai       TTCAAGAGAA GGACTGAATC TGTACTTAAG ACCATCAAAA TATTCGAAAG TTTAAACAAA
Conservation  ********** ********** ********** ********** ********** **********

              ....|....| ....|....| ....|....| ....|....| ....|....| ....|....|
                 2825       2835       2845       2855       2865       2875
1b_SalI       ACCATACATT TTCATAACCA CTTAAACAGG AGCATAAACG AATGTGAAGG CATCAATGTA
1b_Thai       ACCATACATT TTCATAACCA CTTAAACAGG AGCATAAACG AATGTGAAGG CATCAATGTA
Conservation  ********** ********** ********** ********** ********** **********

              ....|....| ....|....| ....|....| ....|....| ....|....| ....|....|
                 2885       2895       2905       2915       2925       2935
1b_SalI       TCGATCAACA GTTTAAAAGG CAAAACAGTC GCGTTAAAGG GCGAACTGCA AAAAGAAATT
1b_Thai       TCGATCAACA GTTTAAAAGG CAAAACAGTC GCGTTAAAGG GCGAACTGCA AAAAGAAATT
Conservation  ********** ********** ********** ********** ********** **********

              ....|....| ....|....| ....|....| ....|....| ....|....| ....|....|
                 2945       2955       2965       2975       2985       2995
1b_SalI       GATGACATTG GAGCAGACCA GCTGATAAGT GAAGCTGTAA AAGTGGCACT ACTAGATAAG
1b_Thai       GATGACATTG GAGCAGACCA GCTGATAAGT GAAGCTGTAA AAGTGGCACT ACTAGATAAG
Conservation  ********** ********** ********** ********** ********** **********

              ....|....| ....|....| ....|....| ....|....| ....|....| ....|....|
                 3005       3015       3025       3035       3045       3055
1b_SalI       TTGAAAGCAG AACTAGGAAG TGTAAACAGC AAATTAGATG AAACTCACAT GGATGATTTG
1b_Thai       TTGAAAGCAG AACTAGGAAG TGTAAACAGC AAATTAGATG AAACTCACAT GGATGATTTG
Conservation  ********** ********** ********** ********** ********** **********

              ....|....| ....|....| ....|....| ....|....| ....|....| ....|....|
```

```
                 3065      3075      3085      3095      3105      3115
1b_SalI      CTCAAAAAAT CAACAGATTT GAATAAATTC TACAGAGATT CAAAAAGCGA TTTGCACGAA
1b_Thai      CTCAAAAAAT CAACAGATTT GAATAAATTC TACAGAGATT CAAAAAGCGA TTTGCACGAA
Conservation ********** ********** ********** ********** ********** **********

             ....|....| ....|....| ....|....| ....|....| ....|....| ....|....|
                 3125      3135      3145      3155      3165      3175
1b_SalI      AAGAGGCAAG AATACCCTGT GCACTATTCT CTAGAAAAGG TAAATGACTG GGAAGAAATT
1b_Thai      AAGAGGCAAG AATACCCTGT GCACTATTCT CTAGAAAAGG TAAATGACTG GGAAGAAATT
Conservation ********** ********** ********** ********** ********** **********

             ....|....| ....|....| ....|....| ....|....| ....|....| ....|....|
                 3185      3195      3205      3215      3225      3235
1b_SalI      AAAACGGAAG TCCACGAATT AAATGCTCAT TATAATGCAT TCAACGACAA TAAGGCTAAA
1b_Thai      AAAACGGAAG TCCACGAATT AAATGCTCAT TATAATGCAT TCAACGACAA TAAGGCTAAA
Conservation ********** ********** ********** ********** ********** **********

             ....|....| ....|....| ....|....| ....|....| ....|....| ....|....|
                 3245      3255      3265      3275      3285      3295
1b_SalI      CTAATTTTAG CCAATTCGCG CATGTACTTG GAATCAATAT ATAACCTTAT AAAAGAGTTG
1b_Thai      CTAATTTTAG CCAATTCGCG CATGTACTTG GAATCAATAT ATAACCTTAT AAAAGAGTTG
Conservation ********** ********** ********** ********** ********** **********


             ....|....| ....|....| ....|....| ....|....| ....|....| ....|....|
                 3305      3315      3325      3335      3345      3355
1b_SalI      GTTCATAACA CAAGGGAGGA GAAAGACAAA ATGGAGAGAT CTCTAAAGGG AATAGAAAAA
1b_Thai      GTTCATAACA CAAGGGAGGA GAAAGACAAA ATGGAGAGAT CTCTAAAGGG AATAGAAAAA
Conservation ********** ********** ********** ********** ********** **********

             ....|....| ....|....| ....|....| ....|....| ....|....| ....|....|
                 3365      3375      3385      3395      3405      3415
1b_SalI      AGCGTAGAAA CTATCGAACT GAACCAGGAT TATAAAAACG CTAAAGTTAC GGACAATGAG
1b_Thai      AGCGTAGAAA CTATCGAACT GAACCAGGAT TATAAAAACG CTAAAGTTAC GGACAATGAG
Conservation ********** ********** ********** ********** ********** **********

             ....|....| ....|....| ....|....| ....|....| ....|....| ....|....|
                 3425      3435      3445      3455      3465      3475
1b_SalI      AATGAAATAA AAAATATAAA TGAAGACACT ATTCCAAAAA TTAAGGAACG AATTGATGAG
1b_Thai      AATGAAATAA AAAATATAAA TGAAGACACT ATTCCAAAAA TTAAGGAACG AATTGATGAG
Conservation ********** ********** ********** ********** ********** **********

             ....|....| ....|....| ....|....| ....|....| ....|....| ....|....|
                 3485      3495      3505      3515      3525      3535
1b_SalI      TATAGCAGAA AACTGACTAA ATTCGAGGAA CAATCGGATA GTTTAAACAA CAAAGACACG
1b_Thai      TGTAGCAGAA AACTGACTAA ATTCGAGGAA CAATCGGATA GTTTAAACAA CAAAGACACG
Conservation *.******** ********** ********** ********** ********** **********

             ....|....| ....|....| ....|....| ....|....| ....|....| ....|....|
                 3545      3555      3565      3575      3585      3595
1b_SalI      AATAAAGGAA GTGACGTTAC TCTCTTGCTT GATGTGATAA GACAAATGAA AGGTACTTAT
1b_Thai      AATAAAGGAA GTGACGTTAC TCTCTTGCTT GATGTGATAA GACAAATGAA AGGTACTTAT
Conservation ********** ********** ********** ********** ********** **********

             ....|....| ....|....| ....|....| ....|....| ....|....| ....|....|
                 3605      3615      3625      3635      3645      3655
1b_SalI      CAAGAATTAA AAAAGATACC TACTGAGATG TTCAAAGAGA GGGACGAAAT AAGGAACACT
1b_Thai      CAAGAATTAA AAAAGATACC TACTGAGATG TTCAAAGAGA GGGACGAAAT AAGGAACACT
Conservation ********** ********** ********** ********** ********** **********

             ....|....| ....|....| ....|....| ....|....| ....|....| ....|....|
                 3665      3675      3685      3695      3705      3715
```

```
1b_SalI      GAAGAAAATG TAAACAAGGT TCAATTCGCA TACGAGAGAA AATTAATTGA GCAATTTTTA
1b_Thai      GAAGAAAATG TAAACAAGGT TCAATTCGCA TACGAGAGAA AATTAATTGA GCAATTTTTA
Conservation ********** ********** ********** ********** ********** **********

             ....|....| ....|....| ....|....| ....|....| ....|....| ....|....|
                 3725       3735       3745       3755       3765       3775
1b_SalI      AAAAGAATTA CTCAGAAGAG GAATGAGGCG GAAGAGCAAA CACAACAGAT CAGCGCAATT
1b_Thai      AAAAGAATTA CTCAGAAGAG GAATGAGGCG GAAGAGCAAA CACAACAGAT CAGCGCAATT
Conservation ********** ********** ********** ********** ********** **********

             ....|....| ....|....| ....|....| ....|....| ....|....| ....|....|
                 3785       3795       3805       3815       3825       3835
1b_SalI      GTTAAACAAA TTGAAGGGAT AAGAAAAGAA ACGGGAGATC CTATCGACAA AGAATTAACC
1b_Thai      GTTAAACAAA TTGAAGGGAT AAGAAAAGAA ACGGGAGATC CTATCGACAA AGAATTAACC
Conservation ********** ********** ********** ********** ********** **********

             ....|....| ....|....| ....|....| ....|....| ....|....| ....|....|
                 3845       3855       3865       3875       3885       3895
1b_SalI      ACCACGAACT GCGAAAAACA TCTTCACGAT GCTGAAGAAA AAGGGGAGGA AATAAAGCAA
1b_Thai      ACCACGAACT GCGAAAAACA TCTTCACGAT GCTGAAGAAA AAGGGGAGGA AATAAAGCAA
Conservation ********** ********** ********** ********** ********** **********

             ....|....| ....|....| ....|....| ....|....| ....|....| ....|....|
                 3905       3915       3925       3935       3945       3955
1b_SalI      ATTGAAGAGT TGTCCACCAA ACTGAGAGGA GAAGCTGCCA CTACTGATGT CATAAGTGAA
1b_Thai      ATTGAAGAGT TGTCCACCAA ACTGAGAGGA GAAGCTGCCA CTACTGATGT CATAAGTGAA
Conservation ********** ********** ********** ********** ********** **********


             ....|....| ....|....| ....|....| ....|....| ....|....| ....|....|
                 3965       3975       3985       3995       4005       4015
1b_SalI      GTCATAAAAA TTAAAGAACA GGTTTACGCA CATTTGGAAA AAGCCACAAG CAATAGTAAC
1b_Thai      GTCATAAAAA TTAAAGAACA GGTTTACGCA CATTTGGAAA AAGCCACAAG CAATAGTAAC
Conservation ********** ********** ********** ********** ********** **********

             ....|....| ....|....| ....|....| ....|....| ....|....| ....|....|
                 4025       4035       4045       4055       4065       4075
1b_SalI      CACGTAAGCG AGGCACTGAA CACAATTAAA GAAATGAAAG AACTAATACT GTCAGAGAGT
1b_Thai      CACGTAAGCG AGGCACTGAA CACAATTAAA GAAATGAAAG AACTAATACT GTCAGAGAGT
Conservation ********** ********** ********** ********** ********** **********

             ....|....| ....|....| ....|....| ....|....| ....|....| ....|....|
                 4085       4095       4105       4115       4125       4135
1b_SalI      TATAACAGTG TTATTAACTT TATCTTAAGG AATGTAAATG ATGCTCATAA ATATGTCGAA
1b_Thai      TATAACAGTG TTATTAACTT TATCTTAAGG AATGTAAATG ATGCTCATAA ATATGTCGAA
Conservation ********** ********** ********** ********** ********** **********

             ....|....| ....|....| ....|....| ....|....| ....|....| ....|....|
                 4145       4155       4165       4175       4185       4195
1b_SalI      TTGGTAAAAA TGGAATTGCT AAAAATTGAA GATGCCACTG ACCATGTGAA GATACGATTT
1b_Thai      TTGGTAAAAA TGGAATTGCT AAAAATTGAA GATGCCACTG ACCATGTGAA GATACGATTT
Conservation ********** ********** ********** ********** ********** **********

             ....|....| ....|....| ....|....| ....|....| ....|....| ....|....|
                 4205       4215       4225       4235       4245       4255
1b_SalI      GAGGAAGCAA AGAAGTTAAA GGAAAAAATC AAGACAGATG TGGATGACGA GGGGGCAGAT
1b_Thai      GAGGAAGCAA AGAAGTTAAA GGAAAAAATC AAGACAGATG TGGATGACGA GGGGGCAGAT
Conservation ********** ********** ********** ********** ********** **********

             ....|....| ....|....| ....|....| ....|....| ....|....| ....|....|
                 4265       4275       4285       4295       4305       4315
1b_SalI      CATATCATCA AAGAAATTGA AGAAATAAGA AAAGTAATTT TGAATGAAAT AAAAGACACA
```

```
1b_Thai      CATATCATCA AAGAAATTGA AGAAATAAGA AAAGTAATTT TGAATGAAAT AAAAGACACA
Conservation ********** ********** ********** ********** ********** **********

             ....|....| ....|....| ....|....| ....|....| ....|....| ....|....|
                4325       4335       4345       4355       4365       4375
1b_SalI      AGCACATTTT TAACAAATGC AGAAAAGGGC AAAGAAAATT GTCTTCTACA TTTCGAGAAT
1b_Thai      AGCACATTTT TAACAAATGC AGAAAAGGGC AAAGAAAATT GTCTTCTACA TTTCGAGAAT
Conservation ********** ********** ********** ********** ********** **********

             ....|....| ....|....| ....|....| ....|....| ....|....| ....|....|
                4385       4395       4405       4415       4425       4435
1b_SalI      GCAAAGGGGG GAAAAGCAAA ATTTGATTAC TTAAAAGAAC ATGGTGACGG TGAGCACAAA
1b_Thai      GCAAAGGGGG GAAAAGCAAA ATTTGATTAC TTAAAAGAAC ATGGTGACGG TGAGCACAAA
Conservation ********** ********** ********** ********** ********** **********

             ....|....| ....|....| ....|....| ....|....| ....|....| ....|....|
                4445       4455       4465       4475       4485       4495
1b_SalI      AGAATATCAC ACAGTGAGAT AAATATGGTG GAAGAAAATA TGACAAAAGT GAAACAACAC
1b_Thai      AGAATATCAC ACAGTGAGAT AAATATGGTG GAAGAAAATA TGACAAAAGT GAAACAACAC
Conservation ********** ********** ********** ********** ********** **********

             ....|....| ....|....| ....|....| ....|....| ....|....| ....|....|
                4505       4515       4525       4535       4545       4555
1b_SalI      ACGGATGAAG CTGACAAGGA TGTGGAACAA ACGAAAAAAT TTTATTACTC TATTTTGGGA
1b_Thai      ACGGATGAAG CTGACAAGGA TGTGGAACAA ACGAAAAAAT TTTATTACTC TATTTTGGGG
Conservation ********** ********** ********** ********** ********** *********.

             ....|....| ....|....| ....|....| ....|....| ....|....| ....|....|
                4565       4575       4585       4595       4605       4615
1b_SalI      TATGAAAAAC TAATCAATGA TCTGCTAAAC GAATCGCTAC TTAGAAAAGT CAAATTAAAA
1b_Thai      TATGAAAAAC TAATCAATGA TCTGCTAAAC GAATCGCTAC TTAGAAAAGT CAAATTAAAA
Conservation ********** ********** ********** ********** ********** **********


             ....|....| ....|....| ....|....| ....|....| ....|....| ....|....|
                4625       4635       4645       4655       4665       4675
1b_SalI      TGCGAGAAAT TGAGAACAGA AGTAGACGGA ATAATGGACG AAATGGAACG TGTAGATGTT
1b_Thai      TGCGAGAAAT TGAGAACAGA AGTAGACGGA ATAATGGACG AAATGGAACG TGTAGATGTT
Conservation ********** ********** ********** ********** ********** **********

             ....|....| ....|....| ....|....| ....|....| ....|....| ....|....|
                4685       4695       4705       4715       4725       4735
1b_SalI      AAGGCCAAGG AAGAGTCCAT CGAACAAGAA CAAAAGGTAA AACAAATGAA GGAGCAATCC
1b_Thai      AAGGCCAAGG AAGAGTCCAT CGAACAAGAA CAAAAGGTAA AACAAATGAA GGAGCAATCC
Conservation ********** ********** ********** ********** ********** **********

             ....|....| ....|....| ....|....| ....|....| ....|....| ....|....|
                4745       4755       4765       4775       4785       4795
1b_SalI      ATTGTTGAGG GTGATCAGCC TAAAGAACTT AACGAAGAGT CTATGAACGC ATTGCTACAA
1b_Thai      ATTGTTGAGG GTGATCAGCC TAAAGAACTT AACGAAGAGT CTATGAACGC ATTGCTACAA
Conservation ********** ********** ********** ********** ********** **********

             ....|....| ....|....| ....|....| ....|....| ....|....| ....|....|
                4805       4815       4825       4835       4845       4855
1b_SalI      ATAAAAAATT ACAGACAGAA ATTGGACGAC GTTATGTTAA GTATAAAAAG TGTGGAAGAG
1b_Thai      ATAAAAAATT ACAGACAGAA ATTGGACGAC GTTATGTTAA GTATAAAAAG TGTGGAAGAG
Conservation ********** ********** ********** ********** ********** **********

             ....|....| ....|....| ....|....| ....|....| ....|....| ....|....|
                4865       4875       4885       4895       4905       4915
1b_SalI      AATATGGAGC AGTTTTTAAG GAATGCTGGC AGTTACGTGA AAACAACGCT AGACATCTCT
1b_Thai      AATATGGAGC AGTTTTTAAG GAATGCTGGC AGTTACGTGA AAACAACGCT AGACATCTCT
```

```
Conservation ********** ********** ********** ********** ********** **********

             ....|....| ....|....| ....|....| ....|....| ....|....| ....|....|
                 4925       4935       4945       4955       4965       4975
1b_SalI      CAACTGCGAA ATGAAAATTC GTTCGATAAT TTGACAGCGG CGGAGGAAAA CTACAAAATA
1b_Thai      CAACTGCGAA ATGAAAATTC GTTCGATAAT TTGACAGCGG CGGAGGAAAA CTACAAAATA
Conservation ********** ********** ********** ********** ********** **********

             ....|....| ....|....| ....|....| ....|....| ....|....| ....|....|
                 4985       4995       5005       5015       5025       5035
1b_SalI      GCATTGAGAA ATGTTCAGAA TGAAAAACAG CGTATGATAA GTGAGGAAAA AAGGCTAGCT
1b_Thai      GCATTGAGAA ATGTTCAGAA TGAAAAACAG CGTATGATAA GTGAGGAAAA AAGGCTAGCT
Conservation ********** ********** ********** ********** ********** **********

             ....|....| ....|....| ....|....| ....|....| ....|....| ....|....|
                 5045       5055       5065       5075       5085       5095
1b_SalI      GAAATGTATA GGGATATCAT TAGTGTAGGA AAAGAATTGG AAGAACAAAA AAAAAGATAC
1b_Thai      GAAATGTATA GGGATATCAT TAGTGTAGGA AAAGAATTGG AAGAACAAAA AAAAAGATAC
Conservation ********** ********** ********** ********** ********** **********

             ....|....| ....|....| ....|....| ....|....| ....|....| ....|....|
                 5105       5115       5125       5135       5145       5155
1b_SalI      GAAGTGGGAT TATTGAAAGG GATAAAAGAG AATGCTGACA GAAGATTGAC CGCATTGGAA
1b_Thai      GAAGTGGGAT TATTGAAAGG GATAAAAGAG AATGCTGACA GAAGATTGAC CGCATTGGAA
Conservation ********** ********** ********** ********** ********** **********

             ....|....| ....|....| ....|....| ....|....| ....|....| ....|....|
                 5165       5175       5185       5195       5205       5215
1b_SalI      TTAACCAAAA AGGAAGTTAA CTCTTTGGTG GATCCGTCCA AATCCATATT TTTTAAATTC
1b_Thai      TTAACCAAAA AGGAAGTTAA CTCTTTGGTG GATCCGTCCA AATCCATATT TTTTAAATTC
Conservation ********** ********** ********** ********** ********** **********

             ....|....| ....|....| ....|....| ....|....| ....|....| ....|....|
                 5225       5235       5245       5255       5265       5275
1b_SalI      AAATTGGACA ACTTAGACGA AACAGATATA ATGAAATATC TATATCGCTA TGCTGATCGA
1b_Thai      AAATTGGACA ACTTAGACGA AACAGATATA ATGAAATATC TATATCGCTA TGCTGATCGA
Conservation ********** ********** ********** ********** ********** **********


             ....|....| ....|....| ....|....| ....|....| ....|....| ....|....|
                 5285       5295       5305       5315       5325       5335
1b_SalI      ATTTACCATA TTTTTGACGA ATTTGTGAAA TTGTACAAAA TGATAGAAGG ATATTTATCC
1b_Thai      ATTTACCATA TTTTTGACGA ATTTGTGAAA TTGTACAAAA TGATAGAAGG ATATTTATCC
Conservation ********** ********** ********** ********** ********** **********

             ....|....| ....|....| ....|....| ....|....| ....|....| ....|....|
                 5345       5355       5365       5375       5385       5395
1b_SalI      CAGAATTCGG ACCCCTCTGT AACATTTAAT GAAGTCAAAA TGGTGAGGGA GAAAGCGCAA
1b_Thai      CAGAATTCGG ACCCCTCTGT AACATTTAAT GAAGTCAAAA TGGTGAGGGA GAAAGCGCAA
Conservation ********** ********** ********** ********** ********** **********

             ....|....| ....|....| ....|....| ....|....| ....|....| ....|....|
                 5405       5415       5425       5435       5445       5455
1b_SalI      ATGGAAGAAA TGAGCCTTGG AGAAAAGGAA GAAGAGTCAA AAGAAGTACT GAGAGACATG
1b_Thai      ATGGAAGAAA TGAGCCTTGG AGAAAAGGAA GAAGAGTCAA AAGAAGTACT GAGAGACATG
Conservation ********** ********** ********** ********** ********** **********

             ....|....| ....|....| ....|....| ....|....| ....|....| ....|....|
                 5465       5475       5485       5495       5505       5515
1b_SalI      AAAAAAAAGG AGTCCATAAG GTTGTTAAAC GAAATGATGG AAATGCTGAA CAGTGCAAAG
1b_Thai      AAAAAAAAGG AGTCCATAAG GTTGTTAAAC GAAATGATGG AAATGCTGAA CAGTGCAAAG
Conservation ********** ********** ********** ********** ********** **********
```

```
              ....|....| ....|....| ....|....| ....|....| ....|....| ....|....|
                   5525       5535       5545       5555       5565       5575
1b_SalI       GAAAGAGTTA CGGAAGATCA TGCAAAAGTC AGCAACTATG TGGAGAATGT TAAAAAAGAC
1b_Thai       GAAAGAGTTA CGGAAGATCA TGCAAAAGTC AGCAACTATG TGGAGAATGT TAAAAAAGAC
Conservation  ********** ********** ********** ********** ********** **********

              ....|....| ....|....| ....|....| ....|....| ....|....| ....|....|
                   5585       5595       5605       5615       5625       5635
1b_SalI       GTCACCGATT TAAAGGTACT GGACGATGTA AATAACGGCG CCAATGTTTT TAATAAGGCA
1b_Thai       GTCACCGATT TAAAGGTACT GGACGATGTA AATAACGGCG CCAATGTTTT TAATAAGGCA
Conservation  ********** ********** ********** ********** ********** **********

              ....|....| ....|....| ....|....| ....|....| ....|....| ....|....|
                   5645       5655       5665       5675       5685       5695
1b_SalI       ATGAATAGTG TTAACGAAAT TAAAGACGCA AAGTATATGA ACCATAGGAA GGAAGCGGAA
1b_Thai       ATGAATAGTG TTAACGAAAT TAAAGACGCA AAGTATATGA ACCATAGGAA GGAAGCGGAA
Conservation  ********** ********** ********** ********** ********** **********

              ....|....| ....|....| ....|....| ....|....| ....|....| ....|....|
                   5705       5715       5725       5735       5745       5755
1b_SalI       AATATATACG AAAATATGAT ACAACTGGCG AACTATTTCT TAGATGACGA TGTTAGGATG
1b_Thai       AATATATACG AAAATATGAT ACAACTGGCG AACTATTTCT TAGATGACGA TGTTAGGATG
Conservation  ********** ********** ********** ********** ********** **********

              ....|....| ....|....| ....|....| ....|....| ....|....| ....|....|
                   5765       5775       5785       5795       5805       5815
1b_SalI       GAGTCCACGG GAAAGTTAAG CGAAGCGGCA TTTGCAAAGG CAGAACCGGG TATAGTGTCA
1b_Thai       GAGTCCACGG GAAAGTTAAG CGAAGCGGCA TTTGCAAAGG CAGAACCGGG TATAGTGTCA
Conservation  ********** ********** ********** ********** ********** **********

              ....|....| ....|....| ....|....| ....|....| ....|....| ....|....|
                   5825       5835       5845       5855       5865       5875
1b_SalI       GACATTTTTG GCAAAATAAA GGAGGCGAAG GAAATTGTAG AAAAAATAGA AGAAGAGTCA
1b_Thai       GACATTTTTG GCAAAATAAA GGAGGCGAAG GAAATTGTAG AAAAAATAGA AGAAGAGTCA
Conservation  ********** ********** ********** ********** ********** **********

              ....|....| ....|....| ....|....| ....|....| ....|....| ....|....|
                   5885       5895       5905       5915       5925       5935
1b_SalI       ATAGGTATAC AAAATAAACA AATAGAAGGG GAAAGGCTCT CCACTGAGGC GAACCACATT
1b_Thai       ATAGGTATAC AAAATAAACA AATAGAAGGG GAAAGGCTCT CCACTGAGGC GAACCACATT
Conservation  ********** ********** ********** ********** ********** **********


              ....|....| ....|....| ....|....| ....|....| ....|....| ....|....|
                   5945       5955       5965       5975       5985       5995
1b_SalI       TACAGCGTGG CAAAGCTGAA AAATGAATTT AAAAACAAAA AGAATGAAGC GAAACTTAAG
1b_Thai       TACAGCGTGG CAAAGCTGAA AAATGAATTT AAAAACAAAA AGAATGAAGC GAAACTTAAG
Conservation  ********** ********** ********** ********** ********** **********

              ....|....| ....|....| ....|....| ....|....| ....|....| ....|....|
                   6005       6015       6025       6035       6045       6055
1b_SalI       GTCATATTCG TGCTAGCCGA AATAGAGGAA GCATTACATA AATTAAAAAG TGTGAATAAA
1b_Thai       GTCATATTCG TGCTAGCCGA AATAGAGGAA GCATTACATA AATTAAAAAG TGTGAATAAA
Conservation  ********** ********** ********** ********** ********** **********

              ....|....| ....|....| ....|....| ....|....| ....|....| ....|....|
                   6065       6075       6085       6095       6105       6115
1b_SalI       GTAAAATGCC ATTATGATAA TTATGATAAT TATAATGATA TACTAGAAAA TAACGAAGAA
1b_Thai       GTAAAATGCC ATTATGATAA TTATGATAAT TATAATGATA TACTAGAAAA TAACGAAGAA
Conservation  ********** ********** ********** ********** ********** **********
```

```
             ....|....| ....|....| ....|....| ....|....| ....|....| ....|....|
                 6125       6135       6145       6155       6165       6175
1b_SalI      CATGAGAATT TCAAGCAAAT TTCTTCTACC TACAAGTTGA AGAAGGCCCA AATAGCGAAA
1b_Thai      CATGAGAATT TCAAGCAAAT TTCTTCTACC TACAAGTTGA AGAAGGCCCA AATAGCGAAA
Conservation ********** ********** ********** ********** ********** **********

             ....|....| ....|....| ....|....| ....|....| ....|....| ....|....|
                 6185       6195       6205       6215       6225       6235
1b_SalI      GAAGCAGATA TAAATGAAAT GAAAAATGAT GCAAATATGT ACAAAGACAA GTTAGCCAGT
1b_Thai      GAAGCAGATA TAAATGAAAT GAAAAATGAT GCAAATATGT ACAAAGACAA GTTAGCCAGT
Conservation ********** ********** ********** ********** ********** **********

             ....|....| ....|....| ....|....| ....|....| ....|....| ....|....|
                 6245       6255       6265       6275       6285       6295
1b_SalI      TTGGATAAAA ACGGGGAGAC TTTTAACGAT GGAAGCGATG AAATTTCTAC TGCACAGAAG
1b_Thai      TTGGATAAAA ACGGGGAGAC TTTTAACGAT GGAAGCGATG AAATTTCTAC TGCACAGAAG
Conservation ********** ********** ********** ********** ********** **********

             ....|....| ....|....| ....|....| ....|....| ....|....| ....|....|
                 6305       6315       6325       6335       6345       6355
1b_SalI      TACAAAACTG ACGTTGAAGG TATTATAGAC AAACTAAACG TTATAGATGA AACCATTAAT
1b_Thai      TACAAAACTG ACGTTGAAGG TATTATAGAC AAACTAAACG TTATAGATGA AACCATTAAT
Conservation ********** ********** ********** ********** ********** **********

             ....|....| ....|....| ....|....| ....|....| ....|....| ....|....|
                 6365       6375       6385       6395       6405       6415
1b_SalI      GGAATTAATT CCACGCTAGA CGAATTGCTA GAGTTAGGAA ATAATTGTCA GCTGCATCGG
1b_Thai      GGAATTAATT CCACGCTAGA CGAATTGCTA GAGTTAGGAA ATAATTGTCA GCTGCATCGG
Conservation ********** ********** ********** ********** ********** **********

             ....|....| ....|....| ....|....| ....|....| ....|....| ....|....|
                 6425       6435       6445       6455       6465       6475
1b_SalI      ACCTTTTTAA TCAGCAGTTC TTTGAATAAT AAAATTGCAA AATTCTTAGT AGAGATTAGG
1b_Thai      ACCTTTTTAA TCAGCAGTTC TTTGAATAAT AAAATTGCAA AATTCTTAGT AGAGATTAGG
Conservation ********** ********** ********** ********** ********** **********

             ....|....| ....|....| ....|....| ....|....| ....|....| ....|....|
                 6485       6495       6505       6515       6525       6535
1b_SalI      GAACAAAAAG AGAACACAAA AAAATGCTTC CAATATGTAA AAAGAAATCA CCAACATTTG
1b_Thai      GAACAAAAAG AGAACACAAA AAAATGCTTC CAATATGTAA AAAGAAATCA CCAACATTTG
Conservation ********** ********** ********** ********** ********** **********

             ....|....| ....|....| ....|....| ....|....| ....|....| ....|....|
                 6545       6555       6565       6575       6585       6595
1b_SalI      GCTAATTTCG TTTCTGAGTT ACACAAAACT CAGGGTGGCA TATTTGAGAA TGTTAACCTG
1b_Thai      GCTAATTTCG TTTCTGAGTT ACACAAAACT CAGGGTGGCA TATTTGAGAA TGTTAACCTG
Conservation ********** ********** ********** ********** ********** **********


             ....|....| ....|....| ....|....| ....|....| ....|....| ....|....|
                 6605       6615       6625       6635       6645       6655
1b_SalI      GTGGATAACA CACCGGATGC AGATAAGTAT TACCATGAAT TTATGGAAAT AGAGCAGGAG
1b_Thai      GTGGATAACA CACCGGATGC AGATAAGTAT TACCATGAAT TTATGGAAAT AGAGCAGGAG
Conservation ********** ********** ********** ********** ********** **********

             ....|....| ....|....| ....|....| ....|....| ....|....| ....|....|
                 6665       6675       6685       6695       6705       6715
1b_SalI      GCAACGAAAA TCGTGAAGGA TATAAAAAAG GAAATATACC ACTTAAACGA TGATGTAGAC
1b_Thai      GCAACGAAAA TCGTGAAGGA TATAAAAAAG GAAATATACC ACTTAAACGA TGATGTAGAC
Conservation ********** ********** ********** ********** ********** **********

             ....|....| ....|....| ....|....| ....|....| ....|....| ....|....|
```

```
                  6725      6735      6745      6755      6765      6775
1b_SalI      GAACCTGTTT TGGAGAAACG GATAAAAGAC GTGATCAACA CGTATAACAA ATTGAAGACG
1b_Thai      GAACCTGTTT TGGAGAAACG GATAAAAGAC GTGATCAACA CGTATAACAA ATTGAAGACG
Conservation ********** ********** ********** ********** ********** **********

             ....|....| ....|....| ....|....| ....|....| ....|....| ....|....|
                  6785      6795      6805      6815      6825      6835
1b_SalI      AAAAAGGTCC AAATGGACCA GAGTTATAAA AATATGTATA TCACTAAATT GAGGGAAGTC
1b_Thai      AAAAAGGTCC AAATGGACCA GAGTTATAAA AATATGTATA TCACTAAATT GAGGGAAGTC
Conservation ********** ********** ********** ********** ********** **********

             ....|....| ....|....| ....|....| ....|....| ....|....| ....|....|
                  6845      6855      6865      6875      6885      6895
1b_SalI      GAGGGAAGCC ACGATCTTTT CAACCAGGTA GCACAATTGA TTAGAGGCGA AACGGATAAA
1b_Thai      GAGGGAAGCC ACGATCTTTT CAACCAGGTA GCACAATTGA TTAGAGGCGA AACGGATAAA
Conservation ********** ********** ********** ********** ********** **********

             ....|....| ....|....| ....|....| ....|....| ....|....| ....|....|
                  6905      6915      6925      6935      6945      6955
1b_SalI      AAAGGGAAAG CCCTGTCAGA GAGAGAAAAT AACTTACACA GTATTTACAA TTTTGTAAAA
1b_Thai      AAAGGGAAAG CCCTGTCAGA GAGAGAAAAT AACTTACACA GTATTTACAA TTTTGTAAAA
Conservation ********** ********** ********** ********** ********** **********

             ....|....| ....|....| ....|....| ....|....| ....|....| ....|....|
                  6965      6975      6985      6995      7005      7015
1b_SalI      TTGCACGAAA CTGAGCTGCA TAATCTTTAC GCCAAATACA CACCGGAGTA TATGGAAAAA
1b_Thai      TTGCACGAAA CTGAGCTGCA TAATCTTTAC GCCAAATACA CACCGGAGTA TATGGAAAAA
Conservation ********** ********** ********** ********** ********** **********

             ....|....| ....|....| ....|....| ....|....| ....|....| ....|....|
                  7025      7035      7045      7055      7065      7075
1b_SalI      ATAAATAAAA TATTCGACGA CATCAATGCT AGGATGATTG CGGTTGACCT TAATGATGAT
1b_Thai      ATAAATAAAA TATTCGACGA CATCAATGCT AGGATGATTG CGGTTGACCT TAATGATGAT
Conservation ********** ********** ********** ********** ********** **********

             ....|....| ....|....| ....|....| ....|....| ....|....| ....|....|
                  7085      7095      7105      7115      7125      7135
1b_SalI      CACAGCAGTG AATATAGCGA CGTGAAAAGG CATGAACACG AGGCCATGCT TTTAATGGAC
1b_Thai      CACAGCAGTG AATATAGCGA CGTGAAAAGG CATGAACACG AGGCCATGCT TTTAATGGAC
Conservation ********** ********** ********** ********** ********** **********

             ....|....| ....|....| ....|....| ....|....| ....|....| ....|....|
                  7145      7155      7165      7175      7185      7195
1b_SalI      GCAACAAATA ATTTGTCGAA GGAAGTAGAA ATGATGCAGA ACGAAAGTGG CGGCAAAAAC
1b_Thai      GCAACAAATA ATTTGTCGAA GGAAGTAGAA ATGATGCAGA ACGAAAGTGG CGGCAAAAAC
Conservation ********** ********** ********** ********** ********** **********

             ....|....| ....|....| ....|....| ....|....| ....|....| ....|....|
                  7205      7215      7225      7235      7245      7255
1b_SalI      GATGGCATAA ATGGAGGAAA AAGCCAACTC GTGGAAGATT ACACAAATAC GATGAGCGAA
1b_Thai      GATGGCATAA ATGGAGGAAA AAGCCAACTC GTGGAAGATT ACACAAATAC GATGAGCGAA
Conservation ********** ********** ********** ********** ********** **********


             ....|....| ....|....| ....|....| ....|....| ....|....| ....|....|
                  7265      7275      7285      7295      7305      7315
1b_SalI      TTCACGGAGC AGGCTAAGAC AGTTGCGAAA AAGATACACG ACTCAAAAGG GGACTACGCA
1b_Thai      TTCACGGAGC AGGCTAAGAC AGTTGCGAAA AAGATACACG ACTCAAAAGG GGACTACGCA
Conservation ********** ********** ********** ********** ********** **********

             ....|....| ....|....| ....|....| ....|....| ....|....| ....|....|
                  7325      7335      7345      7355      7365      7375
```

```
1b_SalI      AATATGTTTG ATCATATAAG GGAGAATGAA GCCATGCTGG AAAGAATTGA CCTGAAGAAG
1b_Thai      AATATGTTTG ATCATATAAG GGAGAATGAA GCCATGCTGG AAAGAATTGA CCTGAAGAAG
Conservation ********** ********** ********** ********** ********** **********

             ....|....| ....|....| ....|....| ....|....| ....|....| ....|....|
                 7385       7395       7405       7415       7425       7435
1b_SalI      AAGGACATTA AAGAAATACT TGCGCATTTA AACAGAATGA AGGAATATTT ATTAAAAAAA
1b_Thai      AAGGACATTA AAGAAATACT TGCGCATTTA AACAGAATGA AGGAATATTT ATTAAAAAAA
Conservation ********** ********** ********** ********** ********** **********

             ....|....| ....|....| ....|....| ....|....| ....|....| ....|....|
                 7445       7455       7465       7475       7485       7495
1b_SalI      TTATCTGAAG AGGAGAAATT ACATCATATG AGAGAAAAAT TAGAAGAAGT TAACACGAGT
1b_Thai      TTATCTGAAG AGGAGAAATT ACATCATATG AGAGAAAAAT TAGAAGAAGT TAACACGAGT
Conservation ********** ********** ********** ********** ********** **********

             ....|....| ....|....| ....|....| ....|....| ....|....| ....|....|
                 7505       7515       7525       7535       7545       7555
1b_SalI      ACTGACGAAA TTGTAAAAAA ATTCAGGACA TATGATCAAA TGGTAGATAT ATCCCAAAAT
1b_Thai      ACTGACGAAA TTGTAAAAAA ATTCAGGACA TATGATCAAA TGGTAGATAT ATCCCAAAAT
Conservation ********** ********** ********** ********** ********** **********

             ....|....| ....|....| ....|....| ....|....| ....|....| ....|....|
                 7565       7575       7585       7595       7605       7615
1b_SalI      ATTGACATCA AAAATGTGCA GTCAAAAAGG TACGACTCTG TCGATGAGAT AGATAAAGAA
1b_Thai      ATTGACATCA AAAATGTGCA GTCAAAAAGG TACGACTCTG TCGATGAGAT AGATAAAGAA
Conservation ********** ********** ********** ********** ********** **********

             ....|....| ....|....| ....|....| ....|....| ....|....| ....|....|
                 7625       7635       7645       7655       7665       7675
1b_SalI      ATGAGTTACA TAAAAACTCA CAACAAAGAT TTAATAGACA GCAAATTTAT AGTGGAAAGG
1b_Thai      ATGAGTTACA TAAAAACTCA CAACAAAGAT TTAATAGACA GCAAATTTAT AGTGGAAAGG
Conservation ********** ********** ********** ********** ********** **********

             ....|....| ....|....| ....|....| ....|....| ....|....| ....|....|
                 7685       7695       7705       7715       7725       7735
1b_SalI      GCACTAGAAA ATGACAAAAG GAAAAAGAGC GAAATGGGTT CAGGGTTTAG GGTTCAGGGT
1b_Thai      GCACTAGAAA ATGACAAAAG GAAAAAGAGC GAAATGGCTC AAATATTTTC TACAATTTCG
Conservation ********** ********** ********** *******.*. .*...***.. ..........

             ....|....| ....|....| ....|....| ....|....| ....|....| ....|....|
                 7745       7755       7765       7775       7785       7795
1b_SalI      TCAGGGTTTA GGGTTCAGGG TTCAGGGTTT AGGGTTCAGG GTTCAGGGTT TAGGTTTAGG
1b_Thai      AGAGATAACT CGAGTATGTA CGAATATGCA AAAAGCTTTT TTGATAGCGT CCTTAAAGAA
Conservation ..**...... .*..*..*.. ...*...... *......... .*........ ..........

             ....|....| ....|....| ....|....| ....|....| ....|....| ....|....|
                 7805       7815       7825       7835       7845       7855
1b_SalI      GTTCAGGGTT TAGGGTTCAG GGTTTAG--- ---------- ---------- ----------
1b_Thai      ATTGAAAAAC TAACTCAAAT GATTAGAAAT ATGGATAAAT TGATAAACGA AAATGAAGCC
Conservation .**.*..... **......*. *.**...

             ....|....| ....|....| ....|....| ....|....| ....|....| ....|....|
                 7865       7875       7885       7895       7905       7915
1b_SalI      ---------- ---------- ---------- ---------- ---------- ----------
1b_Thai      GTAATGGAAA AATTGAAAGA TCAACGAAGG GAGTTACAAA ATGTAGAGAA TGCTTCTACC
Conservation


             ....|....| ....|....| ....|....| ....|....| ....|....| ....|....|
                 7925       7935       7945       7955       7965       7975
1b_SalI      ---------- ---------- ---------- ---------- ---------- ----------
```

```
1b_Thai       GATTTGGGAA AATTAGAAGA AGTAGACAAA ATGGCACAAA CCAAATCGGA AACAGAGCTA
Conservation

              ....|....| ....|....| ....|....| ....|....| ....|....| ....|....|
                 7985       7995       8005       8015       8025       8035
1b_SalI       ---------- ---------- ---------- ---------- ---------- ----------
1b_Thai       TCAGAAAGAA ACGATAGTAG GAATGCAAAG GATGGTGCAA CTTATTCTAC ACTTATGGAT
Conservation

              ....|....| ....|....| ....|....| ....|....| ....|....| ....|....|
                 8045       8055       8065       8075       8085       8095
1b_SalI       ---------- ---------- ---------- ---------- ---------- ----------
1b_Thai       GATAAAGAAA CAGATAGCGT GAACGGAGAA GAGACGAAAC AAGAAAATGT TGTGGTGAAG
Conservation

              ....|....| ....|....| ....|....| ....|....| ....|....| ....|....|
                 8105       8115       8125       8135       8145       8155
1b_SalI       ---------- ---------- ---------- ---------- ---------- ----------
1b_Thai       AAGGGACTAC CCCCTCAAAC AGACATTTAC ACAAGTGGAG TTTTAAAAAA TGACAGAAAC
Conservation

              ....|....| ....|....| ....|....| ....|....| ....|....| ....|....|
                 8165       8175       8185       8195       8205       8215
1b_SalI       ---------- ---------- ---------- ---------- ---------- ----------
1b_Thai       GATCAAAAAT CAGAAAAAAT TGGTGAGAAA AAGTCAAACA AACCAGTAGG CACCGAGGAG
Conservation

              ....|....| ....|....| ....|....| ....|....| ....|....| ....|....|
                 8225       8235       8245       8255       8265       8275
1b_SalI       ---------- ---------- ---------- ---------- ---------- ----------
1b_Thai       AATATACAAC ACAGCAGTTA TTTAAACAAT GATAATAGTA ACAACGATAT TGACGTAGGC
Conservation

              ....|....| ....|....| ....|....| ....|....| ....|....| ....|....|
                 8285       8295       8305       8315       8325       8335
1b_SalI       ---------- ---------- ---------- ---------- ---------- ----------
1b_Thai       ACCTTGTATA CGCTAGGTGG ATATAACGCT CCAAATGATA ATTATAACAC TAATGAATCT
Conservation

              ....|....| ....|....| ....|....| ....|....| ....|....| ....|....|
                 8345       8355       8365       8375       8385       8395
1b_SalI       ---------- ---------- ---------- ---------- ---------- ----------
1b_Thai       GGGGATGATA TAAATGAAGA AGCGAAAAAA AAGAGAAATG CTGTTTTATT CGTCTATGTA
Conservation

              ....|....| ....|....| ....|....| ....|....| ....|....| ....|....|
                 8405       8415       8425       8435       8445       8455
1b_SalI       ---------- ---------- ---------- ---------- ---------- ----------
1b_Thai       GGGGGACTGT TTAGTGCTCT TTTCATTTGT ATTGGTGCTG TCTTCTATTT GTTACATAGA
Conservation

              ....|....| ....|....| ....|....| ....|....| ....|....| ....|....|
                 8465       8475       8485       8495       8505       8515
1b_SalI       ---------- ---------- ---------- ---------- ---------- ----------
1b_Thai       AAAATTGGCA TAGAGGGGGT CGGAAAAAGC GATCACGAAA AAAAACCAAC CATTGAGGAC
Conservation

              ....|....| ....|....| ....|....| ....|....| ....|....| ....|....|
                 8525       8535       8545       8555       8565       8575
1b_SalI       ---------- ---------- ---------- ---------- ---------- ----------
1b_Thai       ACCAAAATTG AGGTATTTGA AGAAACATAT GGTTTAAAAC GTAATGTAAA AGATGAGGTT
Conservation
```

```
              ....|....| ....|....| ....|....| ....|....
                  8585       8595       8605       8615
1b_SalI       ---------- ---------- ---------- ---------
1b_Thai       ATTGATGTAC CCTTTGTCGA TATGGAAGAT AATTTGTAA
Conservation
```

Alignment: Pvrbp2a

```
                ....|....| ....|....| ....|....| ....|....| ....|....| ....|....|
                    5         15         25         35         45         55
2a_SalI         ATGGAAAATA AGGTGCTCTG GGCAGTTTTT TACAATCTAG TGCTGTTCCT TCTGGCATCA
2a_Thai         ATGGAAAATA AGGTGCTCTG GGCAGTTTTT TACAATCTAG TGCTGTTCCT TCTGGCATCA
Conservation    ********** ********** ********** ********** ********** **********

                ....|....| ....|....| ....|....| ....|....| ....|....| ....|....|
                   65         75         85         95        105        115
2a_SalI         AGCAAAGAAA GCAATCGAAT TAAAGCATAT AAACTTAAGA AGGAACCTAA GCTATGGCCA
2a_Thai         AGCAAAGAAA GCAATCGAAT TAAAGCATAT AAACTTAAGA AGGAACCTAA GCTATGGCCA
Conservation    ********** ********** ********** ********** ********** **********

                ....|....| ....|....| ....|....| ....|....| ....|....| ....|....|
                  125        135        145        155        165        175
2a_SalI         CTACAAGATA GCCTTAACGA AAGCGATAAA TTCGAGTATA CCAATGGAGG GAAGGAAAAT
2a_Thai         CTACAAGATA GCCTTAACGA AAGCGATAAA TTCGAGTATA CCAATGGAGG GAAGGAAAAT
Conservation    ********** ********** ********** ********** ********** **********

                ....|....| ....|....| ....|....| ....|....| ....|....| ....|....|
                  185        195        205        215        225        235
2a_SalI         CCCCCAAATT TCTTCAGCTC AAATGTAAAG GCGCATAATA AAAAAGAGGG CAAAAAGTAT
2a_Thai         CCCCCAAATT TCTTCAGCTC AAATGTAAAG GCGCATAATA AAAAAGAGGG CAAAAAGTAT
Conservation    ********** ********** ********** ********** ********** **********

                ....|....| ....|....| ....|....| ....|....| ....|....| ....|....|
                  245        255        265        275        285        295
2a_SalI         GAACAAAATT TGTCTTTACC TGACAACACA TCTTTCGTAA CGGTAAAAAA TTATAACTAC
2a_Thai         GAACAAAATT TGTCTTTACC TGACAACACA TCTTTCGTAA CGGTAAAAAA TTATAACTAC
Conservation    ********** ********** ********** ********** ********** **********

                ....|....| ....|....| ....|....| ....|....| ....|....| ....|....|
                  305        315        325        335        345        355
2a_SalI         ACAAGAACAC CATCACACCA TGCTTACATA AGAAGAGACA ATACACACAC CAACAGTACA
2a_Thai         ACAAGAACAC CATCACACCA TGCTTACATA AGAAGAGACA ATACACACAC CAACAGTACA
Conservation    ********** ********** ********** ********** ********** **********

                ....|....| ....|....| ....|....| ....|....| ....|....| ....|....|
                  365        375        385        395        405        415
2a_SalI         AATAATAATC AAATTAGGAA TGTACCTAAA GAATTAAATC CTCGGGAATT TCTCTTCACA
2a_Thai         AATAATAATC AAATTAGGAA TGTACCTAAA GAATTAAATC CTCGGGAATT TCTCTTCACA
Conservation    ********** ********** ********** ********** ********** **********

                ....|....| ....|....| ....|....| ....|....| ....|....| ....|....|
                  425        435        445        455        465        475
2a_SalI         CCAAAGAATC AAATAAGCGC ATCTCTCATC CAAACTAACG GACCGGTGGC TCCAATGGAT
2a_Thai         CCAAAGAATC AAATAAGCGC ATCTCTCATC CAAACTAACG GACCGGTGGC TCCAATGGAT
Conservation    ********** ********** ********** ********** ********** **********

                ....|....| ....|....| ....|....| ....|....| ....|....| ....|....|
                  485        495        505        515        525        535
2a_SalI         ATACTACGTT ACCTAGATTT CTCAAACAGT TCAGGACAAA TTATTTCTAC AGTGTATCCG
2a_Thai         ATACTACGTT ACCTAGATTT CTCAAACAGT TCAGGACAAA TTATTTCTAC AGTGTATCCG
Conservation    ********** ********** ********** ********** ********** **********

                ....|....| ....|....| ....|....| ....|....| ....|....| ....|....|
                  545        555        565        575        585        595
2a_SalI         TTCTATGTTC AGATGAATTA TTTTGCTGAA ATTAAATATT ACATAACATA TCATTACGAG
2a_Thai         TTCTATGTTC AGATGAGTTA TTTTGCTGAA ATTAAATATT ACATAACATA TCATTACGAG
Conservation    ********** ******.*** ********** ********** ********** **********
```

```
             ....|....| ....|....| ....|....| ....|....| ....|....| ....|....|
                   605        615        625        635        645        655
2a_SalI      GCGAAAAAAA ATTATGATGA AGCATATAAT CAAAGCGTAA ATCCACTTAT GTCAAGTATA
2a_Thai      GCGAAAAAAA ATTATGATGA AGCATATAAT CAAAGCGTAA ATCCACTTAT GTCAAGTATA
Conservation ********** ********** ********** ********** ********** **********

             ....|....| ....|....| ....|....| ....|....| ....|....| ....|....|
                   665        675        685        695        705        715
2a_SalI      CAAAATCAAA TAAATTCTTG TGTACCGAAA AAAGCAGCAT TGGAAAAAAC AATATTCGTA
2a_Thai      CAAAATCAAA TAAATTCTTG TGTACCGAAA AAAGCAGCAT TGGAAAAAAC AATATTCGTA
Conservation ********** ********** ********** ********** ********** **********

             ....|....| ....|....| ....|....| ....|....| ....|....| ....|....|
                   725        735        745        755        765        775
2a_SalI      TTAGAATACC CAGAGAATCA CAATATAAAC CTAAGCAATT ACGAAGCAAA GCATAATGAG
2a_Thai      TTAGAATACC CAGAGAATCA CAATATAAAC CTAAGCAATT ACGAAGCAAA GCATAATGAG
Conservation ********** ********** ********** ********** ********** **********

             ....|....| ....|....| ....|....| ....|....| ....|....| ....|....|
                   785        795        805        815        825        835
2a_SalI      TACAAGCAGC AATTAGATGC ATACAAAAAC TGTGTACAGG CAAATATGGA ATCATACACA
2a_Thai      TACAAGCAGC AATTAGATGC ATACAAAAAC TGTGTACAGG CAAATATGAA ATCATACACA
Conservation ********** ********** ********** ********** ********.* **********

             ....|....| ....|....| ....|....| ....|....| ....|....| ....|....|
                   845        855        865        875        885        895
2a_SalI      GATAGGATGT CAAAATTTAA TGAAAAAATC TATTCAATTT TAAATAGTGT GAAATGCACA
2a_Thai      GATAGGATGT CAAAATTTAA TGAAAAAATC TATTCAATTT TAAACAGTGT GAAATGCACA
Conservation ********** ********** ********** ********** **** ***** **********

             ....|....| ....|....| ....|....| ....|....| ....|....| ....|....|
                   905        915        925        935        945        955
2a_SalI      GATGCATGTG AAACTGATAC GTACGAAATT ATGCTTGAAA TATATGTAGA AAGAGTAAAG
2a_Thai      GATGCATGTG AAACTGATAC GTACGAAATT ATGCTTGAAA TATATGTAGA AAGAGTAAAG
Conservation ********** ********** ********** ********** ********** **********

             ....|....| ....|....| ....|....| ....|....| ....|....| ....|....|
                   965        975        985        995       1005       1015
2a_SalI      GAAGTTAATC ATAATAATTA TGTCAATTAT TTGAGTACTT TGAAAGCATC TCTCCAATTG
2a_Thai      GAAGTTAATC ATAATAATTA TGTCAATTAT TTGAGTACTT TGAAAGCATC TCTCCAATTG
Conservation ********** ********** ********** ********** ********** **********

             ....|....| ....|....| ....|....| ....|....| ....|....| ....|....|
                  1025       1035       1045       1055       1065       1075
2a_SalI      GGGGTTACCC TGATGCTCAA AGTAAAACAA GAAATAGACA ATAATGTCAC CATTAGTGCA
2a_Thai      GGGGTTACCC TGATGCTCAA AGTAAAACAA GAAATAGACA ATAATGTCAC CATTAGTGCA
Conservation ********** ********** ********** ********** ********** **********

             ....|....| ....|....| ....|....| ....|....| ....|....| ....|....|
                  1085       1095       1105       1115       1125       1135
2a_SalI      ATAAATTTTT TACAAGAAGA AATGTTAGAT ATCATCACAA TAGGTGAAGC TCACACAGGT
2a_Thai      ATAAATTTTT TACAAGAAGA AATGTTAGAT ATCATCACAA TAGGTGAAGC TCACACAGGT
Conservation ********** ********** ********** ********** ********** **********

             ....|....| ....|....| ....|....| ....|....| ....|....| ....|....|
                  1145       1155       1165       1175       1185       1195
2a_SalI      AAAATTATAC ATGGTAAGGA AAACGTACTA AAACTTCAAA ATAATAATAT TCCACCACAA
2a_Thai      AAAATTATAC ATGGTAAGGA AAACGTACTA AAACTTCAAA ATAATAATAT TCCACCACAA
Conservation ********** ********** ********** ********** ********** **********
```

```
             ....|....| ....|....| ....|....| ....|....| ....|....| ....|....|
                 1205       1215       1225       1235       1245       1255
2a_SalI      GTCCCTTTAA GTACTCTTAA AAAATTATAT TTTGATTCCG CCAATTTCTA TGCCACTTAT
2a_Thai      GTCCCTTTAA GTACTCTTAA AAAATTATAT TTTGATTCCG CCAATTTCTA TGCCACTTAT
Conservation ********** ********** ********** ********** ********** **********

             ....|....| ....|....| ....|....| ....|....| ....|....| ....|....|
                 1265       1275       1285       1295       1305       1315
2a_SalI      AAGTTTAGCT TAAAAAGAGC CGATACCACG ACAGCTGCGC TAAAGGAAAA AGGAAAATTA
2a_Thai      AAGTTTAGCT TAAAAAGAGC CGATACCACG ACAGCTGCGC TAAAGGAAAA AGGAAAATTA
Conservation ********** ********** ********** ********** ********** **********

             ....|....| ....|....| ....|....| ....|....| ....|....| ....|....|
                 1325       1335       1345       1355       1365       1375
2a_SalI      CTTGCTAACC TTTATAATAA ATTGATAACA TATGTAAGCG AAAAAATAGA TAAAAATTTG
2a_Thai      CTTGCTAACC TTTATAATAA ATTGATAACA TATGTAAGCG AAAAAATAGA TAAAAATTTG
Conservation ********** ********** ********** ********** ********** **********

             ....|....| ....|....| ....|....| ....|....| ....|....| ....|....|
                 1385       1395       1405       1415       1425       1435
2a_SalI      GATTCTTTAT ACTTCATTTC AAAAAGTGAA TCAATGATTT CCGAATTTGA AGATACCTTT
2a_Thai      GATTCTTTAT ACTTCATTTC AAAAAGTGAA TCAATGATTT CCGAATTTGA AGATACCTTT
Conservation ********** ********** ********** ********** ********** **********

             ....|....| ....|....| ....|....| ....|....| ....|....| ....|....|
                 1445       1455       1465       1475       1485       1495
2a_SalI      AAAGCGGGAA CAGAAGCAAG CAAAGAAAAC TCCAGCATAA CAGCAGATTA CGCACGCTAT
2a_Thai      AAAGCGGGAA CAGAAGCAAG CAAAGAAAAC TCCAGCATAA CAGCAGATTA CGCACGCTAT
Conservation ********** ********** ********** ********** ********** **********

             ....|....| ....|....| ....|....| ....|....| ....|....| ....|....|
                 1505       1515       1525       1535       1545       1555
2a_SalI      TCAAATTTGC AAATTATAGA TATCAAATCA AAGTACGATT ACAAAATAAC CGAGTTAAAG
2a_Thai      TCAAATTTGC AAATTATAGA TATCAAATCA AAGTACGATT ACAAAATAAC CGAGTTAAAG
Conservation ********** ********** ********** ********** ********** **********

             ....|....| ....|....| ....|....| ....|....| ....|....| ....|....|
                 1565       1575       1585       1595       1605       1615
2a_SalI      AAATACTTAA ATCAATTAAA GGTACTCATT TCTCCTATGA AAGATTTATA TAATCTAAAT
2a_Thai      AAATACTTAA ATCAATTAAA GGTACTCATT TCTCCTATGA AAGATTTATA TAATCTAAAT
Conservation ********** ********** ********** ********** ********** **********

             ....|....| ....|....| ....|....| ....|....| ....|....| ....|....|
                 1625       1635       1645       1655       1665       1675
2a_SalI      TCTCTACAAA AAGACCAAAG GGACAGTGTA AGAAGTATAC GCTCATCTGA TAGTAGTCCA
2a_Thai      TCTCTACAAA AAGACCAAAG GGACAGTGTA AGAAGTATAC GCTCATCTGA TAGTAGTCCA
Conservation ********** ********** ********** ********** ********** **********

             ....|....| ....|....| ....|....| ....|....| ....|....| ....|....|
                 1685       1695       1705       1715       1725       1735
2a_SalI      CAAGAAAAGG TTAGCGCACT TTTAAACTTC TTATCAACAA TAAAGGAAGA GAATGGCACC
2a_Thai      CAAGAAAAGG TTAGCGCACT TTTAAACTTC TTATCAAAAA TAAAGGAAGA GAATGGCACC
Conservation ********** ********** ********** *******.** ********** **********

             ....|....| ....|....| ....|....| ....|....| ....|....| ....|....|
                 1745       1755       1765       1775       1785       1795
2a_SalI      GTGTTGGAGA ATTTTAAAAA TATAGAAAGA TATTATGAAC AAGGCAAGCC ATTAAAAGGT
2a_Thai      GTGTTGGAGA ATTTTAAAAA TATAGAAAGA TATTATGAAC AAGGCAAGCC ATTAAAAGGT
Conservation ********** ********** ********** ********** ********** **********
```

```
             ....|....| ....|....| ....|....| ....|....| ....|....| ....|....|
                  1805       1815       1825       1835       1845       1855
2a_SalI      ACACTCGAGG GATTAATAAA AATGATCAAA ACCTACGAAC AAGATATAGT AAATTTAATT
2a_Thai      ACACTCGAGG GATTAATAAA AATGATCAAA ACCTACGAAC AAGATATAGT AAATTTAATT
Conservation ********** ********** ********** ********** ********** **********

             ....|....| ....|....| ....|....| ....|....| ....|....| ....|....|
                  1865       1875       1885       1895       1905       1915
2a_SalI      GATAACGAAA AGCACAACAG ATCGTACAAA GATGACATAA AAAACAAAAT AATATATATA
2a_Thai      GATAACGAAA AGCACAACAG ATCGTACAAA GATGACATAA AAAACAAAAT AATATATATA
Conservation ********** ********** ********** ********** ********** **********

             ....|....| ....|....| ....|....| ....|....| ....|....| ....|....|
                  1925       1935       1945       1955       1965       1975
2a_SalI      ACCGAAAAAA CAGATGCTAT AAAAGACATT ATCCCCTTAA ATGAGGAAGT AGACAATATC
2a_Thai      ACCGAAAAAA CAGATGCTAT AAAAGACATT ATCCCCTTAA ATGAGGAAGT AGACAATATC
Conservation ********** ********** ********** ********** ********** **********

             ....|....| ....|....| ....|....| ....|....| ....|....| ....|....|
                  1985       1995       2005       2015       2025       2035
2a_SalI      ATCAAACAAA TTGAGAAACT ATTCAATGAA GACCTATTCC ATGAAGGATT ACCAAATATG
2a_Thai      ATCAAACAAA TTGAGAAACT ATTCAATGAA GACCTATTCC ATGAAGGATT ACCAAATATG
Conservation ********** ********** ********** ********** ********** **********

             ....|....| ....|....| ....|....| ....|....| ....|....| ....|....|
                  2045       2055       2065       2075       2085       2095
2a_SalI      GAAAAACTTA AAAAAGAGAA AGAAAAAATA AAAGCTACTA TAAAATCACT CAACACTAAT
2a_Thai      GAAAAACTTA AAAAAGAGAA AGAAAAAATA AAAGCTACTA TAAAATCGCT CAACACTAAT
Conservation ********** ********** ********** ********** *******.** **********

             ....|....| ....|....| ....|....| ....|....| ....|....| ....|....|
                  2105       2115       2125       2135       2145       2155
2a_SalI      TTCTACGACG GAGACTTAAA AGAATTAGTC GACGATATGT TAAATTTTGT CTACAAACAC
2a_Thai      TTCTACGACG GAGACTTAAA AGAATTAGTC GACGATATGT TAAATTTTGT CTACAAACAC
Conservation ********** ********** ********** ********** ********** **********

             ....|....| ....|....| ....|....| ....|....| ....|....| ....|....|
                  2165       2175       2185       2195       2205       2215
2a_SalI      AGCGATTTCT ACAATCAAGC ATACGCTAAA GGAGATGTTG TAAATCTACA AAAGGAAGCA
2a_Thai      AGCGATTTCT ACGATCAAGC ATACGCTAAA GGAGATGTTG TAAATCTACA AAAGGAAGCA
Conservation ********** **.******* ********** ********** ********** **********

             ....|....| ....|....| ....|....| ....|....| ....|....| ....|....|
                  2225       2235       2245       2255       2265       2275
2a_SalI      GAACAAGAAT ATGAAAAAAT TACAAAACTA AAATCGAATA ACATTCCTCA AATGTTAAAA
2a_Thai      GAACAAGAAT ATGAAAAAAT TACAAAACTA AAATCGAATA ACATTCCTCA AATGTTAAAA
Conservation ********** ********** ********** ********** ********** **********

             ....|....| ....|....| ....|....| ....|....| ....|....| ....|....|
                  2285       2295       2305       2315       2325       2335
2a_SalI      GATTTGAAAT CAGAATCGAA AAATCTTTCA GAGCTTAAAG ACACCATGAT GGATGAAATG
2a_Thai      GATTTGAAAT CAGAATCGAA AAATCTTTCA GAGCTTAAAG ACACCATGAT GGATGAAATG
Conservation ********** ********** ********** ********** ********** **********

             ....|....| ....|....| ....|....| ....|....| ....|....| ....|....|
                  2345       2355       2365       2375       2385       2395
2a_SalI      TTGAACAATG TTCATCAAGA CATATCAAAC GTTTTCGAAC AGATAAGGAA CAAATATAAA
2a_Thai      TTGAACAATG TTCATCAAGA CATATCAAAC GTTTTCGAAC AGATAAGGAA CAAATATAAA
Conservation ********** ********** ********** ********** ********** **********
```

```
             ....|....| ....|....| ....|....| ....|....| ....|....| ....|....|
                 2405       2415       2425       2435       2445       2455
2a_SalI      GACATAACAA CCTCGCTCAG TGCCTACAAA GAGGACAAAC AGAAATTCGA GAAATTTAAA
2a_Thai      GACATAACAA CCTCGCTCAG TGCCTACAAA GAGGACAAAC AGAAATTCGA GAAATTTAAA
Conservation ********** ********** ********** ********** ********** **********

             ....|....| ....|....| ....|....| ....|....| ....|....| ....|....|
                 2465       2475       2485       2495       2505       2515
2a_SalI      GTCGATATGA TACAGACGAA AAACGAACTC CTGGGGAGTC TTCAACAAAA TGATAAAAAA
2a_Thai      GTCGATATGA TACAGACGAA AAACGAACTC CTGGGGAGTC TTCAACAAAA TGATAAAAAA
Conservation ********** ********** ********** ********** ********** **********

             ....|....| ....|....| ....|....| ....|....| ....|....| ....|....|
                 2525       2535       2545       2555       2565       2575
2a_SalI      CTCTCAGAAG CAAATAATAT GTATGAAGAA TTCTTGGCGC ACATAGATAC TACTACCAAT
2a_Thai      CTCTCAGAAG CAAATAATAT GTATGAAGAA TTCTTGGCGC ACATAGATAC TACTACCAAT
Conservation ********** ********** ********** ********** ********** **********

             ....|....| ....|....| ....|....| ....|....| ....|....| ....|....|
                 2585       2595       2605       2615       2625       2635
2a_SalI      AGAGAAAAGC AAATGTATAA CCAAGCCAAC GCGTTAAAGG AAAATTTAGC AAATGTTCAA
2a_Thai      AGAGAAAAGC AAATGTATAA CCAAGCCAAC GCGTTAAAGG AAAATTTAGC AAATGTTCAA
Conservation ********** ********** ********** ********** ********** **********

             ....|....| ....|....| ....|....| ....|....| ....|....| ....|....|
                 2645       2655       2665       2675       2685       2695
2a_SalI      AGTAAAATAT CATCGTACCA GGGAAAAATA GAAAAATTGA AAGCGCATAC TAGGAAAAAT
2a_Thai      AGTAAAATAT CATCGTACCA GGGAAAAATA GAAAAATTGA AAGCGCATAC TAGGAAAAAT
Conservation ********** ********** ********** ********** ********** **********

             ....|....| ....|....| ....|....| ....|....| ....|....| ....|....|
                 2705       2715       2725       2735       2745       2755
2a_SalI      TATGAAAAAA TTAAGACATT ACTGCAAACT TTTGGCAATG AAAATATAAA CGCAAAACTG
2a_Thai      TATGAAAAAA TTAAGACATT ACTGCAAACT TTTGGCAATG AAAATATAAA CGCAAAACTG
Conservation ********** ********** ********** ********** ********** **********

             ....|....| ....|....| ....|....| ....|....| ....|....| ....|....|
                 2765       2775       2785       2795       2805       2815
2a_SalI      GCAGAATACG AAAAAGAATT TAATGAAAAT AAGAGAGTAG TGAATACCAT CATAAACCAA
2a_Thai      GCAGAATACG AAAAAGAATT TAATGAAAAT AAGNAAGTAG TGAATACCAT CATAAACCAA
Conservation ********** ********** ********** *** .***** ********** **********

             ....|....| ....|....| ....|....| ....|....| ....|....| ....|....|
                 2825       2835       2845       2855       2865       2875
2a_SalI      ATTCAAGAAT CAAACAAAAA TATAGAGAAT ATTAAAATAT TAAATAATTC TATCGACCGT
2a_Thai      ATTCAAGAAT CAAACAAAAA TATAGAGAAT ATTAAAATAT TAAATAATTC TATCGACCGT
Conservation ********** ********** ********** ********** ********** **********

             ....|....| ....|....| ....|....| ....|....| ....|....| ....|....|
                 2885       2895       2905       2915       2925       2935
2a_SalI      TCTGAAGTGA ACAAACAGCT GATAGAAGAA TTGGCCACCA ATAAGCAACA ATTAAAGGAA
2a_Thai      TCTGAAGTGA ACAAACAGCT GATAGAAGAA TTGGCCACCA ATAAGCAACA ATTAAAGGAA
Conservation ********** ********** ********** ********** ********** **********

             ....|....| ....|....| ....|....| ....|....| ....|....| ....|....|
                 2945       2955       2965       2975       2985       2995
2a_SalI      AAAATAGATA GTCACATTAA GCAACTAAAC AGTTACAATA TCATTGCAAA AGATGATAAG
2a_Thai      AAAATAGATA GTCACATTAA GCAACTAAAC AGTTACAATA TCATTGCAAA AGATGATAAG
Conservation ********** ********** ********** ********** ********** **********
```

```
             ....|....| ....|....| ....|....| ....|....| ....|....| ....|....|
                  3005       3015       3025       3035       3045       3055
2a_SalI      CTAATTTTTG AGAAAAGGTT AAATGAGGAG AAGGCAAATA TTAACACGCA ACTAAGCGAC
2a_Thai      CTAATTTTTG AGAAAACGTT AAATGAGGAG AAGGCAAATA TTAACACGCA ACTAAGCGAC
Conservation ********** ****** *** ********** ********** ********** **********

             ....|....| ....|....| ....|....| ....|....| ....|....| ....|....|
                  3065       3075       3085       3095       3105       3115
2a_SalI      ACGTCAATAG ATAATTTAAA AGCACAAATA CAAGAAACGC TAGATTATTA CAAAACAGCA
2a_Thai      ACGTCAATAG ATAATTTAAA AGCACAAATA CAAGAAACGC TAGATTATTA CAAAACAGCA
Conservation ********** ********** ********** ********** ********** **********

             ....|....| ....|....| ....|....| ....|....| ....|....| ....|....|
                  3125       3135       3145       3155       3165       3175
2a_SalI      AAGGCAAACA CGAATGATAA AGATGGAACC CATTTAGAAA AATTAGATAA AAAAAAAATG
2a_Thai      AAGGCAAACA CGAATGATAA AGATGGAACC CATTTAGAAA AATTAGATAA AAAAAAAATG
Conservation ********** ********** ********** ********** ********** **********

             ....|....| ....|....| ....|....| ....|....| ....|....| ....|....|
                  3185       3195       3205       3215       3225       3235
2a_SalI      GATTGGAAAG ACAGCAAAGT TAAAATAGAT AAATTAAGCA CCAGCTATCA AGTGCTAGAT
2a_Thai      GATTGGAAAG ACAGCAAAGT TAAAATAGAT NAATTAAGCC CCAGCTATCA AGTGCTAGAT
Conservation ********** ********** **********  ********. ********** **********

             ....|....| ....|....| ....|....| ....|....| ....|....| ....|....|
                  3245       3255       3265       3275       3285       3295
2a_SalI      AAAAAAATTA ATGATTTGAT TAAAAAGCAA CACGACGAAA TTGTAGCTCT CATTGATAAA
2a_Thai      AAAAAAATTA ATGATTTGAT TAAAAAGCAA CCCGACGAAA TTGTAGCTCT CATTGATAAA
Conservation ********** ********** ********** *.******** ********** **********

             ....|....| ....|....| ....|....| ....|....| ....|....| ....|....|
                  3305       3315       3325       3335       3345       3355
2a_SalI      CTTATAACAG AAAAGGGCAA TGAAATAAGG GAGAAAACCG ACGAAAAAAT AAAGCACCTT
2a_Thai      CTTATAACAG AAAAGGGCAA TGAAATAAGG GAGAAAACCG ACGAAAAAAT AAAGCACCTT
Conservation ********** ********** ********** ********** ********** **********

             ....|....| ....|....| ....|....| ....|....| ....|....| ....|....|
                  3365       3375       3385       3395       3405       3415
2a_SalI      AGCGAAATAA AAACAAAATT GTCCTCTTTC ACTGTCTCCG ATAATGTTAA GAATACCCAA
2a_Thai      AGCGAAATAA AAACAAAATT GTCCTCTTTC ACTGTCTCCG ATAATGTTAA GAATACCCAA
Conservation ********** ********** ********** ********** ********** **********

             ....|....| ....|....| ....|....| ....|....| ....|....| ....|....|
                  3425       3435       3445       3455       3465       3475
2a_SalI      AATGTTGCAA TAAAAGGGGA AATAAGTGGA TTCGAAAAAA AAATGGAAGC CCTATTAAGA
2a_Thai      AATGTTGCAA TAAAAGGGGA AATAAGTGGA TTCGAAAAAA AAATGGAAGC CCTATTAAGA
Conservation ********** ********** ********** ********** ********** **********

             ....|....| ....|....| ....|....| ....|....| ....|....| ....|....|
                  3485       3495       3505       3515       3525       3535
2a_SalI      AGAATAGACA ATAATAACGC AAAATTAACT GAACTGAGAG GGAAGGCAGA CCAACATGTA
2a_Thai      AGAATAGACA ATAATAACGC AAAATTAACT GAACTGAGAG GGAAGGCAGA CCAACATGTA
Conservation ********** ********** ********** ********** ********** **********

             ....|....| ....|....| ....|....| ....|....| ....|....| ....|....|
                  3545       3555       3565       3575       3585       3595
2a_SalI      ACCACCCTAC GTGGGGAAAA AAATAAAACA GCTGAATTCA ATGCGAAAAA AGATAGCCTG
2a_Thai      ACCACCCTAC GTGGGGAAAA AAATAAAACA GCTGAATTCA ATGCGAAAAA AGATAGCCTG
Conservation ********** ********** ********** ********** ********** **********
```

```
             ....|....| ....|....| ....|....| ....|....| ....|....| ....|....|
                 3605       3615       3625       3635       3645       3655
2a_SalI      GAAAAAATAC ACCAACAAAT GGAAAATACG CATAAAGAAT TGGAGAGCAT GGAAAATGAA
2a_Thai      GAAAAAATAC ACCAACAAAT GGAAAATACG CATAAAGAAT TGGAGAGCAT GGAAAATGAA
Conservation ********** ********** ********** ********** ********** **********

             ....|....| ....|....| ....|....| ....|....| ....|....| ....|....|
                 3665       3675       3685       3695       3705       3715
2a_SalI      AAAATTCCCA TAAACGACAT TAACCAAATG GAAATAGAAT ATGCACGAAT TTTAATTCAT
2a_Thai      AAAATTCCCA TAAACGACAT TAACCAAATG GAAATAGAAT ATGCACGAAT TTTAATTCAT
Conservation ********** ********** ********** ********** ********** **********

             ....|....| ....|....| ....|....| ....|....| ....|....| ....|....|
                 3725       3735       3745       3755       3765       3775
2a_SalI      CATATGGTTC AACAAATTGG TGAAAAAAAG GAAAGGTCTA AAATCATCTT AGAAGAGATT
2a_Thai      CATATGGTTC AACAAATTGG TGAAAAAAAG GAAAGGTCTA AAATCATCTT AGAAGAGATT
Conservation ********** ********** ********** ********** ********** **********

             ....|....| ....|....| ....|....| ....|....| ....|....| ....|....|
                 3785       3795       3805       3815       3825       3835
2a_SalI      AAAACAACTA TAGCAAGCAT CGATCAAGCA AAGAAAAATA TACCTCCTGA GCAACAAGAT
2a_Thai      AAAACAACTA TAGCAAGCAT CGATCAAGCA AAGAAAAATA TACCTCCTGA GCAACAAGAT
Conservation ********** ********** ********** ********** ********** **********

             ....|....| ....|....| ....|....| ....|....| ....|....| ....|....|
                 3845       3855       3865       3875       3885       3895
2a_SalI      ATCCTTCGCG AATTCGAGTA TAGCGGATAT CGCGACAAAG CTACTGCCAG TAGTACCGAA
2a_Thai      ATCCTTCGCG AATTCGAGTA TAGCGGATAT CGCGACAAAG CTACTGCCAG TAGTACCGAA
Conservation ********** ********** ********** ********** ********** **********

             ....|....| ....|....| ....|....| ....|....| ....|....| ....|....|
                 3905       3915       3925       3935       3945       3955
2a_SalI      ATAGAACAAA TTGATAAAAG TGCAAAAGCA GAGGAGGGTA AAGCCAACAG TAGCACCAAC
2a_Thai      ATAGAACAAA TTGATAAAAG TGCAAAAGCA GAGGAGGGTA AAGCCAACAG TAGCACCAAC
Conservation ********** ********** ********** ********** ********** **********

             ....|....| ....|....| ....|....| ....|....| ....|....| ....|....|
                 3965       3975       3985       3995       4005       4015
2a_SalI      TTAAGCGACA TTGATGTGAT TAAAAAACAG GTACTGTCCT ACTTCAGAAA AGCCACCACT
2a_Thai      TTAAGCGACA TTGATGTGAT TAAAAAACAG GTACTGTCCT ACTTCAGAAA AGCCACCACT
Conservation ********** ********** ********** ********** ********** **********

             ....|....| ....|....| ....|....| ....|....| ....|....| ....|....|
                 4025       4035       4045       4055       4065       4075
2a_SalI      GAAAGTAACA CCATGGAAAA TGCGTTAAGA GAAATTAAAA ATGTGAACAC TTTCTTAACA
2a_Thai      GAAAGTAACA CCATGGAAAA TGCGTTAAGA GAAATTAAAA ATGTGAACAC TTTCTTAACA
Conservation ********** ********** ********** ********** ********** **********

             ....|....| ....|....| ....|....| ....|....| ....|....| ....|....|
                 4085       4095       4105       4115       4125       4135
2a_SalI      TCAAACATTT CAGAAGAAAT TTTAGCTAAC ATACTAAAGA GTGCAGCGAG GACCCAAGAA
2a_Thai      TCAAACATTT CAGAAGAAAT TTTAGCTAAC ATACTAAAGA GTGCAGCGAG GACCCAAGAA
Conservation ********** ********** ********** ********** ********** **********

             ....|....| ....|....| ....|....| ....|....| ....|....| ....|....|
                 4145       4155       4165       4175       4185       4195
2a_SalI      TTAAATCAAC AAGCGGTAGG CGAATTTAAC AAAACAGATC GTCTAATTAA AGAAGTAGAA
2a_Thai      TTAAATCAAC AAGCGGTAGG CGAATTTAAC AAAACAGATC GTCTAATTAA AGAAGTAGAA
Conservation ********** ********** ********** ********** ********** **********
```

```
             ....|....| ....|....| ....|....| ....|....| ....|....| ....|....|
                  4205       4215       4225       4235       4245       4255
2a_SalI      GCGAAGCTAT CCCAGGCAAA CGAACATAAA TCAGCAATTT CAGGAAGTGT AGAATACAAA
2a_Thai      GCGAAGCTAT CCCAGGCAAA CGAACATAAA TCAGCAATTT CAGGAAGTGT AGAATACAAA
Conservation ********** ********** ********** ********** ********** **********

             ....|....| ....|....| ....|....| ....|....| ....|....| ....|....|
                  4265       4275       4285       4295       4305       4315
2a_SalI      CAAATCGAAC AGAAAATAAA CCTAATTAAA CAAATTCAGA AAGAAATTAC GGCGGGGAAA
2a_Thai      CAAATCGAAC AGAAAATAAA CCTAATTAAA CAAATTCAGA AAGAAATTAC GGCGGGGAAA
Conservation ********** ********** ********** ********** ********** **********

             ....|....| ....|....| ....|....| ....|....| ....|....| ....|....|
                  4325       4335       4345       4355       4365       4375
2a_SalI      GAAGAAATAA ACAACTGTTT AAGTAATACC AAAGAATATA AAGAAAAATG CGAGTCTGAA
2a_Thai      GAAGAAATAA ACAACTGTTT AAGTAATACC AAAGAATATA AAGAAAAATG CGAGTCTGAA
Conservation ********** ********** ********** ********** ********** **********

             ....|....| ....|....| ....|....| ....|....| ....|....| ....|....|
                  4385       4395       4405       4415       4425       4435
2a_SalI      GTGAATTCAG TAAATAGAGG AAAAGCTAAA GTAGATTTTC TGCAAAAAAG GGAAGCGCTA
2a_Thai      GTGAATTCAG TAAATAGAGG AAAAGCTAAA GTAGATTTTC TGCAAAAAAG GGAAGCGCTA
Conservation ********** ********** ********** ********** ********** **********

             ....|....| ....|....| ....|....| ....|....| ....|....| ....|....|
                  4445       4455       4465       4475       4485       4495
2a_SalI      GAAAAAAAAA TGAGTCAAGA AAATTTGGGT AAAATTACAG ATAGCATTGA TCAATGCAAA
2a_Thai      GAAAAAAAAA TGAGTCAAGA AAATTTGGGT AAAATTACAG ATAGCATTGA TCAATGCAAA
Conservation ********** ********** ********** ********** ********** **********

             ....|....| ....|....| ....|....| ....|....| ....|....| ....|....|
                  4505       4515       4525       4535       4545       4555
2a_SalI      AAAGATTTAG CAGACATTAC TAGTTTAGAA GTAAAGGTTA AAGCTAATTA CGATTCAATC
2a_Thai      AAAGATTTAG CAGACATTAC TAGTTTAGAA GTAAAGGTTA AAGCTAATTA CGATTCAATC
Conservation ********** ********** ********** ********** ********** **********

             ....|....| ....|....| ....|....| ....|....| ....|....| ....|....|
                  4565       4575       4585       4595       4605       4615
2a_SalI      ATTAAGTATG AAGAATCCAT TAATACCATT TTAAATTATT CCTCCATTTT AGAATACAAA
2a_Thai      ATTAAGTATG AAGAATCCAT TAATACCATT TTAAATTATT CCTCCATTTT AGAATACAAA
Conservation ********** ********** ********** ********** ********** **********

             ....|....| ....|....| ....|....| ....|....| ....|....| ....|....|
                  4625       4635       4645       4655       4665       4675
2a_SalI      ACCAAATTGG AAATACGCAA AAAAGAAAAA ACAGACCTCA TGACTTATAT TAACACAGAA
2a_Thai      ACCAAATTGG AAATACGCAA AAAAGAAAAA ACAGACCTCA TGACTTATAT TAACACAGAA
Conservation ********** ********** ********** ********** ********** **********

             ....|....| ....|....| ....|....| ....|....| ....|....| ....|....|
                  4685       4695       4705       4715       4725       4735
2a_SalI      AATTCTGCCA TTCAGGAGAA GCTGCTAAAC TTGCAAAAAA AATTGAACCA ATTGAATGAA
2a_Thai      AATTCTGCCA TTCAGGAGAA GCTGCTAAAC TTGCAAAAAA AATTGAACCA ATTGAATGAA
Conservation ********** ********** ********** ********** ********** **********

             ....|....| ....|....| ....|....| ....|....| ....|....| ....|....|
                  4745       4755       4765       4775       4785       4795
2a_SalI      AACACGGACT ATACTAAAGT GGGAAACGAT CTCAACAACG CAAAGTCTAC AAAAGCAAAT
2a_Thai      AACACGGACT ATACTAAAGT GGGAAACGAT CTCAACAACG CAAAGTCTAC AAAAGCAAAT
Conservation ********** ********** ********** ********** ********** **********
```

```
             ....|....| ....|....| ....|....| ....|....| ....|....| ....|....|
                  4805       4815       4825       4835       4845       4855
2a_SalI      GTAACTATCC AGTATAACTT AGGACGAGTC AAACATCAAC TGGAAAATTT AAGTGTCATC
2a_Thai      GTAATTATCC AGTATAACTT AGGACGAGTC AAACATCAAC TGGAAAATGT AAGTGTCATC
Conservation **** ***** ********** ********** ********** ******** * **********

             ....|....| ....|....| ....|....| ....|....| ....|....| ....|....|
                  4865       4875       4885       4895       4905       4915
2a_SalI      AAACAGGAGC TAGAAAAAGT CCTAAGTGCA GCTACCGATT TAGAGAGGGA CATCTCAAAA
2a_Thai      AAACAGGAGC TAGAAAAAGT CCTAAGTGCA GCTACCGATT TAGAGAGGGA CATCTCAAAA
Conservation ********** ********** ********** ********** ********** **********

             ....|....| ....|....| ....|....| ....|....| ....|....| ....|....|
                  4925       4935       4945       4955       4965       4975
2a_SalI      ATAGCCGACG TCACAGAATC TAATAACTTG GAGAGCCTAA ATGGGAAGGA AGCAGATTAC
2a_Thai      ATAGCCGACG TCACAGAATC TAATAACTTG GAGAGCCTAA ATGGGAAGGA AGCAGATTAC
Conservation ********** ********** ********** ********** ********** **********

             ....|....| ....|....| ....|....| ....|....| ....|....| ....|....|
                  4985       4995       5005       5015       5025       5035
2a_SalI      ACGAAACGTA TAAGAAGTTT TAACAAATTG AAGCAGCTCG TGCAAGAGAA AGCAGCAAAA
2a_Thai      ACGAAACGTA TAAGAAGTTT TAACAAATTG AAGCAGCTCG TGCAAGAGAA AGCAGCAAAA
Conservation ********** ********** ********** ********** ********** **********

             ....|....| ....|....| ....|....| ....|....| ....|....| ....|....|
                  5045       5055       5065       5075       5085       5095
2a_SalI      GTAGAAGAAA TTTCCTCCGA TACGGATAAC ATAGAAAAAG AACTGACAGA ACATAAAATA
2a_Thai      GTAGAAGAAA TTTCCTCCGA TACGGATAAC ATAGAAAAAG AACTGACAGA ACATAAAATA
Conservation ********** ********** ********** ********** ********** **********

             ....|....| ....|....| ....|....| ....|....| ....|....| ....|....|
                  5105       5115       5125       5135       5145       5155
2a_SalI      ATTTTTGAAG TGGGACTTAC CGAAAGGTTA ATCGAAATAG TTAAAAATAG AAAATCATAC
2a_Thai      ATTTTTGAAG TGGGACTTAC CGAAAGGTTA ATCGAAATAG TTAAAAATAG AAAATCATAC
Conservation ********** ********** ********** ********** ********** **********

             ....|....| ....|....| ....|....| ....|....| ....|....| ....|....|
                  5165       5175       5185       5195       5205       5215
2a_SalI      GTAGACACAA CAAAGGAATT GCTAAATTCA TCATTAAACA ATTTTGCTTC CCTCTTTAAT
2a_Thai      GTAGACACAA CAAAGGAATT GCTAAATTCA TCATTAAACA ATTTTGCTTC CCTCTTTAAT
Conservation ********** ********** ********** ********** ********** **********

             ....|....| ....|....| ....|....| ....|....| ....|....| ....|....|
                  5225       5235       5245       5255       5265       5275
2a_SalI      GGATTGGACT TAAATGGATA CAATCCAAAA GCAAACCTAG AAATGTACAC ACAAAAACTG
2a_Thai      GGATTGGACT TAAAAGGATA CAATCCAAAA GCAAACCTAG AAATGTACAC ACAAAAACTG
Conservation ********** ****.***** ********** ********** ********** **********

             ....|....| ....|....| ....|....| ....|....| ....|....| ....|....|
                  5285       5295       5305       5315       5325       5335
2a_SalI      AACACAATAC ATAACGAATT TATGGCATCT CACAAAATAT TTGATGAAAA ATCAAAGAAG
2a_Thai      AACACAATAC ATAACGAATT TATGGCATCT CACAAAATAT TTGATGAAAA ATCAAAGAAG
Conservation ********** ********** ********** ********** ********** **********

             ....|....| ....|....| ....|....| ....|....| ....|....| ....|....|
                  5345       5355       5365       5375       5385       5395
2a_SalI      GTCTTAGACA AAGATGTGAA CTTCACGGAA GCTAAAACAC TCAGGGAGGA GGCGCAAAAA
2a_Thai      GTCTTAGACA AAGATGTGAA CTTCACGGAA GCTAAAACAC TCAGGGAGGA GGCGCAAAAA
Conservation ********** ********** ********** ********** ********** **********
```

```
             ....|....| ....|....| ....|....| ....|....| ....|....| ....|....|
                  5405       5415       5425       5435       5445       5455
2a_SalI      GAAGATGTTA CTCTTAAAAA TAAAGAGGAA GAAGCAAAAT CGTACTTGAG TGATATCAAA
2a_Thai      GAAGATGTTA CTCTTAAAAA TAAAGAGGAA GAAGCAAAAT CGTACTTGAG TGATATCAAA
Conservation ********** ********** ********** ********** ********** **********

             ....|....| ....|....| ....|....| ....|....| ....|....| ....|....|
                  5465       5475       5485       5495       5505       5515
2a_SalI      AAAAAAGAAT CTTTCGAATT TATACTTCAC ATGAAGGAAA AATTAAACCA AATTAGTAAA
2a_Thai      AAAAAAGAAT CTTTCGAATT TATACTTCAC ATGAAGGAAA AATTAAACCA AATTAGTAAA
Conservation ********** ********** ********** ********** ********** **********

             ....|....| ....|....| ....|....| ....|....| ....|....| ....|....|
                  5525       5535       5545       5555       5565       5575
2a_SalI      ATGTGTGAAC AGCAATATGA ACAAGCTGAT AAAGGCTATA GTGAAGTCAA AACAAGCATT
2a_Thai      ATGTGTGAAC AGCAATATGA ACAAGCTGAT AAAGGCTATA GTGAAGTCAA AACAAGCATT
Conservation ********** ********** ********** ********** ********** **********

             ....|....| ....|....| ....|....| ....|....| ....|....| ....|....|
                  5585       5595       5605       5615       5625       5635
2a_SalI      GATCGCATTG CAAATTTAAA TGATGAAAAT AGTATAGCGG ATGTACTCAA AGAAGCAAAC
2a_Thai      GATCGCATTG CAAATTTAAA TGATGAAAAT AGTATAGCGG ATGTACTCAA AGAAGCAAAC
Conservation ********** ********** ********** ********** ********** **********

             ....|....| ....|....| ....|....| ....|....| ....|....| ....|....|
                  5645       5655       5665       5675       5685       5695
2a_SalI      GATAAAAATG AGCAAGTACA AAATCTGACA CATTATACAT ATAAAAATGA AGCACAAAAT
2a_Thai      GATAAAAATG AGCAAGTACA AAATCTGACA CATTATACAT ATAAAAATGA AGCACAAAAT
Conservation ********** ********** ********** ********** ********** **********

             ....|....| ....|....| ....|....| ....|....| ....|....| ....|....|
                  5705       5715       5725       5735       5745       5755
2a_SalI      GTGCTAAGGC ATATGGCAAA GTCGGCAAAT TTTATAGGCA TTAACCTAGT AACAGGAATA
2a_Thai      GTGCTAAGGC ATATGGCAAA GTCGGCAAAT TTTATAGGCA TTAACCTAGT AACAGGAATA
Conservation ********** ********** ********** ********** ********** **********

             ....|....| ....|....| ....|....| ....|....| ....|....| ....|....|
                  5765       5775       5785       5795       5805       5815
2a_SalI      CAACCGACGG AATTATCATC ACAAGCAAGC GAATCGACAA CGCCAGAGTT AAAATTTGAA
2a_Thai      CAACCGACGG AATTATCATC ACAAGCAAGC GAATCGACAA CGCCAGAGTT AAAATTTGAA
Conservation ********** ********** ********** ********** ********** **********

             ....|....| ....|....| ....|....| ....|....| ....|....| ....|....|
                  5825       5835       5845       5855       5865       5875
2a_SalI      TCAGAAGGGG AAATGAAATT GGAAATTCTA ACATTATCAG GAAATACTAC AAAATTGGAC
2a_Thai      TCAGAAGGGG AAATGAAATT GGAAATTCTA ACATTATCAG GAAATACTAC AAAATTGGAC
Conservation ********** ********** ********** ********** ********** **********

             ....|....| ....|....| ....|....| ....|....| ....|....| ....|....|
                  5885       5895       5905       5915       5925       5935
2a_SalI      TATTATAAAA ATATGAAAGA CGCCTACCAG AGCGTCCTTT CCATATTTAA ATACTCTCAT
2a_Thai      TATTATAAAA ATATGAAAGA CGCCTACCAG AGCGTCCTTT CCATATTTAA ATACTCTCAT
Conservation ********** ********** ********** ********** ********** **********

             ....|....| ....|....| ....|....| ....|....| ....|....| ....|....|
                  5945       5955       5965       5975       5985       5995
2a_SalI      GGTATAGACG AAAAACAAAG AGAAAGCCAA AAAATAACCG AAAGTGCAAA TGGCTCATAT
2a_Thai      GGTATAGACG AAAAACAAAG AGAAAGCCAA AAAATAACCG AAAGTGCAAA TGGCTCATAT
Conservation ********** ********** ********** ********** ********** **********
```

```
             ....|....| ....|....| ....|....| ....|....| ....|....| ....|....|
                  6005       6015       6025       6035       6045       6055
2a_SalI      CTTAATAATA AGGAAATAAA TGAATTCAAA GGAAGACTGA ACAATGTGAA GAGTAAACAA
2a_Thai      CTTAATAATA AGGAAATAAA TGAATTCAAA GGAAGACTGA ACAATGTGAA GAGTAAACAA
Conservation ********** ********** ********** ********** ********** **********

             ....|....| ....|....| ....|....| ....|....| ....|....| ....|....|
                  6065       6075       6085       6095       6105       6115
2a_SalI      ACAGCCATTT CAAACAAAAT TGATAACGCT ACTACGCTGC TGCACAATTT GAACAAAATT
2a_Thai      ACAGCCATTT CAAACAAAAT TGATAACGCT ACTACGCTGC TGCACAATTT GAACAAAATT
Conservation ********** ********** ********** ********** ********** **********

             ....|....| ....|....| ....|....| ....|....| ....|....| ....|....|
                  6125       6135       6145       6155       6165       6175
2a_SalI      AAAACGGATG ATAAGAATTA CGACACAATT TTAGAAAAAG ACGCTTCTGA AGAATTGAAG
2a_Thai      AAAACGGATG ATAAGAATTA CGACACAATT TTAGAAAAAG ACGCTTCTGA AGAATTGAAG
Conservation ********** ********** ********** ********** ********** **********

             ....|....| ....|....| ....|....| ....|....| ....|....| ....|....|
                  6185       6195       6205       6215       6225       6235
2a_SalI      AGGCGACGTG ATTCATTTAA TCAAGAAATG AAAAACACCG TCGATGGCTT AAAATTGAAG
2a_Thai      AGGCGACGTG ATTCATTTAA TCAAGAAATA AAAAACACCG TCGATGGCTT AAAATTGAAG
Conservation ********** ********** *********. ********** ********** **********

             ....|....| ....|....| ....|....| ....|....| ....|....| ....|....|
                  6245       6255       6265       6275       6285       6295
2a_SalI      GAAATCCAAG AAAAGTTTAA TGAGCAAGTA AAACTCCTAC AAAATTTAGA AACAAAAGTG
2a_Thai      GAAATCCAAG AAAAGTTTAA TGAGCAAGTA AAACTCCTAC AAAATTTAGA AACAAAAGTG
Conservation ********** ********** ********** ********** ********** **********

             ....|....| ....|....| ....|....| ....|....| ....|....| ....|....|
                  6305       6315       6325       6335       6345       6355
2a_SalI      AGCACACTAA ATGTGCATGA GGGAAACGCT ACCGAAACTG TGAAAAAAGA AAACACTGCC
2a_Thai      AGCACACTAA ATGTGCATGA GGGAAACGCT ACCGAAACTG TGAAAAAAGA AAACACTGCC
Conservation ********** ********** ********** ********** ********** **********

             ....|....| ....|....| ....|....| ....|....| ....|....| ....|....|
                  6365       6375       6385       6395       6405       6415
2a_SalI      GTTGATGCAA TTCAAGCCGC AATGGAAGGC ATAGAAAAAG ATGTCGAATA TATCAACTAC
2a_Thai      GTTGATGCAA TTCAAGCCGC AATGGAAGGC ATAGAAAAAG ATGTCGAATA TATCAACTAC
Conservation ********** ********** ********** ********** ********** **********

             ....|....| ....|....| ....|....| ....|....| ....|....| ....|....|
                  6425       6435       6445       6455       6465       6475
2a_SalI      TCATATGATG AACTATTAAA GAAAGGACAA AAAATTGAAA ATCAGAGATA CACATCCATA
2a_Thai      TCATATGATG AACTATTAAA GAAAGGACAA AAAATTGAAA ATCAGAGATA CACATCCATA
Conservation ********** ********** ********** ********** ********** **********

             ....|....| ....|....| ....|....| ....|....| ....|....| ....|....|
                  6485       6495       6505       6515       6525       6535
2a_SalI      AGGGAAAACC TCACCAATAA AATAGCGAAT GACTCCTCAG CAATCAACAA AATAAAGAAA
2a_Thai      AGGGAAAACC TCACCAATAA AATAGCGAAT GACTCCTCAG CAATCAACAA AATAAAGAAA
Conservation ********** ********** ********** ********** ********** **********

             ....|....| ....|....| ....|....| ....|....| ....|....| ....|....|
                  6545       6555       6565       6575       6585       6595
2a_SalI      AAAGCTCAAC AGTATTTAGC ATACATTAAG AATAACTACA ACTCTATTTA TAATGATACT
2a_Thai      AAAGCTCAAG AGTATTTAGC ATACATTAAG AATAACTACA ACTCTATTTA TAATGATACT
Conservation *********. ********** ********** ********** ********** **********
```

```
             ....|....| ....|....| ....|....| ....|....| ....|....| ....|....|
                  6605       6615       6625       6635       6645       6655
2a_SalI      GGCACTTTAA ATGAATACTT CGACACCAAA CGGTTAAGTA ACCACGATTT AACAAATGTT
2a_Thai      GGCACTTTAA ATGAATACTT CGACACCAAA CGGTTAAGTA ACCACGATTT AACAAATGTT
Conservation ********** ********** ********** ********** ********** **********

             ....|....| ....|....| ....|....| ....|....| ....|....| ....|....|
                  6665       6675       6685       6695       6705       6715
2a_SalI      CAGGAAGCGA CCAGATTACA CATTGAAATG TCCGCAGCCG TTGAAGCATC AGAAGAGATA
2a_Thai      CAGGAAGCGA CCAGATTACA CATTGAAATG TCCGCAGCCG TTGAAGCATC AGAAGAGATA
Conservation ********** ********** ********** ********** ********** **********

             ....|....| ....|....| ....|....| ....|....| ....|....| ....|....|
                  6725       6735       6745       6755       6765       6775
2a_SalI      ATTGCAGATA TGAAAAATGA ATTCATAACG AACACAGAAG CGGATATAAG CGCTTTACAA
2a_Thai      ATTGCAGATA TGAAAAATGA ATTCATAACG AACACAGAAG CGGATATAAG CGCTTTACAA
Conservation ********** ********** ********** ********** ********** **********

             ....|....| ....|....| ....|....| ....|....| ....|....| ....|....|
                  6785       6795       6805       6815       6825       6835
2a_SalI      AATAGCGCAG ACAGATTAAA GTCGCTTTAT AGTAATCTAA AACGCAAACA AATTAGTATA
2a_Thai      AATAGCGCAG ACAGATTAAN GTCGCTTTAT AGTAATCTAA AACGCAAACA AATTAGTATA
Conservation ********** *********  ********** ********** ********** **********

             ....|....| ....|....| ....|....| ....|....| ....|....| ....|....|
                  6845       6855       6865       6875       6885       6895
2a_SalI      AACCAAATTT ACAAAAAGAT TAACTTAATT AAGTTGCAAG AAATTAAAAC AAGCGCTAAC
2a_Thai      AACCAAATTT ACAAAAAGAT TAACTTAATT AAGTTGCAAG AAATTAAAAC AAGCGCTAAC
Conservation ********** ********** ********** ********** ********** **********

             ....|....| ....|....| ....|....| ....|....| ....|....| ....|....|
                  6905       6915       6925       6935       6945       6955
2a_SalI      AAATATATGG ACATAGCCAA ACTGTTTAAC AATGTGCTAG AGGCCCAACA TAAAGAATTG
2a_Thai      AAATATATGG ACATAGCCAA ACTGTTTAAC AATGTGCTAG AGGCCCAACA TAAAGAATTG
Conservation ********** ********** ********** ********** ********** **********

             ....|....| ....|....| ....|....| ....|....| ....|....| ....|....|
                  6965       6975       6985       6995       7005       7015
2a_SalI      GCACAAGATA GAAGTAAAAT ATTGCAAGCT AAGGAAAACT CCATTAATAT GCAGCAACTG
2a_Thai      GCACAAGATA TAAGTAAAAT ATTGCAAGCT AAGGAAAACT CCATTAATAT GCAGCAACTG
Conservation ********** .********* ********** ********** ********** **********

             ....|....| ....|....| ....|....| ....|....| ....|....| ....|....|
                  7025       7035       7045       7055       7065       7075
2a_SalI      TTAGAATCTC ACATTGATAA ACTTCGAGCC TTGATAACCA ATATTGATAA AGAACTGATA
2a_Thai      TTAGAATCTC ACATTGATAA ACTTCGAGCC TTGATAACCA ATATTGATAA AGAACTGATA
Conservation ********** ********** ********** ********** ********** **********

             ....|....| ....|....| ....|....| ....|....| ....|....| ....|....|
                  7085       7095       7105       7115       7125       7135
2a_SalI      GAATTAACAA ATGGTAAAAT TAAAGAATCA AATCGACGTA TTAGTCAAAT TTCCCCAATG
2a_Thai      GAATTAACAA ATGGTAAAAT TAAAGCATCA AATCGACGTA TTAGTCAAAT TTCCCCAATG
Conservation ********** ********** *****.**** ********** ********** **********

             ....|....| ....|....| ....|....| ....|....| ....|....| ....|....|
                  7145       7155       7165       7175       7185       7195
2a_SalI      GGGCAAGGCA AATTGTTCAG CACCCCAGAA GGACAAGCTT ATAATAATTT ACACAACACA
2a_Thai      GGGCAAGGCA AATTGTTCAG CACCCCAGAA GGACAAGCTT ATAATAATTT ACACAACACA
Conservation ********** ********** ********** ********** ********** **********
```

```
             ....|....| ....|....| ....|....| ....|....| ....|....| ....|....|
                 7205       7215       7225       7235       7245       7255
2a_SalI      GGATATAACC ATTATGGAAG TGGAAACCAT TCAAGAGGAA GAAATGAAAA TGGAAATGTC
2a_Thai      GGATATAACC ATTATGGAAG TGGAAACCAT TCAAGAGGAA GAAATGAAAA TGGAAATGTC
Conservation ********** ********** ********** ********** ********** **********

             ....|....| ....|....| ....|....| ....|....| ....|....| ....|....|
                 7265       7275       7285       7295       7305       7315
2a_SalI      CGTTTTGCAG CAGGGATTGT TGTGGGATTT GGTGTTTGTT CCTTTTTTGC TTCGGCATTG
2a_Thai      CGTTTTGCAG CAGGGATTGT TGTGGGATTT GGTGTTTGTT CCTTTTTTGC TTCGGCATTG
Conservation ********** ********** ********** ********** ********** **********

             ....|....| ....|....| ....|....| ....|....| ....|....| ....|....|
                 7325       7335       7345       7355       7365       7375
2a_SalI      TTTAAAGGCA AAGGGGAAAA CGAAACATAT GGAAGAGATC TAAATTCACG TGATGAAGAA
2a_Thai      TTTAAAGGCA AAGGGGAAAA CGAAACATAT GGAAGAGATC TAAATTCACG TGATGAAGAA
Conservation ********** ********** ********** ********** ********** **********

             ....|....| ....|....| ....|....| ....|....| ....|....| ....|....|
                 7385       7395       7405       7415       7425       7435
2a_SalI      TTTGAAGGCA AAAACAATGG AAATCTTCAA GATAAGGAAG AAATTATTGA AGTGTCCTTT
2a_Thai      TTTGAAGCCA AAAACAATGG AAATCTTCAA GATAAGGAAG AAATTATTGA AGTGTCCTTT
Conservation ******* ** ********** ********** ********** ********** **********

             ....|....| ....|....| ....
                 7445       7455
2a_SalI      CACGAAAGTG AAAATGTGTA TTAA
2a_Thai      CACGAAAGTG AAAATGCGTA TTAA
Conservation ********** ****** *** ****
```

Alignment: Pvrbp2b

```
               ....|....| ....|....| ....|....| ....|....| ....|....| ....|....|
                   5         15         25         35         45         55
2b_SalI        ATGGAAATAA ACGCGCTTTG GACGATTTTG TGCAATCTTC TATTGGTCCT TTTAGCGTCA
2b_Thai        ATGGAAATAA ACGCGCTTTG GACGATTTTG TGCAATCTTC TATTTGTCCT TTTAGCGTCA
Conservation   ********** ********** ********** ********** **** ***** **********

               ....|....| ....|....| ....|....| ....|....| ....|....| ....|....|
                  65         75         85         95        105        115
2b_SalI        AGTAGAGATG TCAACAGGAG TAAACCGAAC AGATTTAAAA AGGAACTCAC GTTATCACCC
2b_Thai        AGTAGAGATG TCAACAGGAG TAAACCGAAC AGATTTAAAA AGGAACTCAC GTTATCACCC
Conservation   ********** ********** ********** ********** ********** **********

               ....|....| ....|....| ....|....| ....|....| ....|....| ....|....|
                 125        135        145        155        165        175
2b_SalI        ATATATCAAA ATTTAATTCG AAGCAGTCAA GTACAACATG ATAAAAACTA TGAAGTTGAT
2b_Thai        ATATATCAAA ATTTAATTCG AAGCAGTCAA GTACAACATG ATAAAAACTA TGAAGTTGAT
Conservation   ********** ********** ********** ********** ********** **********

               ....|....| ....|....| ....|....| ....|....| ....|....| ....|....|
                 185        195        205        215        225        235
2b_SalI        GATGGCAAAA TTAACGATGG AGGTGACGAA AAAGCGCATT CCCCTGATAG CTCATTCTCG
2b_Thai        GATGGCAAAA TTAACGATGG AGGTGACGAA AAAGCGCATT CCCCTGATAG CTCATTCTCG
Conservation   ********** ********** ********** ********** ********** **********

               ....|....| ....|....| ....|....| ....|....| ....|....| ....|....|
                 245        255        265        275        285        295
2b_SalI        GGGGATTCAG AAAGGTATAG CGCTAAAGGA AAACCATCGT ACTATTCTTA CATAAAAAGA
2b_Thai        GGGGATTCAG AAAGGTATAG CGCTAAAGGA AAACCATCGT ACTATTCTTA CATAAAAAGA
Conservation   ********** ********** ********** ********** ********** **********

               ....|....| ....|....| ....|....| ....|....| ....|....| ....|....|
                 305        315        325        335        345        355
2b_SalI        GGGGACATAT ATACACACAG TTCAAACACA AATAAAAGCT TAAATGATAG TAACAAGTTG
2b_Thai        GGGGACATAT ATACACACAG TTCAAACACA AATAAAAGCT TAAATGATAG TAACAAGTTG
Conservation   ********** ********** ********** ********** ********** **********

               ....|....| ....|....| ....|....| ....|....| ....|....| ....|....|
                 365        375        385        395        405        415
2b_SalI        AATAGGAATA CGATTCCAAA CGAATTTATT CAAACTAGTA ATAAGGAAAA ACCTGAGAAG
2b_Thai        AATAGGAATA CGATTCCAAA CGAATTTATT CAAACTAGTA ATAAGAAAAA ACCTGAGAAG
Conservation   ********** ********** ********** ********** *****.**** **********

               ....|....| ....|....| ....|....| ....|....| ....|....| ....|....|
                 425        435        445        455        465        475
2b_SalI        AAAACTACCA AAAGTGAACC AGCTCCAAAA AACGGTCAAA CGTCTCCTTC ACCAAAAGAG
2b_Thai        AAAACTACCA AAAGTGAACC AGCTCCAAAA AACGGTCAAA CGTCTCCTTC ACCAAAAGAG
Conservation   ********** ********** ********** ********** ********** **********

               ....|....| ....|....| ....|....| ....|....| ....|....| ....|....|
                 485        495        505        515        525        535
2b_SalI        CCTTCCCCCG AATCTACACA AATTACCAAT ACAACTGATA ACATAGACTA CTTCGACATT
2b_Thai        CCTTCCCCCG AATCTACACA AATTACCAAT ACAACTGATA ACATAGACTA CTTCGACATT
Conservation   ********** ********** ********** ********** ********** **********

               ....|....| ....|....| ....|....| ....|....| ....|....| ....|....|
                 545        555        565        575        585        595
2b_SalI        TCAGATGAGA GCAATTACTA TTTAATTTCC CAGTTAAGAC CACATTTTTC TAATATATAC
2b_Thai        TCAGATGAGA GCAATTACTA TTTAATTTCC CAGTTAAGAC CACATTTTTC TAATATATAC
Conservation   ********** ********** ********** ********** ********** **********
```

```
             ....|....| ....|....| ....|....| ....|....| ....|....| ....|....|
                   605        615        625        635        645        655
2b_SalI      TTTTTCGATG AATTTAAACG TTACGCATCA TATCACACAG AAATCAAACG TTATGAGGAC
2b_Thai      TTTTTCGATG AATTTAAACG TTACGCATCA TATCACACAG AAATCAAACG TTATGAGGAC
Conservation ********** ********** ********** ********** ********** **********

             ....|....| ....|....| ....|....| ....|....| ....|....| ....|....|
                   665        675        685        695        705        715
2b_SalI      ATACATAAGA CAAAAGTTAA TTCCCTTTTG AACGAAGCAT CCAGAGCAAT CGGCATTTGT
2b_Thai      ATACATAAGA AAAAAGTTAA TTCCCTTTTG AACGAAGCAT CCAGAGCAAT CGGCATTTGT
Conservation ********** .********* ********** ********** ********** **********

             ....|....| ....|....| ....|....| ....|....| ....|....| ....|....|
                   725        735        745        755        765        775
2b_SalI      AACAGGGCAA AAAATACAGT GAAGGGGTTA ATAAATATTT TAGAGAATCC CCAAAAGTTT
2b_Thai      AACAGGGCAA AAAATACAGT GAAGGGGTTA ATAAATATTT TAGAGAATCC CCAAAAGTTT
Conservation ********** ********** ********** ********** ********** **********

             ....|....| ....|....| ....|....| ....|....| ....|....| ....|....|
                   785        795        805        815        825        835
2b_SalI      AAAACGCAAA GAGAATCATA TGATGTAAAA CTTCGCCAAT ATGAAGAAAA AAAGGAAGCA
2b_Thai      AAAACGCAAA GAGAATCATA TGATGTAAAA CTTCGCCAAT ATGAAGAAAA AAAGGAAGCA
Conservation ********** ********** ********** ********** ********** **********

             ....|....| ....|....| ....|....| ....|....| ....|....| ....|....|
                   845        855        865        875        885        895
2b_SalI      TTTAGGGGAT GCTTACTAAA TAAAAATCGG AAGAATTTGG ATCAAATAAA AAAAATAAAT
2b_Thai      TTTAGGGGAT GCTTACTAAA TAAAAATCGG AAGAATTTGG ATCAAATAAA AAAAATAAAT
Conservation ********** ********** ********** ********** ********** **********

             ....|....| ....|....| ....|....| ....|....| ....|....| ....|....|
                   905        915        925        935        945        955
2b_SalI      AACGAAATTC GTGATCTGCT AGAAAAACTG AAATGCTCAC AGGACTGTCA AACTAATGTA
2b_Thai      AACGAAATTC GTGATCTGCT AGAACAACTG AAATGCTCAC AGTACTGTCA AACTAATGTA
Conservation ********** ********** ****.***** ********** ** ******* **********

             ....|....| ....|....| ....|....| ....|....| ....|....| ....|....|
                   965        975        985        995       1005       1015
2b_SalI      TATTTTGACA TGATTAAAAT ATACTTGGTA GATTTTAAAA AAATGCCTTA CGAAAATTAT
2b_Thai      TATTTTGACA TGATTAAAAT ATACTTGGTA GATTTTAAAA AAATGCCTTA CGAAAATTAT
Conservation ********** ********** ********** ********** ********** **********

             ....|....| ....|....| ....|....| ....|....| ....|....| ....|....|
                  1025       1035       1045       1055       1065       1075
2b_SalI      GACACTTTTA TAAAACAGTA TAAAAATTCT TACCTTTCTG GTGTTGATAT GATAAGGAAA
2b_Thai      GACACTTTTA TAAAACAGTA TAAAAATTCT TACCTTTCTG GTGTTGATAT GATAAGGAAA
Conservation ********** ********** ********** ********** ********** **********

             ....|....| ....|....| ....|....| ....|....| ....|....| ....|....|
                  1085       1095       1105       1115       1125       1135
2b_SalI      ATTGAAAAAC AAATAGACAA CCCAGTTACC ATAAATGCGA TAAAATTCAC TCAAAAGGAG
2b_Thai      ATTGAAAAAC AAATAGACAA CCCAGTTACC ATAAATGCGA TAAAATTCAC TCAAAAGGAG
Conservation ********** ********** ********** ********** ********** **********

             ....|....| ....|....| ....|....| ....|....| ....|....| ....|....|
                  1145       1155       1165       1175       1185       1195
2b_SalI      ATGGGATACA TTATAGACAG ATTTGAATAT CACCTGCAAA AAGTGAAACA TTCCATTGAT
2b_Thai      ATGGGATACA TTATAGACAG ATTTGAATAT CACCTGCAAA AAGTGAAACA TTCCATTGAT
Conservation ********** ********** ********** ********** ********** **********
```

```
             ....|....| ....|....| ....|....| ....|....| ....|....| ....|....|
                  1205       1215       1225       1235       1245       1255
2b_SalI      CAAGTGACAG CATTGTCCGA CGGGGTTAAA CCAAAACAGG TCACTAAAAA TCGTCTTAAA
2b_Thai      CAAGTGACAG CATTGTCCGA CGGGGTTAAA CCAAAACAGG TCACTAAAAA TCGTCTTAAA
Conservation ********** ********** ********** ********** ********** **********

             ....|....| ....|....| ....|....| ....|....| ....|....| ....|....|
                  1265       1275       1285       1295       1305       1315
2b_SalI      GAGTACTACT TTAATATAGG TAACTACTAC TCCATTTTTA AATTCGGCAA AGATAGTCTC
2b_Thai      GAGTACTACT TTAATATAGG TAACTACTAC TCCATTTTTA AATTCGGCAA AGATAGTCTC
Conservation ********** ********** ********** ********** ********** **********

             ....|....| ....|....| ....|....| ....|....| ....|....| ....|....|
                  1325       1335       1345       1355       1365       1375
2b_SalI      AATATGTTGA ATAAAGCATT AATACATAAA GAAAAAATAG TCCACAACTT ACTTGGTGAA
2b_Thai      AATATGTTGA ATAAAGCATT AATACATAAA GAAAAAATAG TCCACAACTT ACTTGGTGAA
Conservation ********** ********** ********** ********** ********** **********

             ....|....| ....|....| ....|....| ....|....| ....|....| ....|....|
                  1385       1395       1405       1415       1425       1435
2b_SalI      TTATTTGGCC ATTTAGAAGA GAGAATATCT AAACTTATAG ATTCAGAGTA CTTCATAACA
2b_Thai      TTATTTGGCC ATTTAGAAGA GAGAATATCT AAACTTATAG ATTCAGAGTA CTTCATAACA
Conservation ********** ********** ********** ********** ********** **********

             ....|....| ....|....| ....|....| ....|....| ....|....| ....|....|
                  1445       1455       1465       1475       1485       1495
2b_SalI      GAAAGTAATA ACATCATTTC ACAATCCGAA GAAACTTTAA AACTTGCGGA AGACGTATAT
2b_Thai      GAAAGTAATA ACATCATTTC ACAATCCGAA GAAACTTTAA AACTTGCGAA AGACGTATAT
Conservation ********** ********** ********** ********** ********.* **********

             ....|....| ....|....| ....|....| ....|....| ....|....| ....|....|
                  1505       1515       1525       1535       1545       1555
2b_SalI      GATAAAAACA CCAAACTGAT AGAGGACCTT ACACTGTATC CACACTTAGA AATTAACGAA
2b_Thai      GATAAAAACA CCAAACTGAT AGAGGACCTT ACACTGTATC CACACTTAGA AATTAACGAA
Conservation ********** ********** ********** ********** ********** **********

             ....|....| ....|....| ....|....| ....|....| ....|....| ....|....|
                  1565       1575       1585       1595       1605       1615
2b_SalI      TTCAAAAAAG ACTACGACAA CAATGTGGAA GATTTGAGAG AATCTATCAT CTACATACAA
2b_Thai      TTCAAAAAAG ACTACGACAA CAATGTGGAA GATTTGAGAG AATCTATCAT CTACATACAA
Conservation ********** ********** ********** ********** ********** **********

             ....|....| ....|....| ....|....| ....|....| ....|....| ....|....|
                  1625       1635       1645       1655       1665       1675
2b_SalI      TCATACGTCT CATCTATTAA AAGTGCGTAT AGATATAATG TGTTAGAAAA GGACTCTGTA
2b_Thai      TCATACGTCT CATCTATTAA AAGTGCGTAT AGATATAATG TGTTAGAAAA GGACTCTGTA
Conservation ********** ********** ********** ********** ********** **********

             ....|....| ....|....| ....|....| ....|....| ....|....| ....|....|
                  1685       1695       1705       1715       1725       1735
2b_SalI      GAAAGTAAAC AAAAGAACAT ACCAGCTAAC AGTAATGCCC AGAAGAAAGT CGATGAGCTG
2b_Thai      GAAAGTAAAC GAAAGAACAT ACCAGCTAAC AGTAATGCCC AGGAGAAAGT CGATGAGCTG
Conservation ********** .********* ********** ********** **.******* **********

             ....|....| ....|....| ....|....| ....|....| ....|....| ....|....|
                  1745       1755       1765       1775       1785       1795
2b_SalI      TTGAGCATTA TAGATAGCAT AAGTTATAGC AATTTTTCCG TAGCCGAGAA TTTTCAAAAA
2b_Thai      TTGAGCATTA TAGATAGCAT AAGTTATAGC AATTTTTCCG TAGCCGAGAA TTTTCAAAAA
Conservation ********** ********** ********** ********** ********** **********
```

```
             ....|....| ....|....| ....|....| ....|....| ....|....| ....|....|
                 1805       1815       1825       1835       1845       1855
2b_SalI      ATGAAAGATT ACTATAAAGA AATCGAAAAA TTAAAAATCA AAATACTGCA ATTAATAGAA
2b_Thai      ATGAAAGATT ACTATAAAGA AATCGAAAAA TTAAAAATCA AAATACTGCA ATTAATAGAA
Conservation ********** ********** ********** ********** ********** **********

             ....|....| ....|....| ....|....| ....|....| ....|....| ....|....|
                 1865       1875       1885       1895       1905       1915
2b_SalI      GCCATTAAAA AATACCAACA ACATGTGGAA GAATTAATCA ACAAGGAAAA AGCAGTTGCG
2b_Thai      GCCATTAAAA AATACCAACA ACATGTGGAA GAATTAATCA ACAAGGAAAA AGCAGTTGCG
Conservation ********** ********** ********** ********** ********** **********

             ....|....| ....|....| ....|....| ....|....| ....|....| ....|....|
                 1925       1935       1945       1955       1965       1975
2b_SalI      ATTCTCAAAG AAGATATAAA TAAAATAATA GAATATATAA AAGGAATAAT AGAAAAATTG
2b_Thai      ATTCTCAAAG AAGATATAAA TAAAATAATA GAATATATAA AAGGAATAAT AGAAAAATTG
Conservation ********** ********** ********** ********** ********** **********

             ....|....| ....|....| ....|....| ....|....| ....|....| ....|....|
                 1985       1995       2005       2015       2025       2035
2b_SalI      AAACAACTAA TTTCTGCGAA TAAAGATTTT GATAAAATTT TCCAGCAAGT TGAACAATTA
2b_Thai      AAACAACTAA TTTCTGCGAA TAAAGATTTT GATAAAATTT TCCAGCAAGT TGAACAATTA
Conservation ********** ********** ********** ********** ********** **********

             ....|....| ....|....| ....|....| ....|....| ....|....| ....|....|
                 2045       2055       2065       2075       2085       2095
2b_SalI      ATTAACGAAG CGCTTTTTAA CAAAGACCAA TTTGAACATA ATAAAAATGA CTTACACACC
2b_Thai      ATTAACGAAG CGCTTTTTAA CAAAGACCAA TTTGAACATA ATAAAAATGA CTTACACACC
Conservation ********** ********** ********** ********** ********** **********

             ....|....| ....|....| ....|....| ....|....| ....|....| ....|....|
                 2105       2115       2125       2135       2145       2155
2b_SalI      AAAATGAAAG AAATAATGCA TACATTTCAC GAGCGTGATT TACAACAATT TTTGGACAAT
2b_Thai      AAAATGAAAG AAATAATGCA TACATTTCAC GAGCGTGATT TACAACAATT TTTGGACAAT
Conservation ********** ********** ********** ********** ********** **********

             ....|....| ....|....| ....|....| ....|....| ....|....| ....|....|
                 2165       2175       2185       2195       2205       2215
2b_SalI      ATGTCGAAAT TTTTAAAGGA CCAAGAAGCT TCCTACCAAA ATGCAGACAG TAAGGAAAAG
2b_Thai      ATGTCGAAAT TTTTAAAGGA CCAAGAAGCT TCCTACCAAA ATGCAGACAG TAAGGAAAAG
Conservation ********** ********** ********** ********** ********** **********

             ....|....| ....|....| ....|....| ....|....| ....|....| ....|....|
                 2225       2235       2245       2255       2265       2275
2b_SalI      CTTGACCAAC TGCTAACAAC AGTAAAGGCA AAACAAGATG AACTTAAAGA GATGAAATGC
2b_Thai      CTTGACCAAC TGCTAACAAC AGTAAAGGCA AAACAAGATG AACTTAAAGA GATGAAATGC
Conservation ********** ********** ********** ********** ********** **********

             ....|....| ....|....| ....|....| ....|....| ....|....| ....|....|
                 2285       2295       2305       2315       2325       2335
2b_SalI      GATGACATTC CAGACATAAT CGACAATTTG AAAAAAGAAT CGCAAAATGT TTTAAATCTA
2b_Thai      GATGACATTC CAGACATAAT CGACAATTTG AAAAAAGAAT CGCAAAATGT TTTAAATCTA
Conservation ********** ********** ********** ********** ********** **********

             ....|....| ....|....| ....|....| ....|....| ....|....| ....|....|
                 2345       2355       2365       2375       2385       2395
2b_SalI      AAAGATGAGG TCATAAATAA ACAGTTTGAA AATATGCGCA CAGAAATGTC AAGTTCATTG
2b_Thai      AAAGATGAGG TCATAAATAA ACAGTTTGAA AATATGCGCA CAGAAATGTC AAGTTCATTG
Conservation ********** ********** ********** ********** ********** **********
```

```
             ....|....| ....|....| ....|....| ....|....| ....|....| ....|....|
                 2405       2415       2425       2435       2445       2455
2b_SalI      GATCAAATGA CCAAAGAGTA TAACGCTCTC AAAAGCAGCA TAGAGGAATA CGAAGCGGAA
2b_Thai      GATCAAATGA CCAAAGAGTA TAACGCTCTC AAAAGCAGCA TAGAGGAATA CGAAGCGGAA
Conservation ********** ********** ********** ********** ********** **********

             ....|....| ....|....| ....|....| ....|....| ....|....| ....|....|
                 2465       2475       2485       2495       2505       2515
2b_SalI      AAAAAAGGAA TAGAAAACCA TAAACAAAAT ATAATTAAAA GAAAAAATAC ATTCATCGTT
2b_Thai      AAAAAAGGAA TAGAAAACCA TAAACAAAAT ATAATTAAAA GAAAAAATAC ATTCATCGTT
Conservation ********** ********** ********** ********** ********** **********

             ....|....| ....|....| ....|....| ....|....| ....|....| ....|....|
                 2525       2535       2545       2555       2565       2575
2b_SalI      GCTGAACATG AAAATGATGA AGATGTGCCG GAAGGGAAAA ATACCTACAA CGAGTTTATA
2b_Thai      GCTGAACATG AAAATGATGA AGATGTGCCG GAAGGGAAAA ATACCTACAA CGAGTTTATA
Conservation ********** ********** ********** ********** ********** **********

             ....|....| ....|....| ....|....| ....|....| ....|....| ....|....|
                 2585       2595       2605       2615       2625       2635
2b_SalI      AGCAATAAGG ACACAATTTT GCAAAAAGAA AGCGCAATTA GCAACCAGAT GAACACATTA
2b_Thai      AGCAATAAGG ACACAATTTT GCAAAAAGAA AGCGCAATTA GCAACCAGAT GAACACATTA
Conservation ********** ********** ********** ********** ********** **********

             ....|....| ....|....| ....|....| ....|....| ....|....| ....|....|
                 2645       2655       2665       2675       2685       2695
2b_SalI      GAGGAAAAAA AAAGAAATAG AAAAACTACG TTACAAACAT ATGGTGACGC AATACAAAAA
2b_Thai      GAGGAAAAAA AAAGAAATAG AAAAACTACG TTACAAACAT ATGGTGACGC AATACAAAAA
Conservation ********** ********** ********** ********** ********** **********

             ....|....| ....|....| ....|....| ....|....| ....|....| ....|....|
                 2705       2715       2725       2735       2745       2755
2b_SalI      TTAGAAACGT ACACTGAAAA GAAGGATGAA GAAACTAAAG TGCTGCTAGA CAAGTTTAAC
2b_Thai      TTAGAAACGT ACACTGAAAA GAAGGATGAA GAAACTAAAG TGCTGCTAGA AAAGTTTAAC
Conservation ********** ********** ********** ********** ********** .*********

             ....|....| ....|....| ....|....| ....|....| ....|....| ....|....|
                 2765       2775       2785       2795       2805       2815
2b_SalI      ACCGAAGTGG AAAACTTCAA ACTAGATGAA GATGAAAAAT CATTTAACGA TGCCAAAAGT
2b_Thai      ACCGAAGTGG AAAACTTCAA ACTAGATGAA GATGAAAAAT CATTTAACGA TGCCAAAAGT
Conservation ********** ********** ********** ********** ********** **********

             ....|....| ....|....| ....|....| ....|....| ....|....| ....|....|
                 2825       2835       2845       2855       2865       2875
2b_SalI      ATTGTCAGTA ACACAATAAA TGAAGTCGAA AATGAAAACA AAAATATAGA CTCCATTAAG
2b_Thai      ATTGTCAGTA ACACAATAAA TGAAGTCGAA AATGAAAACA AAAATATAGA CTCCATTAAG
Conservation ********** ********** ********** ********** ********** **********

             ....|....| ....|....| ....|....| ....|....| ....|....| ....|....|
                 2885       2895       2905       2915       2925       2935
2b_SalI      AAAGTGAATA TTGCAATGAA ACGCTCCAGA AGTAACCAAC AATCCGTTGA ACAGCTAATC
2b_Thai      AAAGTGAATA TTGCAATGAA ACGCTCCAGA AGTAACCAAC AATCCGTTGA ACAGCTAATC
Conservation ********** ********** ********** ********** ********** **********

             ....|....| ....|....| ....|....| ....|....| ....|....| ....|....|
                 2945       2955       2965       2975       2985       2995
2b_SalI      AGAAATAAGG ATAATCTTCT AAAAGAAAAA ATGGAAAAAC ACTTTCAAAA AATAAACACA
2b_Thai      AGAAATAAGG ATAATCTTCT AAAAGAAAAA ATGGAAAAAC ACTTTCAAAA AATAAACACA
Conservation ********** ********** ********** ********** ********** **********
```

```
             ....|....| ....|....| ....|....| ....|....| ....|....| ....|....|
                 3005       3015       3025       3035       3045       3055
2b_SalI      GATGAAATAA TAGGACAAAG TGCCAAGGCA AACTTGCTAA ACAATCTAGA GAGCGAAAAA
2b_Thai      GATGAAATAA TAGGACAAAG TGCCAAGGCA AACTTGCTAA ACAATCTAGA GAGCGAAAAA
Conservation ********** ********** ********** ********** ********** **********

             ....|....| ....|....| ....|....| ....|....| ....|....| ....|....|
                 3065       3075       3085       3095       3105       3115
2b_SalI      GCTATCATAA ACGAACAGTT AAATGACGCT CATTTGAATG ACCTAAAATT GCAGATAGAA
2b_Thai      GCTATCATAA ACGAACAGTT AAATGACGCT CATTTGAATG ACCTAAAATT GCAGATAGAA
Conservation ********** ********** ********** ********** ********** **********

             ....|....| ....|....| ....|....| ....|....| ....|....| ....|....|
                 3125       3135       3145       3155       3165       3175
2b_SalI      AAAACTCTAA GTTATTGTGA AAAATCAAGG GAAAATATTA AAGGAAATAA TGGAACACAT
2b_Thai      AAAACTCTAA GTTATTGTGA AAAATCAAGG GAAAATATTA AAGGAAATAA TGGAACACAT
Conservation ********** ********** ********** ********** ********** **********

             ....|....| ....|....| ....|....| ....|....| ....|....| ....|....|
                 3185       3195       3205       3215       3225       3235
2b_SalI      CCAGAAGAAT TAAACGCAAG CGAAATTGAG TGGGAAGCTA CGAAAGGAAA AATAGAAACA
2b_Thai      CCAGAAGAAT TAAACGCAAG CGAAATTGAG TGGGAAGCTA CGAAAGGAAA AATAGAAACA
Conservation ********** ********** ********** ********** ********** **********

             ....|....| ....|....| ....|....| ....|....| ....|....| ....|....|
                 3245       3255       3265       3275       3285       3295
2b_SalI      CTAAACAGTA ATTATCAAGC GTTAAACAAA AACATGGATG ATATAATCAA TAAGCAGCAC
2b_Thai      CTAAACAGTA ATTATCAAGC GTTAAACAAA AACATGGATG ATATAATCAA TAAGCAGCAC
Conservation ********** ********** ********** ********** ********** **********

             ....|....| ....|....| ....|....| ....|....| ....|....| ....|....|
                 3305       3315       3325       3335       3345       3355
2b_SalI      GATGAAATGA TAGTGCTCAT TGATAGAGAT ATAAAGGAGA AGGGAAAAGA CATCGAGCTT
2b_Thai      GATGAAATGA TAGTGCTCAT TGATAGAGAT ATAAAGGAGA AGGGAAAAGA CATCGAGCTT
Conservation ********** ********** ********** ********** ********** **********

             ....|....| ....|....| ....|....| ....|....| ....|....| ....|....|
                 3365       3375       3385       3395       3405       3415
2b_SalI      AAAGTTGAGC AACGTATACA GGCGCTCGAA AAAATGAAAG CAAAATTGTC CTCATTTGAT
2b_Thai      AAAGTTGAGC AACGTATACA GGCGCTCGAA AAAATGAAAG CAAAATTGTC CTCATTTGAT
Conservation ********** ********** ********** ********** ********** **********

             ....|....| ....|....| ....|....| ....|....| ....|....| ....|....|
                 3425       3435       3445       3455       3465       3475
2b_SalI      ACCAATGAGG ATGTTAAAAA ATACCAAAGT GACATAAATA AAGAAGAACT CATAAAATTA
2b_Thai      ACCAATGAGG ATGTTAAAAA ATACCAAAGT GACATAAATA AAGAAGAACT CATAAAATTA
Conservation ********** ********** ********** ********** ********** **********

             ....|....| ....|....| ....|....| ....|....| ....|....| ....|....|
                 3485       3495       3505       3515       3525       3535
2b_SalI      AAAGAAATGT CCACTGCAGT CCTAAACAAA ATTGATCAAA ATAAAAATCA ATTGATTAAC
2b_Thai      AAAGAAATGT CCACTGCAGT CCTAAACAAA ATTGATCAAA ATAAAAATCA ATTGATTAAC
Conservation ********** ********** ********** ********** ********** **********

             ....|....| ....|....| ....|....| ....|....| ....|....| ....|....|
                 3545       3555       3565       3575       3585       3595
2b_SalI      GTAAAGACAA AATCCAGTGG CTACGTCGCA GAATCGAATG GAATAAAAAA TAAACAAACC
2b_Thai      GTAAAGACAA AATCCAGTGG CTACGTCGCA GAATCGAATG GAATAAAAAA TAAACAAACC
Conservation ********** ********** ********** ********** ********** **********
```

```
             ....|....| ....|....| ....|....| ....|....| ....|....| ....|....|
                 3605       3615       3625       3635       3645       3655
2b_SalI      GAACCGAGTC AAGAGAAAGA CAAGCAAATT GTGTACAACA CAAATAGGGG TGACATCGAA
2b_Thai      GAACCGAGTC AAGAGAAAGA CAAGCAAATT GTGTACAACA CAAATAGGGG TGACATCGAA
Conservation ********** ********** ********** ********** ********** **********

             ....|....| ....|....| ....|....| ....|....| ....|....| ....|....|
                 3665       3675       3685       3695       3705       3715
2b_SalI      AAAGTTTATA AAAACATTAG AGACATGTAC AACGAACTGG ATGATGTCGA AAAGGAAGAA
2b_Thai      AAAGTTTATA AAAACATTAG AGACATGTAC AACGAACTGG ATGATGTCGA AAAGGGAGAA
Conservation ********** ********** ********** ********** ********** *****.****

             ....|....| ....|....| ....|....| ....|....| ....|....| ....|....|
                 3725       3735       3745       3755       3765       3775
2b_SalI      ACCACCTCAA ACGAAGTAGA CAACATAGAA ATGCAGTATG ACAGACTTTT AATCAATTTG
2b_Thai      ACCACCTCAA ACGAAGTAGA CAACATAGAA ATGCAGTATG ACAGACTTTT AATCAATTTG
Conservation ********** ********** ********** ********** ********** **********

             ....|....| ....|....| ....|....| ....|....| ....|....| ....|....|
                 3785       3795       3805       3815       3825       3835
2b_SalI      CTTGTTCAAA AGATCAACGA AGAAAACACA AAGGCGAAGG GACTCATGGA AGAAATTAAA
2b_Thai      CTTGTTCAAA AGATCAACGA AGAAAACACA AAGGCGAAGG GACTCATGGA AGAAATTAAA
Conservation ********** ********** ********** ********** ********** **********

             ....|....| ....|....| ....|....| ....|....| ....|....| ....|....|
                 3845       3855       3865       3875       3885       3895
2b_SalI      TCATACATAG AAAATATTGA AAGTGTACAG GCCAAAATGT CAGATGAAAC GCGAGAAGGC
2b_Thai      TCATACATAG AAAATATTGA AAGTGTACAG GCCAAAATGT CAGATGAAAC GCGAGAAGGC
Conservation ********** ********** ********** ********** ********** **********

             ....|....| ....|....| ....|....| ....|....| ....|....| ....|....|
                 3905       3915       3925       3935       3945       3955
2b_SalI      GCAACTGAGT TTGAGTTTAA TCAATATCAG CAGAGCAGTT TAGCCAATGA TAAAAAAATT
2b_Thai      GCAACTGAGT TTGAGTTTAA TCAATATCAG CAGAGCAGTT TAGCCAATGA TAAAAAAATT
Conservation ********** ********** ********** ********** ********** **********

             ....|....| ....|....| ....|....| ....|....| ....|....| ....|....|
                 3965       3975       3985       3995       4005       4015
2b_SalI      ACCGAAATTG TTCAACATGC TATAAATAAG AAGGGAGAAG CAGAAAGAAG TAAAAAAACG
2b_Thai      ACCGAAATTG TTCAACATGC TATAAATAAG AAGGGAGAAG CAGAAAGAAG TAAAAAAACG
Conservation ********** ********** ********** ********** ********** **********

             ....|....| ....|....| ....|....| ....|....| ....|....| ....|....|
                 4025       4035       4045       4055       4065       4075
2b_SalI      AATGAAGTAA AATCCATCAA AAATGACGTT AACACTTACA TGCAACAAGT TATAGATGAA
2b_Thai      AATGAAGTAA AATCCATCAA AAATGACGTT AACACTTACA TGCAACAAGT TATAGATGAA
Conservation ********** ********** ********** ********** ********** **********

             ....|....| ....|....| ....|....| ....|....| ....|....| ....|....|
                 4085       4095       4105       4115       4125       4135
2b_SalI      TATAACGCTA TGCAAAAGGC GTTAGGGGAT ATTAAAAATG TGAACGATTT ATTAATATCA
2b_Thai      TATAACGCTA TGCAAAAGGC GTTAGGGGAT ATTAAAAATG TGAACGATTT ATTAATATCA
Conservation ********** ********** ********** ********** ********** **********

             ....|....| ....|....| ....|....| ....|....| ....|....| ....|....|
                 4145       4155       4165       4175       4185       4195
2b_SalI      ACCAGTGGTA GAAGAATCGC TGAAGAAATA TCGAAGAGCA CTAACGAAGC TGAGAATTTT
2b_Thai      ACCAGTGGTA GAAGAATCGC TGAAGAAATA TCGAAGAGCA CTAACGAAGC TGAGAATTTT
Conservation ********** ********** ********** ********** ********** **********
```

```
             ....|....| ....|....| ....|....| ....|....| ....|....| ....|....|
                  4205       4215       4225       4235       4245       4255
2b_SalI      GAAAAGCAAG CAAAAGGAGA ATTAGGAAAA ATAAATGATA TAATTGAAAA AATGGAAAAT
2b_Thai      GAAAAGCAAG CAAAAGGAGA ATTAGGAAAA ATAAATGATA TAATTGAAAA AATGGAAAAT
Conservation ********** ********** ********** ********** ********** **********

             ....|....| ....|....| ....|....| ....|....| ....|....| ....|....|
                  4265       4275       4285       4295       4305       4315
2b_SalI      CAAATGGTTC AGGCAAAGGA ACACGAAAAT AAAATTCATA TAAACTTAGA GGATAAACAA
2b_Thai      CAAATGGTTC AGGCAAAGGA ACACGAAAAT AAAATTCATA TAAACTTAGA GGATAAACAA
Conservation ********** ********** ********** ********** ********** **********

             ....|....| ....|....| ....|....| ....|....| ....|....| ....|....|
                  4325       4335       4345       4355       4365       4375
2b_SalI      ATAGATGAAG AAGTGCAAAA AATTAGACAA ATTCATGAAG AACTTATAAA TACGAAAATT
2b_Thai      ATAGATGAAG AAGTGCAAAA AATTAGACAA ATTCATGAAG AACTTATAAA TACGAAAATT
Conservation ********** ********** ********** ********** ********** **********

             ....|....| ....|....| ....|....| ....|....| ....|....| ....|....|
                  4385       4395       4405       4415       4425       4435
2b_SalI      GCAATGAATG AACATTTAAG TAAGACAAAA GCCGCTAAAG AAAATTGCCT TTCCCAAGTC
2b_Thai      GCAATGAATG AACATTTAAG TAAGACAAAA GCCGCTAAAG AAAATTGCCT TTCCCAAGTC
Conservation ********** ********** ********** ********** ********** **********

             ....|....| ....|....| ....|....| ....|....| ....|....| ....|....|
                  4445       4455       4465       4475       4485       4495
2b_SalI      CGTAATGCCG AGAGGGGGAA AATTAAAATA GATTTTCTCA ATAATAACAA ACAAAGTAGC
2b_Thai      CGTAATGCCG AGAGGGGGAA AATTAAAATA GATTTTCTCA ATAATAACAA ACAAAGTAGC
Conservation ********** ********** ********** ********** ********** **********

             ....|....| ....|....| ....|....| ....|....| ....|....| ....|....|
                  4505       4515       4525       4535       4545       4555
2b_SalI      AATTTGAAAG ACGTCAATAT GGATCATGTA ACTAAAAATA TTGCCGACTG TGTGGAACAC
2b_Thai      AATTTGAAAG ACGTCAATAT GGATCATGTA ACTAAAAATA TTGCCGACTG TGTGGAACAC
Conservation ********** ********** ********** ********** ********** **********

             ....|....| ....|....| ....|....| ....|....| ....|....| ....|....|
                  4565       4575       4585       4595       4605       4615
2b_SalI      TCAAAAGAAG TTAATAAAAC TGAAAACGAA GCTGAAAAGG AACATGACCT ATTCCTAGAA
2b_Thai      TCAAAAGAAG TTAATAAAAC TGAAAAAGAA GCTGAAAAGG AACATGACCT ATTCCTAGAA
Conservation ********** ********** ******.*** ********** ********** **********

             ....|....| ....|....| ....|....| ....|....| ....|....| ....|....|
                  4625       4635       4645       4655       4665       4675
2b_SalI      TATGAAAAAG ATATTACCAG CATATTAAAC AGATCCTCAA TTTTAGGAAT AGAAACAAAA
2b_Thai      TATGAAAAAG ATATTACCAG CATATTAAAC AGATCCTCAA TTTTAGGAAT AGAAACAAAA
Conservation ********** ********** ********** ********** ********** **********

             ....|....| ....|....| ....|....| ....|....| ....|....| ....|....|
                  4685       4695       4705       4715       4725       4735
2b_SalI      TCGAAAAAAA GAAAAAACGA AGCTACCAAA ATAATGGGCC AAATTGAGGG GGAACATTCT
2b_Thai      TCGAAAAAAA GAAAAAACGA AGCTACCAAA ATAATGGGCC AAATTGAGGG GGAACATTCT
Conservation ********** ********** ********** ********** ********** **********

             ....|....| ....|....| ....|....| ....|....| ....|....| ....|....|
                  4745       4755       4765       4775       4785       4795
2b_SalI      ATAATTAAAA CGGAACTTCA ACAATCTTCA GAAAATTTTA AACAACTGAA TGAAAAATAT
2b_Thai      ATAATTAAAA CGGAACTTCA ACAATCTTCA GAAAATTTTA AACAACTGAA TGAAAAATAT
Conservation ********** ********** ********** ********** ********** **********
```

```
             ....|....| ....|....| ....|....| ....|....| ....|....| ....|....|
                  4805       4815       4825       4835       4845       4855
2b_SalI      AACCTTAGCG AAGAAAAAAG TATGCTTGCC AATCCCACAT CCACCAAAGC AAATGAGTCC
2b_Thai      AACCTTAGCG AAGAAGAAAG TATGCTTGCC AATCCCACAT CCACCAAAGC AAATGAGTCC
Conservation ********** *****.**** ********** ********** ********** **********

             ....|....| ....|....| ....|....| ....|....| ....|....| ....|....|
                  4865       4875       4885       4895       4905       4915
2b_SalI      ATACAGTATA ACATAGGCAA GGTGAAACAA AATTTGTTAT ACATAGACAA TATAGCGAAA
2b_Thai      ATACAGTATA ACATAGGCAA GGTGAAACAA AATTTGTTAT ACATAGACAA TATAGCGAAA
Conservation ********** ********** ********** ********** ********** **********

             ....|....| ....|....| ....|....| ....|....| ....|....| ....|....|
                  4925       4935       4945       4955       4965       4975
2b_SalI      GAAGTAGACA CTATTTTAAC TAACGCACAA GATTCAATTA GATCAATATC GCAGATTTCC
2b_Thai      GAAGTAGACA CTATTTTAAC TAACGCACAA GATTCAATTA GATCAATATC GCAGATTTCC
Conservation ********** ********** ********** ********** ********** **********

             ....|....| ....|....| ....|....| ....|....| ....|....| ....|....|
                  4985       4995       5005       5015       5025       5035
2b_SalI      AAACTAGACG GAAGGAATAC ACTACAAATG GTGGAAAAGG AAGCAGCAGA TTATGTAAAA
2b_Thai      AAACTAGACG GAAGGAATAC ACTACAAATG GTGGAAAAGG AAGCAGCAGA TTATGTAAAA
Conservation ********** ********** ********** ********** ********** **********

             ....|....| ....|....| ....|....| ....|....| ....|....| ....|....|
                  5045       5055       5065       5075       5085       5095
2b_SalI      TATTTAGACA AAATAACCAA AGAGAAGGAA CTAATGAATG ATAAACACAA AGACCTCAAT
2b_Thai      TATTTAGACA AAATAACCAA AGAGAAGGAA CTAATGAATG ATAAACACAA AGACCTCAAT
Conservation ********** ********** ********** ********** ********** **********

             ....|....| ....|....| ....|....| ....|....| ....|....| ....|....|
                  5105       5115       5125       5135       5145       5155
2b_SalI      CCCATAAAGT CCGATATTGC AAATATAGAA ACAGAATTGA AAAAGCATAG GAAACTTTTC
2b_Thai      CCCATAAAGT CCGATATTGC AAATATAGAA ACAGAATTGA AAAAGCATAG GAAACTTTTC
Conservation ********** ********** ********** ********** ********** **********

             ....|....| ....|....| ....|....| ....|....| ....|....| ....|....|
                  5165       5175       5185       5195       5205       5215
2b_SalI      GAAATAGGAC TTTTGAATAA GGTTATCGAG ATAGCTAAAA CGAGAAAATT GTACATGGAT
2b_Thai      GAAATAGGAC TTTTGAATAA GGTTATCGAG ATAGCTAAAA CGAGAAAATT GTACATGGAT
Conservation ********** ********** ********** ********** ********** **********

             ....|....| ....|....| ....|....| ....|....| ....|....| ....|....|
                  5225       5235       5245       5255       5265       5275
2b_SalI      TCAGTGGAAC AGCTCCTAAA TTCCTCCATC AATAATTTTG TTACCCTCTT TAATGGGTTC
2b_Thai      TCAGTGGAAC AGCTCCTAAA TTCCTCCATC AATAATTTTG TTACCCTCTT TAATGGGTTC
Conservation ********** ********** ********** ********** ********** **********

             ....|....| ....|....| ....|....| ....|....| ....|....| ....|....|
                  5285       5295       5305       5315       5325       5335
2b_SalI      AATTTAAAAA AATATAAAAT TGAAACCAGT TTAGACGTTT ATAAAGGAAA AATAAGTGAA
2b_Thai      AATTTAAAAA AATATAAAAT TGAAACCAGT TTAGACGTTT ATAAAGGAAA AATAAGTGAA
Conservation ********** ********** ********** ********** ********** **********

             ....|....| ....|....| ....|....| ....|....| ....|....| ....|....|
                  5345       5355       5365       5375       5385       5395
2b_SalI      TTCCACACTG AATTCCTTCA GCCATACGAA TCAATTCAAA ATAAGGAAAA ACGCGTACTG
2b_Thai      TTCCACACTG AATTCCTTCA GCCATACGAA TCAATTCAAA ATAAGGAAAA ACGCGTACTG
Conservation ********** ********** ********** ********** ********** **********
```

```
             ....|....| ....|....| ....|....| ....|....| ....|....| ....|....|
                 5405       5415       5425       5435       5445       5455
2b_SalI      GACCCTTCGG TAGATTTTAG TGCAGCAAAA AATATGCGAG AAGAAGCTCA AAAGGAGGAA
2b_Thai      GACCCTTCGG TAGATTTTAG TGCAGCAAAA AATATGCGAG AAGAAGCTCA AAAGGAGGAA
Conservation ********** ********** ********** ********** ********** **********

             ....|....| ....|....| ....|....| ....|....| ....|....| ....|....|
                 5465       5475       5485       5495       5505       5515
2b_SalI      AAAAATATCG AAAATGTAGA AAAAAAGACA AAAAAATATC TAAGCGATAT TAACAAAATG
2b_Thai      AAAAATATCG AAAATGTAGA AAAAAAGACA AAAAAATATC TAAGCGATAT TAACAAAATG
Conservation ********** ********** ********** ********** ********** **********

             ....|....| ....|....| ....|....| ....|....| ....|....| ....|....|
                 5525       5535       5545       5555       5565       5575
2b_SalI      GAATCCTTTA CATTCATACG TAATATGAGA CAAACTTTGG ATGAACTAAG CAAAACATGC
2b_Thai      GAATCCTTTA CATTCATACG TAATATGAGA CAAACTTTGG ATGAACTAAG CAAAACATGC
Conservation ********** ********** ********** ********** ********** **********

             ....|....| ....|....| ....|....| ....|....| ....|....| ....|....|
                 5585       5595       5605       5615       5625       5635
2b_SalI      AAACAGGAAT ACGCAGAAGT CGAGGGAGGT CACAAAGACA TTGAAAAAAT CATAGAAGAT
2b_Thai      AAACAGGAAT ACGCAGAAGT CGAGGGAGGT CACAAAGACA TTGAAAAAAT CATAGAAGAT
Conservation ********** ********** ********** ********** ********** **********

             ....|....| ....|....| ....|....| ....|....| ....|....| ....|....|
                 5645       5655       5665       5675       5685       5695
2b_SalI      ATTAAGAGCC TAAACGATGA AAGTGGCTCG TCAGCTAAAT TACAACAAGC AGAGGATAAA
2b_Thai      ATTAAGAGCC TAAACGATGA AAGTGGCTCG TCAGCTAAAT TACAACAAGC AGAGGATAAA
Conservation ********** ********** ********** ********** ********** **********

             ....|....| ....|....| ....|....| ....|....| ....|....| ....|....|
                 5705       5715       5725       5735       5745       5755
2b_SalI      AATAGCCAAA TGAAAGCCAA AGTGAATCAT TATTCACTTA AGAAAGAGGC ACACACTATA
2b_Thai      AATAGCCAAA TGAAAGCCAA AGTGAATCAT TATTCACTTA AGAAAGAGGC ACACACTATA
Conservation ********** ********** ********** ********** ********** **********

             ....|....| ....|....| ....|....| ....|....| ....|....| ....|....|
                 5765       5775       5785       5795       5805       5815
2b_SalI      TTAGAACAAA TTGTCAAATC AGCCAATTTT GTAGGCATTA CAATAGTGAC AGCGTTACAG
2b_Thai      TTAGAACAAA TTGTCAAATC AGCCAATTTT GTAGGCATTA CAATAGTGAC AGCGTTACAG
Conservation ********** ********** ********** ********** ********** **********

             ....|....| ....|....| ....|....| ....|....| ....|....| ....|....|
                 5825       5835       5845       5855       5865       5875
2b_SalI      CCATCAGATT TAGCTGCTAA AGCGAGTTTG CAAAAAGCAC CAGAATTGCA GTTTGAATCC
2b_Thai      CCATCAGATT TAGCTGCTAA AGCGAGTTTG CAAAAAGCAC CAGAATTGCA GTTTGAATCC
Conservation ********** ********** ********** ********** ********** **********

             ....|....| ....|....| ....|....| ....|....| ....|....| ....|....|
                 5885       5895       5905       5915       5925       5935
2b_SalI      CAATCCAAAG TAACACTAGA AAGCGGACAA TTCTTAGAAA AAAAAAACCA ATTGGATGTC
2b_Thai      CAATCCAAAG TAACACTAGA AAGCGGACAA TTCTTAGAAA AAAAAAACCA ATTGGATGTC
Conservation ********** ********** ********** ********** ********** **********

             ....|....| ....|....| ....|....| ....|....| ....|....| ....|....|
                 5945       5955       5965       5975       5985       5995
2b_SalI      CACGAAAATG TGCAAGCCGC CTACAATACC GTTCTTCAGA TATACAAATA TTCAGATGAT
2b_Thai      CACGAAAATG TGCAAGCCGC CTACAATACC GTTCTTCAGA TATACAAATA TTCAGATGAT
Conservation ********** ********** ********** ********** ********** **********
```

```
                ....|....| ....|....| ....|....| ....|....| ....|....| ....|....|
                     6005       6015       6025       6035       6045       6055
2b_SalI         ATAGTAAGGA AGCAGGAAAA ATGCGAACAA TTAGTAAAAG ACGGAAAAGA TATATGCCTT
2b_Thai         ATAGTAAGGA AGCAGGAAAA ATGCGAACAA TTAGTAAAAG ACGGAAAAGA TATATGCCTT
Conservation    ********** ********** ********** ********** ********** **********

                ....|....| ....|....| ....|....| ....|....| ....|....| ....|....|
                     6065       6075       6085       6095       6105       6115
2b_SalI         AAATTCAAAT CAATAAACGA AATTAAAGTT ATGATTCAAA ATAGTAAGGG AAAAGAAAGC
2b_Thai         AAATTCAAAT CAATAAACGA AATTAAAGTT ATGATTCAAA ATAGTAAGGG AAAAGAAAGC
Conservation    ********** ********** ********** ********** ********** **********

                ....|....| ....|....| ....|....| ....|....| ....|....| ....|....|
                     6125       6135       6145       6155       6165       6175
2b_SalI         ACCCTTTCAG CCAAAGTGAG TCACTCCTTT AACAAGCTAA GTGAGCTAAA CAAAATTAAG
2b_Thai         ACCCTTTCAG CCAAAGTGAG TCACTCCTTT AACAAGCTAA GTGAGCTAAA CAAAATTAAG
Conservation    ********** ********** ********** ********** ********** **********

                ....|....| ....|....| ....|....| ....|....| ....|....| ....|....|
                     6185       6195       6205       6215       6225       6235
2b_SalI         TGCAATGATG AAAGTTACGA TGCCATTTTG GAAACACCCA GTAGGGAAGA ACTAAACAAA
2b_Thai         TGCAATGATG AAAGTTACGA TGCCATTTTG GAAACACCCA GTAGGGAAGA ACTAAACAAA
Conservation    ********** ********** ********** ********** ********** **********

                ....|....| ....|....| ....|....| ....|....| ....|....| ....|....|
                     6245       6255       6265       6275       6285       6295
2b_SalI         CTAAGGAGCA CATTTAAACA AGAGAAAGAC ACCATCGCAA ACCAAGCAAA ATTGAGTGGC
2b_Thai         CTAAGGAGCA CATTTAAACA AGAGAAAGAC ACCATCGCAA ACCAAGCAAA ATTGAGTGGC
Conservation    ********** ********** ********** ********** ********** **********

                ....|....| ....|....| ....|....| ....|....| ....|....| ....|....|
                     6305       6315       6325       6335       6345       6355
2b_SalI         TATAAAACAG ATTTCGAAAC TCACATTGGA AAATTAAATG ACTTAGCCAA AATAGTGGAC
2b_Thai         TATAAAACAG ATTTCGAAAC TCACATTGGA AAATTAAATG ACTTAGCCAA AATAGTGGAC
Conservation    ********** ********** ********** ********** ********** **********

                ....|....| ....|....| ....|....| ....|....| ....|....| ....|....|
                     6365       6375       6385       6395       6405       6415
2b_SalI         AACTTAAAAG CCAGCGAAAC TCTTCCAAAA AATATAGAAG AAAAAAAAAC GTCCATCAAC
2b_Thai         AACTTAAAAG CCAGCGAAAC TCTTCCAAAA AATATAGAAG AAAAAAAAAC GTCCATCAAC
Conservation    ********** ********** ********** ********** ********** **********

                ....|....| ....|....| ....|....| ....|....| ....|....| ....|....|
                     6425       6435       6445       6455       6465       6475
2b_SalI         CTAATTTCAA CCAAATTGGA AACCATCGAA AAGGAAATTG AAAGTATTAA TTCCTCCTTT
2b_Thai         CTAATTTCAA CCAAATTGGA AACCATCGAA AAGGAAATTG AAAGTATTAA TTCCTCCTTT
Conservation    ********** ********** ********** ********** ********** **********

                ....|....| ....|....| ....|....| ....|....| ....|....| ....|....|
                     6485       6495       6505       6515       6525       6535
2b_SalI         GATCAGTTGC TCGAGAAAGG AAAGAAGTGC GAAATGACGA AGTACAAATT GGTAAGGGAT
2b_Thai         GATCAGTTGC TCGAGAAAGG AAAGAAGTGC GAAATGACGA AGTACAAATT GGTAAGGGAT
Conservation    ********** ********** ********** ********** ********** **********

                ....|....| ....|....| ....|....| ....|....| ....|....| ....|....|
                     6545       6555       6565       6575       6585       6595
2b_SalI         AGCCTAAGCA CTAAAATAAA TGACCATTCT GCAATCATTA AGGACAACCA AAAAAAAGCC
2b_Thai         AGCCTAAGCA CTAAAATAAA TGACCATTCT GCAATCATTA AGGACAACCA AAAAAAAGCC
Conservation    ********** ********** ********** ********** ********** **********
```

```
             ....|....| ....|....| ....|....| ....|....| ....|....| ....|....|
                 6605       6615       6625       6635       6645       6655
2b_SalI      ACTGAATATT TAACGTACAT TCAAAATAAT CACATTTCCA TATTTAAAGA CATTGATATG
2b_Thai      ACTGAATATT TAACGTACAT TCAAAATAAT CACATTTCCA TATTTAAAGA CATTGATATG
Conservation ********** ********** ********** ********** ********** **********

             ....|....| ....|....| ....|....| ....|....| ....|....| ....|....|
                 6665       6675       6685       6695       6705       6715
2b_SalI      CTAAATGAAA ACCTGGGTGA AAAATCCGTA AGTAGGTATG CCATAGCCAA AATCGAAGAA
2b_Thai      ATAAATGAAA ACCTGGGTGA AAAATCCGTA AGTAGGTATG CCATAGCCAA AATCGAAGAA
Conservation .********* ********** ********** ********** ********** **********

             ....|....| ....|....| ....|....| ....|....| ....|....| ....|....|
                 6725       6735       6745       6755       6765       6775
2b_SalI      GCAAATGATC TTTCCGCACA ACTAACCGCT GCAGTTAGTG AATACGAAGC GATAGCTAAT
2b_Thai      GCAAATGATC TTTCCGCACA GCTAACCGCT GCAGTTAGTG AATACGAAGC GATAGCTAAT
Conservation ********** ********** .********* ********** ********** **********

             ....|....| ....|....| ....|....| ....|....| ....|....| ....|....|
                 6785       6795       6805       6815       6825       6835
2b_SalI      TCTATTAGAA AAGAATTTAC AAATATAAGT GACCACACCG AAATGGACAC TCTTGAAAAT
2b_Thai      TTTATTAGAA AAAAATTTAC AAATATAAGT GACCACACCG AAATGGACAC TCTTGAAAAT
Conservation * ******** **.******* ********** ********** ********** **********

             ....|....| ....|....| ....|....| ....|....| ....|....| ....|....|
                 6845       6855       6865       6875       6885       6895
2b_SalI      GAGGCAAAAA TGTTGAAAGA ACATTATGAC AATCTAATAA ATAAAAAAAA CATCATAACA
2b_Thai      GAGGCAAAAA TGTTGAAAGA ACATTATGAC AATCTAATAA ATAAAAAAAA CATCATAACA
Conservation ********** ********** ********** ********** ********** **********

             ....|....| ....|....| ....|....| ....|....| ....|....| ....|....|
                 6905       6915       6925       6935       6945       6955
2b_SalI      GAATTACATA ACAAAATTAA TTTAATTAAA CTGCTTGAAA TAAGAGCCAC CTCTGATAAA
2b_Thai      GAATTACATA ACAAAATTAA TTTAATTAAA CTGCTTGAAA TAAGAGCCAC CTTTGATAAA
Conservation ********** ********** ********** ********** ********** ** *******

             ....|....| ....|....| ....|....| ....|....| ....|....| ....|....|
                 6965       6975       6985       6995       7005       7015
2b_SalI      TACGTCGATA TAGCTGAACT GCTTGGCGAA GTAGTAAAGG ATCAAAAAAA AAAGTTACAA
2b_Thai      TACGTCGATA TAGCTGAACT GCTTGGCGAA GTAGTAAAGG ATCAAAAAAA AAAGTTACAA
Conservation ********** ********** ********** ********** ********** **********

             ....|....| ....|....| ....|....| ....|....| ....|....| ....|....|
                 7025       7035       7045       7055       7065       7075
2b_SalI      GAAGCAAAAA ATAAACTGGA CACGCTTAAA GACCATATAG CAGTTAAAGA AAAGGAGTTA
2b_Thai      GAAGCAAAAA ATAAACTGGA CACGCTTAAA GACCATATAG CAGTTAAAGA AAAGGAGTTA
Conservation ********** ********** ********** ********** ********** **********

             ....|....| ....|....| ....|....| ....|....| ....|....| ....|....|
                 7085       7095       7105       7115       7125       7135
2b_SalI      ATCAACCATG ACAGCTCCTT CACCCTAGTG TCCATTAAAG CGTTCGACGA AATATATGAT
2b_Thai      ATCAACCATG ACAGCTCCTT CACCCTAGTG TCCATTAAAG CGTTCGACGA AATATATGAT
Conservation ********** ********** ********** ********** ********** **********

             ....|....| ....|....| ....|....| ....|....| ....|....| ....|....|
                 7145       7155       7165       7175       7185       7195
2b_SalI      GACATTAAAT ATAATGTAGG CCAATTGCAC ACATTAGAAG TAACTAACTT TGATGAACTT
2b_Thai      GACATTAAAT ATAATGTAGG CCAATTGCAC ACATTAGAAG TAACTAACTT TGATGAACTT
Conservation ********** ********** ********** ********** ********** **********
```

```
               ....|....| ....|....| ....|....| ....|....| ....|....| ....|....|
                    7205       7215       7225       7235       7245       7255
2b_SalI        AAAAAAGGTA AAACGTATGA AGAAAATGTT ACCCATTTAT TAAACAGAAG GGAAACCTTA
2b_Thai        AAAAAAGGTA AAACGTATGA AGAAAATGTT ACCCATTTAT TAAACAGAAG GGAAACCTTA
Conservation   ********** ********** ********** ********** ********** **********

               ....|....| ....|....| ....|....| ....|....| ....|....| ....|....|
                    7265       7275       7285       7295       7305       7315
2b_SalI        CAGAATGATC TCCACAATTA TGAAGAAAAA GACAAACTGA AAAATACAAA TATAGAAATG
2b_Thai        CAGAATGATC TCCACAATTA TGAAGAAAAA GACAAACTGA AAAATACAAA TATAGAAATG
Conservation   ********** ********** ********** ********** ********** **********

               ....|....| ....|....| ....|....| ....|....| ....|....| ....|....|
                    7325       7335       7345       7355       7365       7375
2b_SalI        TCAAATGAAG AAAACAACCA AATAAGACAA ACCAGTGAAG TAATAAAAAA ATTAGAAAGC
2b_Thai        TCAAATGAAG AAAACAACCA AATAAGACAA ACCAGTGAAG TAATAAAAAA ATTAGAAAGC
Conservation   ********** ********** ********** ********** ********** **********

               ....|....| ....|....| ....|....| ....|....| ....|....| ....|....|
                    7385       7395       7405       7415       7425       7435
2b_SalI        GAATTTCAAA ACTTGCTAAA AATTATTCAA CAAAGTAACA CGCTATGCAG TAATGATAAT
2b_Thai        GAATTTCAAA ACTTGCTAAA AATTATTCAA CAAAGTAACA CGCTATGCAG TAATGATAAT
Conservation   ********** ********** ********** ********** ********** **********

               ....|....| ....|....| ....|....| ....|....| ....|....| ....|....|
                    7445       7455       7465       7475       7485       7495
2b_SalI        ATAAAACAGT TCATTTCTGA TATTCTAAAA AAAGTGGAAA CTATTAGAGA AAGGTTTGTC
2b_Thai        ATAAAACAGT TCATTTCTGA TATTCTAAAA AAAGTGGAAA CTATTAGAGA AAGGTTTGTC
Conservation   ********** ********** ********** ********** ********** **********

               ....|....| ....|....| ....|....| ....|....| ....|....| ....|....|
                    7505       7515       7525       7535       7545       7555
2b_SalI        AAGAATTTCC CCGAGAGAGA GAAGTACCAT CAAATAGAAA TCAACTACAA CGAAATTAAA
2b_Thai        AAGAATTTCC CCGAGAGAGA GAAGTACCAT CAAATAGAAA TCAACTACAA CGAAATTAAA
Conservation   ********** ********** ********** ********** ********** **********

               ....|....| ....|....| ....|....| ....|....| ....|....| ....|....|
                    7565       7575       7585       7595       7605       7615
2b_SalI        GGCATTGTTA AAGAAGTAGA TACAAATCCA GAAATAAGCA TATTTACTGA AAAAATAAAT
2b_Thai        GGCATTGTTA AAGAAGTAGA TACAAATCCA GAAATAAGCA TATTTACTGA AAAAATAAAT
Conservation   ********** ********** ********** ********** ********** **********

               ....|....| ....|....| ....|....| ....|....| ....|....| ....|....|
                    7625       7635       7645       7655       7665       7675
2b_SalI        ACATATATAC GGCAAAAAAT TAGAAGCGCT CACCATTTGG AAGATGCCCA AAAAATAAAA
2b_Thai        ACATATATAC GGCAAAAAAT TAGAAGCGCT CACCATTTGG AAGATGCCCA AAAAATAAAA
Conservation   ********** ********** ********** ********** ********** **********

               ....|....| ....|....| ....|....| ....|....| ....|....| ....|....|
                    7685       7695       7705       7715       7725       7735
2b_SalI        GACATAATAG AAGATGTTAC AAGTAACTAT AGAAAAAATAA AAAGTAAACT CTCTCAAGTT
2b_Thai        GACATAATAG AAGATGTTAC AAGTAACTAT AGAAAAAATAA AAAGTAAACT CTCTCAAGTT
Conservation   ********** ********** ********** ********** ********** **********

               ....|....| ....|....| ....|....| ....|....| ....|....| ....|....|
                    7745       7755       7765       7775       7785       7795
2b_SalI        AATAACGCTT TAGATAGAAT TAAAATAAAG AAAAGTGAGA TGGATACCCT ATTTGAATCA
2b_Thai        AATAACGCTT TAGATAGAAT TAAAATAAAG AAAAGTGAGA TGGATACCCT ATTTGAATCA
Conservation   ********** ********** ********** ********** ********** **********
```

```
             ....|....| ....|....| ....|....| ....|....| ....|....| ....|....|
                 7805       7815       7825       7835       7845       7855
2b_SalI      TTGTCCAAAG AAAATGCCAA TAATTATAAT TCCGCAAAAT ATTTTTTAGT CGATTCGGAT
2b_Thai      TTGTCCAAAG AAAATGCCAA TAATTATAAT TCCGCAAAAT ATTTTTTAGT CGATTCGGAT
Conservation ********** ********** ********** ********** ********** **********

             ....|....| ....|....| ....|....| ....|....| ....|....| ....|....|
                 7865       7875       7885       7895       7905       7915
2b_SalI      AAAATAATTA AGCATTTAGA AGACCAAGTT AGCAAAATGT CAAGCTTAAT AAGTTACGCC
2b_Thai      AAAATAATTA AGCATTTAGA AGACCAAGTT AGCAAAATGT CAAGCTTAAT AAGTTACGCC
Conservation ********** ********** ********** ********** ********** **********

             ....|....| ....|....| ....|....| ....|....| ....|....| ....|....|
                 7925       7935       7945       7955       7965       7975
2b_SalI      GAAAGGGAAA TAAAAGAATT AGAAGAAAAA GTGTATAGCA TATTAAATCG ACCTGTTATT
2b_Thai      GAAAGGGAAA TAAAAGAATT AGAAGAAAAA GTGTATAGCA TATTAAATCG ACCTGTTATT
Conservation ********** ********** ********** ********** ********** **********

             ....|....| ....|....| ....|....| ....|....| ....|....| ....|....|
                 7985       7995       8005       8015       8025       8035
2b_SalI      GATAACTCCG CTTCGGAGCA AATCAAACTG TTAAGTGATA CCCAAGAGAA CGATACAGAC
2b_Thai      GATAACTCCG CTTCGGAGCA AATCAAACTG TTAAGTGATA CCCAAGAGAA CGATACAGAC
Conservation ********** ********** ********** ********** ********** **********

             ....|....| ....|....| ....|....| ....|....| ....|....| ....|....|
                 8045       8055       8065       8075       8085       8095
2b_SalI      GAAGCTCAAA AACGGGAATT TGCTGAAGAA CAAATTCTGA ATTTACCTAA ACAACAGATA
2b_Thai      GAAGCTCAAA AACGGGAATT TGCTGAAGAA CAAATTCTGA ATTTACCTAA ACAACAGATA
Conservation ********** ********** ********** ********** ********** **********

             ....|....| ....|....| ....|....| ....|....| ....|....| ....|....|
                 8105       8115       8125       8135       8145       8155
2b_SalI      CATTCAGATG AGGGCAACAA TAATGATATG AGCACTACAG ATAGTGCAAA TGAAGAGAAA
2b_Thai      CATTCAGATG AGGGCAACAA TAATGATATG AGCACTACAG ATAGTGCAAA TGAAGAGAAA
Conservation ********** ********** ********** ********** ********** **********

             ....|....| ....|....| ....|....| ....|....| ....|....| ....|....|
                 8165       8175       8185       8195       8205       8215
2b_SalI      CAAACTGGAG AGGAAGAATC ACAACATGAG GATCAGTCAC AACCAAGGGG GATACATAGT
2b_Thai      CAAACTGGAG AGGAAGAATC ACAACATGAG GATCAGTCAC AACCAAGGGG GATACATAGT
Conservation ********** ********** ********** ********** ********** **********

             ....|....| ....|....| ....|....| ....|....| ....|....| ....|....|
                 8225       8235       8245       8255       8265       8275
2b_SalI      CGAGTTCGCC TTGCAGAAGG AATTATTTTG GGATTATCTG TCTTTTCAGG TATTATTTTT
2b_Thai      CGAGTTCGCC TTGCAGAAGG AATTATTTTG GGATTATCTG TCTTTTCAGG TATTATTTTT
Conservation ********** ********** ********** ********** ********** **********

             ....|....| ....|....| ....|....| ....|....| ....|....| ....|....|
                 8285       8295       8305       8315       8325       8335
2b_SalI      ATCTCATTCA GGCATAAGGA TGAGGAAAAG AAAGACCCTG AATCCGTCGG GGAACAATTT
2b_Thai      ATCTCATTCA GGCATAAGGA TGAGGAAAAG AAAGACCCTG AATCCGTCGG GGAACAATTT
Conservation ********** ********** ********** ********** ********** **********

             ....|....| ....|....| ....|....| ....|....| ....|....| ....|....|
                 8345       8355       8365       8375       8385       8395
2b_SalI      GAAGGTAATA ACAATTTTAA TGAAGACGAT AAGGAAGAAG TCATAGACGT TTGCTTCGAC
2b_Thai      GAAGGTAATA ACAATTTTAA TGAAGACGAT AAGGAAGAAG TCATAGACGT TTGCTTCGAC
Conservation ********** ********** ********** ********** ********** **********
```

```
             ....|....| ....|....| .
                8405       8415
2b_SalI      AATAGCGATT ATTCTGAATA G
2b_Thai      AATAGCGATT ATTCTGAATA G
Conservation ********** ********** *
```

Alignment: Pvrbp2c

```
             ....|....| ....|....| ....|....| ....|....| ....|....| ....|....|
                   5         15         25         35         45         55
2c_SalI      ATGGAAAAAA ATGTTCTTTG GGTAATCTTT TACAATTTCC TAGTTATTCT TTTGGCATCA
2c_Thai      ATGGAAAAAA ATGTTCTTTG GGTAATCTTT TACAATTTCC TAGTTATTCT TTTGGCATCA
Conservation ********** ********** ********** ********** ********** **********

             ....|....| ....|....| ....|....| ....|....| ....|....| ....|....|
                  65         75         85         95        105        115
2c_SalI      TGTAAAGATT CTAATAGGAG CAAATCTAAC AGTCTAAAAA GTGAAAGTAA GTCGTTACCG
2c_Thai      TGTAATGATT CTAATCGGAG CAAATCTAAC AGTCTAAAAA GTGAAAGTAA GTCGTTACCG
Conservation *****:**** *****.**** ********** ********** ********** **********

             ....|....| ....|....| ....|....| ....|....| ....|....| ....|....|
                 125        135        145        155        165        175
2c_SalI      ACATATGCTA ATTTAATGAG GAATGGTCAA CACAAATACA ACAATGCTAA AACGGAAGAT
2c_Thai      ACATATGCTA ATTTAATGAG GAATGGTCAA GACAAATACA ACAATGCTAA AACGGAAGAT
Conservation ********** ********** ********** .********* ********** **********

             ....|....| ....|....| ....|....| ....|....| ....|....| ....|....|
                 185        195        205        215        225        235
2c_SalI      AATATAGGTA ATCAGATCAA CAATGATAAC AACCATAATG GGTATAATGA TAACAGAACT
2c_Thai      AATATAGGTA ATCAGATCAA CAATGACAAC AACCATAACG GATATAATGA TAACAGAATT
Conservation ********** ********** ****** *** ******** * *.******** ******** *

             ....|....| ....|....| ....|....| ....|....| ....|....| ....|....|
                 245        255        265        275        285        295
2c_SalI      AATAGTGAAT ATCCAAAAAC ATCACATCTA CAGCATAGCC CGTCTCTGGT ACATTTAAAT
2c_Thai      AATAGTGAAT ATCCCAAAAC ATCACATCTA CAGCATAGTC CGTCTTTGGT ACATTTAAAT
Conservation ********** ****.***** ********** ******** * ***** **** **********

             ....|....| ....|....| ....|....| ....|....| ....|....| ....|....|
                 305        315        325        335        345        355
2c_SalI      GATCATAAAT TCACGGCAAA ACCATCTCGA CATGCTTACA TACAAAGAAA CAGCATATAT
2c_Thai      GATCATAAAT TTACGACAAA ACCATCTCGA CATTCTTACG TACAAAGAAA CAGCATATAT
Conservation ********** * ***.**** ********** *** *****. ********** **********

             ....|....| ....|....| ....|....| ....|....| ....|....| ....|....|
                 365        375        385        395        405        415
2c_SalI      ACACTTAACA CAAATAATAA TATGGAAAAT ACAAATAACG AATCGCATAC GGTTCCAAAT
2c_Thai      AAACGTAACA CAAATAATAA TATGGAAAAT ACAAATAACG AATTGCATGT TGTTCCAAAT
Conservation *.** ***** ********** ********** ********** *** ****.    *********

             ....|....| ....|....| ....|....| ....|....| ....|....| ....|....|
                 425        435        445        455        465        475
2c_SalI      TTATTTATTC AAAAAAATCA AAAAGCCCCC CAAAAGCCAG CTACCCAAAA AAAGCCAACG
2c_Thai      TTTTTTATTC AAGAAAAAAA AGCAGCCCCC CAAAAGCCAT CTGTCCAAAA TACACCAACG
Conservation **:******* **.****:.* *..******* *********   **. ****** .*..******

             ....|....| ....|....| ....|....| ....|....| ....|....| ....|....|
                 485        495        505        515        525        535
2c_SalI      GTCCAAAAAA AAACAGAGGC CCCTGAAATT GTATATGGTA ATTTGGACTA CCTTAATACC
2c_Thai      ---------- --------GC CACTCAAATT GTATATAATA ATTTGGACTA CCTCAATGCC
Conservation                    ** *.** ***** ******..** ********** *** ***.**

             ....|....| ....|....| ....|....| ....|....| ....|....| ....|....|
                 545        555        565        575        585        595
2c_SalI      TTCGATGACA CTAATAATAT AATTTCTGCG TTTAAACCTC ACCACCCAAT TATATATTAC
2c_Thai      TTCGATGACA CTAATAATAT AATTTCTGCG TTTAAACCTC ACCACCCAAT TATATATTAC
Conservation ********** ********** ********** ********** ********** **********
```

```
             ....|....| ....|....| ....|....| ....|....| ....|....| ....|....|
                   605        615        625        635        645        655
2c_SalI      TTCAAGGAAA TGGAACATTT TGCAAATAGT TACTATGACC TGAGGAGTAA AATTAGGGAT
2c_Thai      TTCAAGGAAT TGGAACATTT TGCAAATAGT TACTATGACC TGAGGAGTAA AATTAAGGAT
Conservation *********: ********** ********** ********** ********** *****.****

             ....|....| ....|....| ....|....| ....|....| ....|....| ....|....|
                   665        675        685        695        705        715
2c_SalI      TATTTTGCTC TTCCGATGAA ACAGGCTTTC GATGTCGTAG AGCAAAATGT AAAAGACTGT
2c_Thai      TATTTTGCTC TTCCGATGAA ACAGGCTTCC GATGTGGTAG AAAAAAATGT AAAAGACTGT
Conservation ********** ********** ******** * ***** **** *..******* **********

             ....|....| ....|....| ....|....| ....|....| ....|....| ....|....|
                   725        735        745        755        765        775
2c_SalI      TTGAAGAACA TAAATGAATT GAGAACATTA ATGACTCAGT TAGAAAATCC CCAAAACTAT
2c_Thai      TTGCAGAACA TAAATGAATC GAGAATATTA ATGACTCAGT TAGAAAATCC CCAAAACTAT
Conservation ***.****** *********  ***** **** ********** ********** **********

             ....|....| ....|....| ....|....| ....|....| ....|....| ....|....|
                   785        795        805        815        825        835
2c_SalI      AATGATATAA GTAATCAATA TGCTGAAAAA GTAAAAGAAT ACAAAAAGGA AATTGAGGAT
2c_Thai      AATGATATAA GTGATAAATA TGATGAAAAA GTAAAAGAAT ACAAAAAGAA AATTGAGGAT
Conservation ********** **.**.**** **.******* ********** ********.* **********

             ....|....| ....|....| ....|....| ....|....| ....|....| ....|....|
                   845        855        865        875        885        895
2c_SalI      ATGCAAAATT GTTTAAAAGA CAATTATAAA CAAAATTTTA AAGCTATCTT ATCTGCAAAA
2c_Thai      ATGCAAATTT GTTTAAAAGA CAGTTATATA AAAAATTTTA AAGCTATCAT GTCTGCAAAT
Conservation *******:** ********** **.*****:* .********* ********:* .********:

             ....|....| ....|....| ....|....| ....|....| ....|....| ....|....|
                   905        915        925        935        945        955
2c_SalI      TTAAAAATGA ACCTCGCTTT AAAAGATATA TATGCTAGCT GGTGGTCCCA ATTAAGTTCG
2c_Thai      TTAAAAATGA ATCTAGCTTT AAACGGAATA TATATCCACT GGTGGTACCT AACATGTTCG
Conservation ********** * **.***** ***.*..*** ***.....** ******.**. *..*.*****

             ....|....| ....|....| ....|....| ....|....| ....|....| ....|....|
                   965        975        985        995       1005       1015
2c_SalI      ACGGATACTT ACTATGATAT GGTTAAAAGA TATGTATTAG AAGTTAACAA ATTTGATAAA
2c_Thai      ACGAAAACTT ACGATGATAT AGTTAAAGAA TATGCAATAG AGATTAACGA TTTTGATGAA
Conservation ***.*.**** **.******* .******..* ****.*.*** *..*****.* .******.**

             ....|....| ....|....| ....|....| ....|....| ....|....| ....|....|
                  1025       1035       1045       1055       1065       1075
2c_SalI      AACACATCAA CTTCCTTTAT GGATCATATA AAGAAAATAC ATAATTCTGC TATTGATACA
2c_Thai      AAAAAATCGA TTTCTTTTAT GGATAATATG AAGAAAATAC ATAAGTCTGC TATTGATACA
Conservation **.*.***.* .*** ***** ****.****. ********** **** ***** **********

             ....|....| ....|....| ....|....| ....|....| ....|....| ....|....|
                  1085       1095       1105       1115       1125       1135
2c_SalI      ATGAAGAAAA TGAAAGAAGA ATTAAATACA AGTCTGGATT CTGACAGAGT AGAATTTATA
2c_Thai      TTGAAGAAAA TGAAAGCAGA ATTAAATACA AGTCTGGATT CTAAAAGAAC AGAATTTATA
Conservation .********* ******.*** ********** ********** **.*.***.. **********

             ....|....| ....|....| ....|....| ....|....| ....|....| ....|....|
                  1145       1155       1165       1175       1185       1195
2c_SalI      ATAGAAGAAA TTGGACATAT GGTAGAAAAG TTTAATCTTC ACTTGTCTAA AATGCGTTAT
2c_Thai      ATAGGAGAAA TTGGACATAT GATAGAAAAG TTTAATCTTC ATTTGACTAA AATTCGTTAT
Conservation ****.***** ********** *.******** ********** *.***.**** ***.******
```

```
             ....|....| ....|....| ....|....| ....|....| ....|....| ....|....|
                 1205       1215       1225       1235       1245       1255
2c_SalI      GGAGCTGATT ATATCAAAAA TATTGATTCA CAAAAGATAG AAAGTTACGT TTATCAAGTT
2c_Thai      GCATCTGCTT TTATCAAAAG TATTCCTTTA CAAAAGGTAG AAAGTGACAT TTATCGAGTT
Conservation *.*.***.** .********. ****..**.* ******.*** ***** **.* *****.****

             ....|....| ....|....| ....|....| ....|....| ....|....| ....|....|
                 1265       1275       1285       1295       1305       1315
2c_SalI      GAACTAAGAA CTCTTTTTTA TGTGGCAGCT AAACACTACG CTGATTTTAA GTTTAGTTTG
2c_Thai      GAACTAAAAA CTCTTTTTTA TGTGGCAGCT AAACACTACG CTGATTTTAA ATTTAGTTTG
Conservation *******.** ********** ********** ********** ********** .*********

             ....|....| ....|....| ....|....| ....|....| ....|....| ....|....|
                 1325       1335       1345       1355       1365       1375
2c_SalI      GAACATTTAA AAATGTTTGA AAATTTATCC AAAAGCAAAG AAAAAATGCT TTATAGCACT
2c_Thai      GAACATTTAA AAATGTTTGA AAATTTATCC AAAAGCAAAG AAAAAATGCT TTATAGCACT
Conservation ********** ********** ********** ********** ********** **********

             ....|....| ....|....| ....|....| ....|....| ....|....| ....|....|
                 1385       1395       1405       1415       1425       1435
2c_SalI      TTTGAAAAAT TAGAGGGTGA TTTACTAAAT AAAATAAACA CCCTTATGGG TTCTGAACAG
2c_Thai      TTTGAAAAAT TAGAGGGTGA TTTACTAAAT AAAATAAACA CCCTTATGGG TTCTGAACAG
Conservation ********** ********** ********** ********** ********** **********

             ....|....| ....|....| ....|....| ....|....| ....|....| ....|....|
                 1445       1455       1465       1475       1485       1495
2c_SalI      TCTACCTCTG ATCTTACCTC TATTATTGCG GATTCTGAAA AAATCATAAA ATCTGCAGAA
2c_Thai      TCTACCTCTG ATCTTACCTC TATTATTGCG GATTCTGAAA AAATCATAAA ATCTGCAGAA
Conservation ********** ********** ********** ********** ********** **********

             ....|....| ....|....| ....|....| ....|....| ....|....| ....|....|
                 1505       1515       1525       1535       1545       1555
2c_SalI      TCATTAATTA ATTCGAGTTC TGAAGAGATA GCAAAATATG CACTTGATTC AAATGAGAAA
2c_Thai      TCATTAATTA ATTCGAGTTC TGAAGAGATA GCAAAATATG CACTTGATTC AAATGAGAAA
Conservation ********** ********** ********** ********** ********** **********

             ....|....| ....|....| ....|....| ....|....| ....|....| ....|....|
                 1565       1575       1585       1595       1605       1615
2c_SalI      ATTAATGAGA TAAAAAAAAA TTATGACCAA AAGATACTCA GTGTAAAAGA ATTTATAAAT
2c_Thai      ATTAATGAGA TAAAAAAAAA TTATGACCAA AAGATACTCA GTGTAAAAGA ATTTATAAAT
Conservation ********** ********** ********** ********** ********** **********

             ....|....| ....|....| ....|....| ....|....| ....|....| ....|....|
                 1625       1635       1645       1655       1665       1675
2c_SalI      AAATCAAATG GGTTAATTAC AACTGTAAAA GGTACATCTA AACTAAGTGA ATCAGATAAG
2c_Thai      AAATCAAATG GGTTAATTAC AACTGTAAAA GGTACATCTA AACTAAGTGA ATCAGATAAG
Conservation ********** ********** ********** ********** ********** **********

             ....|....| ....|....| ....|....| ....|....| ....|....| ....|....|
                 1685       1695       1705       1715       1725       1735
2c_SalI      AAACAGATAG ATAAAAAAAT AGAGAAAGTA AAAAAAAATA CAAACACTCT AGAGAGGGGA
2c_Thai      AAACAGATAG ATAAAAAAAT AGAGAAAGTA AAAAAAAATA CAAACACTCT AGAGAGGGGA
Conservation ********** ********** ********** ********** ********** **********

             ....|....| ....|....| ....|....| ....|....| ....|....| ....|....|
                 1745       1755       1765       1775       1785       1795
2c_SalI      AATGAGTTTA TAAAAATAAT GAATGAAGTA AAAACAAAAA TAAGCAATTC TTCTAATAGC
2c_Thai      AATGAGTTTA TAAAAATAAT GAATGAAGTA AAAACAAAAA TAAGCAATTC TTCTAATAGC
Conservation ********** ********** ********** ********** ********** **********
```

```
             ....|....| ....|....| ....|....| ....|....| ....|....| ....|....|
                  1805       1815       1825       1835       1845       1855
2c_SalI      GCTACAAATT CAAAGGATTT TTCTCATAAA TTGAAAGAAT TACAAACTGA ATTTGAAGGG
2c_Thai      GCTACAAATT CAAAGGCATT TTCTGATAAA TTGAAAGAAT TACAAACTGA ATTTGAAGGA
Conservation ********** ******..** **** ***** ********** ********** *********.

             ....|....| ....|....| ....|....| ....|....| ....|....| ....|....|
                  1865       1875       1885       1895       1905       1915
2c_SalI      TTAAATAAAA CTATCGAAGA CGATTTGCAA AAAATAAAAG ACATAAAAGT AAAGGAAGAT
2c_Thai      TTAAATAAAA CTATCGAAGA CGATTTGCAA AAAATAAATG AGATAAAAAT AAAAGAAAAT
Conservation ********** ********** ********** ********:* * ******.* ***.***.**

             ....|....| ....|....| ....|....| ....|....| ....|....| ....|....|
                  1925       1935       1945       1955       1965       1975
2c_SalI      GAGGATCGTT CTATGAAAAA TCAAATAGAA GAACATTTGA AATATGCGTC TGATAACAGT
2c_Thai      GAGGATCGTT CTATGAAAGA TCAAATAGAA CAACATCTGA AAGATGCGTC TGATAACAGT
Conservation ********** ********.* ********** .*****.*** **.******* **********

             ....|....| ....|....| ....|....| ....|....| ....|....| ....|....|
                  1985       1995       2005       2015       2025       2035
2c_SalI      GACAAAGTAA AAAAGTTAAT TTCCAAAAAT AATGAAATAC AAAATTATAT TCAGAAAATC
2c_Thai      GAAAAAGTAA AAAGGTTAAT TTCCAAAAAT AATGAAATAC AAAAATATAT TCAGATAATT
Conservation **.******* ***.****** ********** ********** ****:***** *****:***

             ....|....| ....|....| ....|....| ....|....| ....|....| ....|....|
                  2045       2055       2065       2075       2085       2095
2c_SalI      GAAAATTTGA TTAAAGACGC ACCTTCTGGA AAAGAAAAAT TTACAACTCA GAAAACAGAT
2c_Thai      GAAAATTTGA TTAAAGATGC ACCTTCTGGC AAAGACAAAT TTACATCTGA GAAAACAGAT
Conservation ********** ******* ** *********. *****.**** *****.**.* **********

             ....|....| ....|....| ....|....| ....|....| ....|....| ....|....|
                  2105       2115       2125       2135       2145       2155
2c_SalI      TTGCAAAACA AGGTAAAGAC AATAATCGAT GAGTTTCATA AGGAAGATTT ACAATTATTA
2c_Thai      TTGCAAAACA AGGTAAAGGA AATAATCAAT GAGTTTCATA CGGGAGATTT ACAATTATTA
Conservation ********** ********.. *******.** ********** .**.****** **********

             ....|....| ....|....| ....|....| ....|....| ....|....| ....|....|
                  2165       2175       2185       2195       2205       2215
2c_SalI      TTAAATAGTT TATCAACATT TTACGAGGAA CACCAAAAGT TATACAGAGA AGCAAGTACT
2c_Thai      TTAAATAGTT TATCAACATT TTACGAGAAA CACCAAAAGT TATACAGCGG AGCAAGTACT
Conservation ********** ********** *******.** ********** *******.*. **********

             ....|....| ....|....| ....|....| ....|....| ....|....| ....|....|
                  2225       2235       2245       2255       2265       2275
2c_SalI      ATAGAAAACA TTAAAGAGTT GCACCCAAAA ATAAAAGAAG AATATGAAAA ACTTGAAAAA
2c_Thai      ATAGAAAACA TTAAAGAGTT GCACCCAAAA ATAAAAGAAG AATATGAAAA ACTTGAAAAA
Conservation ********** ********** ********** ********** ********** **********

             ....|....| ....|....| ....|....| ....|....| ....|....| ....|....|
                  2285       2295       2305       2315       2325       2335
2c_SalI      AAGAATTATA GTAATATTGA TCAAACATTA CATAATTTGG ATGCAGAACT AGATAAACTG
2c_Thai      AAGAATTATA GTAATATTGA TCAAACATTA CATAATTTGG ATGCAGAACT AGATAAACTG
Conservation ********** ********** ********** ********** ********** **********

             ....|....| ....|....| ....|....| ....|....| ....|....| ....|....|
                  2345       2355       2365       2375       2385       2395
2c_SalI      AAAGCTCTAG AGAAAAATAT TGTAGAAGAG CAAACTAATA ATATCAATAA AGATATGACA
2c_Thai      AAAGCTCTAG AGAAAAATAT TGTAGAAGAG CAAACTAATA ATATCAATAA AGATATGACA
Conservation ********** ********** ********** ********** ********** **********
```

```
             ....|....| ....|....| ....|....| ....|....| ....|....| ....|....|
                   2405       2415       2425       2435       2445       2455
2c_SalI      GATTCGTTGA ATAAGTTAAC TGCTGAAGTC AACAGTTTGA GAAGTGCCTT GGATAGCTAT
2c_Thai      GATTCGTTGA CTAAGTTAAA TACCGAAGTC GACAGTTTGA GAAATGCCTT GGATGGCTAT
Conservation ********** .********. *.*.****** .********* ***.****** ****.*****

             ....|....| ....|....| ....|....| ....|....| ....|....| ....|....|
                   2465       2475       2485       2495       2505       2515
2c_SalI      AAGGCGGATG AAGGTCATCT CAAAACCTAT AAAAACCAAA TAAATGAGAG GAAAGAGAAA
2c_Thai      AAGGCGGATG AAAGTGAGCT CAAAACCTAT AAAAACCGAA TGAATGAAAG GAAAGAAAAT
Conservation ********** **.** * ** ********** *******.** *.*****.** ******.**:

             ....|....| ....|....| ....|....| ....|....| ....|....| ....|....|
                   2525       2535       2545       2555       2565       2575
2c_SalI      TTTCTCAGTA CTTTAAAGGA ACAAGAGGAT GATATTCCAG ATGGAAAAAA TATTTATGAA
2c_Thai      TTTATCAGTA CTTTAAAGGA ACAAGAGGAT GATATTCCAG ATGGAAAAAA TATTTATGAA
Conservation ***.****** ********** ********** ********** ********** **********

             ....|....| ....|....| ....|....| ....|....| ....|....| ....|....|
                   2585       2595       2605       2615       2625       2635
2c_SalI      GAGTATAATA AACATAAAGG TGTTATGGAT AACAAAGAAC AAAAAATAAC CAGTGATATA
2c_Thai      GAGTGTAATA AACATAAAGG TGTTATGGAT AACAAAGAAC ATAAAATAAC TAGTGATATA
Conservation ****.***** ********** ********** ********** *:********  *********

             ....|....| ....|....| ....|....| ....|....| ....|....| ....|....|
                   2645       2655       2665       2675       2685       2695
2c_SalI      AATCAATGCA GAGAAAATAT TAAAAATGCT GAAAAAAATA GAGAGAAATT CAATACTCTA
2c_Thai      AATCAATGCA AAGAAAATAT TGTAAATGCC GAAAAAAATA TAGAGACATT CAATACTCTA
Conservation ********** .********* *.:******  **********  *****.*** **********

             ....|....| ....|....| ....|....| ....|....| ....|....| ....|....|
                   2705       2715       2725       2735       2745       2755
2c_SalI      GTGCAAACAT TAGAAGCACA CACAGGAGAA AAAGATCCGA ACGTTCATGA TTCATTGGAA
2c_Thai      GTGAAAACAT TAGAAGCACA CACAGGTAAA ACATATCCAG ACGCTCATAC TTCATTGCAA
Conservation ***.****** ********** ******:.** *.* ****.. ***.****.. *******.**

             ....|....| ....|....| ....|....| ....|....| ....|....| ....|....|
                   2765       2775       2785       2795       2805       2815
2c_SalI      AAGTTTAAGA CTAATCTTGA AAATTTAAAT TTGAGTAAAC TAGAAACTGA ATTTAAATCT
2c_Thai      AAGCTTAAAA GTAATCTAGA AAATTTAAAT TTGAGTGAAC TCGAAAATGA ATTTAAATCT
Conservation *** ****.* .******.** ********** ******.*** *.****.*** **********

             ....|....| ....|....| ....|....| ....|....| ....|....| ....|....|
                   2825       2835       2845       2855       2865       2875
2c_SalI      CTCATCGATT CAGCTAGTAC AACCAATAAA CAGATTGAAA ATATAATAAA AAATATAGAC
2c_Thai      CTCTTCGATT CAGCTAGTAC AACCAATAAA CAGATTGAAA ATATAAGAAA AAATATAGAC
Conservation ***.****** ********** ********** ********** ****** *** **********

             ....|....| ....|....| ....|....| ....|....| ....|....| ....|....|
                   2885       2895       2905       2915       2925       2935
2c_SalI      ACAATTAAAT CATTAAATTT TACGAAAAAT AGTTCGGACA GCAGCAAATT GTCATTGGAA
2c_Thai      ACAATTAAAT CATTAAATTT TACGAAAAAT AGTTCGGACA GCAGCAAATT GTCATTGGAA
Conservation ********** ********** ********** ********** ********** **********

             ....|....| ....|....| ....|....| ....|....| ....|....| ....|....|
                   2945       2955       2965       2975       2985       2995
2c_SalI      AAGATAAAAG AAAATAAAGC AGATTTAATA AAAAAACTAG AACAACACAC TCAAGAAATA
2c_Thai      AAGATAAAAG AAAATAAAGC AGATTTAATA AAAAAACTAG AACAACACAC TCAAGAAATA
Conservation ********** ********** ********** ********** ********** **********
```

```
               ....|....| ....|....| ....|....| ....|....| ....|....| ....|....|
                   3005       3015       3025       3035       3045       3055
2c_SalI        GAAAAATACA CATTCATAGA AAAAGAAGAA ACGTTACCCC TTTTAAGCGA TTTACGAGAA
2c_Thai        GAAAAATACA CATTCATAGA AAAAGAAGAA ACGTCACCCC TTTTAAGCGA TTTACGAGAA
Conservation   ********** ********** ********** **** ***** ********** **********

               ....|....| ....|....| ....|....| ....|....| ....|....| ....|....|
                   3065       3075       3085       3095       3105       3115
2c_SalI        GAAAAAAACC GCGTACAGCG TGACATGTCT GAAGAATTGA TAAGCCAATT AAACACAAAA
2c_Thai        GAAAAAAACC GCGTACAGCG TGACATGTCT GAAGAGTTGA TAAACCAGTT AAACACAAAA
Conservation   ********** ********** ********** *****.**** ***.***.** **********

               ....|....| ....|....| ....|....| ....|....| ....|....| ....|....|
                   3125       3135       3145       3155       3165       3175
2c_SalI        ATTAATGCTA TATTAGAATA TTACGATAAA TCAAAGGATA GTTTCAATGG GGATGATGAA
2c_Thai        ATTAATGATA TACTGGAATA TTACGATAAA TCAAAGGATA GTTTCAATGG GGATGATGAA
Conservation   *******.** ** *.***** ********** ********** ********** **********

               ....|....| ....|....| ....|....| ....|....| ....|....| ....|....|
                   3185       3195       3205       3215       3225       3235
2c_SalI        ACAAAATTGG AACAATTAGA TGAGTTTAAA AAAGAGTGTC AATACGTTCA ACAAGAAATA
2c_Thai        AAAAAATTGG AACAATTAGA TGACTTTAAA AGAAAGTGCC AAGATGTTCA ACAAGGAATA
Conservation   *.******** ********** *** ****** *.*.**** * ** * ***** *****.****

               ....|....| ....|....| ....|....| ....|....| ....|....| ....|....|
                   3245       3255       3265       3275       3285       3295
2c_SalI        GAAAAGTTAA CAACTAACTA TAAAGTTTTA GAAAATAAGA TAAATGACAT AATTAACGAA
2c_Thai        GAAAAGTTAA CAACTAACTA TAAAGTATTA GAAAATAGGA TAAATGACAT AATTAAAAAA
Conservation   ********** ********** ******:*** *******.** ********** ******..**

               ....|....| ....|....| ....|....| ....|....| ....|....| ....|....|
                   3305       3315       3325       3335       3345       3355
2c_SalI        CAACATGAAA AAGTTATAAC ACTGAGTGAA AATCATATAA CAGGGAAAGA CAAAAAGATA
2c_Thai        CAACATGAGA AAGTTATAAC AATGACTGAA AATCATATAA CAGGGAAAGA CAAAAAGATA
Conservation   ********.* ********** *.*** **** ********** ********** **********

               ....|....| ....|....| ....|....| ....|....| ....|....| ....|....|
                   3365       3375       3385       3395       3405       3415
2c_SalI        AATGAAAAGA TTCAACAAAA TATAAATTCG CTAAATGATA TGAAAACGAA ATTGGGTTTA
2c_Thai        AATGAAAAGA TTCAACAAAA TGTAAATTCG CTAAATGAAA TGAAAGCGAA ATTGGGTTTA
Conservation   ********** ********** *.******** ********.* *****.**** **********

               ....|....| ....|....| ....|....| ....|....| ....|....| ....|....|
                   3425       3435       3445       3455       3465       3475
2c_SalI        CTCGAAATTA ACCAAGATAT CAAAAATAGC AAAGATGCTA CAATTAAAAA TAAAATACAG
2c_Thai        CTCGAAATTA ACGAAGATAT CAAAAATAGC AAAGATGTTA CAATTAAAAA TAAAATACAG
Conservation   ********** **.******* ********** *******.** ********** **********

               ....|....| ....|....| ....|....| ....|....| ....|....| ....|....|
                   3485       3495       3505       3515       3525       3535
2c_SalI        GAATTTGAAA ACAAAGTTAA AACTATACTT GGAAATATAG ATAAAGCTAA TAATAAAATA
2c_Thai        GAATTTGAAA ACAAAGTTAA AACTATACTT GGAAATATAG GTAATGCTAA TAATAAAATA
Conservation   ********** ********** ********** ********** .***:***** **********

               ....|....| ....|....| ....|....| ....|....| ....|....| ....|....|
                   3545       3555       3565       3575       3585       3595
2c_SalI        GATGAAATTA AAAGAGTGCA CGTTGAGAAT AAAGTTGCAT TCGATAAAGA AAAAGTTGAA
2c_Thai        GATGCAATTA AAAGAGTGCA TGATGCGAAT AAAGTTGAAT TCGATAGAGA AAAAGTTAAA
Conservation   ****.***** **********  *.**.**** *******.** ******.*** *******.**
```

```
               ....|....| ....|....| ....|....| ....|....| ....|....| ....|....|
                   3605       3615       3625       3635       3645       3655
2c_SalI        AATACAAATT TCGAAAAAAA AAAGAAAAGC ATAGAAAAAG TTTATGAACA AATGGAAAAA
2c_Thai        AAAACAAGTT TCGATGAAAA AAAGGAAATC ATAGGAAAAG TTTATCAACA AATGGAAAAA
Conservation   **.****.** ****:.**** ****.*** * ****.***** ***** **** **********

               ....|....| ....|....| ....|....| ....|....| ....|....| ....|....|
                   3665       3675       3685       3695       3705       3715
2c_SalI        ACGATTAAAG AATTAGAGGA AATGAATGAC GAAAGTAACA TAGCAAAAGA AGTAGAAGAA
2c_Thai        ACTCTTAAAG AATTAGAGGA AATGGATGAC GAAAGTAGCA TAACAAAAGA TGTAGACGTA
Conservation   **..****** ********** ****.***** *******.** **.******* .*****.*.*

               ....|....| ....|....| ....|....| ....|....| ....|....| ....|....|
                   3725       3735       3745       3755       3765       3775
2c_SalI        GCTCAAATAC AATACAAAAG AATTTTTATT GATCATGATG TTAATTTGAT GAATGATGAA
2c_Thai        GCTCAAATAC TATACAGAAG AATTTTTATT AATCATGCTG TTAATTTGAT GGATGATGAA
Conservation   ********** .*****.*** ********** .******.** ********** *.********

               ....|....| ....|....| ....|....| ....|....| ....|....| ....|....|
                   3785       3795       3805       3815       3825       3835
2c_SalI        GTTGAAAAGT CCAAAACTGT GATGGAAGAA ATCGAATTAT ATAAAAAAGA AATTGACCAA
2c_Thai        GTTGAAAAGT CCAAAACTGT GATGGCAGAA ATCGAATTAT ATAAAAAAGA AATTGACGAA
Conservation   ********** ********** *****.**** ********** ********** *******.**

               ....|....| ....|....| ....|....| ....|....| ....|....| ....|....|
                   3845       3855       3865       3875       3885       3895
2c_SalI        ATTAAAAAGA AAATGATTGA GTATAATAAA GCTGATACAT CTAATTTTGA TTATACAGTA
2c_Thai        ACTAAACAGA AAACGATTGA ATATAAGCAA GGTGATACAT CTAATTTTGA TTATACAAAA
Conservation   *.****.*** ***.****** .***** .** *.******** ********** *******..*

               ....|....| ....|....| ....|....| ....|....| ....|....| ....|....|
                   3905       3915       3925       3935       3945       3955
2c_SalI        CCATACAACA GTGCTACACA GAGTAAAGCT AAAATAGAAC AATTTATTAC GAATGCTAAA
2c_Thai        GAATACAACA ATGCTACACA GAGTAAAGCT AAAATAGAAA ATTTTATTGA AAATGTTAAG
Conservation   ..******** .********* ********** *********. *.******.. .****.***.

               ....|....| ....|....| ....|....| ....|....| ....|....| ....|....|
                   3965       3975       3985       3995       4005       4015
2c_SalI        ACAAAAAAAG GAACGTCTGA CGCCAGCCAA GATATAAAAG AATTAGAACG TATTAAAGAA
2c_Thai        ACAAAAAAAG GAACGTCTGA CACAAGCCAA AATATAAAAG AATTAGAAAG TATTAAAGAA
Conservation   ********** ********** *.*.****** .********* ********.* **********

               ....|....| ....|....| ....|....| ....|....| ....|....| ....|....|
                   4025       4035       4045       4055       4065       4075
2c_SalI        GAGGTGCATA CTAATTTACA ACAAGTCAAA CTAGAAAATA ATTCTATGGA GGAAAAGCGA
2c_Thai        GAGGTGCATG ATAATTTACA ACAAGTCAAA CTAGAAAATG TTTCTATGGA GGAAATTCGA
Conservation   *********. .********* ********** *********. .********* *****..***

               ....|....| ....|....| ....|....| ....|....| ....|....| ....|....|
                   4085       4095       4105       4115       4125       4135
2c_SalI        AAACAAATTC ATAATATGAA AAATTTGCTA ATTTTGAACA ATTCTGAAAC CATAGCTAAA
2c_Thai        AAACAAATTC TTAATATGAA AAATTTACTA ATTTCGAACA ATTCTGAAAC CATAGCAAAA
Conservation   ********** .********* ******.*** ****.***** ********** ******.***

               ....|....| ....|....| ....|....| ....|....| ....|....| ....|....|
                   4145       4155       4165       4175       4185       4195
2c_SalI        GAAATATCAA ATAATACTCA AAACGCATTA AATTTTAGCC TGGATGCAAA AAAAGAACTT
2c_Thai        GAAATATCAA ATAATACTCA AAACGCATTA GGTTTTAGGG ATAATGCAAA AACAGAACTT
Conservation   ********** ********** ********** ..******   ...******* **.*******
```

```
             ....|....| ....|....| ....|....| ....|....| ....|....| ....|....|
                  4205       4215       4225       4235       4245       4255
2c_SalI      AAAAAAACAG ATGAACTATT ACAAAGTGTG GAAGCTAAGA TAGCTGAGGC AGAGGCACAT
2c_Thai      AATAAAACAA ATGAACTATT ACAAAGTGTG GAAGCTAAGA TAGCTGAGGC AGAGGCACAT
Conservation **.******. ********** ********** ********** ********** **********

             ....|....| ....|....| ....|....| ....|....| ....|....| ....|....|
                  4265       4275       4285       4295       4305       4315
2c_SalI      AAAAAAAAAA TTGATATAGC TTTAGAAGAT GGACAAATAG ATACGGAGGT AAGCAAAATT
2c_Thai      AAAAAAAAAA TTGGCATAGC TTTAGAAGAT GGACAAATAG ACACGGAGGT AAGCGAAATT
Conservation ********** ***..***** ********** ********** * ******** ****.*****

             ....|....| ....|....| ....|....| ....|....| ....|....| ....|....|
                  4325       4335       4345       4355       4365       4375
2c_SalI      GAACAAATTA ATCAGGAAAT TATGAATAAG AGAGATGAAA TTAAATCCTA TTTAAGTGAA
2c_Thai      GAACAAATTA AGCGAGAAAT TATAAATAAA AAAAATGAAA TTGAATCCTA TTTAAATGAA
Conservation ********** * *..***** ***.*****. *.*.****** **.******* *****.****

             ....|....| ....|....| ....|....| ....|....| ....|....| ....|....|
                  4385       4395       4405       4415       4425       4435
2c_SalI      ATAAAAAAAT ATAAAGAGAA ATGTACAACC GAAATCAGTA ATTCAAAAAG AGGAAAAGAT
2c_Thai      ATAAAAGAAT ATAAAGACAA ATGCACAACC GAAATCAGTA ATTCAAAAAG AGGAAAAGAT
Conservation ******.*** ******* ** *** ****** ********** ********** **********

             ....|....| ....|....| ....|....| ....|....| ....|....| ....|....|
                  4445       4455       4465       4475       4485       4495
2c_SalI      AAAATTGAGT TCTTGAAAAT ATTTAAGCCT AATGAAGATA GCAATCGGAA TAAGGTTAAC
2c_Thai      AAAATTGAGT TCTTGGAAAA ATTTAAGCCT AATGAGGAAA GCAATCGGAA TAAGGTTAAC
Conservation ********** *****.***. ********** *****.**.* ********** **********

             ....|....| ....|....| ....|....| ....|....| ....|....| ....|....|
                  4505       4515       4525       4535       4545       4555
2c_SalI      ATTAATGAAA TAGATGGAAA TATAAGCAAA TCTGAACAAT ACTTAACAGA TATAAAGGGC
2c_Thai      ATTAATGAAA TAAATGAAAA TATAAGAAAT TCTGAACAAT ACTTAAAAGA TATAGAAGAC
Conservation ********** **.***.*** ******.**. ********** ******.*** ****.*.*.*

             ....|....| ....|....| ....|....| ....|....| ....|....| ....|....|
                  4565       4575       4585       4595       4605       4615
2c_SalI      ACAGAAAAAC AAGCTAATAC AAATGTAGAA CTATTCCATA ATCATGAAAC AAATATCAGT
2c_Thai      GCAGAAAAAC AAGCTAGTAC AAAAGTAGAA CTATTCCATA AACATGAAAC AACTATCAGT
Conservation .********* ******.*** ***.****** ********** *.******** **.*******

             ....|....| ....|....| ....|....| ....|....| ....|....| ....|....|
                  4625       4635       4645       4655       4665       4675
2c_SalI      AATATTTTCA AGCAATCTGA AATTTTAGGA GTAGAAACTA AATCAAAAAA AAAAATTAGA
2c_Thai      AATATTTTCA AGGAATCTGA AATTTTAGGA GTGGAAACTA AATCCCAAAA AAAAATTAAT
Conservation ********** ** ******* ********** **.******* ****..**** ********..

             ....|....| ....|....| ....|....| ....|....| ....|....| ....|....|
                  4685       4695       4705       4715       4725       4735
2c_SalI      GAAGCAGATG ACATAATGAA AGAAATTGAG GGCCACAATT CTGAAATTCA AACACAGGTG
2c_Thai      AAAGCAGAAG ACATAATGAA AGAAATTGAG CGTCACAATT CTGAAATTCA AACACAGGTG
Conservation .*******.* ********** ********** .*.******* ********** **********

             ....|....| ....|....| ....|....| ....|....| ....|....| ....|....|
                  4745       4755       4765       4775       4785       4795
2c_SalI      AAGAGTTTCC AAGAAAATCT AAATAAATTG AACGAGCCCC ATAATTATGA CAACGCAGAA
2c_Thai      AAAGGTTTCC AAGAAAATCT AAATAAACTG AACGAGCCCC ATAATTATGA CAACGCAGAA
Conservation **..****** ********** *******.** ********** ********** **********
```

```
             ....|....| ....|....| ....|....| ....|....| ....|....| ....|....|
                4805       4815       4825       4835       4845       4855
2c_SalI      GATGAACTTA ATAATGATAA ATCTACGAGT GCAAAGGTGC TTATACAGAC TAACTTAGAA
2c_Thai      GATGAACTTA ATAATGATAA ATCTACGAAT GCAAAGGTAC TTATAGAAAC TAACCTAGAA
Conservation ********** ********** ********.* ********.* ***** *.** **** *****

             ....|....| ....|....| ....|....| ....|....| ....|....| ....|....|
                4865       4875       4885       4895       4905       4915
2c_SalI      AGTGTAAAAC ATAATTTATC CGAAATTACT AACATTAAAG ATGGAGGAGA AAACATATAC
2c_Thai      AGTGTAAAAC ATAATTTATC AGAAATTACT AATATTAAAC AGGGAGGAGA AAAAATATAC
Conservation ********** ********** .********* ** ******  *.******** ***.******

             ....|....| ....|....| ....|....| ....|....| ....|....| ....|....|
                4925       4935       4945       4955       4965       4975
2c_SalI      AATAAAGCTA ATGATATCAT GCAAAAAATA AAAGCAATTT CAAAAAATTC TGCAGAGAAA
2c_Thai      AGTAAAGCTA AAGATATCAT GCAAAAAATA AAAGCAACTT CAGAAAATAC TGCAGAGAAA
Conservation *.******** *.******** ********** ******* ** **.*****.* **********

             ....|....| ....|....| ....|....| ....|....| ....|....| ....|....|
                4985       4995       5005       5015       5025       5035
2c_SalI      ACTTTGGACA AGGTGAAAGA CGACCATTCT AATTATGTTA ATTATTTAGA TCAAATAATT
2c_Thai      ACTTTAGAGA AGGTGAAAGA CGACCAATCT AATTATGTTA ATTATTTAAA TCAAATAACC
Conservation *****.** * ********** ******.*** ********** ********.* ********..

             ....|....| ....|....| ....|....| ....|....| ....|....| ....|....|
                5045       5055       5065       5075       5085       5095
2c_SalI      ACAGAAAGAG ATCTTATCGT TACAGAAAAA AATAGACTTA ATGGTAAAGA TTCCACTATT
2c_Thai      ACAGAAAGAA ATCTTATCGT TACGGAAAAA AATAGACTAA ATGGTATAGA TTCCACTATT
Conservation *********. ********** ***.****** ********.* ******.*** **********

             ....|....| ....|....| ....|....| ....|....| ....|....| ....|....|
                5105       5115       5125       5135       5145       5155
2c_SalI      AAAAATATAG AAGCGGCACT GAAAGAATCC AAAAAGAATT ACGAAATTGG ACTTTTGGAA
2c_Thai      ACAAATATAG AAGGGGCACT TAAAGAATCC AAAAAGAATT ATGAAATTGG ATTTTTGGAA
Conservation *.******** ***.******  ********* ********** *.******** *.********

             ....|....| ....|....| ....|....| ....|....| ....|....| ....|....|
                5165       5175       5185       5195       5205       5215
2c_SalI      AAGTTAGAAG AAATAGGTAA AAATATAAAA TTAAAGGTTG ACATAACCAA AGAATCAATA
2c_Thai      AAGTTAGAAG AAATAGGTAA AAATAGAAAA TTAAAGGTTG ACATAACCAA AAAATCAATA
Conservation ********** ********** ***** **** ********** ********** *.********

             ....|....| ....|....| ....|....| ....|....| ....|....| ....|....|
                5225       5235       5245       5255       5265       5275
2c_SalI      AATTCAACAG TGGGAAACTT TTCTTCCCTC TTCAACAATT TTGATTTAAA TCAATATGAC
2c_Thai      AATTCAACAG TGGGAAACTT TTCTTCCCTC TTCAACAATT TTGATTTAAA TCAATATGAC
Conservation ********** ********** ********** ********** ********** **********

             ....|....| ....|....| ....|....| ....|....| ....|....| ....|....|
                5285       5295       5305       5315       5325       5335
2c_SalI      TTTAATAATA ATATAAATGA TTATAAAAAA AAAATGGGAG AAATATATAA CGAATTTGAA
2c_Thai      TTTAATAAAA ATATAAATGA TTATGAAAAT AAAATGGGAG AAATATATAA CGAATTTGAA
Conservation ********:* ********** ****.****. ********** ********** **********

             ....|....| ....|....| ....|....| ....|....| ....|....| ....|....|
                5345       5355       5365       5375       5385       5395
2c_SalI      GGATCATTAA ATAAAATTAG TGAAATTTTA AGAAATGCTT CAGAAAAAGC TTCAGATTAT
2c_Thai      GGATCATTAA ATAAAATTAG TGAAATTTTA AGAAATGCTT CGGAAAACAC TTCAGACTAT
Conservation ********** ********** ********** ********** *.*****..* ****** ***
```

```
             ....|....| ....|....| ....|....| ....|....| ....|....| ....|....|
                  5405       5415       5425       5435       5445       5455
2c_SalI      AACTTAGCCA AAACACTGAG GCAAGATGCA CAGAAAGAAA AAGTTAATCT ATTAAATAAA
2c_Thai      AACTCAGCAA AAACACTGAG GCTAGAGGCA CAGAAAGAAA AAGTTAATCT ATTAAATAAA
Conservation **** ***.* ********** **.*** *** ********** ********** **********

             ....|....| ....|....| ....|....| ....|....| ....|....| ....|....|
                  5465       5475       5485       5495       5505       5515
2c_SalI      GAAGAAGAGG CAAATAAATA TTTAAGAGAT GTTAAAAAAG TGGAATCATT CAGATTTATA
2c_Thai      GAAGAAGAGG CAAATAAATA TTTAAGAGAT GTTAAAAAAG TGGAATCATT CAGATTTATA
Conservation ********** ********** ********** ********** ********** **********

             ....|....| ....|....| ....|....| ....|....| ....|....| ....|....|
                  5525       5535       5545       5555       5565       5575
2c_SalI      CTTAATATGA AAGAAAGCCT AGATAAGATT AATGATATGA TTAAAACAGA AAAATTAACA
2c_Thai      TTTAATATGA AAGAAAGCTT AGATAAGATT AATGAGATGA TTAAAAAAGA ACAACTAACA
Conservation  ********* ******** * ********** ***** **** ******.*** *.**.*****

             ....|....| ....|....| ....|....| ....|....| ....|....| ....|....|
                  5585       5595       5605       5615       5625       5635
2c_SalI      GTCGATGAAG GACATGGTAA CGTTAAACAG CTAGTTGAAA ATATTAAAGA TTTAGTTGAT
2c_Thai      GTCAATGAAG GACAAGGTAA CGTTAAACAA CTAGTTGAAA ATATTAAAGA GTTAGTTGAT
Conservation ***.****** ****.***** *********. ********** **********  *********

             ....|....| ....|....| ....|....| ....|....| ....|....| ....|....|
                  5645       5655       5665       5675       5685       5695
2c_SalI      GAAAACAACT TATCAGATAT GTTAAAACAA GCATCAGGCA AAAATGACGA AATACAGAAA
2c_Thai      GAAAACAACT TATCAGATAT ATTAAAACAA GCGACGGGCA AAAATGAGGA AATACAGAAA
Conservation ********** ********** .********* **..*.**** *******.** **********

             ....|....| ....|....| ....|....| ....|....| ....|....| ....|....|
                  5705       5715       5725       5735       5745       5755
2c_SalI      ATAACGCACT CTATGCATAA AAATAAAGCA AAAACTATTT TAGGACACGT AGATACTTCT
2c_Thai      ATAACGCACT CTACGCTTAA AAATAAAGCA AAAACTATTT TAGGACACGT AGATACTTCT
Conservation ********** *** **.*** ********** ********** ********** **********

             ....|....| ....|....| ....|....| ....|....| ....|....| ....|....|
                  5765       5775       5785       5795       5805       5815
2c_SalI      GCAAAATATG TAGGCATTAA AATAATGCCA GAGTTGGCAT TAACAGAATT GTTAGGAGAT
2c_Thai      GCAAAATATG TAGGCATTAA AATAACACCT GAGTTGGCAC TAACAGAATT GTTAGGAGAT
Conservation ********** ********** *****..**. *********. ********** **********

             ....|....| ....|....| ....|....| ....|....| ....|....| ....|....|
                  5825       5835       5845       5855       5865       5875
2c_SalI      TCAAATTTGA AAAATGCACA AGAATTAATA TTTGAGCCCA AAACTAATGT ACCACTAGAA
2c_Thai      GCAAAATTGA AAACTGCACA GGAATTAAAA TTTGAGTCAA AAAATAATGT AGTACTAGAA
Conservation  ****:**** ***.****** .*******.* ******.*.* ***.****** *..*******

             ....|....| ....|....| ....|....| ....|....| ....|....| ....|....|
                  5885       5895       5905       5915       5925       5935
2c_SalI      ACAGAACATA TGTCAAAGAA TACAAACGAA CTGGATGTTT ATAAAAATAT ACAGGATGCT
2c_Thai      ACAGAAAATA TGTCAAAGAA TACAAACGAA TTGGATGTTC ATAAAAATAT ACAGGATGCT
Conservation ******.*** ********** **********  ********  ********** **********

             ....|....| ....|....| ....|....| ....|....| ....|....| ....|....|
                  5945       5955       5965       5975       5985       5995
2c_SalI      TACAAGGTTG CATTGGAAAT ACTTGCCCAC TCAGACGAAA TAGATACAAA ACAAAAAGAC
2c_Thai      TACAAGGTTG CACTGGAAAT ACTTGCCCAC TCAGACGAAA TAGATACAAA ACAAAAAGAC
Conservation ********** ** ******* ********** ********** ********** **********
```

```
             ....|....| ....|....| ....|....| ....|....| ....|....| ....|....|
                 6005       6015       6025       6035       6045       6055
2c_SalI      AGTTCTAAAT TAATAGAAAT GGGAAACGAA ATATATCTTA AGGTTGTGCT AATAAATCAA
2c_Thai      AGTTCTAAAT TAATAGAAAT GGGAAACGAA ATATATCTTA AAGTTGTGCT AATAAATCAA
Conservation ********** ********** ********** ********** *.******** **********

             ....|....| ....|....| ....|....| ....|....| ....|....| ....|....|
                 6065       6075       6085       6095       6105       6115
2c_SalI      TACAAAAATA AAATAAGCTC TATAAAAATT AAGGAAGAAG CTGTTTCAGG CAAAATAGAC
2c_Thai      TACAAAAATA AAATAAGCTC TATAAAAATT AAGGAAGAAG CTGTTTCAGG CAAAATAGAC
Conservation ********** ********** ********** ********** ********** **********

             ....|....| ....|....| ....|....| ....|....| ....|....| ....|....|
                 6125       6135       6145       6155       6165       6175
2c_SalI      AATGTTTCCA AAAAACATAG TGAGTTAAGC AAAATTACAT GCAGCGATAA AACTTACGAT
2c_Thai      AATGTTTCCA AAAAACATAG TGAGTTAAGC AAAATTACAT GCAGCGATAA AACTTACGAT
Conservation ********** ********** ********** ********** ********** **********

             ....|....| ....|....| ....|....| ....|....| ....|....| ....|....|
                 6185       6195       6205       6215       6225       6235
2c_SalI      AACATCATAG CGTTAGAGAA ACAAACTGAA TTACAAGATC TACGCAATTC TTTCACGCAA
2c_Thai      AACATCATAG CGTTAGAGAA ACAAACTGAA TTACAAGATC TACGCAATTC TTTCACGCAA
Conservation ********** ********** ********** ********** ********** **********

             ....|....| ....|....| ....|....| ....|....| ....|....| ....|....|
                 6245       6255       6265       6275       6285       6295
2c_SalI      GAAAAGACCG AAACGAATAG CGATTCGAAG TTGGAAACAA TTACAAAAGA TTTCGAAAGT
2c_Thai      GAAAAGACCG AAACGAATAG CGATTCGAAG TTGGAAACAA TTACAAAAGA TTTCGAAAGT
Conservation ********** ********** ********** ********** ********** **********

             ....|....| ....|....| ....|....| ....|....| ....|....| ....|....|
                 6305       6315       6325       6335       6345       6355
2c_SalI      TTGAAAAATG CATTAAAAAC ACTGGAAGGA GAAGTTAATG CTCTAAAAGC AAGCTCGAAC
2c_Thai      TTGAAAAATG CATTAAAAAC ACTGGAAGGA GAAGTTAATG CTCTAAAAGC AAGCTCGAAC
Conservation ********** ********** ********** ********** ********** **********

             ....|....| ....|....| ....|....| ....|....| ....|....| ....|....|
                 6365       6375       6385       6395       6405       6415
2c_SalI      GATCATGAAT ATGTAAAAGG TAAAAGTGCA CCAATAAATG CTGTGCAAAC CGAAATGGAA
2c_Thai      GATCATGAAT ATGTAAAAGG TAAAAGTGCA CCAATAAATG CTGTGCAAAC CGAAATGGAA
Conservation ********** ********** ********** ********** ********** **********

             ....|....| ....|....| ....|....| ....|....| ....|....| ....|....|
                 6425       6435       6445       6455       6465       6475
2c_SalI      AAGACAGAAA CAGGCATAGA TAGCCTTGAT ACGGCCCTTG ATGAATTATT ACAAAAAGGA
2c_Thai      AAGACAGAAA CAGGCATAGA TAGCCTTGAT ACGGCCCTTG ATGAATTATT ACAAAAAGGA
Conservation ********** ********** ********** ********** ********** **********

             ....|....| ....|....| ....|....| ....|....| ....|....| ....|....|
                 6485       6495       6505       6515       6525       6535
2c_SalI      AGGAAATGCG AAGTATCTAG GTACACATTG ATAAAGGATA CCGTTGTCAA AGAAATAAGT
2c_Thai      AGGAAATGCG AAGTATCTAG GTACACATTG ATAAAGGATA CCGTTGTCAA AGAAATAAGT
Conservation ********** ********** ********** ********** ********** **********

             ....|....| ....|....| ....|....| ....|....| ....|....| ....|....|
                 6545       6555       6565       6575       6585       6595
2c_SalI      GATGACACCG AATTAATCAA CACTATAGAG AAGAATGTTA AAGCATACTT GGCATATGTT
2c_Thai      GATGACACCG AATTAATCAA CACTATAGAG AAGAATGTTA AAGCATACTT GGCATATGTT
Conservation ********** ********** ********** ********** ********** **********
```

```
             ....|....| ....|....| ....|....| ....|....| ....|....| ....|....|
                  6605       6615       6625       6635       6645       6655
2c_SalI      AAAAAAAATT ATGAAGACAC AGTGCAGGAT GTTCTCACAT TAAATGAACA TTTCAAAGAA
2c_Thai      AAAAAAAATT ATGAAGACAC AGTGCAGGAT GTTCTCACAT TAAATGAACA TTTCAAAGAA
Conservation ********** ********** ********** ********** ********** **********

             ....|....| ....|....| ....|....| ....|....| ....|....| ....|....|
                  6665       6675       6685       6695       6705       6715
2c_SalI      AAACAGGTAA GTAATCACGA GCCAACTAAT TTTGATAAAT CAAATAAGTC ATCCGAAGAG
2c_Thai      AAACAGGTAA GTAATCACGA GCCAACTAAT TTTGATAAAT CAAATAAGTC ATCCGAAGAG
Conservation ********** ********** ********** ********** ********** **********

             ....|....| ....|....| ....|....| ....|....| ....|....| ....|....|
                  6725       6735       6745       6755       6765       6775
2c_SalI      TTAACTAAAG CTGTTACTGA CTCAAAAACA ATAATAAGTA AACTAAAAGG TGTAATTATA
2c_Thai      TTAACTAAAG CTGTTACTGA CTCAAAAACA ATAATAAGTA AACTAAAAGG TGTAATTATA
Conservation ********** ********** ********** ********** ********** **********

             ....|....| ....|....| ....|....| ....|....| ....|....| ....|....|
                  6785       6795       6805       6815       6825       6835
2c_SalI      GAAGTTAACG AAAACACTGA AATGAACACT ATAGAAAGCA GTGCAAAAGA AATTGAAGCT
2c_Thai      GAAGTTAACG AAAACACTGA AATGAACACT ATAGAAAGCA GTGCAAAAGA AATTGAAGCT
Conservation ********** ********** ********** ********** ********** **********

             ....|....| ....|....| ....|....| ....|....| ....|....| ....|....|
                  6845       6855       6865       6875       6885       6895
2c_SalI      CTCTATAACG AATTAAAAAA TAAAAAAACA TCATTAAACG AAATTTATCA AACATCAAAT
2c_Thai      CTCTATAACG AATTAAAAAA TAAAAAAACA TCATTAAACG AAATTTATCA AACATCAAAT
Conservation ********** ********** ********** ********** ********** **********

             ....|....| ....|....| ....|....| ....|....| ....|....| ....|....|
                  6905       6915       6925       6935       6945       6955
2c_SalI      GAAGTTAAAT TGCAAGAAAT GAAATCAAAT GCTGATAAAT ACATCGATGT ATCTAAAATA
2c_Thai      GAAGTTAAAT TGCAAGAAAT GAAAACAAAT GCTGATAAAT ACATCGATGT ATCTAAAATA
Conservation ********** ********** ****:***** ********** ********** **********

             ....|....| ....|....| ....|....| ....|....| ....|....| ....|....|
                  6965       6975       6985       6995       7005       7015
2c_SalI      TTTAACACTG TATTAGACAC TCAAAAGTCA AATATAGTAA CTAATCAACA TAGCATAAAC
2c_Thai      TTTAACACTG TATTAGACAC TCAAAAGTCA AAAATAGTGG CTAATCAAAA TAGTATAAAC
Conservation ********** ********** ********** **:*****.. ********.* *** ******

             ....|....| ....|....| ....|....| ....|....| ....|....| ....|....|
                  7025       7035       7045       7055       7065       7075
2c_SalI      AATGTTAAAG ACAAATTAAA AGGAAAGCTA CAGGAATTAA TTGACGCTGA CAGTTCATTT
2c_Thai      AGTGTTAAAG GCCAATTAAA CGGAAAGCTA AAGGAATTAA TTCAGGCTGA CAGCTCATTT
Conservation *.******** .*.******* .********* .********* ** * ***** *** ******

             ....|....| ....|....| ....|....| ....|....| ....|....| ....|....|
                  7085       7095       7105       7115       7125       7135
2c_SalI      ACATTAGAGT CCATTAAAAA GTTTAACGAA ATATATAGTC ATATTAAGAC TAATATAGGT
2c_Thai      ACATTAGAGT CCATTAAAAA GTTTAACGAA ATATATAGTC ATATTAAGAC TAATATAGGT
Conservation ********** ********** ********** ********** ********** **********

             ....|....| ....|....| ....|....| ....|....| ....|....| ....|....|
                  7145       7155       7165       7175       7185       7195
2c_SalI      GAACTAGAAC AGTTACAACA AACTAATAAA AGTGAACATG ATAATGTCGC AAAGCACAAA
2c_Thai      GAACTAGAAC AGTTACAACA AACTAATAAA AGTGAACATG ATAATGTCGC AAAGCACAAA
Conservation ********** ********** ********** ********** ********** **********
```

```
             ....|....| ....|....| ....|....| ....|....| ....|....| ....|....|
                  7205       7215       7225       7235       7245       7255
2c_SalI      GAAAAAATTG TACATTTAAT AAACAGGGTA GAAAGTTTGA AAGGTGATGT GAAAAATCAT
2c_Thai      GAAAAAATTG TACATTTAAT AAACAGGGTA GAAAGTTTGA AAGGTGATGT GAAAAATCAT
Conservation ********** ********** ********** ********** ********** **********

             ....|....| ....|....| ....|....| ....|....| ....|....| ....|....|
                  7265       7275       7285       7295       7305       7315
2c_SalI      GATGATGACC AATATATGAA AAAATTAAAT GCTAGTCTAT TAAATGATAA TATTAAAAAT
2c_Thai      GATGATGACC AATATATGAA AAAATTAAAT GCTAGTCTAT TAAATGATAA TATTAAAAAT
Conservation ********** ********** ********** ********** ********** **********

             ....|....| ....|....| ....|....| ....|....| ....|....| ....|....|
                  7325       7335       7345       7355       7365       7375
2c_SalI      ACAACGAATT CTATAAACAT ATCAGATGAA GAACTTAAGA AACTATTGAA AAAAGTTGAA
2c_Thai      ACAACGAATT CTATAAACAT ATCAGATGAA GAACTTAAGA AACTATTGAA AAAAGTTGAA
Conservation ********** ********** ********** ********** ********** **********

             ....|....| ....|....| ....|....| ....|....| ....|....| ....|....|
                  7385       7395       7405       7415       7425       7435
2c_SalI      GAGAATGATC AGCTATGTAA AAATAACAAT ACGCAGAACT TCATTTCGGA CATTATGAAA
2c_Thai      GAGAATGATC AGCTATGTAA AAATAACAAT ACGCAGAACT TCATTTCGGA CATTATGAAA
Conservation ********** ********** ********** ********** ********** **********

             ....|....| ....|....| ....|....| ....|....| ....|....| ....|....|
                  7445       7455       7465       7475       7485       7495
2c_SalI      CGTGTAGAAG ACTTGAATAG ACGATTTACA GAAAATTTAC CGGAAAAAGA AAAACTTCAT
2c_Thai      CGTGTAGAAG ACTTGAATAG ACGATTTACA GAAAATTTAC CGGAAAAAGA AAAACTTCAT
Conservation ********** ********** ********** ********** ********** **********

             ....|....| ....|....| ....|....| ....|....| ....|....| ....|....|
                  7505       7515       7525       7535       7545       7555
2c_SalI      CAAATTGAAA ACAACTATAA TGAAATTAGT TCAATATTTA GTGAAATAAA CTTACAAGAC
2c_Thai      CAAATTGAAA ACAACTATAA TGAAATTAGT TCAATATTTA GTGAAATAAA CTTACAAGAC
Conservation ********** ********** ********** ********** ********** **********

             ....|....| ....|....| ....|....| ....|....| ....|....| ....|....|
                  7565       7575       7585       7595       7605       7615
2c_SalI      GTCGATGAAT TTGTTGCAAA AATTCACAAA CAAATTGATG CTGAAAAGGC AAGCGTAAAT
2c_Thai      GTCGATGAAT TTGTTGCAAA AATTCACAAA CAAATTGATG CTGAAAAGGC AAGCGTAAAT
Conservation ********** ********** ********** ********** ********** **********

             ....|....| ....|....| ....|....| ....|....| ....|....| ....|....|
                  7625       7635       7645       7655       7665       7675
2c_SalI      AATGTAAGAG AGGCTGAAAA AATAAGGACC GCAATCCAAA ATGTTACAAG TTATGATACA
2c_Thai      AATGTAAGAG AGGCTGAAAA AATAAGGACC GCAATCCAAA ATGTTACAAG TTATGATACA
Conservation ********** ********** ********** ********** ********** **********

             ....|....| ....|....| ....|....| ....|....| ....|....| ....|....|
                  7685       7695       7705       7715       7725       7735
2c_SalI      GAAATAATAA GTAGACTATC TGAAATGAAT AATGTACTAG AAAGGATTAC TACACGGAAA
2c_Thai      GAAATAATAA GTAGACTATC TGAAATGAAT AATGTACTAG AAAGGATTAC TACACGGAAA
Conservation ********** ********** ********** ********** ********** **********

             ....|....| ....|....| ....|....| ....|....| ....|....| ....|....|
                  7745       7755       7765       7775       7785       7795
2c_SalI      ACAAAAATGG ATCAATTATT AAAATCATTG TCCCCAGATA ATACAAGTCT AAATTTAAAT
2c_Thai      ACAAAAATGG ATCAATTATT AAAATCATTG TCCCCAGATA ATACAAGTCT AAATTTAAAT
Conservation ********** ********** ********** ********** ********** **********
```

```
             ....|....| ....|....| ....|....| ....|....| ....|....| ....|....|
                 7805       7815       7825       7835       7845       7855
2c_SalI      GCAAGAACAC ACGTAAGGAA ATCAGAAGAC ATAATTAAAC AGCTAAATAG CCATATAGAA
2c_Thai      GCAAGAACAC ACGTAAAGAA CTCAGAAGAC TTAATTAATA GGCTAAATAG CCATATAGAA
Conservation ********** ******.*** .********* .*******.. .********* **********

             ....|....| ....|....| ....|....| ....|....| ....|....| ....|....|
                 7865       7875       7885       7895       7905       7915
2c_SalI      AAAATAACAG AACTAAATAC ATATGCCCAT GAAGTAATGA CATACTTAGA AAATGAACTA
2c_Thai      AAAATAACAA AACTAAATAC ACATGCCCAC GAAGTAATGA CATACTTAGA AAATGAACTA
Conservation *********. ********** *.*******  ********** ********** **********

             ....|....| ....|....| ....|....| ....|....| ....|....| ....|....|
                 7925       7935       7945       7955       7965       7975
2c_SalI      AATAAATTAT TAAAACAACT TGAAATTGAA AGTGCAAAAG TTGAGCCTGA AGCTTTGCCA
2c_Thai      AGTAAATTAT TAAAACAACT TGAAATTGAA AGTGCAAAAG TTGAGCCTGA AGCTTTGCCA
Conservation *.******** ********** ********** ********** ********** **********

             ....|....| ....|....| ....|....| ....|....| ....|....| ....|....|
                 7985       7995       8005       8015       8025       8035
2c_SalI      AGTGATACGG AAGTAAAAGA AGAAAAAGTT CCACCAACGG TAACAGAAAA TGGTCCACAA
2c_Thai      AGTGATACGG AAGTAAAAGA AGAAAAAGTT CCACCAACGG TAACAGAAAA TGGTCCACAA
Conservation ********** ********** ********** ********** ********** **********

             ....|....| ....|....| ....|....| ....|....| ....|....| ....|....|
                 8045       8055       8065       8075       8085       8095
2c_SalI      GAGAATTTGA CAAGCGTTCC CCAAGAGACA TTAGAAGATA ACACACCACA GATCCAAGAA
2c_Thai      GAGAATTTGA CAAGCGTTCC CCAAGAGACA TCAGAAGATA ACACACCACA GATCCAAGAA
Conservation ********** ********** ********** * ******** ********** **********

             ....|....| ....|....| ....|....| ....|....| ....|....| ....|....|
                 8105       8115       8125       8135       8145       8155
2c_SalI      AATGTTGTAC AAGAGGATTC AGTAATCGCT CCTCAGGAGC AAGTAGAATA TAGTACACTA
2c_Thai      AATGTTGTAC AAGAGGATTC AGTAATCGCT CCTCAGGAGC AAGTAGAATA TAGTACACTA
Conservation ********** ********** ********** ********** ********** **********

             ....|....| ....|....| ....|....| ....|....| ....|....| ....|....|
                 8165       8175       8185       8195       8205       8215
2c_SalI      GCGGTACCAG AAAATGATGA AACAACAGAA GAAGAAAGTG AACATGACGA TGCGCATGAC
2c_Thai      GCGGTACCAG AAAATGATGA AACAACAGAA GAAGAAAGTG AACATGACGA TGCGCATGAC
Conservation ********** ********** ********** ********** ********** **********

             ....|....| ....|....| ....|....| ....|....| ....|....| ....|....|
                 8225       8235       8245       8255       8265       8275
2c_SalI      GATACGCATG ACGAATCACA AACTGGAAGG GATTCAACGG CAAAAGAAGC AATTGGAAAG
2c_Thai      GATACGCATG ACGAATCACA AACTGGAAGG GATTCAACGG CAAAAGAAGC AATTGGAAAG
Conservation ********** ********** ********** ********** ********** **********

             ....|....| ....|....| ....|....| ....|....| ....|....| ....|....|
                 8285       8295       8305       8315       8325       8335
2c_SalI      ACTCGTTTAG CAGGAGCTGT TATTATTGCC ATGTCTGTTT TATCCGGATT TATTATAATA
2c_Thai      ACTCGTTTAG CAGGAGCTGT TATTATTGCC ATGTCTGTTT TATCCGGATT TATTATAATA
Conservation ********** ********** ********** ********** ********** **********

             ....|....| ....|....| ....|....| ....|....| ....|....| ....|....|
                 8345       8355       8365       8375       8385       8395
2c_SalI      GTATTTAAAG ATAAAGATGA AGAAGAAAAG GATCACAACG AACACGGATA TAACGAAGCA
2c_Thai      GTATTTAAAG ATAAAGATGA AGAAGAAAAG GATCACAACG AACACGGATA TAACGAAGCA
Conservation ********** ********** ********** ********** ********** **********
```

```
             ....|....| ....|....| ....|....| ....|....| ....|....| ....|....|
                8405       8415       8425       8435       8445       8455
2c_SalI      TTTGGAGAAC ATGACGAATA TAATATGCAT GATAAAGAAG AAGTTATCGA AGTTTGCTTT
2c_Thai      TTTGGAGAAC ATGACGAATA TAATATGCAT GATAAAGAAG AAGTTATCGA AGTTTGCTTT
Conservation ********** ********** ********** ********** ********** **********

             ....|....| ....|
                8465       8475
2c_SalI      AATGAAGAAG ATTAA
2c_Thai      AATGAAGAAG ATTAA
Conservation ********** *****
```

Alignment: Pvrbp2p1

```
             ....|....| ....|....| ....|....| ....|....| ....|....| ....|....|
                     5         15         25         35         45         55
2p1_SalI     ATGGAAAAAA ATATCCTGTG GGTAATCTTT TACAATTTCC TGTTTATTCT TTTGGCATCA
2p1_Thai     ATGGAAAAAA ATATCCTGTG GGTAATCTTT TACAATTTCC TGTTTATTCT TTTGGCATCA
Conservation ********** ********** ********** ********** ********** **********

             ....|....| ....|....| ....|....| ....|....| ....|....| ....|....|
                    65         75         85         95        105        115
2p1_SalI     TGTAAGGACA CACATCGAAG CAAACCTAGC AGACTAAAAC ATGACCATAA TTTATTACCA
2p1_Thai     TGTAAGGACA CACATCGAAG CAAACCTAGC AGACTAAAAC ATGACCATAA TTTATTGCCA
Conservation ********** ********** ********** ********** ********** ******.***

             ....|....| ....|....| ....|....| ....|....| ....|....| ....|....|
                   125        135        145        155        165        175
2p1_SalI     AATTACGCTA ATTTAATGAG GGATGATCAA AACGGACAAA ACAGTGAAAA CAGAGGAGAC
2p1_Thai     AATTACGTTA ATTTAATGAA GGATGATCAA AACGGACAAA ACAGTGAAAA CAGAGGAGAC
Conservation *******.** *********. ********** ********** ********** **********

             ....|....| ....|....| ....|....| ....|....| ....|....| ....|....|
                   185        195        205        215        225        235
2p1_SalI     AACATAAACA ATCACAATAA AAACCATAAC GATCAGAACA ACCATAATGG TAATAATGAT
2p1_Thai     AACATAAACA ATCACAATAA AAACCATAAC GATCAGAACA ACCATAATGG TAATAATGAT
Conservation ********** ********** ********** ********** ********** **********

             ....|....| ....|....| ....|....| ....|....| ....|....| ....|....|
                   245        255        265        275        285        295
2p1_SalI     AACAGCATTA ATAGTGAATA CCTAAAAACG TCACATCTAC AGAATAGTTC GGCCATGGTA
2p1_Thai     AACAGCATTA ATAGTGAATA CCTAAAAACT TCACATCTAC AGAATAGTTC GGCCATGGTA
Conservation ********** ********** *********. ********** ********** **********

             ....|....| ....|....| ....|....| ....|....| ....|....| ....|....|
                   305        315        325        335        345        355
2p1_SalI     CACCTAAACG ATCATAAAAT TACAACAAAG CCAGCTCGCT ATTCTTACAT ACAAAGAAGC
2p1_Thai     CACCTAAATG ATCATAAAAT TACAACAAAG CCAGCTCGCT ATTCTTACAT ACAAAGAAGC
Conservation ******** * ********** ********** ********** ********** **********

             ....|....| ....|....| ....|....| ....|....| ....|....| ....|....|
                   365        375        385        395        405        415
2p1_SalI     AAAATATATG CCTTTAACCC AAATAATAAA AAAATAGAAA ATATTAATAA CGAATTGCAT
2p1_Thai     AAAATATATG CACTTAACCC AAATAATAAA AAAATAGAAA ATATTAATAA CGAATTGCAT
Conservation ********** *..******* ********** ********** ********** **********

             ....|....| ....|....| ....|....| ....|....| ....|....| ....|....|
                   425        435        445        455        465        475
2p1_SalI     AGTGTTCCAA ATTCATTTGT TCAGTATTAT GTATACACCA ATCTAAATGT TATAGTACAA
2p1_Thai     AGTGTTCCAA ATTCATTTGT TCAGTATTAT GTATACACCA ATCTAAATGC TACAGTATAT
Conservation ********** ********** ********** ********** *********. **.****.*.

             ....|....| ....|....| ....|....| ....|....| ....|....| ....|....|
                   485        495        505        515        525        535
2p1_SalI     AAGATGCGTT ACATTAGTAT CATCGATGAG TATAATAATA TAATTTCTTT GCATCTGCCT
2p1_Thai     AAGATGCGTT ACATTAGTAT CTTCGATGAG TATAATAATA TAGTTTCTTT GCATCTGCCT
Conservation ********** ********** *:******** ********** **.******* **********

             ....|....| ....|....| ....|....| ....|....| ....|....| ....|....|
                   545        555        565        575        585        595
2p1_SalI     TATCATGCTA GAGTATATTA CTTAATGGAA TTAAAAAATT ATTCAGTGGC TCATAAAGAA
2p1_Thai     TATCATGCTA GAGTAAATTA CTTAATGGAA ATAAAAAATT ATTCAGTGGC TTATAGAGAA
Conservation ********** *****:**** ********** .********* ********** *.***.****
```

```
             ....|....| ....|....| ....|....| ....|....| ....|....| ....|....|
                   605        615        625        635        645        655
2p1_SalI     TTACAGAGTT ATGTTTATAG TTATTATGAT CCACAAATAG ACAAAGTTTT CAAGGCTGCA
2p1_Thai     TTACAGAGTG ATGTTTATAG TTATTATGAT CCACAAATAG ACAAAGTTTT CAAGGTTGCA
Conservation *********. ********** ********** ********** ********** *****.****

             ....|....| ....|....| ....|....| ....|....| ....|....| ....|....|
                   665        675        685        695        705        715
2p1_SalI     GAGAAAAATG GCTTTGTCTG TGAAAATGAC ATAAACAATT TATATAATAT AGTAACTCAT
2p1_Thai     GAGAAAAATG CAATTGTCTG TGAAAAGTAC ATAAACAATT TATATAATAC AGTAACTCGT
Conservation ********** ...******* ******..** ********** *********  ********.*

             ....|....| ....|....| ....|....| ....|....| ....|....| ....|....|
                   725        735        745        755        765        775
2p1_SalI     TTAGAAAATC CAGAACACTA TAAAAATAAG AGATACAATT ATTCCAAAGA CAGAGAAAAA
2p1_Thai     TTAGAAAATC CAGAACAATT TAAAAATAAC AGAGACAATT ATTCCAAAGA AATAAAAGAA
Conservation ********** *******.*. *********. ***.****** ********** .*.*.**.**

             ....|....| ....|....| ....|....| ....|....| ....|....| ....|....|
                   785        795        805        815        825        835
2p1_SalI     TTCCAAAAAC AACTTTCGTC CGTTCATGGC TGCTTATTAA CAAATCATAA ACAAAATTAC
2p1_Thai     TACCAAAAAC AACTTACGTC CCTTCATGAT TGCTTATTTG AAAATCATAG ACCAAATTAC
Conservation *:******** *****.**** *.******.. ********.. .********. **.*******

             ....|....| ....|....| ....|....| ....|....| ....|....| ....|....|
                   845        855        865        875        885        895
2p1_SalI     AAAGACATGA TATATGCTGA CACAACAATT TTCGATTATT TACATTATAT ATACTGTTAT
2p1_Thai     AAAGACATAA TATATGCTGA TACAACAATT TTCGATTCTT TACAAAATAT ATACTGTTAT
Conservation ********.* **********  ********* *******.** ****..**** **********

             ....|....| ....|....| ....|....| ....|....| ....|....| ....|....|
                   905        915        925        935        945        955
2p1_SalI     CCGGAAGATA AATGTTCATC AAAATATTAT CGTGATATGC TTAATATATC TATGGGAAAA
2p1_Thai     TCGGAAGATA AATGTCTATC AAAATTGTAT CGTGATATGC TTGGTTTATC TATGAAAAAA
Conservation  ********* *****..*** *****..*** ********** **..*.**** ****..****

             ....|....| ....|....| ....|....| ....|....| ....|....| ....|....|
                   965        975        985        995       1005       1015
2p1_SalI     ATTAATGAAT ATGAACAGAA AAAAAAAAAT GAAGCTATAA ATAAAATTAT TGATGTACAC
2p1_Thai     ATTAATGAAT ATGAACAGAA AAAAAAAAAT GAAGATATAA ATAAAATTAT TAATATACAC
Conservation ********** ********** ********** ****.***** ********** *.**.*****

             ....|....| ....|....| ....|....| ....|....| ....|....| ....|....|
                  1025       1035       1045       1055       1065       1075
2p1_SalI     GAAACTGGTG CATATGTAAT GAGGAAAATA AAAGAAGAAT TAAATCCAAG CTTAAATTCT
2p1_Thai     GATACTGCTG TAGATGTAAT GAGGAAAATA AAAAGGGAAT TAAATCCAAG CTTAAATTCT
Conservation **.****.** .*.******* ********** ***...**** ********** **********

             ....|....| ....|....| ....|....| ....|....| ....|....| ....|....|
                  1085       1095       1105       1115       1125       1135
2p1_SalI     GATGTGGCAG ATTTCGTAAT AGATGAAATT AAATATATTA TAGAGAGACT TAATGCACAC
2p1_Thai     GATGTGGCAG ATTTCGTAAT AGGTGAAATT AAATATATTA TAGAGAGACT TAAGGCACAC
Conservation ********** ********** **.******* ********** ********** ***.******

             ....|....| ....|....| ....|....| ....|....| ....|....| ....|....|
                  1145       1155       1165       1175       1185       1195
2p1_SalI     TCAGAAAAAA TAAAATGTGC ATCCGATTTT ATAAAACATA TATATAAAGA AAAAGTGCAG
2p1_Thai     TCAGAAAAAA TTAAAAGTGC ATCCGATTTG GTAAAATATA TAAATCAAGA AACTGTGCCG
Conservation ********** *.***.**** *********. .*****.*** **.**.**** **..****.*
```

```
              ....|....| ....|....| ....|....| ....|....| ....|....| ....|....|
                 1205       1215       1225       1235       1245       1255
2p1_SalI      AACGAAATCT CAAAAAATGA GCTTGAAAGT AATTATATTG TATTAAGTAT TCACCACGGT
2p1_Thai      AACGAAATCT CAAAAAATGA GATTAAAAGT AATTATATTG TGTTAGCTAT TCACACCGGT
Conservation  ********** ********** *.**.***** ********** *.***..*** ****..****

              ....|....| ....|....| ....|....| ....|....| ....|....| ....|....|
                 1265       1275       1285       1295       1305       1315
2p1_SalI      TCTTTTCGTT TTAGTACTGA ACATGTAATC ATGTTAGAGG AAATATTCAA AAGTAAAGAA
2p1_Thai      TCCTTTCTTT TTAGTACTGA ACATGTAAAA ATGTTAGAGG AAATATTCAA AAGTAAAGAA
Conservation  **.****.** ********** ********.. ********** ********** **********

              ....|....| ....|....| ....|....| ....|....| ....|....| ....|....|
                 1325       1335       1345       1355       1365       1375
2p1_SalI      CAAATACTTC ACAACATTTG CAGCAAATTT TTGAATGAGT TGAAAAACAA AATAACTGCT
2p1_Thai      CAAATACTTT ACAAAATTTG TAGTAAATTT TTGAATGACT TGAAAAACAG AATAACTACT
Conservation  *********. ****.*****  **.****** ********.* *********. *******.**

              ....|....| ....|....| ....|....| ....|....| ....|....| ....|....|
                 1385       1395       1405       1415       1425       1435
2p1_SalI      CTTATAAATT CGGAATATTC TTCATCAAAA TGCACTCCTA TTGTTTCTAC TTGTGAAGAA
2p1_Thai      CTGATAAATT CGGAATATTC TTCATCAAAC TGCACTCCTA TTGTTTCTAC TTGTGAAGAA
Conservation  **.******* ********** *********. ********** ********** **********

              ....|....| ....|....| ....|....| ....|....| ....|....| ....|....|
                 1445       1455       1465       1475       1485       1495
2p1_SalI      GCCAAAACAT CCTTAGAATC CTTGCGTACT TCGAGCACCG AAAAACTAGG TAAGCATGAT
2p1_Thai      GCCAAAAAAT CCTTAGAATC CTTGCGTACT TCGAGCACCG GAAAACTAGG TAAGCATGAT
Conservation  *******.** ********** ********** ********** .********* **********

              ....|....| ....|....| ....|....| ....|....| ....|....| ....|....|
                 1505       1515       1525       1535       1545       1555
2p1_SalI      CTAAATTCAA AGCCGGAAAT TGCATCAGTT AAACAGAGTT ATGATGGGAA AATGATTAAA
2p1_Thai      CTAAATTCAA AGCCGGAAAT TGCATCAGTT AAACAGAGTT ATGATGGGAA AATGATTAAA
Conservation  ********** ********** ********** ********** ********** **********

              ....|....| ....|....| ....|....| ....|....| ....|....| ....|....|
                 1565       1575       1585       1595       1605       1615
2p1_SalI      TTAGCAGAAG CTATAAAAAG AGCCGAAGAA ATAATCAATT CTGTAAAGGA CATCGTTCAG
2p1_Thai      TTAGCAGAAG CTATAAAAAG AGCCGAAGAA ATAATCAATT CTGTAAAGGA CATCGTTCAG
Conservation  ********** ********** ********** ********** ********** **********

              ....|....| ....|....| ....|....| ....|....| ....|....| ....|....|
                 1625       1635       1645       1655       1665       1675
2p1_SalI      TTTAACACAA CCGAAACAGA TACTATGAAA AAGGAAACAG ATAGAATAAT ATCCCTAAAT
2p1_Thai      TTTAACACAA CCGAAACAGA AGCAAAGAAA AAGGAAACAG ATAG---CAT ACCCCAAAAT
Conservation  ********** ********** ..*.*.**** ********** ****   .** * ***.****

              ....|....| ....|....| ....|....| ....|....| ....|....| ....|....|
                 1685       1695       1705       1715       1725       1735
2p1_SalI      ATAAATCCAC TATCGAAGGA TAAAATGCTT TTAGAAGTAA TAGATTCAAT AAAAAAACAA
2p1_Thai      ATAAATGCAC TAGAGAAGGA TAAAAAACTT TTAGAAGTAA TAGATGCAAT AAAAAAACAA
Conservation  ****** *** **..****** *****..*** ********** ***** **** **********

              ....|....| ....|....| ....|....| ....|....| ....|....| ....|....|
                 1745       1755       1765       1775       1785       1795
2p1_SalI      AAAGAAAAAA TATCGGAAAA TTCTAACAAA ATAAAAGAAT CTTCTGACGC AGCTGATGCT
2p1_Thai      AAACAAAAAA TATCGGAAAA TTCTAACAAA ATAAAAGAAT TTTCTGGTGC AGCTGATACT
Conservation  *** ****** ********** ********** ********** .*****. ** *******.**
```

```
             ....|....| ....|....| ....|....| ....|....| ....|....| ....|....|
                1805       1815       1825       1835       1845       1855
2p1_SalI     TTAAAAGCTG AAGTTGAAGA ATTAAAGAAA GGTATTGATG AAGATGTGAA TAAAATATTA
2p1_Thai     TTAAAAGCTG AAGTCGAAGA ATTAAAGAAA GGTATTGATG AAGATGTTAA TAAAATATTA
Conservation ********** **** ***** ********** ********** *******.** **********


             ....|....| ..
                1865
2p1_SalI     AAGCCATTCT AA
2p1_Thai     AAGCCATTCT AA
Conservation ********** **
```

Alignment: Pvrbp2d

```
             ....|....| ....|....| ....|....| ....|....| ....|....| ....|....|
                    5         15         25         35         45         55
2d_SalI      ATGGAAAAAA GAGCCTTTTG GATCATCTTT TACAATTTAA TATTTATTTT TTTCGCATCA
2d_Thai      ATGGAAAAAA AAGCCCTTTG GATCATCTTT TACAATTTCA TACTTATTCT TTTCGCATCA
Conservation ********** .**** **** ********** ********.* ** ***** * **********

             ....|....| ....|....| ....|....| ....|....| ....|....| ....|....|
                   65         75         85         95        105        115
2d_SalI      TGTAAGGATG TCAATCGAAA CAAACCCGGA AGACTAAAAT ATCATCGGAA GCTATTACCA
2d_Thai      TGTAAGGATG TCAATCGAAA CAAACCCGGA AGACTAAAAT ATCATCGGAA GCTATTACCA
Conservation ********** ********** ********** ********** ********** **********

             ....|....| ....|....| ....|....| ....|....| ....|....| ....|....|
                  125        135        145        155        165        175
2d_SalI      GCATATGTTA ACTTAATGAG CCCTGATAAA GACGGATATA AGGGTGCCGA AAAGGATGAT
2d_Thai      GCATATGTTA ACTTAATGAG CCCTGATAAA GACGGATATA AGGGTGCCGA AAAGGATGAT
Conservation ********** ********** ********** ********** ********** **********

             ....|....| ....|....| ....|....| ....|....| ....|....| ....|....|
                  185        195        205        215        225        235
2d_SalI      AAGGCAAATA ATCATAACGA CAACAATAAT GGCAACAATA ATGACAACAA TAATAATAGT
2d_Thai      AAGGCAAATA ATCATAACGA CAACAATAAT GGCAACAATA ATGACAACAA TAATAATAGT
Conservation ********** ********** ********** ********** ********** **********

             ....|....| ....|....| ....|....| ....|....| ....|....| ....|....|
                  245        255        265        275        285        295
2d_SalI      GACAATAATG ATGAAAACAG TAATAATAGT GAAAATCTCA GAACGTCAAA TTTACAAAAT
2d_Thai      GACAATAATG ATGAAAACAG TAATAATAGT GAAAATCTCA GAACGTCAAA TTTACAAAAT
Conservation ********** ********** ********** ********** ********** **********

             ....|....| ....|....| ....|....| ....|....| ....|....| ....|....|
                  305        315        325        335        345        355
2d_SalI      AGTTCATCAG TGGGGCATTT AAACAACCAC GAAGATACAA CGAAACCATC CCATTACTCT
2d_Thai      AGTTCATCAG TGGGGCATTT AAACAACCAC GAAGATACAA CGAAACCATC CCATTATTCT
Conservation ********** ********** ********** ********** ********** ****** ***

             ....|....| ....|....| ....|....| ....|....| ....|....| ....|....|
                  365        375        385        395        405        415
2d_SalI      TACTTGAAAA AAAGCAACAT ATATGCACCG GATGCAAAGA ATAATAAAAT GGAAGATGAT
2d_Thai      TACTTGAAAA AAAGCAACAT ATATGCACCG GATGCAAAGA ATAATAAAAT GGAAGATGAT
Conservation ********** ********** ********** ********** ********** **********

             ....|....| ....|....| ....|....| ....|....| ....|....| ....|....|
                  425        435        445        455        465        475
2d_SalI      AATAAAAAAT TGCATACTGT TTCAAATTCC TTCATTCAAA ATAGTAAACA AGCCCAAAAT
2d_Thai      AATAAAAAAT TGCATACTGT TTCAAATTCC TTCATTCAAA ATAGTAAACA AGCCCAAAAT
Conservation ********** ********** ********** ********** ********** **********

             ....|....| ....|....| ....|....| ....|....| ....|....| ....|....|
                  485        495        505        515        525        535
2d_SalI      ATAATTGCAA CTAATAATTT GGATTATATT AGTGCCATTG ATGATTATAA CAATATAATT
2d_Thai      ATAATTGCAA CTAATAATTT GGATTATATT AGTGCCATTG ATGATTATAA CAATATAATT
Conservation ********** ********** ********** ********** ********** **********

             ....|....| ....|....| ....|....| ....|....| ....|....| ....|....|
                  545        555        565        575        585        595
2d_SalI      TCTTCATTTC AACCATATCA TGCTAGTGTA TATTACTTGA ATGAATTGAA ATACTATGCA
2d_Thai      TCTTCATTTC AACCATAACA TGCTAGTGTA TATTACTTGA ATGAATTGAA ATACTATGCA
Conservation ********** *******.** ********** ********** ********** **********
```

```
             ....|....| ....|....| ....|....| ....|....| ....|....| ....|....|
                  605        615        625        635        645        655
2d_SalI      ACACATTACA ATGAATTGAA GGTTCTTGTT CAAAAAGATG TCATTCCTCA AGTAACACAC
2d_Thai      ACACATTACA ATGAATTGAA GGTTCTTGTT CAAAAAGATG TCATTCCTCA AGTAACACAC
Conservation ********** ********** ********** ********** ********** **********

             ....|....| ....|....| ....|....| ....|....| ....|....| ....|....|
                  665        675        685        695        705        715
2d_SalI      ATTACCAATA TTGTTGAGAA AAACGTTAAG ATCTGTTTGG AGAAAACACA TGAATTGAAT
2d_Thai      ATTACCAATA TTGTTGAGAA AAACGTTAAG ATCTGTTTGG AGAAAACACA TGAATTGAAT
Conservation ********** ********** ********** ********** ********** **********

             ....|....| ....|....| ....|....| ....|....| ....|....| ....|....|
                  725        735        745        755        765        775
2d_SalI      ACATTAATAA CTCAATTAGA AAATCCACAA ACATATAATA TTCAAAGAAG CCATTATAGT
2d_Thai      ACATTAATAA CTCAATTAGA AAATCCACAA ACATATAATA TTCAAAGAAG CCATTATAGT
Conservation ********** ********** ********** ********** ********** **********

             ....|....| ....|....| ....|....| ....|....| ....|....| ....|....|
                  785        795        805        815        825        835
2d_SalI      AATAGAGTAA AAGATTACCG TAAGAAAATT GAGGATATTC AAAATTGTTT AAAATTAAAT
2d_Thai      AATAGAGTAA AAGATTACCG TAAGAAAATT GAGGATATTC AAAATTGTTT AAAATTAAAT
Conservation ********** ********** ********** ********** ********** **********

             ....|....| ....|....| ....|....| ....|....| ....|....| ....|....|
                  845        855        865        875        885        895
2d_SalI      TATAAAAGAA ATGTTAAAGC TACAGTATTT GCCACAATAA TATGGTTCCA GACACTAATT
2d_Thai      TATAAAAGAA ATGTTAAAGC TACAGTATTT GCCACAATAA TATGGTTCCA GACACTAATT
Conservation ********** ********** ********** ********** ********** **********

             ....|....| ....|....| ....|....| ....|....| ....|....| ....|....|
                  905        915        925        935        945        955
2d_SalI      AAAATGGAAT GTAGTTGGTG GACCAAATCA TGTTCAACGA GAATCTATTA TAATATGATT
2d_Thai      AAAATGGAAT GTAGTTGGTG GACCAAATCA TGTTCAACGA GAATCTATTA TAATATGATT
Conservation ********** ********** ********** ********** ********** **********

             ....|....| ....|....| ....|....| ....|....| ....|....| ....|....|
                  965        975        985        995       1005       1015
2d_SalI      AAGATGTATA CATTAAAAAT TAAGGAGTAT AAAATCAAAA AACCAACTGC ATATATGGAT
2d_Thai      AAGATGTATA CATTAAAAAT TAAGGAGTAT AAAATCAAAA AACCAACTGC ATATATGGAT
Conservation ********** ********** ********** ********** ********** **********

             ....|....| ....|....| ....|....| ....|....| ....|....| ....|....|
                 1025       1035       1045       1055       1065       1075
2d_SalI      AAAATAAAGG CTGTTTATAA GTTAGCTAAT GATACAATAT GGAGAATAAA AGTAGAATTA
2d_Thai      AAAATAAAGG CTGTTTATAA GTTAGCTAAT GATACAATAT GGAGAATAAA AGTAGAATTA
Conservation ********** ********** ********** ********** ********** **********

             ....|....| ....|....| ....|....| ....|....| ....|....| ....|....|
                 1085       1095       1105       1115       1125       1135
2d_SalI      AACTCAAATC TTGATTCGGA TACAACCGAC TTTATTCTAG AAGAATTTAA ATACATTATA
2d_Thai      AACTCAAATC TTGATTCGGA TACAACCGAC TTTATTCTAG AAGAATTTAA ATACATTATA
Conservation ********** ********** ********** ********** ********** **********

             ....|....| ....|....| ....|....| ....|....| ....|....| ....|....|
                 1145       1155       1165       1175       1185       1195
2d_SalI      GAAAAGTATA ATGGTCACAT TGATAAAATT AATTTGGGAA CTTCTTATAT AGATCATATT
2d_Thai      GAAAAGTATA ATGGTCACAT TGATAAAATT AATTTGGGAA CTTCTTATAT AGATCATATT
Conservation ********** ********** ********** ********** ********** **********
```

```
             ....|....| ....|....| ....|....| ....|....| ....|....| ....|....|
                 1205       1215       1225       1235       1245       1255
2d_SalI      CATGAACAAG AAGGAACTTT GAATAATACT AAAATAGAAA TTATAACTTT GTATTCTGTG
2d_Thai      CATGAACAAG AAGGAACTTT GAATAATACT AAAATAGAAA TTATAACTTT GTATTCTGTG
Conservation ********** ********** ********** ********** ********** **********

             ....|....| ....|....| ....|....| ....|....| ....|....| ....|....|
                 1265       1275       1285       1295       1305       1315
2d_SalI      ATAGCTAATC GTTACACTGC TTTTAAATTT AGTTTAGAAC ATATAAATAT GTTTGAAAAT
2d_Thai      ATAGCTAATC GTTACACTGC TTTTAAATTT AGTTTAGAAC ATATAAATAT GTTTGAAAAT
Conservation ********** ********** ********** ********** ********** **********

             ....|....| ....|....| ....|....| ....|....| ....|....| ....|....|
                 1325       1335       1345       1355       1365       1375
2d_SalI      ACATCCAAAA GTAAAGAACA GGTACTTTAT AACTCTTTTA GCAAATTTGA GGATAGATTA
2d_Thai      ACATCCAAAA GTAAAGAACA GGTACTTTAT AACTCTTTTA GCAAATTTGA GGATAGATTA
Conservation ********** ********** ********** ********** ********** **********

             ....|....| ....|....| ....|....| ....|....| ....|....| ....|....|
                 1385       1395       1405       1415       1425       1435
2d_SalI      CAAAAGAAAA TAAACGATCT TATTAATTCT GAATTTTCTA TATCAACCGT TAACTCTGTC
2d_Thai      CAAAAGAAAA TAAACGATCT TATTAATTCT GAATTTTCTA TATCAACCGT TAACTCTGTC
Conservation ********** ********** ********** ********** ********** **********

             ....|....| ....|....| ....|....| ....|....| ....|....| ....|....|
                 1445       1455       1465       1475       1485       1495
2d_SalI      ATTTTGGATT CCGAAAAAAA TATACAATTA TCAGAATCAT TACTCAACTC GAGTTCTAAA
2d_Thai      ATTTTGGATT CCGAAAAAAA TATACAATTA TCAGAATCAT TACTCAACTC GAGTTCTAAA
Conservation ********** ********** ********** ********** ********** **********

             ....|....| ....|....| ....|....| ....|....| ....|....| ....|....|
                 1505       1515       1525       1535       1545       1555
2d_SalI      GAAATAGCAC AACATGAAAT TAAATTAAAT GCGGAAATTG AAAAGAAAAA AAAAGATTAT
2d_Thai      GAAATAGCAC AACATGAAAT TAAATTAAAT GCGGAAATTG AAAAGAAAAA AAAAGATTAT
Conservation ********** ********** ********** ********** ********** **********

             ....|....| ....|....| ....|....| ....|....| ....|....| ....|....|
                 1565       1575       1585       1595       1605       1615
2d_SalI      GATCAAAAAA TTATAAGAGC AAGAGAAACT ATAAAAAAAT CAAAGGAATT AATTACATCT
2d_Thai      GATCAAAAAA TTATAAGAGC AAGAGAAACT ATAAAAAAAT CAAAGGAATT AATTACATCT
Conservation ********** ********** ********** ********** ********** **********

             ....|....| ....|....| ....|....| ....|....| ....|....| ....|....|
                 1625       1635       1645       1655       1665       1675
2d_SalI      GTAAAAAACG CATTTAAGTT AAGTTTAGAA GAAAAGGAAA AGATAGAAAA GAAAGTAACA
2d_Thai      GTAAAAAACG CATTTAAGTT AAGTTTAGAA GAAAAGGAAA AGATAGAAAA GAAAGTAACA
Conservation ********** ********** ********** ********** ********** **********

             ....|....| ....|....| ....|....| ....|....| ....|....| ....|....|
                 1685       1695       1705       1715       1725       1735
2d_SalI      GAAGCAAAAA AATTGCCAAA CGCTCTAGAA AGAGACAAAG CATATTTAGA CATTATGAGT
2d_Thai      GAAGCAAAAA AATTGCCAAA CGCTCTAGAA AGAGACAAAG CATATTTAGA CATTATGAGT
Conservation ********** ********** ********** ********** ********** **********

             ....|....| ....|....| ....|....| ....|....| ....|....| ....|....|
                 1745       1755       1765       1775       1785       1795
2d_SalI      GAAATAAGAA AAATTAAAAA TAAGTTATCT GATAATGCTA GAAAGACAAC CGAATTTACT
2d_Thai      GAAATAAGAA AAATTAAAAA TAAGTTATCT GATAATGCTA GAAAGACAAC CGAATTTACT
Conservation ********** ********** ********** ********** ********** **********
```

```
             ....|....| ....|....| ....|....| ....|....| ....|....| ....|....|
                  1805       1815       1825       1835       1845       1855
2d_SalI      AAAAAATCAA ATGCTTTAAA AAAGGAAGTT CAAGAATTAA ATACAAGTGT TAACAACTAT
2d_Thai      AAAAAATCAA ATGCTTTAAA AAAGGAAGTT CAAGAATTAA ATACAAGTGT TAACAACTAT
Conservation ********** ********** ********** ********** ********** **********

             ....|....| ....|....| ....|....| ....|....| ....|....| ....|....|
                  1865       1875       1885       1895       1905       1915
2d_SalI      GTGCAAGCAA TAAAGGAACA AAAAGAAAAA GAAATTCGGA ATAATTCTTT GAAAGATGAA
2d_Thai      GTGCAAGCAA TAAAGGAACA AAAAGAAAAA GAAATTCGGA ATAATTCTTT GAAAGATGAA
Conservation ********** ********** ********** ********** ********** **********

             ....|....| ....|....| ....|....| ....|....| ....|....| ....|....|
                  1925       1935       1945       1955       1965       1975
2d_SalI      ATAAAAAAGT TTTTGGAATA TATACCTAAT AACAGAGAAA AAATAAAAGA ATTAATTACC
2d_Thai      ATAAAAAAGT TTTTGGAATA TATACCTAAT AACAGAGAAA AAATAAAAGA ATTAATTACC
Conservation ********** ********** ********** ********** ********** **********

             ....|....| ....|....| ....|....| ....|....| ....|....| ....|....|
                  1985       1995       2005       2015       2025       2035
2d_SalI      AAAAAGAATG AAGTGCAACA ATATATTCCC AAAATTGAAG AATTAATTAA AGACGCACAC
2d_Thai      AAAAAGAATG AAGTGCAACA ATATATTCCC AAAATTGAAG AATTAATTAA AGACGCACAC
Conservation ********** ********** ********** ********** ********** **********

             ....|....| ....|....| ....|....| ....|....| ....|....| ....|....|
                  2045       2055       2065       2075       2085       2095
2d_SalI      TTTGGTGTAG AAGAATTTAC AGCCAAAAAA ACAGAATTGC AAAATCAGGT AAATTCAATA
2d_Thai      TTTGGTGTAG AAGAATTTAC AGCCAAAAAA ACAGAATTGC AAAATCAGGT AAATTCAATA
Conservation ********** ********** ********** ********** ********** **********

             ....|....| ....|....| ....|....| ....|....| ....|....| ....|....|
                  2105       2115       2125       2135       2145       2155
2d_SalI      ATTGATGCAT TTTATAAAGA AGACCTACAA TTATTTTTAG ACAATTTATC AAAATCTTAT
2d_Thai      ATTGATGCAT TTTATAAAGA AGACCTACAA TTATTTTTAG ACAATTTATC AAAATCTTAT
Conservation ********** ********** ********** ********** ********** **********

             ....|....| ....|....| ....|....| ....|....| ....|....| ....|....|
                  2165       2175       2185       2195       2205       2215
2d_SalI      GATGAAAATC AAGTTTTAGA GAAGGAAGCT GATACTACAG AAAAGATTGA AGAACTTCAC
2d_Thai      GATGAAAATC AAGTTTTAGA GAAGGAAGCT GATACTACAG AAAAGATTGA AGAACTTCAC
Conservation ********** ********** ********** ********** ********** **********

             ....|....| ....|....| ....|....| ....|....| ....|....| ....|....|
                  2225       2235       2245       2255       2265       2275
2d_SalI      AAAAGAACCA AAATGGATTA TGAAAAACTT CTAAATATGA AATGTGATGA TATACCCGAA
2d_Thai      AAAAGAACCA AAATGGATTA TGAAAAACTT CTAAATATGA AATGTGATGA TATACCCGAA
Conservation ********** ********** ********** ********** ********** **********

             ....|....| ....|....| ....|....| ....|....| ....|....| ....|....|
                  2285       2295       2305       2315       2325       2335
2d_SalI      ATAATAAAAA AGTTGAATAC AGAATTAAAT AATCTCAAAA ATTTAGAAAA AAATATTGTA
2d_Thai      ATAATAAAAA AGTTGAATAC AGAATTAAAT AATCTCAAAA ATTTAGAAAA AAATATTGTA
Conservation ********** ********** ********** ********** ********** **********

             ....|....| ....|....| ....|....| ....|....| ....|....| ....|....|
                  2345       2355       2365       2375       2385       2395
2d_SalI      GAAGAACAGA CTAAAAACAT TAATAAATAT GTTTCAGATT CATTTGATAA CCTAACTGAT
2d_Thai      GAAGAACAGA CTAAAAACAT TAATAAATAT GTTTCAGATT CATTTGATAA CCTAACTGAT
Conservation ********** ********** ********** ********** ********** **********
```

```
             ....|....| ....|....| ....|....| ....|....| ....|....| ....|....|
                  2405       2415       2425       2435       2445       2455
2d_SalI      GAAATGAACC GTTTAAGAAA TAGCTTGAAT GAATATAAGC AGGATGAAGG TAAGCTCCAA
2d_Thai      GAAATGAACC GTTTAAGAAA TAGCTTGAAT GAATATAAGC AGGATGAAGG TAAGCTCCAA
Conservation ********** ********** ********** ********** ********** **********

             ....|....| ....|....| ....|....| ....|....| ....|....| ....|....|
                  2465       2475       2485       2495       2505       2515
2d_SalI      ACATATAAAG GTAGTATAAA TGAAAGAAAA GACAAATTCC TCAATACTTC AAGCGAAAAA
2d_Thai      ACATATAAAG GTAGTATAAA TGAAAGAAAA GACAAATTCC TCAATACTTC AAGCGAAAAA
Conservation ********** ********** ********** ********** ********** **********

             ....|....| ....|....| ....|....| ....|....| ....|....| ....|....|
                  2525       2535       2545       2555       2565       2575
2d_SalI      GAGGAAGATA TTCCAGAGGG ACAAAATATT TATGATGAAT ATAAAAGTCA TAAAGATCTT
2d_Thai      GAGGAAGATA TTCCAGAGGG ACAAAATATT TATGATGAAT ATAAAAGTCA TAAAGATCTT
Conservation ********** ********** ********** ********** ********** **********

             ....|....| ....|....| ....|....| ....|....| ....|....| ....|....|
                  2585       2595       2605       2615       2625       2635
2d_SalI      ATGGTTAACA AAGAACTTAA ATTATCCAGT GATATTAATG AATTCAAGGA AAATATAAAA
2d_Thai      ATGGTTAACA AAGAACTTAA ATTATCCAGT GATATTAATG AATTCAAGGA AAATATAAAA
Conservation ********** ********** ********** ********** ********** **********

             ....|....| ....|....| ....|....| ....|....| ....|....| ....|....|
                  2645       2655       2665       2675       2685       2695
2d_SalI      AAAGTCCAAA CAAAAATAGA GGCCTTCAGC AGTGTAATGC AAAAATTAGG AGCGAACGCC
2d_Thai      AAAGTCCAAA CAAAAATAGA GGCCTTCAGC AGTGTAATGC AAAAATTAGG AGCGAACGCC
Conservation ********** ********** ********** ********** ********** **********

             ....|....| ....|....| ....|....| ....|....| ....|....| ....|....|
                  2705       2715       2725       2735       2745       2755
2d_SalI      GATGAACAAC ATCAAGAAAC TATAAATTCT TTGAAGAACT TTAATACTAA AATCGAAGGT
2d_Thai      GATGAACAAC ATCAAGAAAC TATAAATTCT TTGAAGAACT TTAATACTAA AATCGAAGGT
Conservation ********** ********** ********** ********** ********** **********

             ....|....| ....|....| ....|....| ....|....| ....|....| ....|....|
                  2765       2775       2785       2795       2805       2815
2d_SalI      TTAAAATTAA GTGAACTTGA AGGTGAATTT AAAATTAACA ATGAATCAGC TGCCAAATTA
2d_Thai      TTAAAATTAA GTGAACTTGA AGGTGAATTT AAAATTAACA ATGAATCAGC TGCCAAATTA
Conservation ********** ********** ********** ********** ********** **********

             ....|....| ....|....| ....|....| ....|....| ....|....| ....|....|
                  2825       2835       2845       2855       2865       2875
2d_SalI      TATAATCAAA TTGAAAATAC AATGAAAAAT ATAGACACAA TTAAATCCTT AAATTATACT
2d_Thai      TATAATCAAA TTGAAAATAC AATGAAAAAT ATAGACACAA TTAAATCCTT AAATTATACT
Conservation ********** ********** ********** ********** ********** **********

             ....|....| ....|....| ....|....| ....|....| ....|....| ....|....|
                  2885       2895       2905       2915       2925       2935
2d_SalI      AGAAACAGCG CCAACAATAG CAGAGAATTA ATGGTAAAAA TAGTAAAAGA TAAAAATGAT
2d_Thai      AGAAACAGCG CCAACAATAG CAGAGAATTA ATGGAAAAAA TAGTAAAAGA TAAAAATGAT
Conservation ********** ********** ********** ****:***** ********** **********

             ....|....| ....|....| ....|....| ....|....| ....|....| ....|....|
                  2945       2955       2965       2975       2985       2995
2d_SalI      TTAATAAAAA AACTAGATCA ACAGTCTGAA GAAATAAAAC AATACACATT AATAGAAGAA
2d_Thai      TTAATAAAAA AACTAGATCA ACAGTCTGAA GAAATAAAAC AATACACATT AATAGAAGAA
Conservation ********** ********** ********** ********** ********** **********
```

```
             ....|....| ....|....| ....|....| ....|....| ....|....| ....|....|
                 3005       3015       3025       3035       3045       3055
2d_SalI      AAAGAAAAGT CACCCCTTCT AAGCGCTTTA AATGAAGAAC AAAATCATGT AGAAAGTGAA
2d_Thai      GAAGAAAAGT CACCCCTTCT AAGCGCTTTA AATGAAGAAC AAAATCATGT AGAAAGTGAA
Conservation .********* ********** ********** ********** ********** **********

             ....|....| ....|....| ....|....| ....|....| ....|....| ....|....|
                 3065       3075       3085       3095       3105       3115
2d_SalI      ATTCCTAAAG AGTCGATAGA CAAATTAAAC ACAGAAATAA ATGCTATACT GGAATATTAC
2d_Thai      ATTCCTAAAG AGTCGATAGA CAAATTAAAC ACAGAAATAA ATGCTATACT GGAATATTAC
Conservation ********** ********** ********** ********** ********** **********

             ....|....| ....|....| ....|....| ....|....| ....|....| ....|....|
                 3125       3135       3145       3155       3165       3175
2d_SalI      AACAGTTCAA AGGGTAATTT TAATGAGGAC ACGGAAATAA AATTGGAAAA GTTAGACAAT
2d_Thai      AACAGTTCAA AGGGTAATTT TAATGAGGAC ACGGAAATAA AATTGGAAAA GTTAGACAAT
Conservation ********** ********** ********** ********** ********** **********

             ....|....| ....|....| ....|....| ....|....| ....|....| ....|....|
                 3185       3195       3205       3215       3225       3235
2d_SalI      TTTAAAACTC AATGTGATAA TATTAAGCAA GAAATAGAAA CATTAAACAC TAAATATAAA
2d_Thai      TTTAAAACTC AATGTGATAA TATTAAGCAA GAAATAGAAA CATTAAACAC TAAATATAAA
Conservation ********** ********** ********** ********** ********** **********

             ....|....| ....|....| ....|....| ....|....| ....|....| ....|....|
                 3245       3255       3265       3275       3285       3295
2d_SalI      GTGTTAGAAA AAAGGATAGA CACCCTAATT GATGAACAAC ATGCTAAAAT TGTGACGTTG
2d_Thai      GTGTTAGAAA AAAGGATAGA CACCCTAATT GATGAACAAC ATGCTAAAAT TGTGACGTTG
Conservation ********** ********** ********** ********** ********** **********

             ....|....| ....|....| ....|....| ....|....| ....|....| ....|....|
                 3305       3315       3325       3335       3345       3355
2d_SalI      ACAGATAAGC ACATAACAAC CAAAGATAAT ATGATTAATC AAAAGATTGA ACATAGCACA
2d_Thai      ACAGATAAGC ACATAACAAC CAAAGATAAT ATGATTAATC AAAAGATTGA ACATAGCACA
Conservation ********** ********** ********** ********** ********** **********

             ....|....| ....|....| ....|....| ....|....| ....|....| ....|....|
                 3365       3375       3385       3395       3405       3415
2d_SalI      AAATCGCTAG ATGATATGAA AACTAAGTTG AATTCTCTCA AATTTAACGA TGATATCAGA
2d_Thai      AAATCGCTAG ATGATATGAA AACTAAGTTG AATTCTCTCA AATTTAACGA TGATATCAGA
Conservation ********** ********** ********** ********** ********** **********

             ....|....| ....|....| ....|....| ....|....| ....|....| ....|....|
                 3425       3435       3445       3455       3465       3475
2d_SalI      AAGAATGAAA ATGTCGAAAT TCAAGGTAAA ATACAGGAAT TTGAAAAAAT GGTTCAAACT
2d_Thai      AAGAATGAAA ATGTCGAAAT TCAAGGTAAA ATACAGGAAT TTGAAAAAAT GGTTCAAACT
Conservation ********** ********** ********** ********** ********** **********

             ....|....| ....|....| ....|....| ....|....| ....|....| ....|....|
                 3485       3495       3505       3515       3525       3535
2d_SalI      ATAGAGGAAA GTATAAATAA AAATAAAACT AAAATATATG AAATTAAAGG AGTATACGAT
2d_Thai      ATAGAGGAAA GTATAAATAA AAATAAAACT AAAATATATG AAATTAAAGG AGTATACGAT
Conservation ********** ********** ********** ********** ********** **********

             ....|....| ....|....| ....|....| ....|....| ....|....| ....|....|
                 3545       3555       3565       3575       3585       3595
2d_SalI      GTGTACAAGG GTGAATTCAA TGAAGAAAAA AATAAAAGCA CAAAAATCGT AGAAAAAAAA
2d_Thai      GTGTACAAGG GTGAATTCAA TGAAGAAAAA AATAAAAGCA CAAAAATCGT AGAAAAAAAA
Conservation ********** ********** ********** ********** ********** **********
```

```
             ....|....| ....|....| ....|....| ....|....| ....|....| ....|....|
                  3605       3615       3625       3635       3645       3655
2d_SalI      AATAGCATGG AAAAAGTTTA TAACAAAATG GAAGATACTC TTAAAAAATT AGAAGATATA
2d_Thai      AATAGCATGG AAAAAGTTTA TAACAAAATG GAAGATACTC TTAAAAAATT AGAAGATATA
Conservation ********** ********** ********** ********** ********** **********

             ....|....| ....|....| ....|....| ....|....| ....|....| ....|....|
                  3665       3675       3685       3695       3705       3715
2d_SalI      GATAACGAAA AAAATATATT AAATGAAATA GAAAAGGCTG AAGTACTATA CAGGAAAGTT
2d_Thai      GATAACGAAA AAAATATATT AAATGAAATA GAAAAGGCTG AAGTACTATA CAGGAAAGTT
Conservation ********** ********** ********** ********** ********** **********

             ....|....| ....|....| ....|....| ....|....| ....|....| ....|....|
                  3725       3735       3745       3755       3765       3775
2d_SalI      TTCATTCATA ATACTGTTCA TATGATGGAT AATGAAACTA AAAAGTCTAA AAATTTGATG
2d_Thai      TTCATTCATA ATACTGTTCA TATGATGGAT AATGAAACTA AAAAGTCTAA AAATTTGATG
Conservation ********** ********** ********** ********** ********** **********

             ....|....| ....|....| ....|....| ....|....| ....|....| ....|....|
                  3785       3795       3805       3815       3825       3835
2d_SalI      GACCAAATCG AATTGTCTAA AAGAGAAATT GAGGAAACAA AGAAGCAAAT GCTTGAATAC
2d_Thai      GACCAAATCG AATTGTCTAA AAGAGAAATT GAGGAAACAA AGAAGCAAAT GCTTGAATAC
Conservation ********** ********** ********** ********** ********** **********

             ....|....| ....|....| ....|....| ....|....| ....|....| ....|....|
                  3845       3855       3865       3875       3885       3895
2d_SalI      AAAAATGATG AGGTATCTAA TTATGATTAT ATGAAATATT ACAACCAGGC TACAGAGAGT
2d_Thai      AAAAATGATG AGGTATCTAA TTATGATTAT ATGAAATATT ACAACCAGGC TACAGAGAGT
Conservation ********** ********** ********** ********** ********** **********

             ....|....| ....|....| ....|....| ....|....| ....|....| ....|....|
                  3905       3915       3925       3935       3945       3955
2d_SalI      AATGCTAAAA TAAAAGAAAC TTTTAAAAAT GCTACAGATA AGAAAGAAAA GGCTGAAAAT
2d_Thai      AATGCTAAAA TAAAAGAAAC TTTTAAAAAT GCTACAGATA AGAAAGAAAA GGCTGAAAAT
Conservation ********** ********** ********** ********** ********** **********

             ....|....| ....|....| ....|....| ....|....| ....|....| ....|....|
                  3965       3975       3985       3995       4005       4015
2d_SalI      ATCGAAAAAG TAAGTGAAAT CGATGACATT AAAAACCAAG TTAATCATAA TTTACATCAG
2d_Thai      ATCGAAAAAG TAAGTGAAAT CGATGACATT AAAAACCAAG TTAATCATAA TTTACATCAG
Conservation ********** ********** ********** ********** ********** **********

             ....|....| ....|....| ....|....| ....|....| ....|....| ....|....|
                  4025       4035       4045       4055       4065       4075
2d_SalI      ATCCAATCAG AAAATAGTTC TATAGAGGAG ATGTTAAAAC AAATTAATAA TATGAAAGAT
2d_Thai      ATCCAATCAG AAAATAGTTC TATAGAGGAG ATGTTAAAAC AAATTAATAA TATGAAAGAT
Conservation ********** ********** ********** ********** ********** **********

             ....|....| ....|....| ....|....| ....|....| ....|....| ....|....|
                  4085       4095       4105       4115       4125       4135
2d_SalI      TTATTAAAAA CGAACAGTTC TAAAGGAATA TCGGATACAG TATTAAGTAG TACCAAAAAT
2d_Thai      TTATTAAAAA CGAACAGTTC TAAAGGAATA TCGGATACAG TATTAAGTAG TACCAAAAAT
Conservation ********** ********** ********** ********** ********** **********

             ....|....| ....|....| ....|....| ....|....| ....|....| ....|....|
                  4145       4155       4165       4175       4185       4195
2d_SalI      GCAGAAGAAT TTGGCAAACA AGCAATAAGT GAATTTAATA AAACGAATGA TGTAATACAA
2d_Thai      GCAGAAGAAT TTGGCAAACA AGCAATAAGT GAATTTAATA AAACGAATGA TGTAATACAA
Conservation ********** ********** ********** ********** ********** **********
```

```
             ....|....| ....|....| ....|....| ....|....| ....|....| ....|....|
                  4205       4215       4225       4235       4245       4255
2d_SalI      GCAATCAGAG ATTTGATAAC TAAGGCTGAA GCACATAAAA ATTTAATCAA TATAAATTTA
2d_Thai      GCAATCAGAG ATTTGATAAC TAAGGCTGAA GCACATAAAA ATTTAATCAA TATAAATTTA
Conservation ********** ********** ********** ********** ********** **********

             ....|....| ....|....| ....|....| ....|....| ....|....| ....|....|
                  4265       4275       4285       4295       4305       4315
2d_SalI      GAAGATGAAA AAATAAATGC CGAAGTGAAA GAAATTGAAA AAATTAAGGA ACAAATTGCC
2d_Thai      GAAGATGAAA AAATAAATGC CGAAGTGAAA GAAATTGAAA AAATTAAGGA ACAAATTGCC
Conservation ********** ********** ********** ********** ********** **********

             ....|....| ....|....| ....|....| ....|....| ....|....| ....|....|
                  4325       4335       4345       4355       4365       4375
2d_SalI      AATAAGAAAG AAGAAATAGA ATCCCATCTG AGCAAGGCAA AAGAATTTAA AGAAAAATGT
2d_Thai      AATAAGAAAG AAGAAATAGA ATCCCATCTG AGCAAGGCAA AAGAATTTAA AGAAAAATGT
Conservation ********** ********** ********** ********** ********** **********

             ....|....| ....|....| ....|....| ....|....| ....|....| ....|....|
                  4385       4395       4405       4415       4425       4435
2d_SalI      TCCTCAGAAA CAAGTAACGC CAAAAGAGGT AAAAGTAAAA TAGAATTTTT GCAAAGTCGT
2d_Thai      TCCTCAGAAA CAAGTAACGC CAAAAGAGGT AAAAGTAAAA TAGAATTTTT GCAAAGTCGT
Conservation ********** ********** ********** ********** ********** **********

             ....|....| ....|....| ....|....| ....|....| ....|....| ....|....|
                  4445       4455       4465       4475       4485       4495
2d_SalI      GAAGGCAGTT CGGAAGAGGT AGATATGAAG GAGATAGAAG AAAATATTAC CAAAGCAGAA
2d_Thai      GAAGGCAGTT CGGAAGAGGT AGATATGAAG GAGATAGAAG AAAATATTAC CAAAGCAGAA
Conservation ********** ********** ********** ********** ********** **********

             ....|....| ....|....| ....|....| ....|....| ....|....| ....|....|
                  4505       4515       4525       4535       4545       4555
2d_SalI      GGATATTTAA ATCAAGCCAT TGCTGCAGAA ACAGAAGCTA ATAAGAATGT AGAATTGTTC
2d_Thai      GGATATTTAA ATCAAGCCAT TGCTGCAGAA ACAGAAGCTA ATAAGAATGT AGAATTGTTC
Conservation ********** ********** ********** ********** ********** **********

             ....|....| ....|....| ....|....| ....|....| ....|....| ....|....|
                  4565       4575       4585       4595       4605       4615
2d_SalI      GGTGAACTTC AAAAAAATAT CTGTGACATT TTCGATGAAT CATCAATTTT AGGAATAGAG
2d_Thai      GGTGAACTTC AAAAAAATAT CTGTGACATT TTCGATGAAT CATCAATTTT AGGAATAGAG
Conservation ********** ********** ********** ********** ********** **********

             ....|....| ....|....| ....|....| ....|....| ....|....| ....|....|
                  4625       4635       4645       4655       4665       4675
2d_SalI      ACTACATCAA AGAAAAAAAT TAATAAAGCA ACAGAAATAA TGGAAGAAAT TAAAAGGAAA
2d_Thai      ACTACATCAA AGAAAAAAAT TAATAAAGCA ACAGAAATAA TGGAAGAAAT TAAAAGGAAA
Conservation ********** ********** ********** ********** ********** **********

             ....|....| ....|....| ....|....| ....|....| ....|....| ....|....|
                  4685       4695       4705       4715       4725       4735
2d_SalI      AATTTTGAAA TTCAAGGCGA GGTGAAAATT TTCCATGAAA CACTCGTTAA ATTGAAAGAA
2d_Thai      AATTTTGAAA TTCAAGGCGA GGTGAAAATT TTCCATGAAA CACTCGTTAA ATTGAAAGAA
Conservation ********** ********** ********** ********** ********** **********

             ....|....| ....|....| ....|....| ....|....| ....|....| ....|....|
                  4745       4755       4765       4775       4785       4795
2d_SalI      ACACACCCTG ATAACAACGT AGATGCTGAA CTTAACAATG CGAAATCGAC CAATGCAAAT
2d_Thai      ACACACCCTG ATAACAACGT AGATGCTGAA CTTAACAATG CGAAATCGAC CAATGCAAAT
Conservation ********** ********** ********** ********** ********** **********
```

```
              ....|....| ....|....| ....|....| ....|....| ....|....| ....|....|
                  4805       4815       4825       4835       4845       4855
2d_SalI       GTACTTATAC AAACTAACCT AGAAATGGTG GAACATAACT TATCCGTAAT AGCTAAAATT
2d_Thai       GTACTTATAC AAACTAACCT AGAAATGGTG GAACATAACT TATCCGTAAT AGCTAAAATT
Conservation  ********** ********** ********** ********** ********** **********

              ....|....| ....|....| ....|....| ....|....| ....|....| ....|....|
                  4865       4875       4885       4895       4905       4915
2d_SalI       AAACAGGAGG GAGAAAATAT ATATAATAGA GCCTCATCTA CGATAAATTC CATGACAGAA
2d_Thai       AAACAGGAGG GAGAAAATAT ATATAATAGA GCCTCATCTA CGATAAATTC CATGACAGAA
Conservation  ********** ********** ********** ********** ********** **********

              ....|....| ....|....| ....|....| ....|....| ....|....| ....|....|
                  4925       4935       4945       4955       4965       4975
2d_SalI       ATCTCCAAAA ATACTGAAAA AAAAACATTA GACAAGGCAA AAAGTGACGA ATCTAAATAT
2d_Thai       ATCTCCAAAA ATACTGAAAA AAAAACATTA GACAAGGCAA AAAGTGACGA ATCTAAATAT
Conservation  ********** ********** ********** ********** ********** **********

              ....|....| ....|....| ....|....| ....|....| ....|....| ....|....|
                  4985       4995       5005       5015       5025       5035
2d_SalI       ATATCCTATT TAACCAAAAT AACTTCAGAA AGAGATCTAA TCATTGAAGA AAGGAATAAA
2d_Thai       ATATCCTATT TAACCAAAAT AACTTCAGAA AGAGATCTAA TCATTGAAGA AAGGAATAAA
Conservation  ********** ********** ********** ********** ********** **********

              ....|....| ....|....| ....|....| ....|....| ....|....| ....|....|
                  5045       5055       5065       5075       5085       5095
2d_SalI       CTAAATGGTA TAAGCCCCAA TATAATAAGT ATAGAAAAGG AATTGAATAA AGCCAGAAAG
2d_Thai       CTAAATGGTA TAAGCCCCAA TATAATAAGT ATAGAAAAGG AATTGAATAA AGCCAGAAAG
Conservation  ********** ********** ********** ********** ********** **********

              ....|....| ....|....| ....|....| ....|....| ....|....| ....|....|
                  5105       5115       5125       5135       5145       5155
2d_SalI       GATTACGAAA TTGGACTTTT GCAAAAGATA GATGAAATAG GCAAAAACAG AAAAAAAGAA
2d_Thai       GATTACGAAA TTGGACTTTT GCAAAAGATA GATGAAATAG GCAAAAACAA AAAAAAAAAA
Conservation  ********** ********** ********** ********** *********. *******.**

              ....|....| ....|....| ....|....| ....|....| ....|....| ....|....|
                  5165       5175       5185       5195       5205       5215
2d_SalI       TATG-ATTTG ACAAAAGAAT CAATAAAATT AACATTGAGC AACTTTTCTT CCCATTTCAA
2d_Thai       TATTGATTTG ACAAAAGAAT CAATAAAATT AACATTGAGC AACTTTTCTT CCCATTTCAA
Conservation  ***. ***** ********** ********** ********** ********** **********

              ....|....| ....|....| ....|....| ....|....| ....|....| ....|....|
                  5225       5235       5245       5255       5265       5275
2d_SalI       AGGATTTGAT TTAAAGGAAT ATGTCTTTAA TAAAAATATA AATGACTATG AACAAAAGAT
2d_Thai       AGGATTTGAT TTAAAGGAAT ATGTCTTTAA TAAAAATATA AATGACTATG AACAAAAGAT
Conservation  ********** ********** ********** ********** ********** **********

              ....|....| ....|....| ....|....| ....|....| ....|....| ....|....|
                  5285       5295       5305       5315       5325       5335
2d_SalI       GAAAGAAATA CATGACAAAT ATGACAAATC GATAAATAAA ATTAGTAAAA ACTTGAAAAA
2d_Thai       GAAAGAAATA CATGACAAAT ATGACAAATC GATAAATAAA ATTAGTAAAA ACTTGAAAAA
Conservation  ********** ********** ********** ********** ********** **********

              ....|....| ....|....| ....|....| ....|....| ....|....| ....|....|
                  5345       5355       5365       5375       5385       5395
2d_SalI       GGCTGAAGAG GATAATACAA ACTATACCCT AGCGAATGAG CTGAGGAAAG AAGCGCAACA
2d_Thai       GGCTGAAGAG GATAATACAA ACTATACCCT AGCGAATGAG CTGAGGAAAG AAGCGCAACA
Conservation  ********** ********** ********** ********** ********** **********
```

```
             ....|....| ....|....| ....|....| ....|....| ....|....| ....|....|
                  5405       5415       5425       5435       5445       5455
2d_SalI      AGAAAAAGCA GAACTTACAA ATATACAAGA AGAAGTAAAT AAATATTTAA GGGATATAAA
2d_Thai      AGAAAAAGCA GAACTTACAA ATATACAAGA AGAAGTAAAT AAATATTTAA GGGATATAAA
Conservation ********** ********** ********** ********** ********** **********

             ....|....| ....|....| ....|....| ....|....| ....|....| ....|....|
                  5465       5475       5485       5495       5505       5515
2d_SalI      AAAATGGGAA TCATTCAGAT TTATACTTGA TATGAAAGAC AATTTAAGTA ATATAAATAC
2d_Thai      AAAATGGGAA TCATTCAGAT TTATACTTGA TATGAAAGAC AATTTAAGTA ATATAAATAC
Conservation ********** ********** ********** ********** ********** **********

             ....|....| ....|....| ....|....| ....|....| ....|....| ....|....|
                  5525       5535       5545       5555       5565       5575
2d_SalI      ACTGATTAAA CAAGAAGAAT TAAAGGTTAA TGATGGATAT GAATACATTA AACAATTAGT
2d_Thai      ACTGATTAAA CAAGAAGAAT TAAAGGTTAA TGATGGATAT GAATACATTA AACAATTAGT
Conservation ********** ********** ********** ********** ********** **********

             ....|....| ....|....| ....|....| ....|....| ....|....| ....|....|
                  5585       5595       5605       5615       5625       5635
2d_SalI      TGACAATATT AAAGAGTCAA ATGATGAAAG CAGTATATCA GATAACTTAC AAAAAGGAAA
2d_Thai      TGACAATATT AAAGAGTCAA ATGATGAAAG CAGTATATCA GATAACTTAC AAAAAGGAAA
Conservation ********** ********** ********** ********** ********** **********

             ....|....| ....|....| ....|....| ....|....| ....|....| ....|....|
                  5645       5655       5665       5675       5685       5695
2d_SalI      AGAAAAAAAT GTAGAAATAC AGAACAAAAT GCAATCTACG TATAAAAACA AGGCAAAAGT
2d_Thai      AGAAAAAAAT GTAGAAATAC AGAACAAAAT GCAATCTACG TATAAAAACA AGGCAAAAGT
Conservation ********** ********** ********** ********** ********** **********

             ....|....| ....|....| ....|....| ....|....| ....|....| ....|....|
                  5705       5715       5725       5735       5745       5755
2d_SalI      TATTTTAGGA CATATAATTG ACTCCGCAAA ATTTATAGAT ATTAATATAA TTACAAAATC
2d_Thai      TATTTTAGGA CATATAATTG ACTCCGCAAA ATTTATAGAT ATTAATATAA TTACAAAATC
Conservation ********** ********** ********** ********** ********** **********

             ....|....| ....|....| ....|....| ....|....| ....|....| ....|....|
                  5765       5775       5785       5795       5805       5815
2d_SalI      ACCACTAAAC GAATTGTCTT CAGAATCACA TTTGATGAAT GCAGGAGAAT TAAAATTTCA
2d_Thai      ACCACTAAAC GAATTGTCTT CAGAATCACA TTTGATGAAT GCAGGAGAAT TAAAATTTCA
Conservation ********** ********** ********** ********** ********** **********

             ....|....| ....|....| ....|....| ....|....| ....|....| ....|....|
                  5825       5835       5845       5855       5865       5875
2d_SalI      GCAAGAAAAT AAAGTAACAT TAGAAACAGA ACATATGACA AACAATAAGA GCGAATTAGA
2d_Thai      GCAAGAAAAT AAAGTAACAT TAGAAACAGA ACATATGACA AACAATAAGA GCGAATTAGA
Conservation ********** ********** ********** ********** ********** **********

             ....|....| ....|....| ....|....| ....|....| ....|....| ....|....|
                  5885       5895       5905       5915       5925       5935
2d_SalI      TGTTTATAAA AATATACGCG ATGCTTACAA CATCGTTGTG GAGATAATTG CATATTCAGA
2d_Thai      TGTTTATAAA AATATACGCG ATGCTTACAA CATCGTTGTG GAGATAATTG CATATTCAGA
Conservation ********** ********** ********** ********** ********** **********

             ....|....| ....|....| ....|....| ....|....| ....|....| ....|....|
                  5945       5955       5965       5975       5985       5995
2d_SalI      TAAAATAGAC ACAAAACAAA GACAAAGTAC ACAATTATTG GATGATGGAA ATGATATATA
2d_Thai      TAAAATAGAC ACAAAACAAA GACAAAGTAC ACAATTATTG GATGATGGAA ATGATATATA
Conservation ********** ********** ********** ********** ********** **********
```

```
             ....|....| ....|....| ....|....| ....|....| ....|....| ....|....|
                  6005       6015       6025       6035       6045       6055
2d_SalI      TCTTACGCTA AAGTCAATAA ACGAGCTAAA AAATAAAATA AACTCTGTAA AAAGTAAAGA
2d_Thai      TCTTACGCTA AAGTCAATAA ACGAGCTAAA AAATAAAATA AACTCTGTAA AAAGTAAAGA
Conservation ********** ********** ********** ********** ********** **********

             ....|....| ....|....| ....|....| ....|....| ....|....| ....|....|
                  6065       6075       6085       6095       6105       6115
2d_SalI      AAATGCTATT TCAGGCAAAA TAGATAATAT TTCAAACAAA CTTAATGAGT TAAACAAAAT
2d_Thai      AAATGCTATT TCAGGCAAAA TAGATAATAT TTCAAACAAA CTTAATGAGT TAAACAAAAT
Conservation ********** ********** ********** ********** ********** **********

             ....|....| ....|....| ....|....| ....|....| ....|....| ....|....|
                  6125       6135       6145       6155       6165       6175
2d_SalI      TACATGTGAT GATAAAAGTT ACGATAATAT TTTAGACATA ATAAAACATA CAGAATTAAA
2d_Thai      TACATGTGAT GATAAAAGTT ACGATAATAT TTTAGACATA ATAAAACATA CAGAATTAAA
Conservation ********** ********** ********** ********** ********** **********

             ....|....| ....|....| ....|....| ....|....| ....|....| ....|....|
                  6185       6195       6205       6215       6225       6235
2d_SalI      AACTATACGT GATTCTTTTA AACAAGAAAA AATTAACAAA AAGAGCGATT CTAAATTGGA
2d_Thai      AACTATACGT GATTCTTTTA AACAAGAAAA AATTAACAAA AAGAGCGATT CTAAATTGGA
Conservation ********** ********** ********** ********** ********** **********

             ....|....| ....|....| ....|....| ....|....| ....|....| ....|....|
                  6245       6255       6265       6275       6285       6295
2d_SalI      AAAAATAAAG GAAGAATTTG AAAATTCAAA AAATACATTA AAAAATATAG AAGAAGAAGT
2d_Thai      AAAAATAAAG GAAGAATTTG AAAATTCAAA AAATACATTA AAAAATATAG AAGAAGAAGT
Conservation ********** ********** ********** ********** ********** **********

             ....|....| ....|....| ....|....| ....|....| ....|....| ....|....|
                  6305       6315       6325       6335       6345       6355
2d_SalI      TAATGCTCTA AAGGCTAGTT TGGTAAAACA TGATAATATA CAAAGCAGAA ACAAGCCAAT
2d_Thai      TAATGCTCTA AAGGCTAGTT TGGTAAAACA TGATAATATA CAAAGCAGAA ACAAGCCAAT
Conservation ********** ********** ********** ********** ********** **********

             ....|....| ....|....| ....|....| ....|....| ....|....| ....|....|
                  6365       6375       6385       6395       6405       6415
2d_SalI      TGATGATGTG CTAATCGAAA TAGAAAAAAC AGAAAAAGGT ATAGATAGTT TTAGTGCCTC
2d_Thai      TGATGATGTG CTAATCGAAA TAGAAAAAAC AGAAAAAGGT ATAGATAGTT TTAGTGCCTC
Conservation ********** ********** ********** ********** ********** **********

             ....|....| ....|....| ....|....| ....|....| ....|....| ....|....|
                  6425       6435       6445       6455       6465       6475
2d_SalI      CCTTGATGAG ATGTTGAAGA AAGGAAGAGA ATGCGAAATA TTTAGGTATA CATCCATAAA
2d_Thai      CCTTGATGAG ATGTTGAAGA AAGGAAGAGA ATGCGAAATA TTTAGGTATA CATCCATAAA
Conservation ********** ********** ********** ********** ********** **********

             ....|....| ....|....| ....|....| ....|....| ....|....| ....|....|
                  6485       6495       6505       6515       6525       6535
2d_SalI      AGATGGTGTT ATCGCAAAAA TAAATGATGA CGCAGAATTA ATCGATAATA TACACAATAA
2d_Thai      AGATGGTGTT ATCGCAAAAA TAAATGATGA CGCAGAATTA ATCGATAATA TACACAATAA
Conservation ********** ********** ********** ********** ********** **********

             ....|....| ....|....| ....|....| ....|....| ....|....| ....|....|
                  6545       6555       6565       6575       6585       6595
2d_SalI      TACTAATGAA TATTTGACAT ATGTTCAAAA AAATTATAGT GAAACAGCAG AGGATGTTCG
2d_Thai      TACTAATGAA TATTTGACAT ATGTTCAAAA AAATTATAGT GAAACAGCAG AGGATGTTCG
Conservation ********** ********** ********** ********** ********** **********
```

```
             ....|....| ....|....| ....|....| ....|....| ....|....| ....|....|
                  6605       6615       6625       6635       6645       6655
2d_SalI      TACATTAAAT CAACATTTCA TGACAGAAAC GATAAGCGAT CATGCGCCAA CTAATTACGA
2d_Thai      TACATTAAAT CAACATTTCA TGACAGAAAC GATAAGCGAT CATGCGCCAA CTAATTACGA
Conservation ********** ********** ********** ********** ********** **********

             ....|....| ....|....| ....|....| ....|....| ....|....| ....|....|
                  6665       6675       6685       6695       6705       6715
2d_SalI      AACATCAAAT AAATCTTATG AAGAGATAAC TGAAGCTGTT AATAATTCCA AAGCAATAAT
2d_Thai      AACATCAAAT AAATCTTATG AAGAGATAAC TGAAGCTGTT AATAATTCCA AAGCAATAAT
Conservation ********** ********** ********** ********** ********** **********

             ....|....| ....|....| ....|....| ....|....| ....|....| ....|....|
                  6725       6735       6745       6755       6765       6775
2d_SalI      AGATAATATA CAAAATTCAA TTATACAAGT TAACGAAAAA ACGGAAATTA GTTCTTTAGA
2d_Thai      AGATAATATA CAAAATTCAA TTATACAAGT TAACGAAAAA ACGGAAATTA GTTCTTTAGA
Conservation ********** ********** ********** ********** ********** **********

             ....|....| ....|....| ....|....| ....|....| ....|....| ....|....|
                  6785       6795       6805       6815       6825       6835
2d_SalI      AAATAGCGCA GAAAAAAATAG AAAAGCTTTA TAAAGAATTA CAGGATAAAA AAAACACCAT
2d_Thai      AAATAGCGCA GAAAAAAATAG AAAACCTTTA TAAAGAATTA CAGGATAAAA AAAACACCAT
Conservation ********** ********** **** ***** ********** ********** **********

             ....|....| ....|....| ....|....| ....|....| ....|....| ....|....|
                  6845       6855       6865       6875       6885       6895
2d_SalI      AAACGAAATT TATAAAAAGG CTAACATAGT TAAATTGCAA GAAATAAAAT CAGATGCTGA
2d_Thai      AAACGAAATT TATAAAAAGG CTAACATAGT TAAATTGCAA GAAATAAAAT CAGATGCTGA
Conservation ********** ********** ********** ********** ********** **********

             ....|....| ....|....| ....|....| ....|....| ....|....| ....|....|
                  6905       6915       6925       6935       6945       6955
2d_SalI      TAAATATTTG GATGTAGCTA ACATATTTAA TAATGTATTG GACGGTCAAA AATCAAGAAT
2d_Thai      TAAATATTTG GATGTAGCTA ACATATTTAA TAATGTATTG GACGGTCAAA AATCAAGAAT
Conservation ********** ********** ********** ********** ********** **********

             ....|....| ....|....| ....|....| ....|....| ....|....| ....|....|
                  6965       6975       6985       6995       7005       7015
2d_SalI      AATAAATAAC CTAGGTAAAA TAGCCCAAGT TAAAGAAACA ATAAACCTAA AACTGAAGAA
2d_Thai      AATAAATAAC CTAGGTAAAA TAGCCCAAGT TAAAGAAACA ATAAACCTAA AACTGAAGAA
Conservation ********** ********** ********** ********** ********** **********

             ....|....| ....|....| ....|....| ....|....| ....|....| ....|....|
                  7025       7035       7045       7055       7065       7075
2d_SalI      ATTGGTTGAG ACTGACAATA CATTTACGAC AGAGTCTATT AACAGGTTTG GAGAAATTTA
2d_Thai      ATTGGTTGAG ACTGACAATA CATTTACGAC AGAGTCTATT AACAGGTTTG GAGAAATTTA
Conservation ********** ********** ********** ********** ********** **********

             ....|....| ....|....| ....|....| ....|....| ....|....| ....|....|
                  7085       7095       7105       7115       7125       7135
2d_SalI      TAATGACATT AAGACTAGTA TAGATGAACT GGAAATTATA GAACAAACAA ATTATAGCGA
2d_Thai      TAATGACATT AAGACTAGTA TAGATGAACT GGAAATTATA GAACAAACAA ATTATAGCGA
Conservation ********** ********** ********** ********** ********** **********

             ....|....| ....|....| ....|....| ....|....| ....|....| ....|....|
                  7145       7155       7165       7175       7185       7195
2d_SalI      ACAAAATGAT GTAAAAAATC ATAAAGAAAA AATTACATAC TTAATAAGCA GAAGAGAAAC
2d_Thai      ACAAAATGAT GTAAAAAATC ATAAAGAAAA AATTACATAC TTAATAAGCA GAAGAGAAAC
Conservation ********** ********** ********** ********** ********** **********
```

```
             ....|....| ....|....| ....|....| ....|....| ....|....| ....|....|
                 7205       7215       7225       7235       7245       7255
2d_SalI      TTTAGAAAGT GACCTAAAAG GACATGAAGA GGACACAAAT GTGAAAAAAT TAAATGCGAA
2d_Thai      TTTAGAAAGT GACCTAAAAG GACATGAAGA GGACACAAAT GTGAAAAAAT TAAATGCGAA
Conservation ********** ********** ********** ********** ********** **********

             ....|....| ....|....| ....|....| ....|....| ....|....| ....|....|
                 7265       7275       7285       7295       7305       7315
2d_SalI      TACATTAAGT GAAGTAAATA TTGGCATTGC AAGTGCAAAA GAAGCTATAA GCAACTCAGA
2d_Thai      TACATTAAGT GAAGTAAATA TTGGCATTGC AAGTGCAAAA GAAGCTATAA GCAACTCAGA
Conservation ********** ********** ********** ********** ********** **********

             ....|....| ....|....| ....|....| ....|....| ....|....| ....|....|
                 7325       7335       7345       7355       7365       7375
2d_SalI      GGAAGTATTT AAGAAATTAT TAAGAAAAAT AGAAGAAAAT GAAAGATTAT GTAATAATAA
2d_Thai      GGAAGTATTT AAGAAATTAT TAAGAAAAAT AGAAGAAAAT GAAAGATTAT GTAATAATAA
Conservation ********** ********** ********** ********** ********** **********

             ....|....| ....|....| ....|....| ....|....| ....|....| ....|....|
                 7385       7395       7405       7415       7425       7435
2d_SalI      TGATGCAAAG AATTTTATTT CGGATATTAT GCAAAATATA GATGATCTGA ATAAACGTTT
2d_Thai      TGATGCAAAG AATTTTATTT CGGATATTAT GCAAAATATA GATGATCTGA ATAAACGTTT
Conservation ********** ********** ********** ********** ********** **********

             ....|....| ....|....| ....|....| ....|....| ....|....| ....|....|
                 7445       7455       7465       7475       7485       7495
2d_SalI      CACAAAAAAT ATACCAGAAA GAGAAAAACT TTTTGAAATA AAAAGG-ATT ATATATAATG
2d_Thai      CACAAAAAAT ATACCAGAAA GAGAAAAACT TTTTGAAATA GAAAAGGATT ATATATAATG
Conservation ********** ********** ********** ********** .***.* *** **********

             ....|....| ....|....| ....|....| ....|....| ....|....| ....|....|
                 7505       7515       7525       7535       7545       7555
2d_SalI      AAATTAATTC CATTTTCAAT GAAATAAGAA CACTTAACGT TGACGAATTT GTTGAAAAAA
2d_Thai      AAATTAATTC CATTTTCAAT GAAATAAGAA CACTTAACGT TGACGAATTT GTTGAAAAAA
Conservation ********** ********** ********** ********** ********** **********

             ....|....| ....|....| ....|....| ....|....| ....|....| ....|....|
                 7565       7575       7585       7595       7605       7615
2d_SalI      TGTATAAACA AATTGATAGT GAAAAGGAAA GCGTAAATAA CATAACAGAT ACTGATAAGA
2d_Thai      TGTATAAACA AATTGATAGT GAAAAGGAAA GCGTAAATAA CATAACAGAT ACTGATAAGA
Conservation ********** ********** ********** ********** ********** **********

             ....|....| ....|....| ....|....| ....|....| ....|....| ....|....|
                 7625       7635       7645       7655       7665       7675
2d_SalI      TAAGTGTTGC AATTCAAAAT GTTACAAATT ACGATGCAGA AATAAAAGGC TTATTGTCTA
2d_Thai      TAAGTGTTGC AATTCAAAAT GTTACAAATT ACGATGCAGA AATAAAAGGC TTATTGTCTA
Conservation ********** ********** ********** ********** ********** **********

             ....|....| ....|....| ....|....| ....|....| ....|....| ....|....|
                 7685       7695       7705       7715       7725       7735
2d_SalI      GAACTATTAA CGTATTAGAA AGAATAAGTA GAAGGAAAGC GGAAATGGAT ACTTTATTCA
2d_Thai      GAACTATTAA CGTATTAGAA AGAATAAGTA GAAGGAAAGC GGAAATGGAT ACTTTATTCA
Conservation ********** ********** ********** ********** ********** **********

             ....|....| ....|....| ....|....| ....|....| ....|....| ....|....|
                 7745       7755       7765       7775       7785       7795
2d_SalI      ATAGTCTATC ATCGAATAAT AAAAACCTAA ATGAAAATGC AAAAAGATAC ATGAATGATT
2d_Thai      ATAGTCTATC ATCGAATAAT AAAAACCTAA ATGAAAATGC AAAAAGATAC ATGAATGATT
Conservation ********** ********** ********** ********** ********** **********
```

```
             ....|....| ....|....| ....|....| ....|....| ....|....| ....|....|
                  7805       7815       7825       7835       7845       7855
2d_SalI      CAGCTGAAAT AATTAATCAA TTAAATAGCC ATGTTCAAAA AATAACAAAA CTGAAAACAT
2d_Thai      CAGCTGAAAT AATTAATCAA TTAAATAGCC ATGTTCAAAA AATAACAAAA CTGAAAACAT
Conservation ********** ********** ********** ********** ********** **********

             ....|....| ....|....| ....|....| ....|....| ....|....| ....|....|
                  7865       7875       7885       7895       7905       7915
2d_SalI      ATGCAAATGA GGTAATTGAA CAATTACAAA AAGAACTAAC AAGATTATTA GATCAACGTA
2d_Thai      ATGCAAATGA GGTAATTGAA CAATTACAAA AAGAACTAAC AAGATTATTA GATCAACGTA
Conservation ********** ********** ********** ********** ********** **********

             ....|....| ....|....| ....|....| ....|....| ....|....| ....|....|
                  7925       7935       7945       7955       7965       7975
2d_SalI      AAATTGAAAT TCCAGTTGAT GATACTCAAA CTTCTTTAAG TACCGAGGAC GTAAAAGAAG
2d_Thai      AAATTGAAAT TCCAGTTGAT GATACTCAAA CTTCTTTAAG TACCGAGGAC GTAAAAGAAG
Conservation ********** ********** ********** ********** ********** **********

             ....|....| ....|....| ....|....| ....|....| ....|....| ....|....|
                  7985       7995       8005       8015       8025       8035
2d_SalI      AAACTGCTAA TCAGAAGGAG CTAGAAACTG ATAAGCAGAA GGAGCTAGAA AAAGCCATAG
2d_Thai      AAACTGCTAA TCAGAAGGAG CTAGAAACTG ATAAGCAGAA GGAGCTAGAA AAAGCCATAG
Conservation ********** ********** ********** ********** ********** **********

             ....|....| ....|....| ....|....| ....|....| ....|....| ....|....|
                  8045       8055       8065       8075       8085       8095
2d_SalI      AAAAGGAACA AGAAGAAGCT GTAGAAGGTG AACTACAAAA AACTATAGAA GTGGAAAAAG
2d_Thai      AAAAGGAACA AGAAGAAGCT GTAGAAGGTG AACTACAAAA AACTATAGAA GTGGAAAAAG
Conservation ********** ********** ********** ********** ********** **********

             ....|....| ....|....| ....|....| ....|....| ....|....| ....|....|
                  8105       8115       8125       8135       8145       8155
2d_SalI      AATCTGCTAT AAAAGAGGAA CAAGAAACTC CTGAGCAGGA GGAACGAGAA ACATCTATAA
2d_Thai      AATCTGCTAT AAAAGAGGAA CAAGAAACTC CTGAGCAGGA GGAACGAGAA ACATCTATAA
Conservation ********** ********** ********** ********** ********** **********

             ....|....| ....|....| ....|....| ....|....| ....|....| ....|....|
                  8165       8175       8185       8195       8205       8215
2d_SalI      ATCAGGAACC AGAACATGTT ACTTATGAGA CACTAGGATA TGATACACCA CATGCCCAAG
2d_Thai      ATCAGGAACC AGAACATGTT ACTTATGAGA CACTAGGATA TGATACACCA CATGCCCAAG
Conservation ********** ********** ********** ********** ********** **********

             ....|....| ....|....| ....|....| ....|....| ....|....| ....|....|
                  8225       8235       8245       8255       8265       8275
2d_SalI      AAAACGAGAG CGATGGTACT CAACACGACG ATTCAGAAAG AGATGATAAA TCAAAGGCAA
2d_Thai      AAAACGAGAG CGATGGTACT CAACACGACG ATTCAGAAAG AGATGATAAA TCAAAGGCAA
Conservation ********** ********** ********** ********** ********** **********

             ....|....| ....|....| ....|....| ....|....| ....|....| ....|....|
                  8285       8295       8305       8315       8325       8335
2d_SalI      AAGATGCAAT GGGAAGAACT CGTTTGGCAG GAGCTATTAT TATCGGTTTA TCTTTTTTTC
2d_Thai      AAGATGCAAT GGGAAGAACT CGTTTGGCAG GAGCTATTAT TATCGGTTTA TCTTTTTTTC
Conservation ********** ********** ********** ********** ********** **********

             ....|....| ....|....| ....|....| ....|....| ....|....| ....|....|
                  8345       8355       8365       8375       8385       8395
2d_SalI      CCGGAGTTCT TGTATTAGCA TTTAGGGACA CGCAAACAGA AGAAGAGGAA AGCCATGAAC
2d_Thai      CCGGAGTTCT TGTATTAGCA TTTAGGGACA CGCAAACAGA AGAAGAGGAA AGCCATGAAC
Conservation ********** ********** ********** ********** ********** **********
```

```
                ....|....| ....|....| ....|....| ....|....| ....|....| ....|....|
                    8405       8415       8425       8435       8445       8455
2d_SalI         ATGATCATCA TCAAGCATTT GGAGGTACTG ATGATTATAA TATGCAAGAT AAAGAAGAAG
2d_Thai         ATGATTATCA TCAAGCATTT GGAGGTACTG ATGATTATAA TATGCAAGAT AAAGAAGAAG
Conservation    ***** **** ********** ********** ********** ********** **********

                ....|....| ....|....| ....|....| ..
                    8465       8475       8485
2d_SalI         TTATCGAAGT CTGTTTTAAT GAGGATGATT AA
2d_Thai         TTATCGAAGT CTGTTTTAAT GAGGATGATT AA
Conservation    ********** ********** ********** **
```

Alignment: Pvrbp2dtr (align with genomic DNA of SalI)

```
               ....|....| ....|....| ....|....| ....|....| ....|....| ....|....|
                   5         15         25         35         45         55
2d_SalI_Ge     ATGGAAAAAA GAGCCTTTTG GATCATCTTT TACAATTTAA TATTTATTTT TTTCGgtaaa
2dtr_Thai      ATGGAAAAAA GAGCCTTTTG AATCATCTTT TACAATTTAA TATTTATTTT TTTCGGTAAA
Conservation   ********** ********** .********* ********** ********** **********

               ....|....| ....|....| ....|....| ....|....| ....|....| ....|....|
                  65         75         85         95        105        115
2d_SalI_Ge     attaaatcat gcgcacatac acgtaaattt atttgaatat gttcgctcaa attattgtaa
2dtr_Thai      ATTAAATCAT GCGCACATAC ACGTAAATTT ATTTGAATAT GTTCGCTCAA ATTATTGTAA
Conservation   ********** ********** ********** ********** ********** **********

               ....|....| ....|....| ....|....| ....|....| ....|....| ....|....|
                 125        135        145        155        165        175
2d_SalI_Ge     ccctaaaccc tgaaacctaa accctaaatt gaatgcaaat gttcacgttt gtacatttcc
2dtr_Thai      CCCTAAACCC TGAACCCTAA ACCAT----- ---------- ---------- ----------
Conservation   ********** ****.***** ***.*

               ....|....| ....|....| ....|....| ....|....| ....|....| ....|....|
                 185        195        205        215        225        235
2d_SalI_Ge     ataaattttc atttaactga atcgttacat atgctaaaat ctgttgtaac atgtattcat
2dtr_Thai      ---------- ---------- ---------- ---------- ---------- ----------
Conservation

               ....|....| ....|....| ....|....| ....|....| ....|....| ....|....|
                 245        255        265        275        285        295
2d_SalI_Ge     ttgaatttta atatcttata tttttcagCA TCATGTAAGG ATGTCAATCG AAACAAACCC
2dtr_Thai      ---------- ---------- ---------- ---------- ---------- ----------
Conservation

               ....|....| ....|....| ....|....| ....|....| ....|....| ....|....|
                 305        315        325        335        345        355
2d_SalI_Ge     GGAAGACTAA AATATCATCG GAAGCTATTA CCAGCATATG TTAACTTAAT GAGCCCTGAT
2dtr_Thai      ---------- ---------- ---------- ---------- ---------- ----------
Conservation

               ....|....| ....|....| ....|....| ....|....| ....|....| ....|....|
                 365        375        385        395        405        415
2d_SalI_Ge     AAAGACGGAT ATAAGGGTGC CGAAAAGGAT GATAAGGCAA ATAATCATAA CGACAACAAT
2dtr_Thai      ---------- ---------- ---------- ---------- ---------- ----------
Conservation

               ....|....| ....|....| ....|....| ....|....| ....|....| ....|....|
                 425        435        445        455        465        475
2d_SalI_Ge     AATGGCAACA ATAATGACAA CAATAATAAT AGTGACAATA ATGATGAAAA CAGTAATAAT
2dtr_Thai      ---------- ---------- ---------- ---------- ---------- ----------
Conservation

               ....|....| ....|....| ....|....| ....|....| ....|....| ....|....|
                 485        495        505        515        525        535
2d_SalI_Ge     AGTGAAAATC TCAGAACGTC AAATTTACAA AATAGTTCAT CAGTGGGGCA TTTAAACAAC
2dtr_Thai      ---------- ---------- ---------- ---------- ---------- ----------
Conservation

               ....|....| ....|....| ....|....| ....|....| ....|....| ....|....|
                 545        555        565        575        585        595
2d_SalI_Ge     CACGAAGATA CAACGAAACC ATCCCATTAC TCTTACTTGA AAAAAAGCAA CATATATGCA
2dtr_Thai      ---------- ---------- ---------- ---------- ---------- ----------
Conservation
```

```
             ....|....| ....|....| ....|....| ....|....| ....|....| ....|....|
                   605        615        625        635        645        655
2d_SalI_Ge   CCGGATGCAA AGAATAATAA AATGGAAGAT GATAATAAAA AATTGCATAC TGTTTCAAAT
2dtr_Thai    ---------- ---------- ---------- ---------- ---------- ----------
Conservation

             ....|....| ....|....| ....|....| ....|....| ....|....| ....|....|
                   665        675        685        695        705        715
2d_SalI_Ge   TCCTTCATTC AAAATAGTAA ACAAGCCCAA AATATAATTG CAACTAATAA TTTGGATTAT
2dtr_Thai    ---------- ---------- ---------- ---------- ---------- ----------
Conservation

             ....|....| ....|....| ....|....| ....|....| ....|....| ....|....|
                   725        735        745        755        765        775
2d_SalI_Ge   ATTAGTGCCA TTGATGATTA TAACAATATA ATTTCTTCAT TTCAACCATA TCATGCTAGT
2dtr_Thai    ---------- ---------- ---------- ---------- ---------A TCATGCTAGT
Conservation                                                     * **********

             ....|....| ....|....| ....|....| ....|....| ....|....| ....|....|
                   785        795        805        815        825        835
2d_SalI_Ge   GTATATTACT TGAATGAATT GAAATACTAT GCAACACATT ACAATGAATT GAAGGTTCTT
2dtr_Thai    GTATATTACT TGAATGAATT GAAATACTAT GCAACACATT ACAATGAATT GAAGGTTCTT
Conservation ********** ********** ********** ********** ********** **********

             ....|....| ....|....| ....|....| ....|....| ....|....| ....|....|
                   845        855        865        875        885        895
2d_SalI_Ge   GTTCAAAAAG ATGTCATTCC TCAAGTAACA CACATTACCA ATATTGTTGA GAAAAACGTT
2dtr_Thai    GTTCAAAAAG ATGTCATTCC TCAAGTAACA CACATTACCA ATATTGTTGA GAAAAACGTT
Conservation ********** ********** ********** ********** ********** **********

             ....|....| ....|....| ....|....| ....|....| ....|....| ....|....|
                   905        915        925        935        945        955
2d_SalI_Ge   AAGATCTGTT TGGAGAAAAC ACATGAATTG AATACATTAA TAACTCAATT AGAAAATCCA
2dtr_Thai    AAGATCTGTT TGGAGAAAAC ACATGAATTG AATACATTAA TAACTCAATT AGAAAATCCA
Conservation ********** ********** ********** ********** ********** **********

             ....|....| ....|....| ....|....| ....|....| ....|....| ....|....|
                   965        975        985        995       1005       1015
2d_SalI_Ge   CAAACATATA ATATTCAAAG AAGCCATTAT AGTAATAGAG TAAAAGATTA CCGTAAGAAA
2dtr_Thai    CAAACATATA ATATTCAAAG AAGCCATTAT AGTAATAGAG TAAAAGATTA CCGTAAGAAA
Conservation ********** ********** ********** ********** ********** **********

             ....|....| ....|....| ....|....| ....|....| ....|....| ....|....|
                  1025       1035       1045       1055       1065       1075
2d_SalI_Ge   ATTGAGGATA TTCAAAATTG TTTAAAATTA AATTATAAAA GAAATGTTAA AGCTACAGTA
2dtr_Thai    ATTGAGGATA TTCAAAATTG TTTAAAATTA AATTATAAAA GAAATGTTAA AGCTACAGTA
Conservation ********** ********** ********** ********** ********** **********

             ....|....| ....|....| ....|....| ....|....| ....|....| ....|....|
                  1085       1095       1105       1115       1125       1135
2d_SalI_Ge   TTTGCCACAA TAATATGGTT CCAGACACTA ATTAAAATGG AATGTAGTTG GTGGACCAAA
2dtr_Thai    TTTGCCACAA TAATATGGTT CCAGACACTA ATTAAAATGG AATGTAGTTG GTGGACCAAA
Conservation ********** ********** ********** ********** ********** **********

             ....|....| ....|....| ....|....| ....|....| ....|....| ....|....|
                  1145       1155       1165       1175       1185       1195
2d_SalI_Ge   TCATGTTCAA CGAGAATCTA TTATAATATG ATTAAGATGT ATACATTAAA AATTAAGGAG
2dtr_Thai    TCATGTTCAA CGAGAATCTA TTATAATATG ATTAAGATGT ATACATTAAA AATTAAGGAG
Conservation ********** ********** ********** ********** ********** **********
```

```
               ....|....| ....|....| ....|....| ....|....| ....|....| ....|....|
                    1205       1215       1225       1235       1245       1255
2d_SalI_Ge     TATAAAATCA AAAAACCAAC TGCATATATG GATAAAATAA AGGCTGTTTA TAAGTTAGCT
2dtr_Thai      TATAAAATCA AAAAACCAAC TGCATATATG GATAAAATAA AGGCTGTTTA TAAGTTAGCT
Conservation   ********** ********** ********** ********** ********** **********

               ....|....| ....|....| ....|....| ....|....| ....|....| ....|....|
                    1265       1275       1285       1295       1305       1315
2d_SalI_Ge     AATGATACAA TATGGAGAAT AAAAGTAGAA TTAAACTCAA ATCTTGATTC GGATACAACC
2dtr_Thai      AATGATACAA TATGGAGAAT AAAAGTAGAA TTAAACTCAA ATCTTGATTC GGATACAACC
Conservation   ********** ********** ********** ********** ********** **********

               ....|....| ....|....| ....|....| ....|....| ....|....| ....|....|
                    1325       1335       1345       1355       1365       1375
2d_SalI_Ge     GACTTTATTC TAGAAGAATT TAAATACATT ATAGAAAAGT ATAATGGTCA CATTGATAAA
2dtr_Thai      GACTTTATTC TAGAAGAATT TAAATACATT ATAGAAAAGT ATAATGGTCA CATTGATAAA
Conservation   ********** ********** ********** ********** ********** **********

               ....|....| ....|....| ....|....| ....|....| ....|....| ....|....|
                    1385       1395       1405       1415       1425       1435
2d_SalI_Ge     ATTAATTTGG GAACTTCTTA TATAGATCAT ATTCATGAAC AAGAAGGAAC TTTGAATAAT
2dtr_Thai      ATTAATTTGG GAACTTCTTA TATAGATCAT ATTCATGAAC AAGAAGGAAC TTTGAATAAT
Conservation   ********** ********** ********** ********** ********** **********

               ....|....| ....|....| ....|....| ....|....| ....|....| ....|....|
                    1445       1455       1465       1475       1485       1495
2d_SalI_Ge     ACTAAAATAG AAATTATAAC TTTGTATTCT GTGATAGCTA ATCGTTACAC TGCTTTTAAA
2dtr_Thai      ACTAAAATAG AAATTATAAC TTTGTATTCT GTGATAGCTA ATCGTTACAC TGCTTTTAAA
Conservation   ********** ********** ********** ********** ********** **********

               ....|....| ....|....| ....|....| ....|....| ....|....| ....|....|
                    1505       1515       1525       1535       1545       1555
2d_SalI_Ge     TTTAGTTTAG AACATATAAA TATGTTTGAA AATACATCCA AAAGTAAAGA ACAGGTACTT
2dtr_Thai      TTTAGTTTAG AACATATAAA TATGTTTGAA AATACATCCA AAAGTAAAGA ACAGGTACTT
Conservation   ********** ********** ********** ********** ********** **********

               ....|....| ....|....| ....|....| ....|....| ....|....| ....|....|
                    1565       1575       1585       1595       1605       1615
2d_SalI_Ge     TATAACTCTT TTAGCAAATT TGAGGATAGA TTACAAAAGA AAATAAACGA TCTTATTAAT
2dtr_Thai      TATAACTCTT TTAGCAAATT TGAGGATAGA TTACAAAAGA AAATAAACGA TCTTATTAAT
Conservation   ********** ********** ********** ********** ********** **********

               ....|....| ....|....| ....|....| ....|....| ....|....| ....|....|
                    1625       1635       1645       1655       1665       1675
2d_SalI_Ge     TCTGAATTTT CTATATCAAC CGTTAACTCT GTCATTTTGG ATTCCGAAAA AAATATACAA
2dtr_Thai      TCTGAATTTT CTATATCAAC CGTTAACTCT GTCATTTTGG ATTCCGAAAA AAATATACAA
Conservation   ********** ********** ********** ********** ********** **********

               ....|....| ....|....| ....|....| ....|....| ....|....| ....|....|
                    1685       1695       1705       1715       1725       1735
2d_SalI_Ge     TTATCAGAAT CATTACTCAA CTCGAGTTCT AAAGAAATAG CACAACATGA AATTAAATTA
2dtr_Thai      TTATCAGAAT CATTACTCAA CTCGAGTTCT AAAGAAATAG CACAACATGA AATTAAATTA
Conservation   ********** ********** ********** ********** ********** **********

               ....|....| ....|....| ....|....| ....|....| ....|....| ....|....|
                    1745       1755       1765       1775       1785       1795
2d_SalI_Ge     AATGCGGAAA TTGAAAAGAA AAAAAAAGAT TATGATCAAA AAATTATAAG AGCAAGAGAA
2dtr_Thai      AATGCGGAAA TTGAAAAGAA AAAAAAAGAT TATGATCAAA AAATTATAAG AGCAAGAGAA
Conservation   ********** ********** ********** ********** ********** **********
```

```
             ....|....| ....|....| ....|....| ....|....| ....|....| ....|....|
                  1805       1815       1825       1835       1845       1855
2d_SalI_Ge   ACTATAAAAA AATCAAAGGA ATTAATTACA TCTGTAAAAA ACGCATTTAA GTTAAGTTTA
2dtr_Thai    ACTATAAAAA AATCAAAGGA ATTAATTACA TCTGTAAAAA ACGCATTTAA GTTAAGTTTA
Conservation ********** ********** ********** ********** ********** **********

             ....|....| ....|....| ....|....| ....|....| ....|....| ....|....|
                  1865       1875       1885       1895       1905       1915
2d_SalI_Ge   GAAGAAAAGG AAAAGATAGA AAAGAAAGTA ACAGAAGCAA AAAAATTGCC AAACGCTCTA
2dtr_Thai    GAAGAAAAGG AAAAGATAGA AAAGAAAGTA ACAGAAGCAA AAAAATTGCC AAACGCTCTA
Conservation ********** ********** ********** ********** ********** **********

             ....|....| ....|....| ....|....| ....|....| ....|....| ....|....|
                  1925       1935       1945       1955       1965       1975
2d_SalI_Ge   GAAAGAGACA AAGCATATTT AGACATTATG AGTGAAATAA GAAAAATTAA AAATAAGTTA
2dtr_Thai    GAAAGAGACA AAGCATATTT AGACATTATG AGTGAAATAA GAAAAATTAA AAATAAGTTA
Conservation ********** ********** ********** ********** ********** **********

             ....|....| ....|....| ....|....| ....|....| ....|....| ....|....|
                  1985       1995       2005       2015       2025       2035
2d_SalI_Ge   TCTGATAATG CTAGAAAGAC AACCGAATTT ACTAAAAAAT CAAATGCTTT AAAAAAGGAA
2dtr_Thai    TCTGATAATG CTAGAAAGAC AACCGAATTT ACTAAAAAAT CAAATGCTTT AAAAAAGGAA
Conservation ********** ********** ********** ********** ********** **********

             ....|....| ....|....| ....|....| ....|....| ....|....| ....|....|
                  2045       2055       2065       2075       2085       2095
2d_SalI_Ge   GTTCAAGAAT TAAATACAAG TGTTAACAAC TATGTGCAAG CAATAAAGGA ACAAAAAGAA
2dtr_Thai    GTTCAAGAAT TAAATACAAG TGTTAACAAC TATGTGCAAG CAATAAAGGA ACAAAAAGAA
Conservation ********** ********** ********** ********** ********** **********

             ....|....| ....|....| ....|....| ....|....| ....|....| ....|....|
                  2105       2115       2125       2135       2145       2155
2d_SalI_Ge   AAAGAAATTC GGAATAATTC TTTGAAAGAT GAAATAAAAA AGTTTTTGGA ATATATACCT
2dtr_Thai    AAAGAAATTC GGAATAATTC TTTGAAAGAT GAAATAAAAA AGTTTTTGGA ATATATACCT
Conservation ********** ********** ********** ********** ********** **********

             ....|....| ....|....| ....|....| ....|....| ....|....| ....|....|
                  2165       2175       2185       2195       2205       2215
2d_SalI_Ge   AATAACAGAG AAAAAATAAA AGAATTAATT ACCAAAAAGA ATGAAGTGCA ACAATATATT
2dtr_Thai    AATAACAGAG AAAAAATAAA AGAATTAATT ACCAAAAAGA ATGAAGTGCA ACAATATATT
Conservation ********** ********** ********** ********** ********** **********

             ....|....| ....|....| ....|....| ....|....| ....|....| ....|....|
                  2225       2235       2245       2255       2265       2275
2d_SalI_Ge   CCCAAAATTG AAGAATTAAT TAAAGACGCA CACTTTGGTG TAGAAGAATT TACAGCCAAA
2dtr_Thai    CCCAAAATTG AAGAATTAAT TAAAGACGCA CACTTTGGTG TAGAAGAATT TACAGCCAAA
Conservation ********** ********** ********** ********** ********** **********

             ....|....| ....|....| ....|....| ....|....| ....|....| ....|....|
                  2285       2295       2305       2315       2325       2335
2d_SalI_Ge   AAAACAGAAT TGCAAAATCA GGTAAATTCA ATAATTGATG CATTTTATAA AGAAGACCTA
2dtr_Thai    AAAACAGAAT TGCAAAATCA GGTAAATTCA ATAATTGATG CATTTTATAA AGAAGACCTA
Conservation ********** ********** ********** ********** ********** **********

             ....|....| ....|....| ....|....| ....|....| ....|....| ....|....|
                  2345       2355       2365       2375       2385       2395
2d_SalI_Ge   CAATTATTTT TAGACAATTT ATCAAAATCT TATGATGAAA ATCAAGTTTT AGAGAAGGAA
2dtr_Thai    CAATTATTTT TAGACAATTT ATCAAAATCT TATGATGAAA ATCAAGTTTT AGAGAAGGAA
Conservation ********** ********** ********** ********** ********** **********
```

```
             ....|....| ....|....| ....|....| ....|....| ....|....| ....|....|
                  2405       2415       2425       2435       2445       2455
2d_SalI_Ge   GCTGATACTA CAGAAAAGAT TGAAGAACTT CACAAAAGAA CCAAAATGGA TTATGAAAAA
2dtr_Thai    GCTGATACTA CAGAAAAGAT TGAAGAACTT CACAAAAGAA CCAAAATGGA TTATGAAAAA
Conservation ********** ********** ********** ********** ********** **********

             ....|....| ....|....| ....|....| ....|....| ....|....| ....|....|
                  2465       2475       2485       2495       2505       2515
2d_SalI_Ge   CTTCTAAATA TGAAATGTGA TGATATACCC GAAATAATAA AAAAGTTGAA TACAGAATTA
2dtr_Thai    CTTCTAAATA TGAAATGTGA TGATATACCC GAAATAATAA AAAAGTTGAA TACAGAATTA
Conservation ********** ********** ********** ********** ********** **********

             ....|....| ....|....| ....|....| ....|....| ....|....| ....|....|
                  2525       2535       2545       2555       2565       2575
2d_SalI_Ge   AATAATCTCA AAAATTTAGA AAAAAATATT GTAGAAGAAC AGACTAAAAA CATTAATAAA
2dtr_Thai    AATAATCTCA AAAATTTAGA AAAAAATATT GTAGAAGAAC AGACTAAAAA CATTAATAAA
Conservation ********** ********** ********** ********** ********** **********

             ....|....| ....|....| ....|....| ....|....| ....|....| ....|....|
                  2585       2595       2605       2615       2625       2635
2d_SalI_Ge   TATGTTTCAG ATTCATTTGA TAACCTAACT GATGAAATGA ACCGTTTAAG AAATAGCTTG
2dtr_Thai    TATGTTTCAG ATTCATTTGA TAACCTAACT GATGAAATGA ACCGTTTAAG AAATAGCTTG
Conservation ********** ********** ********** ********** ********** **********

             ....|....| ....|....| ....|....| ....|....| ....|....| ....|....|
                  2645       2655       2665       2675       2685       2695
2d_SalI_Ge   AATGAATATA AGCAGGATGA AGGTAAGCTC CAAACATATA AAGGTAGTAT AAATGAAAGA
2dtr_Thai    AATGAATATA AGCAGGATGA AGGTAAGCTC CAAACATATA AAGGTAGTAT AAATGAAAGA
Conservation ********** ********** ********** ********** ********** **********

             ....|....| ....|....| ....|....| ....|....| ....|....| ....|....|
                  2705       2715       2725       2735       2745       2755
2d_SalI_Ge   AAAGACAAAT TCCTCAATAC TTCAAGCGAA AAAGAGGAAG ATATTCCAGA GGGACAAAAT
2dtr_Thai    AAAGACAAAT TCCTCAATAC TTCAAGCGAA AAAGAGGAAG ATATTCCAGA GGGACAAAAT
Conservation ********** ********** ********** ********** ********** **********

             ....|....| ....|....| ....|....| ....|....| ....|....| ....|....|
                  2765       2775       2785       2795       2805       2815
2d_SalI_Ge   ATTTATGATG AATATAAAAG TCATAAAGAT CTTATGGTTA ACAAAGAACT TAAATTATCC
2dtr_Thai    ATTTATGATG AATATAAAAG TCATAAAGAT CTTATGGTTA ACAAAGAACT TAAATTATCC
Conservation ********** ********** ********** ********** ********** **********

             ....|....| ....|....| ....|....| ....|....| ....|....| ....|....|
                  2825       2835       2845       2855       2865       2875
2d_SalI_Ge   AGTGATATTA ATGAATTCAA GGAAAATATA AAAAAAGTCC AAACAAAAAT AGAGGCCTTC
2dtr_Thai    AGTGATATTA ATGAATTCAA GGAAAATATA AAAAAAGTCC AAACAAAAAT AGAGGCCTTC
Conservation ********** ********** ********** ********** ********** **********

             ....|....| ....|....| ....|....| ....|....| ....|....| ....|....|
                  2885       2895       2905       2915       2925       2935
2d_SalI_Ge   AGCAGTGTAA TGCAAAAATT AGGAGCGAAC GCCGATGAAC AACATCAAGA AACTATAAAT
2dtr_Thai    AGCAGTGTAA TGCAAAAATT AGGAGCGAAC GCCGATGAAC AACATCAAGA AACTATAAAT
Conservation ********** ********** ********** ********** ********** **********

             ....|....| ....|....| ....|....| ....|....| ....|....| ....|....|
                  2945       2955       2965       2975       2985       2995
2d_SalI_Ge   TCTTTGAAGA ACTTTAATAC TAAAATCGAA GGTTTAAAAT TAAGTGAACT TGAAGGTGAA
2dtr_Thai    TCTTTGAAGA ACTTTAATAC TAAAATCGAA GGTTTAAAAT TAAGTGAACT TGAAGGTGAA
Conservation ********** ********** ********** ********** ********** **********
```

```
             ....|....| ....|....| ....|....| ....|....| ....|....| ....|....|
                  3005       3015       3025       3035       3045       3055
2d_SalI_Ge   TTTAAAATTA ACAATGAATC AGCTGCCAAA TTATATAATC AAATTGAAAA TACAATGAAA
2dtr_Thai    TTTAAAATTA ACAATGAATC AGCTGCCAAA TTATATAATC AAATTGAAAA TACAATGAAA
Conservation ********** ********** ********** ********** ********** **********

             ....|....| ....|....| ....|....| ....|....| ....|....| ....|....|
                  3065       3075       3085       3095       3105       3115
2d_SalI_Ge   AATATAGACA CAATTAAATC CTTAAATTAT ACTAGAAACA GCGCCAACAA TAGCAGAGAA
2dtr_Thai    AATATAGACA CAATTAAATC CTTAAATTAT ACTAGAAACA GCGCCAACAA TAGCAGAGAA
Conservation ********** ********** ********** ********** ********** **********

             ....|....| ....|....| ....|....| ....|....| ....|....| ....|....|
                  3125       3135       3145       3155       3165       3175
2d_SalI_Ge   TTAATGGTAA AAATAGTAAA AGATAAAAAT GATTTAATAA AAAAACTAGA TCAACAGTCT
2dtr_Thai    TTAATGGAAA AAATAGTAAA AGATAAAAAT GATTTAATAA AAAAACTAGA TCAACAGTCT
Conservation *******:** ********** ********** ********** ********** **********

             ....|....| ....|....| ....|....| ....|....| ....|....| ....|....|
                  3185       3195       3205       3215       3225       3235
2d_SalI_Ge   GAAGAAATAA AACAATACAC ATTAATAGAA GAAAAAGAAA AGTCACCCCT TCTAAGCGCT
2dtr_Thai    GAAGAAATAA AACAATACAC ATTAATAGAA GAAGAAGAAA AGTCACCCCT TCTAAGCGCT
Conservation ********** ********** ********** ***.****** ********** **********

             ....|....| ....|....| ....|....| ....|....| ....|....| ....|....|
                  3245       3255       3265       3275       3285       3295
2d_SalI_Ge   TTAAATGAAG AACAAAATCA TGTAGAAAGT GAAATTCCTA AAGAGTCGAT AGACAAATTA
2dtr_Thai    TTAAATGAAG AACAAAATCA TGTAGAAAGT GAAATTCCTA AAGAGTCGAT AGACAAATTA
Conservation ********** ********** ********** ********** ********** **********

             ....|....| ....|....| ....|....| ....|....| ....|....| ....|....|
                  3305       3315       3325       3335       3345       3355
2d_SalI_Ge   AACACAGAAA TAAATGCTAT ACTGGAATAT TACAACAGTT CAAAGGGTAA TTTTAATGAG
2dtr_Thai    AACACAGAAA TAAATGCTAT ACTGGAATAT TACAACAGTT CAAAGGGTAA TTTTAATGAG
Conservation ********** ********** ********** ********** ********** **********

             ....|....| ....|....| ....|....| ....|....| ....|....| ....|....|
                  3365       3375       3385       3395       3405       3415
2d_SalI_Ge   GACACGGAAA TAAAATTGGA AAAGTTAGAC AATTTTAAAA CTCAATGTGA TAATATTAAG
2dtr_Thai    GACACGGAAA TAAAATTGGA AAAGTTAGAC AATTTTAAAA CTCAATGTGA TAATATTAAG
Conservation ********** ********** ********** ********** ********** **********

             ....|....| ....|....| ....|....| ....|....| ....|....| ....|....|
                  3425       3435       3445       3455       3465       3475
2d_SalI_Ge   CAAGAAATAG AAACATTAAA CACTAAATAT AAAGTGTTAG AAAAAAGGAT AGACACCCTA
2dtr_Thai    CAATAAATAG AAACATTAAA CACTAAATAT AAAGTGTTAG AAAAAAGGAT AGACACCCTA
Conservation *** ****** ********** ********** ********** ********** **********

             ....|....| ....|....| ....|....| ....|....| ....|....| ....|....|
                  3485       3495       3505       3515       3525       3535
2d_SalI_Ge   ATTGATGAAC AACATGCTAA AATTGTGACG TTGACAGATA AGCACATAAC AACCAAAGAT
2dtr_Thai    ATTGATGAAC AACATGCTAA AATTGTGACG TTGACAGATA AGCACATAAC AACCAAAGAT
Conservation ********** ********** ********** ********** ********** **********

             ....|....| ....|....| ....|....| ....|....| ....|....| ....|....|
                  3545       3555       3565       3575       3585       3595
2d_SalI_Ge   AATATGATTA ATCAAAAGAT TGAACATAGC ACAAAATCGC TAGATGATAT GAAAACTAAG
2dtr_Thai    AATATGATTA ATCAAAAGAT TGAACATAGC ACAAAATCGC TAGATGATAT GAAAACTAAG
Conservation ********** ********** ********** ********** ********** **********
```

```
             ....|....| ....|....| ....|....| ....|....| ....|....| ....|....|
                 3605       3615       3625       3635       3645       3655
2d_SalI_Ge   TTGAATTCTC TCAAATTTAA CGATGATATC AGAAAGAATG AAAATGTCGA AATTCAAGGT
2dtr_Thai    TTGAATTCTC TCAAATTTAA CGATGATATC AGAAAGAATG AAAATGTCGA AATTCAAGGT
Conservation ********** ********** ********** ********** ********** **********

             ....|....| ....|....| ....|....| ....|....| ....|....| ....|....|
                 3665       3675       3685       3695       3705       3715
2d_SalI_Ge   AAAATACAGG AATTTGAAAA AATGGTTCAA ACTATAGAGG AAAGTATAAA TAAAAATAAA
2dtr_Thai    AAAATACAGG AATTTGAAAA AATGGTTCAA ACTATAGAGG AAAGTATAAA TAAAAATAAA
Conservation ********** ********** ********** ********** ********** **********

             ....|....| ....|....| ....|....| ....|....| ....|....| ....|....|
                 3725       3735       3745       3755       3765       3775
2d_SalI_Ge   ACTAAAATAT ATGAAATTAA AGGAGTATAC GATGTGTACA AGGGTGAATT CAATGAAGAA
2dtr_Thai    ACTAAAATAT ATGAAATTAA AGGAGTATAC GATGTGTACA AGGGTGAATT CAATGAAGAA
Conservation ********** ********** ********** ********** ********** **********

             ....|....| ....|....| ....|....| ....|....| ....|....| ....|....|
                 3785       3795       3805       3815       3825       3835
2d_SalI_Ge   AAAAATAAAA GCACAAAAAT CGTAGAAAAA AAAAATAGCA TGGAAAAAGT TTATAACAAA
2dtr_Thai    AAAAATAAAA GCACAAAAAT CGTAGAAAAA AAAAATAGCA TGGAAAAAGT TTATAACAAA
Conservation ********** ********** ********** ********** ********** **********

             ....|....| ....|....| ....|....| ....|....| ....|....| ....|....|
                 3845       3855       3865       3875       3885       3895
2d_SalI_Ge   ATGGAAGATA CTCTTAAAAA ATTAGAAGAT ATAGATAACG AAAAAAATAT ATTAAATGAA
2dtr_Thai    ATGGAAGATA CTCTTAAAAA ATTAGAAGAT ATAGATAACG AAAAAAATAT ATTAAATGAA
Conservation ********** ********** ********** ********** ********** **********

             ....|....| ....|....| ....|....| ....|....| ....|....| ....|....|
                 3905       3915       3925       3935       3945       3955
2d_SalI_Ge   ATAGAAAAGG CTGAAGTACT ATACAGGAAA GTTTTCATTC ATAATACTGT TCATATGATG
2dtr_Thai    ATAGAAAAGG CTGAAGTACT ATACAGGAAA GTTTTCATTC ATAATACTGT TCATATGATG
Conservation ********** ********** ********** ********** ********** **********

             ....|....| ....|....| ....|....| ....|....| ....|....| ....|....|
                 3965       3975       3985       3995       4005       4015
2d_SalI_Ge   GATAATGAAA CTAAAAAGTC TAAAAATTTG ATGGACCAAA TCGAATTGTC TAAAAGAGAA
2dtr_Thai    GATAATGAAA CTAAAAAGTC TAAAAATTTG ATGGACCAAA TCGAATTGTC TAAAAAAGAA
Conservation ********** ********** ********** ********** ********** *****.****

             ....|....| ....|....| ....|....| ....|....| ....|....| ....|....|
                 4025       4035       4045       4055       4065       4075
2d_SalI_Ge   ATTGAGGAAA CAAAGAAGCA AATGCTTGAA TACAAAAATG ATGAGGTATC TAATTATGAT
2dtr_Thai    ATTGAGGAAA CAAAGAAGCA AATGCTTGAA TACAAAAATG ATGAGGTATC TAATTATGAT
Conservation ********** ********** ********** ********** ********** **********

             ....|....| ....|....| ....|....| ....|....| ....|....| ....|....|
                 4085       4095       4105       4115       4125       4135
2d_SalI_Ge   TATATGAAAT ATTACAACCA GGCTACAGAG AGTAATGCTA AAATAAAAGA AACTTTTAAA
2dtr_Thai    TATATGAAAT ATTACAACCA GGCTACAGAG AGTAATGCTA AAATAAAAGA AACTTTTAAA
Conservation ********** ********** ********** ********** ********** **********

             ....|....| ....|....| ....|....| ....|....| ....|....| ....|....|
                 4145       4155       4165       4175       4185       4195
2d_SalI_Ge   AATGCTACAG ATAAGAAAGA AAAGGCTGAA AATATCGAAA AAGTAAGTGA AATCGATGAC
2dtr_Thai    AATGCTACAG ATCAGAAAGA AAAGGCTGAA AATATCGAAA AAGTAAGTGA AATCGATGAC
Conservation ********** **.******* ********** ********** ********** **********
```

```
             ....|....| ....|....| ....|....| ....|....| ....|....| ....|....|
                  4205       4215       4225       4235       4245       4255
2d_SalI_Ge   ATTAAAAACC AAGTTAATCA TAATTTACAT CAGATCCAAT CAGAAAATAG TTCTATAGAG
2dtr_Thai    ATTAAAAACC AAGTTAATCA TAATTTACAT CAGATCCAAT CAGAAAATAG TTCTATAGAG
Conservation ********** ********** ********** ********** ********** **********

             ....|....| ....|....| ....|....| ....|....| ....|....| ....|....|
                  4265       4275       4285       4295       4305       4315
2d_SalI_Ge   GAGATGTTAA AACAAATTAA TAATATGAAA GATTTATTAA AAACGAACAG TTCTAAAGGA
2dtr_Thai    GAGATGTTAA AACAAATTAA TAATATGAAA GATTTATTAA AAACGAACAG TTCTAAAGGA
Conservation ********** ********** ********** ********** ********** **********

             ....|....| ....|....| ....|....| ....|....| ....|....| ....|....|
                  4325       4335       4345       4355       4365       4375
2d_SalI_Ge   ATATCGGATA CAGTATTAAG TAGTACCAAA AATGCAGAAG AATTTGGCAA ACAAGCAATA
2dtr_Thai    ATATCGGATA CAGTATTAAG TAGTACCAAA AATGCAGAAG AATTTGGCAA ACAAGCAATA
Conservation ********** ********** ********** ********** ********** **********

             ....|....| ....|....| ....|....| ....|....| ....|....| ....|....|
                  4385       4395       4405       4415       4425       4435
2d_SalI_Ge   AGTGAATTTA ATAAAACGAA TGATGTAATA CAAGCAATCA GAGATTTGAT AACTAAGGCT
2dtr_Thai    AGTGAATTTA ATAAAACGAA TGATGTAATA CAAGCAATCA GAGATTTGAT AACTAAGGCT
Conservation ********** ********** ********** ********** ********** **********

             ....|....| ....|....| ....|....| ....|....| ....|....| ....|....|
                  4445       4455       4465       4475       4485       4495
2d_SalI_Ge   GAAGCACATA AAAATTTAAT CAATATAAAT TTAGAAGATG AAAAAATAAA TGCCGAAGTG
2dtr_Thai    GAAGCACATA AAAATTTAAT CAATATAAAT TTAGAAGATG AAAAAATAAA TGCCGAAGTG
Conservation ********** ********** ********** ********** ********** **********

             ....|....| ....|....| ....|....| ....|....| ....|....| ....|....|
                  4505       4515       4525       4535       4545       4555
2d_SalI_Ge   AAAGAAATTG AAAAAATTAA GGAACAAATT GCCAATAAGA AAGAAGAAAT AGAATCCCAT
2dtr_Thai    AAAGAAATTG AAAAAATTAA GGAACAAATT GCCAATAAGA AAGAAGAAAT AGAATCCCAT
Conservation ********** ********** ********** ********** ********** **********

             ....|....| ....|....| ....|....| ....|....| ....|....| ....|....|
                  4565       4575       4585       4595       4605       4615
2d_SalI_Ge   CTGAGCAAGG CAAAAGAATT TAAAGAAAAA TGTTCCTCAG AAACAAGTAA CGCCAAAAGA
2dtr_Thai    CTGAGCAAGG CAAAAGAATT TAAAGAAAAA TGTTCCTCAG AAACAAGTAA CGCCAAAAGA
Conservation ********** ********** ********** ********** ********** **********

             ....|....| ....|....| ....|....| ....|....| ....|....| ....|....|
                  4625       4635       4645       4655       4665       4675
2d_SalI_Ge   GGTAAAAGTA AAATAGAATT TTTGCAAAGT CGTGAAGGCA GTTCGGAAGA GGTAGATATG
2dtr_Thai    GGTAAAAGTA AAATAGAATT TTTGCAAAGT CGTGAAGGCA GTTCGGAAGA GGTAGATATG
Conservation ********** ********** ********** ********** ********** **********

             ....|....| ....|....| ....|....| ....|....| ....|....| ....|....|
                  4685       4695       4705       4715       4725       4735
2d_SalI_Ge   AAGGAGATAG AAGAAAATAT TACCAAAGCA GAAGGATATT TAAATCAAGC CATTGCTGCA
2dtr_Thai    AAGGAGATAG AAGAAAATAT TACCAAAGCA GAAGGATATT TAAATCAAGC CATTGCTGCA
Conservation ********** ********** ********** ********** ********** **********

             ....|....| ....|....| ....|....| ....|....| ....|....| ....|....|
                  4745       4755       4765       4775       4785       4795
2d_SalI_Ge   GAAACAGAAG CTAATAAGAA TGTAGAATTG TTCGGTGAAC TTCAAAAAAA TATCTGTGAC
2dtr_Thai    GAAACAGAAG CTAATAAGAA TGTAGAATTG TTCGGTGAAC TTCAAAAAAA TATCTGTGAC
Conservation ********** ********** ********** ********** ********** **********
```

```
             ....|....| ....|....| ....|....| ....|....| ....|....| ....|....|
                  4805       4815       4825       4835       4845       4855
2d_SalI_Ge   ATTTTCGATG AATCATCAAT TTTAGGAATA GAGACTACAT CAAAGAAAAA AATTAATAAA
2dtr_Thai    ATTTTCGATG AATCATCAAT TTTAGGAATA GAGACTACAT CAAAGAAAAA AATTAATAAA
Conservation ********** ********** ********** ********** ********** **********

             ....|....| ....|....| ....|....| ....|....| ....|....| ....|....|
                  4865       4875       4885       4895       4905       4915
2d_SalI_Ge   GCAACAGAAA TAATGGAAGA AATTAAAAGG AAAAATTTTG AAATTCAAGG CGAGGTGAAA
2dtr_Thai    GCAACAGAAA TAATGGAAGA AATTAAAAGG AAAAATTTTG AAATTCAAGG CGAGGTGAAA
Conservation ********** ********** ********** ********** ********** **********

             ....|....| ....|....| ....|....| ....|....| ....|....| ....|....|
                  4925       4935       4945       4955       4965       4975
2d_SalI_Ge   ATTTTCCATG AAACACTCGT TAAATTGAAA GAAACACACC CTGATAACAA CGTAGATGCT
2dtr_Thai    ATTTTCCATG AAACACTCGT TAAATTGAAA GAAACACACC CTGATAACAA CGTAGATGCT
Conservation ********** ********** ********** ********** ********** **********

             ....|....| ....|....| ....|....| ....|....| ....|....| ....|....|
                  4985       4995       5005       5015       5025       5035
2d_SalI_Ge   GAACTTAACA ATGCGAAATC GACCAATGCA AATGTACTTA TACAAACTAA CCTAGAAATG
2dtr_Thai    GAACTTAACA ATGCGAAATC GACCAATGCA AATGTACTTA TACAAACTAA CCTAGAAATG
Conservation ********** ********** ********** ********** ********** **********

             ....|....| ....|....| ....|....| ....|....| ....|....| ....|....|
                  5045       5055       5065       5075       5085       5095
2d_SalI_Ge   GTGGAACATA ACTTATCCGT AATAGCTAAA ATTAAACAGG AGGGAGAAAA TATATATAAT
2dtr_Thai    GTGGAACATA ACTTATCCGT AATAGCTAAA ATTAAACAGG AGGGAGAAAA TATATATAAT
Conservation ********** ********** ********** ********** ********** **********

             ....|....| ....|....| ....|....| ....|....| ....|....| ....|....|
                  5105       5115       5125       5135       5145       5155
2d_SalI_Ge   AGAGCCTCAT CTACGATAAA TTCCATGACA GAAATCTCCA AAAATACTGA AAAAAAAACA
2dtr_Thai    AGAGCCTCAT CTACGATAAA TTCCATGACA GAAATCTCCA AAAATACTGA AAAAAAAACA
Conservation ********** ********** ********** ********** ********** **********

             ....|....| ....|....| ....|....| ....|....| ....|....| ....|....|
                  5165       5175       5185       5195       5205       5215
2d_SalI_Ge   TTAGACAAGG CAAAAAGTGA CGAATCTAAA TATATATCCT ATTTAACCAA AATAACTTCA
2dtr_Thai    TTAGACAAGG CAAAAAGTGA CGAATCTAAA TATATATCCT ATTTAACCAA AATAACTTCA
Conservation ********** ********** ********** ********** ********** **********

             ....|....| ....|....| ....|....| ....|....| ....|....| ....|....|
                  5225       5235       5245       5255       5265       5275
2d_SalI_Ge   GAAAGAGATC TAATCATTGA AGAAAGGAAT AAACTAAATG GTATAAGCCC CAATATAATA
2dtr_Thai    GAAAGAAATC TAATCATTGA AGAAAGGAAT AAACTAAATG GTATAAGCCC CAATATAATA
Conservation ******.*** ********** ********** ********** ********** **********

             ....|....| ....|....| ....|....| ....|....| ....|....| ....|....|
                  5285       5295       5305       5315       5325       5335
2d_SalI_Ge   AGTATAGAAA AGGAATTGAA TAAAGCCAGA AAGGATTACG AAATTGGACT TTTGCAAAAG
2dtr_Thai    AGTATAGAAA AGGAATTGAA TAAAGCCAGA AAGGATTACG AAATTGGACT TTTGCAAAAG
Conservation ********** ********** ********** ********** ********** **********

             ....|....| ....|....| ....|....| ....|....| ....|....| ....|....|
                  5345       5355       5365       5375       5385       5395
2d_SalI_Ge   ATAGATGAAA TAGGCAAAAA CAGAAAAAAA GAATATTGAT TTGACAAAAG AATCAATAAA
2dtr_Thai    ATAGATGAAA TAGGCAAAAA CAGAAAAAAA -AATATTGAT TTGACAAAAG AATCAATAAA
Conservation ********** ********** **********  ********* ********** **********
```

```
             ....|....| ....|....| ....|....| ....|....| ....|....| ....|....|
                  5405       5415       5425       5435       5445       5455
2d_SalI_Ge   ATTAACATTG AGCAACTTTT CTTCCCATTT CAAAGGATTT GATTTAAAGG AATATGTCTT
2dtr_Thai    ATTAACATTG AGCAACTTTT CTTCCCATTT CAAAGGATTT GATTTAAAGG AATATGTCTT
Conservation ********** ********** ********** ********** ********** **********

             ....|....| ....|....| ....|....| ....|....| ....|....| ....|....|
                  5465       5475       5485       5495       5505       5515
2d_SalI_Ge   TAATAAAAAT ATAAATGACT ATGAACAAAA GATGAAAGAA ATACATGACA AATATGACAA
2dtr_Thai    TAATAAAAAT ATAAATGACT ATGAACAAAA GATGAAAGAA ATACATGACA AATATGACAA
Conservation ********** ********** ********** ********** ********** **********

             ....|....| ....|....| ....|....| ....|....| ....|....| ....|....|
                  5525       5535       5545       5555       5565       5575
2d_SalI_Ge   ATCGATAAAT AAAATTAGTA AAAACTTGAA AAAGGCTGAA GAGGATAATA CAAACTATAC
2dtr_Thai    ATCGATAAAT AAAATTAGTA AAAACTTGAA AAAGGCTGAA GAGGATAATA CAAACTATAC
Conservation ********** ********** ********** ********** ********** **********

             ....|....| ....|....| ....|....| ....|....| ....|....| ....|....|
                  5585       5595       5605       5615       5625       5635
2d_SalI_Ge   CCTAGCGAAT GAGCTGAGGA AAGAAGCGCA ACAAGAAAAA GCAGAACTTA CAAATATACA
2dtr_Thai    CCTAGCGAAT GAGCTGAGGA AAGAAGCGCA ACAAGAAAAA GCAGAACTTA CAAATATACA
Conservation ********** ********** ********** ********** ********** **********

             ....|....| ....|....| ....|....| ....|....| ....|....| ....|....|
                  5645       5655       5665       5675       5685       5695
2d_SalI_Ge   AGAAGAAGTA AATAAATATT TAAGGGATAT AAAAAAATGG GAATCATTCA GATTTATACT
2dtr_Thai    AGAAGAAGTA AATAAATATT TAAGGGATAT AAAAAAATGG GAATCATTCA GATTTATACT
Conservation ********** ********** ********** ********** ********** **********

             ....|....| ....|....| ....|....| ....|....| ....|....| ....|....|
                  5705       5715       5725       5735       5745       5755
2d_SalI_Ge   TGATATGAAA GACAATTTAA GTAATATAAA TACACTGATT AAACAAGAAG AATTAAAGGT
2dtr_Thai    TGATATGAAA GACAATTTAA GTAATATAAA TACACTGATT AAACAAGAAG AATTAAAGGT
Conservation ********** ********** ********** ********** ********** **********

             ....|....| ....|....| ....|....| ....|....| ....|....| ....|....|
                  5765       5775       5785       5795       5805       5815
2d_SalI_Ge   TAATGATGGA TATGAATACA TTAAACAATT AGTTGACAAT ATTAAAGAGT CAAATGATGA
2dtr_Thai    TAATGATGGA TATGAATACA TTAAACAATT AGTTGACAAT ATTAAAGAGT CAAATGATGA
Conservation ********** ********** ********** ********** ********** **********

             ....|....| ....|....| ....|....| ....|....| ....|....| ....|....|
                  5825       5835       5845       5855       5865       5875
2d_SalI_Ge   AAGCAGTATA TCAGATAACT TACAAAAAGG AAAAGAAAAA AATGTAGAAA TACAGAACAA
2dtr_Thai    AAGCAGTATA TCAGATAACT TACAAAAAGG AAAAGAAAAA AATGTAGAAA TACAGAACAA
Conservation ********** ********** ********** ********** ********** **********

             ....|....| ....|....| ....|....| ....|....| ....|....| ....|....|
                  5885       5895       5905       5915       5925       5935
2d_SalI_Ge   AATGCAATCT ACGTATAAAA ACAAGGCAAA AGTTATTTTA GGACATATAA TTGACTCCGC
2dtr_Thai    AATGCAATCT ACGTATAAAA ACAAGGCAAA AGTTATTTTA GGACATATAA TTGACTCCGC
Conservation ********** ********** ********** ********** ********** **********

             ....|....| ....|....| ....|....| ....|....| ....|....| ....|....|
                  5945       5955       5965       5975       5985       5995
2d_SalI_Ge   AAAATTTATA GATATTAATA TAATTACAAA ATCACCACTA AACGAATTGT CTTCAGAATC
2dtr_Thai    AAAATTTATA GATATTAATA TAATTACAAA ATCACCACTA AACGAATTGT CTTCAGAATC
Conservation ********** ********** ********** ********** ********** **********
```

```
             ....|....| ....|....| ....|....| ....|....| ....|....| ....|....|
                 6005       6015       6025       6035       6045       6055
2d_SalI_Ge   ACATTTGATG AATGCAGGAG AATTAAAATT TCAGCAAGAA AATAAAGTAA CATTAGAAAC
2dtr_Thai    ACATTTGATG AATGCAGGAG AATTAAAATT TCAGCAAGAA AATAAAGTAA CATTAGAAAC
Conservation ********** ********** ********** ********** ********** **********

             ....|....| ....|....| ....|....| ....|....| ....|....| ....|....|
                 6065       6075       6085       6095       6105       6115
2d_SalI_Ge   AGAACATATG ACAAACAATA AGAGCGAATT AGATGTTTAT AAAAATATAC GCGATGCTTA
2dtr_Thai    AGAACATATG ACAAACAATA AGAGCGAATT AGATGTTTAT AAAAATATAC GCGATGCTTA
Conservation ********** ********** ********** ********** ********** **********

             ....|....| ....|....| ....|....| ....|....| ....|....| ....|....|
                 6125       6135       6145       6155       6165       6175
2d_SalI_Ge   CAACATCGTT GTGGAGATAA TTGCATATTC AGATAAAATA GACACAAAAC AAAGACAAAG
2dtr_Thai    CAACATCGTT GTGGAGATAA TTGCATATTC AGATAAAATA GACACAAAAC AAAGACAAAG
Conservation ********** ********** ********** ********** ********** **********

             ....|....| ....|....| ....|....| ....|....| ....|....| ....|....|
                 6185       6195       6205       6215       6225       6235
2d_SalI_Ge   TACACAATTA TTGGATGATG GAAATGATAT ATATCTTACG CTAAAGTCAA TAAACGAGCT
2dtr_Thai    TACACAATTA TTGGATGATG GAAATGATAT ATATCTTACG CTAAAGTCAA TAAACGAGCT
Conservation ********** ********** ********** ********** ********** **********

             ....|....| ....|....| ....|....| ....|....| ....|....| ....|....|
                 6245       6255       6265       6275       6285       6295
2d_SalI_Ge   AAAAAATAAA ATAAACTCTG TAAAAAGTAA AGAAAATGCT ATTTCAGGCA AAATAGATAA
2dtr_Thai    AAAAAATAAA ATAAACTCTG TAAAAAGTAA AGAAAATGCT ATTTCAGGCA AAATAGATAA
Conservation ********** ********** ********** ********** ********** **********

             ....|....| ....|....| ....|....| ....|....| ....|....| ....|....|
                 6305       6315       6325       6335       6345       6355
2d_SalI_Ge   TATTTCAAAC AAACTTAATG AGTTAAACAA AATTACATGT GATGATAAAA GTTACGATAA
2dtr_Thai    TATTTCAAAC AAACTTAATG AGTTAAACAA AATTACATGT GATGATAAAA GTTACGATAA
Conservation ********** ********** ********** ********** ********** **********

             ....|....| ....|....| ....|....| ....|....| ....|....| ....|....|
                 6365       6375       6385       6395       6405       6415
2d_SalI_Ge   TATTTTAGAC ATAATAAAAC ATACAGAATT AAAAACTATA CGTGATTCTT TTAAACAAGA
2dtr_Thai    TATTTTAGAC ATAATAAAAC ATACAGAATT AAAAACTATA CGTGATTCTT TTAAACAAGA
Conservation ********** ********** ********** ********** ********** **********

             ....|....| ....|....| ....|....| ....|....| ....|....| ....|....|
                 6425       6435       6445       6455       6465       6475
2d_SalI_Ge   AAAAATTAAC AAAAAGAGCG ATTCTAAATT GGAAAAAATA AAGGAAGAAT TTGAAAATTC
2dtr_Thai    AAAAATTAAC AAAAAGAGCG ATTCTAAATT GGAAAAAATA AAGGAAGAAT TTGAAAATTC
Conservation ********** ********** ********** ********** ********** **********

             ....|....| ....|....| ....|....| ....|....| ....|....| ....|....|
                 6485       6495       6505       6515       6525       6535
2d_SalI_Ge   AAAAAATACA TTAAAAAAATA TAGAAGAAGA AGTTAATGCT CTAAAGGCTA GTTTGGTAAA
2dtr_Thai    AAAAAATACA TTAAAAAAATA TAGAAGAAGA AGTTAATGCT CTAAAGGCTA GTTTGGTAAA
Conservation ********** ********** ********** ********** ********** **********

             ....|....| ....|....| ....|....| ....|....| ....|....| ....|....|
                 6545       6555       6565       6575       6585       6595
2d_SalI_Ge   ACATGATAAT ATACAAAGCA GAAACAAGCC AATTGATGAT GTGCTAATCG AAATAGAAAA
2dtr_Thai    ACATGATAAT ATACAAAGCA GAAACAAGCC AATTGATGAT GTGCTAATCG AAATAGAAAA
Conservation ********** ********** ********** ********** ********** **********
```

```
             ....|....| ....|....| ....|....| ....|....| ....|....| ....|....|
                  6605       6615       6625       6635       6645       6655
2d_SalI_Ge   AACAGAAAAA GGTATAGATA GTTTTAGTGC CTCCCTTGAT GAGATGTTGA AGAAAGGAAG
2dtr_Thai    AACAGAAAAA GGTATAGATA GTTTTAGTGC CTCCCTTGAT GAGATGTTGA AGAAAGGAAG
Conservation ********** ********** ********** ********** ********** **********

             ....|....| ....|....| ....|....| ....|....| ....|....| ....|....|
                  6665       6675       6685       6695       6705       6715
2d_SalI_Ge   AGAATGCGAA ATATTTAGGT ATACATCCAT AAAAGATGGT GTTATCGCAA AAATAAATGA
2dtr_Thai    AGAATGCGAA ATATTTAGGT ATACATCCAT AAAAGATGGT GTTATCGCAA AAATAAATGA
Conservation ********** ********** ********** ********** ********** **********

             ....|....| ....|....| ....|....| ....|....| ....|....| ....|....|
                  6725       6735       6745       6755       6765       6775
2d_SalI_Ge   TGACGCAGAA TTAATCGATA ATATACACAA TAATACTAAT GAATATTTGA CATATGTTCA
2dtr_Thai    TGACGCAGAA TTAATCGATA ATATACACAA TAATACTAAT GAATATTTGA CATATGTTCA
Conservation ********** ********** ********** ********** ********** **********

             ....|....| ....|....| ....|....| ....|....| ....|....| ....|....|
                  6785       6795       6805       6815       6825       6835
2d_SalI_Ge   AAAAAATTAT AGTGAAACAG CAGAGGATGT TCGTACATTA AATCAACATT TCATGACAGA
2dtr_Thai    AAAAAATTAT AGTGAAACAG CAGAGGATGT TCGCACATTA AATCAACATT TCATGACAGA
Conservation ********** ********** ********** *** ****** ********** **********

             ....|....| ....|....| ....|....| ....|....| ....|....| ....|....|
                  6845       6855       6865       6875       6885       6895
2d_SalI_Ge   AACGATAAGC GATCATGCGC CAACTAATTA CGAAACATCA AATAAATCTT ATGAAGAGAT
2dtr_Thai    AACGATAAGC GATCATGCGC CAACTAATTA CGAAACATCA AATAAATCTT ATGAAGAGAT
Conservation ********** ********** ********** ********** ********** **********

             ....|....| ....|....| ....|....| ....|....| ....|....| ....|....|
                  6905       6915       6925       6935       6945       6955
2d_SalI_Ge   AACTGAAGCT GTTAATAATT CCAAAGCAAT AATAGATAAT ATACAAAATT CAATTATACA
2dtr_Thai    AACTGAAGCT GTTAATAATT CCAAAGCAAT AATAGATAAT ATACAAAATT CAATTATACA
Conservation ********** ********** ********** ********** ********** **********

             ....|....| ....|....| ....|....| ....|....| ....|....| ....|....|
                  6965       6975       6985       6995       7005       7015
2d_SalI_Ge   AGTTAACGAA AAAACGGAAA TTAGTTCTTT AGAAAATAGC GCAGAAAAAA TAGAAAAGCT
2dtr_Thai    AGTTAACGAA AAAACGGAAA TTAGTTCTTT AGAAAATAGC GCAGAAAAAA TAGAAAAGCT
Conservation ********** ********** ********** ********** ********** **********

             ....|....| ....|....| ....|....| ....|....| ....|....| ....|....|
                  7025       7035       7045       7055       7065       7075
2d_SalI_Ge   TTATAAAGAA TTACAGGATA AAAAAAACAC CATAAACGAA ATTTATAAAA AGGCTAACAT
2dtr_Thai    TTATAAAGAA TTACAGGATA AAAAAAACAC CATAAACGAA ATTTATAAAA AGGCTAACAT
Conservation ********** ********** ********** ********** ********** **********

             ....|....| ....|....| ....|....| ....|....| ....|....| ....|....|
                  7085       7095       7105       7115       7125       7135
2d_SalI_Ge   AGTTAAATTG CAAGAAATAA AATCAGATGC TGATAAATAT TTGGATGTAG CTAACATATT
2dtr_Thai    AGTTAAATTG CAAGAAATAA AATCAGATGC TGATAAATAT TTGGATGTAG CTAACATATT
Conservation ********** ********** ********** ********** ********** **********

             ....|....| ....|....| ....|....| ....|....| ....|....| ....|....|
                  7145       7155       7165       7175       7185       7195
2d_SalI_Ge   TAATAATGTA TTGGACGGTC AAAAATCAAG AATAATAAAT AACCTAGGTA AAATAGCCCA
2dtr_Thai    TAATAATGTA TTGGACGGTC AAAAATCAAG AATAATAAAT AACCTAGGTA AAATAGCCCA
Conservation ********** ********** ********** ********** ********** **********
```

```
             ....|....| ....|....| ....|....| ....|....| ....|....| ....|....|
                 7205       7215       7225       7235       7245       7255
2d_SalI_Ge   AGTTAAAGAA ACAATAAACC TAAAACTGAA GAAATTGGTT GAGACTGACA ATACATTTAC
2dtr_Thai    AGTTAAAGAA ACAATAAACC TAAAACTGAA GAAATTGGTT GAGACTGACA ATACATTTAC
Conservation ********** ********** ********** ********** ********** **********

             ....|....| ....|....| ....|....| ....|....| ....|....| ....|....|
                 7265       7275       7285       7295       7305       7315
2d_SalI_Ge   GACAGAGTCT ATTAACAGGT TTGGAGAAAT TTATAATGAC ATTAAGACTA GTATAGATGA
2dtr_Thai    GACAGAGTCT ATTAACAGGT TTGGAGAAAT TTATAATGAC ATTAAGACTA GTATAGATGA
Conservation ********** ********** ********** ********** ********** **********

             ....|....| ....|....| ....|....| ....|....| ....|....| ....|....|
                 7325       7335       7345       7355       7365       7375
2d_SalI_Ge   ACTGGAAATT ATAGAACAAA CAAATTATAG CGAACAAAAT GATGTAAAAA ATCATAAAGA
2dtr_Thai    ACTGGAAATT ATAGAACAAA CAAATTATAG CGAACAAAAT GATGTAAAAA ATCATAAAGA
Conservation ********** ********** ********** ********** ********** **********

             ....|....| ....|....| ....|....| ....|....| ....|....| ....|....|
                 7385       7395       7405       7415       7425       7435
2d_SalI_Ge   AAAAATTACA TACTTAATAA GCAGAAGAGA AACTTTAGAA AGTGACCTAA AAGGACATGA
2dtr_Thai    AAAAATTACA TACTTAATAA GCAGAAGAGA AACTTTAGAA AGTGACCTAA AAGGACATGA
Conservation ********** ********** ********** ********** ********** **********

             ....|....| ....|....| ....|....| ....|....| ....|....| ....|....|
                 7445       7455       7465       7475       7485       7495
2d_SalI_Ge   AGAGGACACA AATGTGAAAA AATTAAATGC GAATACATTA AGTGAAGTAA ATATTGGCAT
2dtr_Thai    AGAGGACACA AATGTGAAAA AATTAAATGC GAATACATTA AGTGAAGTAA ATATTGGCAT
Conservation ********** ********** ********** ********** ********** **********

             ....|....| ....|....| ....|....| ....|....| ....|....| ....|....|
                 7505       7515       7525       7535       7545       7555
2d_SalI_Ge   TGCAAGTGCA AAAGAAGCTA TAAGCAACTC AGAGGAAGTA TTTAAGAAAT TATTAAGAAA
2dtr_Thai    TGCAAGTGCA AAAGAAGCTA TAAGCAACTC AGAGGAAGTA TTTAAGAAAT TATTAAGAAA
Conservation ********** ********** ********** ********** ********** **********

             ....|....| ....|....| ....|....| ....|....| ....|....| ....|....|
                 7565       7575       7585       7595       7605       7615
2d_SalI_Ge   AATAGAAGAA AATGAAAGAT TATGTAATAA TAATGATGCA AAGAATTTTA TTTCGGATAT
2dtr_Thai    AATAGAAGAA AATGAAAGAT TATGTAATAA TAATGATGCA AAGAATTTTA TTTCGGATAT
Conservation ********** ********** ********** ********** ********** **********

             ....|....| ....|....| ....|....| ....|....| ....|....| ....|....|
                 7625       7635       7645       7655       7665       7675
2d_SalI_Ge   TATGCAAAAT ATAGATGATC TGAATAAACG TTTCACAAAA AATATACCAG AAAGAGAAAA
2dtr_Thai    TATGCAAAAT ATAGATGATC TGAATAAACG TTTCACAAAA AATATACCAG AAAGAGAAAA
Conservation ********** ********** ********** ********** ********** **********

             ....|....| ....|....| ....|....| ....|....| ....|....| ....|....|
                 7685       7695       7705       7715       7725       7735
2d_SalI_Ge   ACTTTTTGAA ATAGAAAAGG ATTATATATA ATGAAATTAA TTCCATTTTC AATGAAATAA
2dtr_Thai    ACTTTTTGAA ATAGAAAAGG ATTATATATA ATGAAATTAA TTCCATTTTC AATGAAATAA
Conservation ********** ********** ********** ********** ********** **********

             ....|....| ....|....| ....|....| ....|....| ....|....| ....|....|
                 7745       7755       7765       7775       7785       7795
2d_SalI_Ge   GAACACTTAA CGTTGACGAA TTTGTTGAAA AAATGTATAA ACAAATTGAT AGTGAAAAGG
2dtr_Thai    GAACACTTAA CGTTGACGAA TTTGTTGAAA AAATGTATAA ACAAATTGAT AGTGAAAAGG
Conservation ********** ********** ********** ********** ********** **********
```

```
             ....|....| ....|....| ....|....| ....|....| ....|....| ....|....|
                  7805       7815       7825       7835       7845       7855
2d_SalI_Ge   AAAGCGTAAA TAACATAACA GATACTGATA AGATAAGTGT TGCAATTCAA AATGTTACAA
2dtr_Thai    AAAGCGTAAA TAACATAACA GATACTGATA AGATAAGTGT TGCAATTCAA AATGTTACAA
Conservation ********** ********** ********** ********** ********** **********

             ....|....| ....|....| ....|....| ....|....| ....|....| ....|....|
                  7865       7875       7885       7895       7905       7915
2d_SalI_Ge   ATTACGATGC AGAAATAAAA GGCTTATTGT CTAGAACTAT TAACGTATTA GAAAGAATAA
2dtr_Thai    ATTACGATGC AGAAATAAAA GGCTTATTGT CTAGAACTAT TAACGTATTA GAAAGAATAA
Conservation ********** ********** ********** ********** ********** **********

             ....|....| ....|....| ....|....| ....|....| ....|....| ....|....|
                  7925       7935       7945       7955       7965       7975
2d_SalI_Ge   GTAGAAGGAA AGCGGAAATG GATACTTTAT TCAATAGTCT ATCATCGAAT AATAAAAACC
2dtr_Thai    GTAGAAGGAA AGCGGAAATG GATACTTTAT TCAATAGTCT ATCATCGAAT AATAAAAACC
Conservation ********** ********** ********** ********** ********** **********

             ....|....| ....|....| ....|....| ....|....| ....|....| ....|....|
                  7985       7995       8005       8015       8025       8035
2d_SalI_Ge   TAAATGAAAA TGCAAAAAGA TACATGAATG ATTCAGCTGA AATAATTAAT CAATTAAATA
2dtr_Thai    TAAATGAAAA TGCAAAAAGA TACATGAATG ATTCAGCTGA AATAATTAAT CAATTAAATA
Conservation ********** ********** ********** ********** ********** **********

             ....|....| ....|....| ....|....| ....|....| ....|....| ....|....|
                  8045       8055       8065       8075       8085       8095
2d_SalI_Ge   GCCATGTTCA AAAAATAACA AAACTGAAAA CATATGCAAA TGAGGTAATT GAACAATTAC
2dtr_Thai    GCCATGTTCA AAAAATAACA AAACTGAAAA CATATGCAAA TGAGGTAATT GAACAATTAC
Conservation ********** ********** ********** ********** ********** **********

             ....|....| ....|....| ....|....| ....|....| ....|....| ....|....|
                  8105       8115       8125       8135       8145       8155
2d_SalI_Ge   AAAAAGAACT AACAAGATTA TTAGATCAAC GTAAAATTGA AATTCCAGTT GATGATACTC
2dtr_Thai    AAAAAGAACT AACAAGATTA TTAGATCAAC GTAAAATTGA AATTCCAGTT GATGATACTC
Conservation ********** ********** ********** ********** ********** **********

             ....|....| ....|....| ....|....| ....|....| ....|....| ....|....|
                  8165       8175       8185       8195       8205       8215
2d_SalI_Ge   AAACTTCTTT AAGTACCGAG GACGTAAAAG AAGAAACTGC TAATCAGAAG GAGCTAGAAA
2dtr_Thai    AAACTTCTTT AAGTACCGAG GACGTAAAAG AAGAAACTGC TAATCAGAAG GAGCTAGAAA
Conservation ********** ********** ********** ********** ********** **********

             ....|....| ....|....| ....|....| ....|....| ....|....| ....|....|
                  8225       8235       8245       8255       8265       8275
2d_SalI_Ge   CTGATAAGCA GAAGGAGCTA GAAAAAGCCA TAGAAAAGGA ACAAGAAGAA GCTGTAGAAG
2dtr_Thai    CTGATAAGCA GAAGGAGCTA GAAAAAGCCA TAGAAAAGGA ACAAGAAGAA GCTGTAGAAG
Conservation ********** ********** ********** ********** ********** **********

             ....|....| ....|....| ....|....| ....|....| ....|....| ....|....|
                  8285       8295       8305       8315       8325       8335
2d_SalI_Ge   GTGAACTACA AAAAACTATA GAAGTGGAAA AAGAATCTGC TATAAAAGAG GAACAAGAAA
2dtr_Thai    GTGAACTACA AAAAACTATA GAAGTGGAAA AAGAATCTGC TATAAAAGAG GAACAAGAAA
Conservation ********** ********** ********** ********** ********** **********

             ....|....| ....|....| ....|....| ....|....| ....|....| ....|....|
                  8345       8355       8365       8375       8385       8395
2d_SalI_Ge   CTCCTGAGCA GGAGGAACGA GAAACATCTA TAAATCAGGA ACCAGAACAT GTTACTTATG
2dtr_Thai    CTCCTGAGCA GGAGGAACGA GAAACATCTA TAAATCAGGA ACCAGAACAT GTTACTTATG
Conservation ********** ********** ********** ********** ********** **********
```

```
             ....|....| ....|....| ....|....| ....|....| ....|....| ....|....|
                8405       8415       8425       8435       8445       8455
2d_SalI_Ge   AGACACTAGG ATATGATACA CCACATGCCC AAGAAAACGA GAGCGATGGT ACTCAACACG
2dtr_Thai    AGACACTAGG ATATGATACA CCACATGCCC AAGAAAACGA GAGCGATGGT ACTCAACACG
Conservation ********** ********** ********** ********** ********** **********

             ....|....| ....|....| ....|....| ....|....| ....|....| ....|....|
                8465       8475       8485       8495       8505       8515
2d_SalI_Ge   ACGATTCAGA AAGAGATGAT AAATCAAAGG CAAAAGATGC AATGGGAAGA ACTCGTTTGG
2dtr_Thai    ACGATTCAGA AAGAGATGAT AAATCAAAGG CAAAAGATGC AATGGGAAGA ACTCGTTTGG
Conservation ********** ********** ********** ********** ********** **********

             ....|....| ....|....| ....|....| ....|....| ....|....| ....|....|
                8525       8535       8545       8555       8565       8575
2d_SalI_Ge   CAGGAGCTAT TATTATCGGT TTATCTTTTT TTCCCGGAGT TCTTGTATTA GCATTTAGGG
2dtr_Thai    CAGGAGCTAT TATTATCGGT TTATCTTTTT TTCCCGGAGT TCTTGTATTA GCATTTAGGG
Conservation ********** ********** ********** ********** ********** **********

             ....|....| ....|....| ....|....| ....|....| ....|....| ....|....|
                8585       8595       8605       8615       8625       8635
2d_SalI_Ge   ACACGCAAAC AGAAGAAGAG GAAAGCCATG AACATGATCA TCATCAAGCA TTTGGAGGTA
2dtr_Thai    ACACGCAAAC AGAAGAAGAG GAAAGCCATG AACATGATTA TCATCAAGCA TTTGGAGGTA
Conservation ********** ********** ********** ******** * ********** **********

             ....|....| ....|....| ....|....| ....|....| ....|....| ....|....|
                8645       8655       8665       8675       8685       8695
2d_SalI_Ge   CTGATGATTA TAATATGCAA GATAAAGAAG AAGTTATCGA AGTCTGTTTT AATGAGGATG
2dtr_Thai    CTGATGATTA TAATATGCAA GATAAAGAAG AAGTTATCGA AGTCTGTTTT AATGAGGATG
Conservation ********** ********** ********** ********** ********** **********

             ....|
                8705
2d_SalI_Ge   ATTAA
2dtr_Thai    ATTAA
Conservation *****
```

Alignment: Pvrbp2e

```
               ....|....| ....|....| ....|....| ....|....| ....|....| ....|....|
                   5         15         25         35         45         55
2e_Brazil      ---------- ---------- ---------- ---------- ---------- ----------
2e_Thai        ATGGAAAAGA ACCTATTTTT TGTGATCTTT TGTAACCTCT TGTTTATTCT TTCCGCAGTA
Conservation

               ....|....| ....|....| ....|....| ....|....| ....|....| ....|....|
                  65         75         85         95        105        115
2e_Brazil      ---------- ---------- ---------- ---------- ---------- ----------
2e_Thai        TGTAAAGACA ATAACAAAAA CCAACCTAAC AGGCTCAAAA GGGATTTCAA GCTATCACCA
Conservation

               ....|....| ....|....| ....|....| ....|....| ....|....| ....|....|
                 125        135        145        155        165        175
2e_Brazil      ---------- ---------- ---------- ---------- ---------- ----------
2e_Thai        ATACATGCCA CTTCCTTGGG AAGTGACCAG TTCGAACATG GCAGTGAAGC AAAGGGGAAC
Conservation

               ....|....| ....|....| ....|....| ....|....| ....|....| ....|....|
                 185        195        205        215        225        235
2e_Brazil      ---------- ---------- ---------- ---------- ---------- ----------
2e_Thai        GAACAAAATA ATCGTAACAG TGACAATGAT TACAGTAATG AGCACCCTAA TGATGATAAC
Conservation

               ....|....| ....|....| ....|....| ....|....| ....|....| ....|....|
                 245        255        265        275        285        295
2e_Brazil      ---------- ---------- ---------- ---------- ---------- ----------
2e_Thai        AAAATAAGAG GAGAAAACGT AAACACGACT CACTTACGAA ATAATTCATC CTTTATATCC
Conservation

               ....|....| ....|....| ....|....| ....|....| ....|....| ....|....|
                 305        315        325        335        345        355
2e_Brazil      ---------- ---------- ---------- ---------- ---------- ----------
2e_Thai        TTAAAAAATT ATAAAATTGC AAGAAAACCA TCTCGCTATT CATACCTAGA TAAGGACAAC
Conservation

               ....|....| ....|....| ....|....| ....|....| ....|....| ....|....|
                 365        375        385        395        405        415
2e_Brazil      ---------- ---------- ---------- ---------- ---------- ----------
2e_Thai        ACATATGCAG TTACTACCAA AAATAATGAT AAAAAAAACT TACAGAAAGA GATTAATACT
Conservation

               ....|....| ....|....| ....|....| ....|....| ....|....| ....|....|
                 425        435        445        455        465        475
2e_Brazil      ---------- ---------- ---------- ---------- ---------- ----------
2e_Thai        ATTCCAAGTA CATTCATACA AAAAACTCAT TATGAACCCC AGAGAGTGAG TGATTCATGC
Conservation

               ....|....| ....|....| ....|....| ....|....| ....|....| ....|....|
                 485        495        505        515        525        535
2e_Brazil      ---------- ---------- ---------- ---------- ---------- ----------
2e_Thai        GATTAATCTG GATTATGTTT GTATCTACGG TGACCATAAA AGTATAATTT CTTTATTGTA
Conservation

               ....|....| ....|....| ....|....| ....|....| ....|....| ....|....|
                 545        555        565        575        585        595
2e_Brazil      ---------- ---------- ---------- ---------- ---------- ----------
2e_Thai        TCCATATAAT CCTGAAATTT ATGCTTTTAG AGAACTGAGA AGTTATGCAC TACTTGACGA
Conservation
```

```
              ....|....| ....|....| ....|....| ....|....| ....|....| ....|....|
                 605       615       625       635       645       655
2e_Brazil     ---------- ---------- ---------- ---------- ---------- ----------
2e_Thai       AGAAATGAAG AGAGCAATCG ACGAAACGTA TAATGTCGAA ATACCTCGCA TTGAAAACAT
Conservation

              ....|....| ....|....| ....|....| ....|....| ....|....| ....|....|
                 665       675       685       695       705       715
2e_Brazil     ---------- ---------- ---------- ---------- ---------- ----------
2e_Thai       TGTGTCTCAA AGTTTGAATG AATGCTCAGA TCAAATAAGA CAATTGAATA CATTAATATA
Conservation

              ....|....| ....|....| ....|....| ....|....| ....|....| ....|....|
                 725       735       745       755       765       775
2e_Brazil     ---------- ---------- ---------- ---------- ---------- ----------
2e_Thai       TCATCTAGAA AATCCAGAAG GACATGGAAT TCAAGAACGT GTATACGGTA CTAAGAAGCA
Conservation

              ....|....| ....|....| ....|....| ....|....| ....|....| ....|....|
                 785       795       805       815       825       835
2e_Brazil     ---------- ---------- ---------- ---------- ---------- ----------
2e_Thai       AGAGTACAAA AACAGACTTC AATCTTTACA TCAATGCATA AAAGAAAAAT ATAAGAAAAA
Conservation

              ....|....| ....|....| ....|....| ....|....| ....|....| ....|....|
                 845       855       865       875       885       895
2e_Brazil     ---------- ---------- ---------- ---------- ---------- ----------
2e_Thai       TGAGAAAGAA ATTTTACTAA GACAAATATC TACGAAACTA TAAAGGATAT ATCGTGCAAC
Conservation

              ....|....| ....|....| ....|....| ....|....| ....|....| ....|....|
                 905       915       925       935       945       955
2e_Brazil     ---------- ---------- ---------- ---------- ---------- ----------
2e_Thai       TGGATACACT TGTGTTCAAC AGATATATAT TATAATATGA TTAAGATACG TGAGATAAAT
Conservation

              ....|....| ....|....| ....|....| ....|....| ....|....| ....|....|
                 965       975       985       995       1005      1015
2e_Brazil     ---------- ---------- ---------- ---------- ---------- ----------
2e_Thai       GTTAAAGAAT ATGACGTTAG ATCAATTACT TCATACTTTA ATGCAACTAA GAAAATACAT
Conservation

              ....|....| ....|....| ....|....| ....|....| ....|....| ....|....|
                 1025      1035      1045      1055      1065      1075
2e_Brazil     ---------- ---------- ---------- ---------- ---------- ----------
2e_Thai       AGGTCTGGTG AAGATACAAT AAAGAGAGTA AAAAAACAGT TAAATAATAG TCTTGCCTAT
Conservation

              ....|....| ....|....| ....|....| ....|....| ....|....| ....|....|
                 1085      1095      1105      1115      1125      1135
2e_Brazil     ---------- ---------- ---------- ---------- --------AT GAGTCAACAC
2e_Thai       GAAACAGTAG AATTTATATT AGAAGAAATT AAATATATTA CAGAAAAAAT GAGTCAACAC
Conservation                                                      ** **********

              ....|....| ....|....| ....|....| ....|....| ....|....| ....|....|
                 1145      1155      1165      1175      1185      1195
2e_Brazil     TTAGAAAAAG TTAAGCAAGA AACTGAAACT ATAAATAAGC TTTCTAAACA AGAAGTCTGG
2e_Thai       TTAGAAAAAG TTAAGCAAGA AACTGAAACT ATAAATAAGC TTTCTAAACA AGAAGTCTGG
Conservation  ********** ********** ********** ********** ********** **********
```

```
             ....|....| ....|....| ....|....| ....|....| ....|....| ....|....|
                 1205       1215       1225       1235       1245       1255
2e_Brazil    TCCCACTTCT TTCGATTTAA TCTTCAAAGG GAATTATTCT TTTTAGCCTA CAGCTATTCC
2e_Thai      TCCCACTTCT TTCGATTTAA TCTTCAAAGG GAATTATTCT TTTTAGCCTA CAGCTATTCC
Conservation ********** ********** ********** ********** ********** **********

             ....|....| ....|....| ....|....| ....|....| ....|....| ....|....|
                 1265       1275       1285       1295       1305       1315
2e_Brazil    AGTTTTAAAT TTAGTACTGA CAATTTAAGA GTCTTGAAAA ATGTGTCCGA GAGCAAAACA
2e_Thai      AGTTTTAAAT TTAGTACTGA CAATTTAAGA GTCTTGAAAA ATGTGTCCGA GAGCAAAACA
Conservation ********** ********** ********** ********** ********** **********

             ....|....| ....|....| ....|....| ....|....| ....|....| ....|....|
                 1325       1335       1345       1355       1365       1375
2e_Brazil    GGAAAACTTT ATCAATATTT TGCAAAATTC GAGGGCAGTC TCAACCACAT AATAAATACT
2e_Thai      GGAAAACTTT ATCAATATTT TGCAAAATTC GAGGGCAGTC TCAACCACAT AATAAATACT
Conservation ********** ********** ********** ********** ********** **********

             ....|....| ....|....| ....|....| ....|....| ....|....| ....|....|
                 1385       1395       1405       1415       1425       1435
2e_Brazil    CTTATGTCCT CTAAATATTC CACGTCAAGT ATTAATTCTG TTCTCTCGGA TTCTGAGGAA
2e_Thai      CTTATGTCCT CTAAATATTC CACGTCAAGT ATTAATTCTG TTCTCTCGGA TTCTGAGGAA
Conservation ********** ********** ********** ********** ********** **********

             ....|....| ....|....| ....|....| ....|....| ....|....| ....|....|
                 1445       1455       1465       1475       1485       1495
2e_Brazil    ATTTTAAAAT CTGTAGAAAC ATTACGCAGT TCTACGTCCG AGCAGATAAA AGAACATGTA
2e_Thai      ATTTTAAAAT CTGTAGAAAC ATTACGCAGT TCTACGTCCG AGCAGATAAA AGAACATGTA
Conservation ********** ********** ********** ********** ********** **********

             ....|....| ....|....| ....|....| ....|....| ....|....| ....|....|
                 1505       1515       1525       1535       1545       1555
2e_Brazil    CTTTATTCGA ATGCCCAAAT AGAAGAGGCT AAGAAAATTT TAGATGAAAA AATGCTTAAA
2e_Thai      CTTTATTCGA ATGCCCAAAT AGAAGAGGCT AAGAAAATTT TAGATGAAAA AATGCTTAAA
Conservation ********** ********** ********** ********** ********** **********

             ....|....| ....|....| ....|....| ....|....| ....|....| ....|....|
                 1565       1575       1585       1595       1605       1615
2e_Brazil    TTAGAAGAAT ATGCACAAGA AACAAAAGGG GTAATTAAAA CAGTAAATGA AACATTTAAA
2e_Thai      TTAGAAGAAT ATGCACAAGA AACAAAAGGG GTAATTAAAA CAGTAAATGA AACATTTAAA
Conservation ********** ********** ********** ********** ********** **********

             ....|....| ....|....| ....|....| ....|....| ....|....| ....|....|
                 1625       1635       1645       1655       1665       1675
2e_Brazil    TTAAATATAA TAGCAAGTGA AGAAATAGAA AAGAAAAGAC GCGAAACTAA CAAAAACAAA
2e_Thai      TTAAATATAA TAGCAAGTGA AGAAATAGAA AAGAAAAGAC GCGAAACTAA CAAAAACAAA
Conservation ********** ********** ********** ********** ********** **********

             ....|....| ....|....| ....|....| ....|....| ....|....| ....|....|
                 1685       1695       1705       1715       1725       1735
2e_Brazil    ACTTCTCTAG AAAAATGTAA GATATTCGTA GAAGTTATAG ACTTAATAAA ATCAAATAAA
2e_Thai      ACTTCTCTAG AAAAATGTAA GATATTCGTA GAAGTTATAG ACTTAATAAA ATCAAATAAA
Conservation ********** ********** ********** ********** ********** **********

             ....|....| ....|....| ....|....| ....|....| ....|....| ....|....|
                 1745       1755       1765       1775       1785       1795
2e_Brazil    TATAAGGCAA CCCAAAATAA TAAAAAAATA GAGGAACTTT CAAAACAGAT TATCACTATA
2e_Thai      TATAAGGCAA CCCAAAATAA TAAAAAAATA GAGGAACTTT CAAAACAGAT TATCACTATA
Conservation ********** ********** ********** ********** ********** **********
```

```
             ....|....| ....|....| ....|....| ....|....| ....|....| ....|....|
                  1805       1815       1825       1835       1845       1855
2e_Brazil    AAAAGCAATT TTCAACAATC AGAGACAGCT ATTAACGAAG ATGTGCAAAA AATTAAAGAA
2e_Thai      AAAAGCAATT TTCAACAATC AGAGACAGCT ATTAACGAAG ATGTGCAAAA AATTAAAGAA
Conservation ********** ********** ********** ********** ********** **********

             ....|....| ....|....| ....|....| ....|....| ....|....| ....|....|
                  1865       1875       1885       1895       1905       1915
2e_Brazil    TTAATTGAAA ATGAAAAAAA ACGTCGTTCC ATGAAAGATG AAATAAAAAA ACATTTGAAA
2e_Thai      TTAATTGAAA ATGAAAAAAA ACGTCGTTCC ATGAAAGATG AAATAAAAAA ACATTTGAAA
Conservation ********** ********** ********** ********** ********** **********

             ....|....| ....|....| ....|....| ....|....| ....|....| ....|....|
                  1925       1935       1945       1955       1965       1975
2e_Brazil    TATTTTCCTG AGACGGTGGA TAACATAAAA AAAATCATTT CCCAAAGGGA AGAAATAGAA
2e_Thai      TATTTTCCTG AGACGGTGGA TAACATAAAA AAAATCATTT CCCAAAGGGA AGAAATAGAA
Conservation ********** ********** ********** ********** ********** **********

             ....|....| ....|....| ....|....| ....|....| ....|....| ....|....|
                  1985       1995       2005       2015       2025       2035
2e_Brazil    AAATATATTC TAAGCATTGA AAATTTGATT AAAGACGCGC CCTCTGCTGT GGACGAATTT
2e_Thai      AAATATATTC TAAGCATTGA AAATTTGATT AAAGACGCGC CCTCTGCTGT GGACGAATTT
Conservation ********** ********** ********** ********** ********** **********

             ....|....| ....|....| ....|....| ....|....| ....|....| ....|....|
                  2045       2055       2065       2075       2085       2095
2e_Brazil    ACAACTGAAA AAACGGAATT GCAAGACAAA GCAAAATCAA TCGTTGATTC ATTTTATAAG
2e_Thai      ACAACTGAAA AAACGGAATT GCAAGACAAA GCAAAATCAA TCGTTGATTC ATTTTATAAG
Conservation ********** ********** ********** ********** ********** **********

             ....|....| ....|....| ....|....| ....|....| ....|....| ....|....|
                  2105       2115       2125       2135       2145       2155
2e_Brazil    GAAAATTTAC AAACATTTGT AGACGATTTA GCAGAATTTT ATACAAAACA TCAAGCCTTA
2e_Thai      GAAAATTTAC AAACATTTGT AGACGATTTA GCAGAATTTT ATACAAAACA TCAAGCCTTA
Conservation ********** ********** ********** ********** ********** **********

             ....|....| ....|....| ....|....| ....|....| ....|....| ....|....|
                  2165       2175       2185       2195       2205       2215
2e_Brazil    GACAAGGAAG AAAATACTAA AGAAGAAACT GAAGCATTGT ACGAAAAAAC GAAAGAAAAA
2e_Thai      GACAAGGAAG AAAATACTAA AGAAGAAACT GAAGCATTGT ACGAAAAAAC GAAAGAAAAA
Conservation ********** ********** ********** ********** ********** **********

             ....|....| ....|....| ....|....| ....|....| ....|....| ....|....|
                  2225       2235       2245       2255       2265       2275
2e_Brazil    TATCAACAGC TTGTAGATAT GAAGTGCGAT GGTATACCAG AAATGTTAAA CAATTTGAGT
2e_Thai      TATCAACAGC TTGTAGATAT GAAGTGCGAT GGTATACCAG AAATGTTAAA CAATTTGAGT
Conservation ********** ********** ********** ********** ********** **********

             ....|....| ....|....| ....|....| ....|....| ....|....| ....|....|
                  2285       2295       2305       2315       2325       2335
2e_Brazil    GAAGTGTTAA ATAGTCTCAC ACAGCTAAAG AACAAAATTA TAGAGGGGAA TATTCAAAAT
2e_Thai      GAAGTGTTAA ATAGTCTCAC ACAGCTAAAG AACAAAATTA TAGAGGGGAA TATTCAAAAT
Conservation ********** ********** ********** ********** ********** **********

             ....|....| ....|....| ....|....| ....|....| ....|....| ....|....|
                  2345       2355       2365       2375       2385       2395
2e_Brazil    ATCAAAAAAG ATGTGTCAAG TTCGTTAAAG AATCTAACTG AAGAAACAGA CCGATTAAAA
2e_Thai      ATCAAAAAAG ATGTGTCAAG TTCGTTAAAG AATCTAACTG AAGAAACAGA CCGATTAAAA
Conservation ********** ********** ********** ********** ********** **********
```

```
             ....|....| ....|....| ....|....| ....|....| ....|....| ....|....|
                  2405       2415       2425       2435       2445       2455
2e_Brazil    AATAGCCTGA AGGACTACGA GCAAAGTGCA GATAAACTGA AAACCTACAT AAATAGCATA
2e_Thai      AATAGCCTGA AGGACTACGA GCAAAGTGCA GATAAACTGA AAACCTACAT AAATAGCATA
Conservation ********** ********** ********** ********** ********** **********

             ....|....| ....|....| ....|....| ....|....| ....|....| ....|....|
                  2465       2475       2485       2495       2505       2515
2e_Brazil    AACGGGATAA AAGACAAAGT TCGAACTACT TTAATCGAAA AAGATGAAGA CATTCCAGAA
2e_Thai      AACGGGATAA AAGACAAAGT TCGAACTACT TTAATCGAAA AAGATGAAGA CATTCCAGAA
Conservation ********** ********** ********** ********** ********** **********

             ....|....| ....|....| ....|....| ....|....| ....|....| ....|....|
                  2525       2535       2545       2555       2565       2575
2e_Brazil    TTAAAAACTA CCTATGAAGA ATATACAAAG CAAAAGGGCG AAG------- ----------
2e_Thai      TTAAAAACTA CCTATGAAGA ATATACAAAG CAAAAGGGCG AAGGTACTAA TAAGGAAAGC
Conservation ********** ********** ********** ********** ***

             ....|....| ....|....| ....|....| ....|....| ....|....| ....|....|
                  2585       2595       2605       2615       2625       2635
2e_Brazil    ---------- ---------- ---------- ---------- ---------- ----------
2e_Thai      ACAGTAACAC ACGATATTGA CGTACTCAAA GAAAATATAA GAAAGGCTAA CGAACAAATT
Conservation

             ....|....| ....|....| ....|....| ....|....| ....|....| ....|....|
                  2645       2655       2665       2675       2685       2695
2e_Brazil    ---------- ---------- ---------- ---------- ---------- ----------
2e_Thai      TAAACTTACA AAAACGTAAT GCAAAGATTA GAAACACAGA ATAGTGAGAC ATATCAGGAA
Conservation

             ....|....| ....|....| ....|....| ....|....| ....|....| ....|....|
                  2705       2715       2725       2735       2745       2755
2e_Brazil    ---------- ------AAGG TTTTAACACT AAAATCGAAA GTTTAGAATT TGAAAAACTT
2e_Thai      ACTAAAACTT TGTTAGAAGG TTTTAACACT AAAATCGAAA GTTTAGAATT TGAAAAACTT
Conservation                  **** ********** ********** ********** **********

             ....|....| ....|....| ....|....| ....|....| ....|....| ....|....|
                  2765       2775       2785       2795       2805       2815
2e_Brazil    CAAGATGAAT TAAGGAGTCA CAAAGAATCC GCTAGTAGTA CCATAAACCA AATAGAAAAT
2e_Thai      CAAGATGAAT TAAGGAGTCA CAAAGAATCC GCTAGTAGTA CCATAAACCA AATAGAAAAT
Conservation ********** ********** ********** ********** ********** **********

             ....|....| ....|....| ....|....| ....|....| ....|....| ....|....|
                  2825       2835       2845       2855       2865       2875
2e_Brazil    ACAAAAAAAG TTATAGACAC GCTGAAGTCT TTAAATACTC TCACAAAGAA CTCCAACACA
2e_Thai      ACAAAAAAAG TTATAGACAC GCTGAAGTCT TTAAATACTC TCACAAAGAA CTCCAACACA
Conservation ********** ********** ********** ********** ********** **********

             ....|....| ....|....| ....|....| ....|....| ....|....| ....|....|
                  2885       2895       2905       2915       2925       2935
2e_Brazil    AACGAACAAT CGATCGCAAA AATAAAAGAG AGAAAGAATG ATTTAGTAAA AAAAATAGAT
2e_Thai      AACGAACAAT CGATCGCAAA AATAAAAGAG AGAAAGAATG ATTTAGTAAA AAAAATAGAT
Conservation ********** ********** ********** ********** ********** **********

             ....|....| ....|....| ....|....| ....|....| ....|....| ....|....|
                  2945       2955       2965       2975       2985       2995
2e_Brazil    AAACATATTG AAGCATTAAA TAGTTACAAC ATAGTAGAAG ATAAAGAAAG AACATCCCTT
2e_Thai      AAACATATTG AAGCATTAAA TAGTTACAAC ATAGTAGAAG ATAAAGAAAG AACATCCCTT
Conservation ********** ********** ********** ********** ********** **********
```

```
                ....|....| ....|....| ....|....| ....|....| ....|....| ....|....|
                     3005       3015       3025       3035       3045       3055
2e_Brazil       TTGAACACTT TAAATGAAGA AAAAAATAAT GTAGAAAGTG AAATACCTGA AACATCAATA
2e_Thai         TTGAACACTT TAAATGAAGA AAAAAATAAT GTAGAAAGTG AAATACCTGA AACATCAATA
Conservation    ********** ********** ********** ********** ********** **********

                ....|....| ....|....| ....|....| ....|....| ....|....| ....|....|
                     3065       3075       3085       3095       3105       3115
2e_Brazil       TCTAAATTGC AAGTAGATGT AAAAGCTTCA CAAGATTACT GTAAAAATGA AAAGATCAAT
2e_Thai         TCTAAATTGC AAGTAGATGT AAAAGCTTCA CAAGATTACT GTAAAAATGA AAAGATCAAT
Conservation    ********** ********** ********** ********** ********** **********

                ....|....| ....|....| ....|....| ....|....| ....|....| ....|....|
                     3125       3135       3145       3155       3165       3175
2e_Brazil       ATCGACAAAG GCATGGAAAC CAATTCGGAA GAACTGGGAA AATATAAAAC TGAGTGCCAT
2e_Thai         ATCGACAAAG GCATGGAAAC CAATTCGGAA GAACTGGGAA AATATAAAAC TGAGTGCCAT
Conservation    ********** ********** ********** ********** ********** **********

                ....|....| ....|....| ....|....| ....|....| ....|....| ....|....|
                     3185       3195       3205       3215       3225       3235
2e_Brazil       AACACAGAAC TGGAAATAAA CAAATTAAAC GCCAATTGTC AAGCATTAGA AAAAAAAATA
2e_Thai         AACACAGAAC TGGAAATAAA CAAATTAAAC GCCAATTGTC AAGCATTAGA AAAAAAAATA
Conservation    ********** ********** ********** ********** ********** **********

                ....|....| ....|....| ....|....| ....|....| ....|....| ....|....|
                     3245       3255       3265       3275       3285       3295
2e_Brazil       GAAGAGTTGG TACAAGATCA ACATGGCCAA ATTATAATAT TTGTTGATAA ACTTATAACA
2e_Thai         GAAGAGTTGG TACAAGATCA ACATGGCCAA ATTATAATAT TTGTTGATAA ACTTATAACA
Conservation    ********** ********** ********** ********** ********** **********

                ....|....| ....|....| ....|....| ....|....| ....|....| ....|....|
                     3305       3315       3325       3335       3345       3355
2e_Brazil       ACGAGGGACA CGCAGATAAA TGGAAAAATC GAGCTGAACC TAAATTCTCT AAAGGATATG
2e_Thai         ACGAGGGACA CGCAGATAAA TGGAAAAATC GAGCTGAACC TAAATTCTCT AAAGGATATG
Conservation    ********** ********** ********** ********** ********** **********

                ....|....| ....|....| ....|....| ....|....| ....|....| ....|....|
                     3365       3375       3385       3395       3405       3415
2e_Brazil       AAAACAAAAT TAGACGCTTT TAATTTTGAA GAAGCTGTTA ACAATAACGG AAACGTTACT
2e_Thai         AAAACAAAAT TAGACGCTTT TAATTTTGAA GAAGCTGTTA ACAATAACGG AAACGTTACT
Conservation    ********** ********** ********** ********** ********** **********

                ....|....| ....|....| ....|....| ....|....| ....|....| ....|....|
                     3425       3435       3445       3455       3465       3475
2e_Brazil       GTTCAAAGCA AAATAAGTGC TTTTAAAGAA AAAGTGAAAA GTACATTGCA AAATATAGAA
2e_Thai         GTTCAAAGCA AAATAAGTGC TTTTAAAGAA AAAGTGAAAA GTACATTGCA AAATATAGAA
Conservation    ********** ********** ********** ********** ********** **********

                ....|....| ....|....| ....|....| ....|....| ....|....| ....|....|
                     3485       3495       3505       3515       3525       3535
2e_Brazil       AATGAAATCG GCAAATTTAA CGGAATTAAA CAGAACTACG ATGCATATAT GGAAACATTC
2e_Thai         AATGAAATCG GCAAATTTAA CGGAATTAAA CAGAACTACG ATGCATATAT GGAAACATTC
Conservation    ********** ********** ********** ********** ********** **********

                ....|....| ....|....| ....|....| ....|....| ....|....| ....|....|
                     3545       3555       3565       3575       3585       3595
2e_Brazil       AACAAAGGAA AAGATCAGAT CACCGAATTC AAAGATAAAA AAGAGAACAT GGAAAAAATT
2e_Thai         AACAAAGGAA AAGATCAGAT CACCGAATTC AAAGATAAAA AAGAGAACAT GGAAAAAATT
Conservation    ********** ********** ********** ********** ********** **********
```

```
             ....|....| ....|....| ....|....| ....|....| ....|....| ....|....|
                  3605       3615       3625       3635       3645       3655
2e_Brazil    TATAAACAAA TGAAAGAAAC CACACTCAAC AAATTAGAAG AGGCAGAAAA AGAAAGTAGT
2e_Thai      TATAAACAAA TGAAAGAAAC CACACTCAAC AAATTAGAAG AGGCAGAAAA AGAAAGTAGT
Conservation ********** ********** ********** ********** ********** **********

             ....|....| ....|....| ....|....| ....|....| ....|....| ....|....|
                  3665       3675       3685       3695       3705       3715
2e_Brazil    ATATTAAACC AAGTAAAACA AATCGAATTG GAATACAGAA GAATTTTAAT TCATCATGCT
2e_Thai      ATATTAAACC AAGTAAAACA AATCGAATTG GAATACAGAA GAATTTTAAT TCATCATGCT
Conservation ********** ********** ********** ********** ********** **********

             ....|....| ....|....| ....|....| ....|....| ....|....| ....|....|
                  3725       3735       3745       3755       3765       3775
2e_Brazil    GTTCACATGA TCGATCAGGA AAACACAAAA GCTAAATCGG TTATGGCGGA TATCGAAACA
2e_Thai      GTTCACATGA TCGATCAGGA AAACACAAAA GCTAAATCGG TTATGGCGGA TATCGAAACA
Conservation ********** ********** ********** ********** ********** **********

             ....|....| ....|....| ....|....| ....|....| ....|....| ....|....|
                  3785       3795       3805       3815       3825       3835
2e_Brazil    TCTAAAAGCA AAATTGATGA AATAAAACTT CAAACTGAAG AATACAAACA GAAGAATATG
2e_Thai      TCTAAAAGCA AAATTGATGA AATAAAACTT CAAACTGAAG AATACAAACA GAAGAATATG
Conservation ********** ********** ********** ********** ********** **********

             ....|....| ....|....| ....|....| ....|....| ....|....| ....|....|
                  3845       3855       3865       3875       3885       3895
2e_Brazil    ACTAATTTTG AATATGAAAA TCATTACAGC AAGGCTAAAC AGAGCAACAC CAAAATAAAT
2e_Thai      ACTAATTTTG AATATGAAAA TCATTACAGC AAGGCTAAAC AGAGCAACAC CAAAATAAAT
Conservation ********** ********** ********** ********** ********** **********

             ....|....| ....|....| ....|....| ....|....| ....|....| ....|....|
                  3905       3915       3925       3935       3945       3955
2e_Brazil    GAATTTTCTA TAGACGCCAC AAGCGATAAG GAAAAGGCTG ACAAAAGCGA AGATGTAAAC
2e_Thai      GAATTTTCTA TAGACGCCAC AAGCGATAAG GAAAAGGCTG ACAAAAGCGA AGATGTAAAC
Conservation ********** ********** ********** ********** ********** **********

             ....|....| ....|....| ....|....| ....|....| ....|....| ....|....|
                  3965       3975       3985       3995       4005       4015
2e_Brazil    GAAATAGAAC GTATCAAAGA AAAAATTAAT GACAATCTGC AAAAAGTCAA AGAAGAATAT
2e_Thai      GAAATAGAAC GTATCAAAGA AAAAATTAAT GACAATCTGC AAAAAGTCAA AGAAGAATAT
Conservation ********** ********** ********** ********** ********** **********

             ....|....| ....|....| ....|....| ....|....| ....|....| ....|....|
                  4025       4035       4045       4055       4065       4075
2e_Brazil    ACTTCTATGG AGGAAATAAA AAAACAAATG AATAGTATGA AAGATTTACT AGCTTTGAAC
2e_Thai      ACTTCTATGG AGGAAATAAA AAAACAAATG AATAGTATGA AAGATTTACT AGCTTTGAAC
Conservation ********** ********** ********** ********** ********** **********

             ....|....| ....|....| ....|....| ....|....| ....|....| ....|....|
                  4085       4095       4105       4115       4125       4135
2e_Brazil    AATTCTGAAA CGACTGCTAC AAACATATTA GATAACACAA AAAACGCATT AGGTTTTAGC
2e_Thai      AATTCTGAAA CGACTGCTAC AAACATATTA GATAACACAA AAAACGCATT AGGTTTTAGC
Conservation ********** ********** ********** ********** ********** **********

             ....|....| ....|....| ....|....| ....|....| ....|....| ....|....|
                  4145       4155       4165       4175       4185       4195
2e_Brazil    GAGGAGGCGG CAAAGGAACT TGAGAAATCA AAACAATTAT TAGAAGAAGC GGTAGCTCAG
2e_Thai      GAGGAGGCGG CAAAGGAACT TGAGAAATCA AAACAATTAT TAGAAGAAGC GGTAGCTCAG
Conservation ********** ********** ********** ********** ********** **********

             ....|....| ....|....| ....|....| ....|....| ....|....| ....|....|
                  4205       4215       4225       4235       4245       4255
2e_Brazil    ATAGCTAAGG CAGGGGAACA TAAAGGTAAA ATTGAGATAG CCTTAGAAGA TGAACAAATA
```

```
2e_Thai      ATAGCTAAGG CAGGGGAACA TAAAGGTAAA ATTGAGATAG CCTTAGAAGA TGAACAAATA
Conservation ********** ********** ********** ********** ********** **********

             ....|....| ....|....| ....|....| ....|....| ....|....| ....|....|
                 4265       4275       4285       4295       4305       4315
2e_Brazil    AATGCCCACG TAAACCAGAT TGAACTAATA AAGAATGAAA TTGTGAATAA AAAAAAAGAA
2e_Thai      AATGCCCACG TAAACCAGAT TGAACTAATA AAGAATGAAA TTGTGAATAA AAAAAAAGAA
Conservation ********** ********** ********** ********** ********** **********

             ....|....| ....|....| ....|....| ....|....| ....|....| ....|....|
                 4325       4335       4345       4355       4365       4375
2e_Brazil    ATGGAGTCCT ATTTAAGCAA GATAAAAGAA TATAAAGAGA AATGCACCAC CCAAGTCAAC
2e_Thai      ATGGAGTCCT ATTTAAGCAA GATAAAAGAA TATAAAGAGA AATGCACCAC CCAAGTCAAC
Conservation ********** ********** ********** ********** ********** **********

             ....|....| ....|....| ....|....| ....|....| ....|....| ....|....|
                 4385       4395       4405       4415       4425       4435
2e_Brazil    AATTCGAATA GAGGAAAAGA TAAAATTGAA TTCTTGAAAA CGGTTAAGCA CAGCGTAGAA
2e_Thai      AATTCGAATA GAGGAAAAGA TAAAATTGAA TTCTTGAAAA CGGTTAAGCA CAGCGTAGAA
Conservation ********** ********** ********** ********** ********** **********

             ....|....| ....|....| ....|....| ....|....| ....|....| ....|....|
                 4445       4455       4465       4475       4485       4495
2e_Brazil    AGTAGTTCGA ATAAGGTTGA CATGGATCAG ATAAATGAAA GTATAGAAAA ATCCGAACAA
2e_Thai      AGTAGTTCGA ATAAGGTTGA CATGGATCAG ATAAATGAAA GTATAGAAAA ATCCGAACAA
Conservation ********** ********** ********** ********** ********** **********

             ....|....| ....|....| ....|....| ....|....| ....|....| ....|....|
                 4505       4515       4525       4535       4545       4555
2e_Brazil    TATTTAAAGG ATGTAGAAGC TCTGGAGACA CAAGCTGATA ACAATCTAAA GTCATACATA
2e_Thai      TATTTAAAGG ATGTAGAAGC TCTGGAGACA CAAGCTGATA ACAATCTAAA GTCATACATA
Conservation ********** ********** ********** ********** ********** **********

             ....|....| ....|....| ....|....| ....|....| ....|....| ....|....|
                 4565       4575       4585       4595       4605       4615
2e_Brazil    CAACATGAAC AAACGATCAA TGATACTTTC CAGCAATCTG AAATTCTAGG AATAGAAACT
2e_Thai      CAACATGAAC AAACGATCAA TGATACTTTC CAGCAATCTG AAATTCTAGG AATAGAAACT
Conservation ********** ********** ********** ********** ********** **********

             ....|....| ....|....| ....|....| ....|....| ....|....| ....|....|
                 4625       4635       4645       4655       4665       4675
2e_Brazil    AAATCAAAAA AGAAGTTCAA CGAAGCAACA GCAATAATGG AAGAAATTAA AAAACAAAAT
2e_Thai      AAATCAAAAA AGAAGTTCAA CGAAGCAACA GCACTAATGG AAGAAATTAA AAAACAAAAT
Conservation ********** ********** ********** ***.****** ********** **********

             ....|....| ....|....| ....|....| ....|....| ....|....| ....|....|
                 4685       4695       4705       4715       4725       4735
2e_Brazil    TCTGAAATTC AAACAAAGGT GAATGATTTC CAAGTAAAAC TGAACAAATT AAAGGAACCC
2e_Thai      TCTGAAATTC AAACAAAGGT GAATGATTTC CAAGTAAAAC TGAACAAATT AAAGGAACCC
Conservation ********** ********** ********** ********** ********** **********

             ....|....| ....|....| ....|....| ....|....| ....|....| ....|....|
                 4745       4755       4765       4775       4785       4795
2e_Brazil    CAAAATGCCG AAGACGTAGA AGAGGAGCTT AATAATGAAA AGTCTTCCAA TGCAAAAGCA
2e_Thai      CAAAATGCCG AAGACGTAGA AGAGGAGCTT AATAATGAAA AGTCTTCCAA TGCAAAAGCA
Conservation ********** ********** ********** ********** ********** **********

             ....|....| ....|....| ....|....| ....|....| ....|....| ....|....|
                 4805       4815       4825       4835       4845       4855
2e_Brazil    CTTATACACA CGAACCTAGA AAGAGTGCAA TCTAATTTAA CCCAAGTTGC CAATATTAAG
2e_Thai      CTTATACACA CGAACCTAGA AAGAGTGCAA TCTAATTTAA CCCAAGTTGC CAATATTAAG
Conservation ********** ********** ********** ********** ********** **********
```

```
             ....|....| ....|....| ....|....| ....|....| ....|....| ....|....|
                4865       4875       4885       4895       4905       4915
2e_Brazil    CAGGAAGTGG ATGGCATATT TAAGAGAGTC ACAGACACGA TGAATTCCGC AACAGAAACC
2e_Thai      CAGGAAGTGG ATGGCATATT TAAGAGAGTC ACAGACACGA TGAATTCCGC AACAGAAACC
Conservation ********** ********** ********** ********** ********** **********

             ....|....| ....|....| ....|....| ....|....| ....|....| ....|....|
                4925       4935       4945       4955       4965       4975
2e_Brazil    TCTCAAAATA AAGATGGCAA AACATTAGGC ACGGTGAAAA GGGACGAACC TAAATATGAA
2e_Thai      TCTCAAAATA AAGATGGCAA AACATTAGGC ACGGTGAAAA GGGACGAACC TAAATATGAA
Conservation ********** ********** ********** ********** ********** **********

             ....|....| ....|....| ....|....| ....|....| ....|....| ....|....|
                4985       4995       5005       5015       5025       5035
2e_Brazil    GAGTACTTAA CCCAAATAAC TGCAGATAAA AATCTAATGG ACGCAGAATT GAATAAGCTA
2e_Thai      GAGTACTTAA CCCAAATAAC TGCAGATAAA AATCTAATGG ACGCAGAATT GAATAAGCTA
Conservation ********** ********** ********** ********** ********** **********

             ....|....| ....|....| ....|....| ....|....| ....|....| ....|....|
                5045       5055       5065       5075       5085       5095
2e_Brazil    AACGGAATAA GTTCCACTAT CGAAATCTTA GAAAAGGAAT TGAATGAATC CAGAATGAAT
2e_Thai      AACGGAATAA GTTCCACTAT CGAAATCTTA GAAAAGGAAT TGAATGAATC CAGAATGAAT
Conservation ********** ********** ********** ********** ********** **********

             ....|....| ....|....| ....|....| ....|....| ....|....| ....|....|
                5105       5115       5125       5135       5145       5155
2e_Brazil    TACGAAATTG GACTCCTGCA AAAGATAGAT GAAGTAGGCA AAAGTAAGAA GACAAATATT
2e_Thai      TACGAAATTG GACTCCTGCA AAAGATAGAT GAAGTAGGCA AAAGTAAGAA GACAAATATT
Conservation ********** ********** ********** ********** ********** **********

             ....|....| ....|....| ....|....| ....|....| ....|....| ....|....|
                5165       5175       5185       5195       5205       5215
2e_Brazil    GATTTAAAAA AAGAGTTAAT AAGTTCAACG CTGAATTCCT TTTCTTCCCT GTTCAATGGT
2e_Thai      GATTTAAAAA AAGAGTTAAT AAGTTCAACG CTGAATTCCT TTTCTTCCCT GTTCAATGGT
Conservation ********** ********** ********** ********** ********** **********

             ....|....| ....|....| ....|....| ....|....| ....|....| ....|....|
                5225       5235       5245       5255       5265       5275
2e_Brazil    CTCGATTTAA ATCAATACGA ATTTAAGAAA AACATAGATG GTTACGAACA GAAGATGAAA
2e_Thai      CTCGATTTAA ATCAATACGA ATTTAAGAAA AACATAGATG GTTACGAACA GAAGATGAAA
Conservation ********** ********** ********** ********** ********** **********

             ....|....| ....|....| ....|....| ....|....| ....|....| ....|....|
                5285       5295       5305       5315       5325       5335
2e_Brazil    GAAATACAAG GCAAATTTGA CGTATCGCTG AACAAAATTG GTGAACATTT AAAAAAGGCT
2e_Thai      GAAATACAAG GCAAATTTGA CGTATCGCTG AACAAAATTG GTGAACATTT AAAAAAGGCT
Conservation ********** ********** ********** ********** ********** **********

             ....|....| ....|....| ....|....| ....|....| ....|....| ....|....|
                5345       5355       5365       5375       5385       5395
2e_Brazil    TCAGAGGAGG GCGCAAATTA TACCTTAGCG AATAAACTCA GACGAGAAGC TCAACAGGAA
2e_Thai      TCAGAGGAGG GCGCAAATTA TACCTTAGCG AATAAACTCA GACGAGAAGC TCAACAGGAA
Conservation ********** ********** ********** ********** ********** **********

             ....|....| ....|....| ....|....| ....|....| ....|....| ....|....|
                5405       5415       5425       5435       5445       5455
2e_Brazil    AAAGCCAAAC TTACAAGTAG CGAAGATGAA GCAAGCAAAT ACATAGAAGA TATTAAAAAG
2e_Thai      AAAGCCAAAC TTACAAGTAG CGAAGATGAA GCAAGCAAAT ACATAGAAGA TATTAAAAAG
Conservation ********** ********** ********** ********** ********** **********

             ....|....| ....|....| ....|....| ....|....| ....|....| ....|....|
                5465       5475       5485       5495       5505       5515
2e_Brazil    GTAGAATCCA TAAGATTTAT AAATCACATG AAAGAAAATT TAGACAAAAT AAGCATATCG
```

```
2e_Thai      GTAGAATCCA TAAGATTTAT AAATCACATG AAAGAAAATT TAGACAAAAT AAGCACATCG
Conservation ********** ********** ********** ********** ********** ***** ****

             ....|....| ....|....| ....|....| ....|....| ....|....| ....|....|
                 5525       5535       5545       5555       5565       5575
2e_Brazil    ATCAAAGAAG AAAAATTAAA AATTAATGAA GGACATGAAT TCATCAAACA GATAGTTGAA
2e_Thai      ATCAAAGAAG AAAAATTAAA AATTAATGAA GGACATGAAT TCATCAAACA GATAGTTGAA
Conservation ********** ********** ********** ********** ********** **********

             ....|....| ....|....| ....|....| ....|....| ....|....| ....|....|
                 5585       5595       5605       5615       5625       5635
2e_Brazil    GGCATTAAAA ACACAGATGA TGAACGCGAT GTATCAAACA AATTACAACA GGCGGAAGGA
2e_Thai      GGCATTAAAA ACACAGATGA TGAACGCGAT GTATCAAACA AATTACAACA GGCGGAAGGA
Conservation ********** ********** ********** ********** ********** **********

             ....|....| ....|....| ....|....| ....|....| ....|....| ....|....|
                 5645       5655       5665       5675       5685       5695
2e_Brazil    AAAAATGAAG AAATGCAGAA AATAATGCAC TCTAAGCATA AAAACGAGGC AAAAGAAATG
2e_Thai      AAAAATGAAG AAATGCAGAA AATAATGCAC TCTAAGCATA AAAACGAGGC AAAAGAAATG
Conservation ********** ********** ********** ********** ********** **********

             ....|....| ....|....| ....|....| ....|....| ....|....| ....|....|
                 5705       5715       5725       5735       5745       5755
2e_Brazil    CTAGGACATA TAGCTACCTC TGCAAATTTT ATAAATATTA AAATAATTCC AGAATTAACT
2e_Thai      CTAGGACATA TAGCTACCTC TGCAAATTTT ATAAATATTA AAATAATTCC AGAATTAACT
Conservation ********** ********** ********** ********** ********** **********

             ....|....| ....|....| ....|....| ....|....| ....|....| ....|....|
                 5765       5775       5785       5795       5805       5815
2e_Brazil    CTAACCGAGT CAAACGTAAA CGAAGCAGTA GAACTAAAAT TTCAACCGGA TGGCAAAGTA
2e_Thai      CTAACCGAGT CAAACGTAAA CGAAGCAGTA GAACTAAAAT TTCAACCGGA TGGCAAAGTA
Conservation ********** ********** ********** ********** ********** **********

             ....|....| ....|....| ....|....| ....|....| ....|....| ....|....|
                 5825       5835       5845       5855       5865       5875
2e_Brazil    ACCTTAAAAA CAAGAAACAT CTCGAAAAGC GAAGCCGAAT TGGATGTGCA TAAAAATATA
2e_Thai      ACCTTAAAAA CAAGAAACAT CTCGAAAAGC GAAGCCGAAT TGGATGTGCA TAAAAATATA
Conservation ********** ********** ********** ********** ********** **********

             ....|....| ....|....| ....|....| ....|....| ....|....| ....|....|
                 5885       5895       5905       5915       5925       5935
2e_Brazil    AAGGAAGCTT ACCATCTCGC TTTGGAGATA CAAAAATATG CAAACGAAAT AGACGCACAA
2e_Thai      AAGGAAGCTT ACCATCTCGC TTTGGAGATA CAAAAATATG CAAACGAAAT AGACGCACAA
Conservation ********** ********** ********** ********** ********** **********

             ....|....| ....|....| ....|....| ....|....| ....|....| ....|....|
                 5945       5955       5965       5975       5985       5995
2e_Brazil    CAAACGAATA ACACACAGTT AATGGTCACA GTAAACGACC TACATCATAA AATTAAGTTC
2e_Thai      CAAACGAATA ACACACAGTT AATGGTCACA GTAAACGACC TACATCATAA AATTAAGTTC
Conservation ********** ********** ********** ********** ********** **********

             ....|....| ....|....| ....|....| ....|....| ....|....| ....|....|
                 6005       6015       6025       6035       6045       6055
2e_Brazil    ATAAATGAAT TAAAAAATAA AGTAAAAATT GAAAAGAGTA ATGAAAATTC CCTATCGGGT
2e_Thai      ATAAATGAAT TAAAAAATAA AGTAAAAATT GAAAAGAGTA ATGAAAATTC CCTATCGGGT
Conservation ********** ********** ********** ********** ********** **********

             ....|....| ....|....| ....|....| ....|....| ....|....| ....|....|
                 6065       6075       6085       6095       6105       6115
2e_Brazil    AAAGTAATGG GCATTTCTAA CAAAATTGCC GAATTAGACA AACATTCATG CAGTGAAAAA
2e_Thai      AAAGTAATGG GCATTTCTAA CAAAATTGCC GAATTAGACA AACATTCATG CAGTGAAAAA
Conservation ********** ********** ********** ********** ********** **********
```

```
               ....|....| ....|....| ....|....| ....|....| ....|....| ....|....|
                   6125       6135       6145       6155       6165       6175
2e_Brazil      AGTTATGATA AATTTTTAGA AAACACGAAG AAAACAGATC TACAAAATAT ACGTGATTCC
2e_Thai        AGTTATGATA AATTTTTAGA AAACACGAAG AAAACAGATC TACAAAATAT ACGTGATTCC
Conservation   ********** ********** ********** ********** ********** **********

               ....|....| ....|....| ....|....| ....|....| ....|....| ....|....|
                   6185       6195       6205       6215       6225       6235
2e_Brazil      TTTAATCAAG AAAAGAGAAA CGCAAATATT GACTTGAATT TGAAAAATAT AAAAGAAGAT
2e_Thai        TTTAATCAAG AAAAGAGAAA CGCAAATATT GACTTGAATT TGAAAAATAT AAAAGAAGAT
Conservation   ********** ********** ********** ********** ********** **********

               ....|....| ....|....| ....|....| ....|....| ....|....| ....|....|
                   6245       6255       6265       6275       6285       6295
2e_Brazil      TTTGCCAATT CACAAACATC CTTAAAAATT GTTGAACAGG AAATTGAAGC TTTAAAGGCA
2e_Thai        TTTGCCAATT CACAAACATC CTTAAAAATT GTTGAACAGG AAATTGAAGC TTTAAAGGCA
Conservation   ********** ********** ********** ********** ********** **********

               ....|....| ....|....| ....|....| ....|....| ....|....| ....|....|
                   6305       6315       6325       6335       6345       6355
2e_Brazil      AATGAAATCA ACACTGAAAA TCTGCAAGGC AAATCTACTC AGATAGAAAC ACTACATAAC
2e_Thai        AATGAAATCA ACACTGAAAA TCTGCAAGGC AAATCTACTC AGATAGAAAC ACTACATAAC
Conservation   ********** ********** ********** ********** ********** **********

               ....|....| ....|....| ....|....| ....|....| ....|....| ....|....|
                   6365       6375       6385       6395       6405       6415
2e_Brazil      AAAATAGGAA GAATAGAACA AAATATTGCA AGTCTAAATA CGTCTTTGGA CAAATTACTA
2e_Thai        AAAATAGGAA GCATAGAACA AAATATTGCA AGTCTAAATA CGTCTTTGGA CAAATTACTA
Conservation   ********** *.******** ********** ********** ********** **********

               ....|....| ....|....| ....|....| ....|....| ....|....| ....|....|
                   6425       6435       6445       6455       6465       6475
2e_Brazil      AACAGTGGAA AAAAATGCGA AATAGCTAGA TACACATCCT TGAGGGATAG TATTAAAG--
2e_Thai        AACAGTGGAA AAAAATGCGA AATAGCTAGA TACACATCCT TGAGGGATAG TATTAAAGGT
Conservation   ********** ********** ********** ********** ********** ********

               ....|....| ....|....| ....|....| ....|....| ....|....| ....|....|
                   6485       6495       6505       6515       6525       6535
2e_Brazil      ---------- ---------- ---------- ---------- ---------- ----------
2e_Thai        AAAATAAATG CAGATGAAGA AACAATCGAT AATCTGCAGA AGCATGTTAG TCAATATTTG
Conservation

               ....|....| ....|....| ....|....| ....|....| ....|....| ....|....|
                   6545       6555       6565       6575       6585       6595
2e_Brazil      ---------- ---------- ---------- ---------- ---------- ----------
2e_Thai        ACATATGTTG AAGATAACTA TAATGCCACA GTGAAGGACG TCCTTACATT AAATGAACAT
Conservation

               ....|....| ....|....| ....|....| ....|....| ....|....| ....|....|
                   6605       6615       6625       6635       6645       6655
2e_Brazil      ---------- ---------- ---------- ---------- ---------- ----------
2e_Thai        TTNAGCAACA AAATGGTGAC TGATCATGCT GCGACTAATT TTGAAAAATC AAATAAAACT
Conservation

               ....|....| ....|....| ....|....| ....|....| ....|....| ....|....|
                   6665       6675       6685       6695       6705       6715
2e_Brazil      ---------- ---------- ---------- ---------- ---------- ----------
2e_Thai        TCGGAAGAAT TATCCACAGC GGTCAATCAA TCAAAGGCCA TAATAAACGA TATAAAAAAT
Conservation
```

```
              ....|....| ....|....| ....|....| ....|....| ....|....| ....|....|
                   6725       6735       6745       6755       6765       6775
2e_Brazil     ---------- ---------- ---------- ---------- ---------- ----------
2e_Thai       GCCTTCATAG AAGTTAACGA AAAAACGGAA TTTACTTTGT TAGAAAACAG CGCACAAGCA
Conservation

              ....|....| ....|....| ....|....| ....|....| ....|....| ....|....|
                   6785       6795       6805       6815       6825       6835
2e_Brazil     ---------- ---------- ---------- ---------- ----TGGACG AAATTTACAA
2e_Thai       ATAGAAAAGC TTTACAATAC ATTACATGAA AAAAAAAAAC GCAGTGGACG AAATTTACAA
Conservation                                                   ****** **********

              ....|....| ....|....| ....|....| ....|....| ....|....| ....|....|
                   6845       6855       6865       6875       6885       6895
2e_Brazil     AACATCCATT TTAGTTAAAT CGCAAAAAAT GAAATCAAAT GCTGATAAAT ATACCGATAT
2e_Thai       AACATCCATT TTAGTTAAAT CGCAAAAAAT GAAATCAAAT GCTGATAAAT ATACCGATAT
Conservation  ********** ********** ********** ********** ********** **********

              ....|....| ....|....| ....|....| ....|....| ....|....| ....|....|
                   6905       6915       6925       6935       6945       6955
2e_Brazil     GGCTAAAATA TTTGAAAACG TGTTAGACAC CCAAAAATCG AGAATTACAA GTAACCAGAG
2e_Thai       GGCTAAAATA TTTGAAAACG TGTTAGACAC CCAAAAATCG AGAATTACAA GTAACCAGAG
Conservation  ********** ********** ********** ********** ********** **********

              ....|....| ....|....| ....|....| ....|....| ....|....| ....|....|
                   6965       6975       6985       6995       7005       7015
2e_Brazil     TAGCGTATCC CACGTTAAAG ACGTGATAAA TGTACAGCTT AAGAAGTTAG AAGCTACGGA
2e_Thai       TAGCGTATCC CACGTTAAAG ATATGATAAA CGTACAGCTT AAGAAGTTAG AAGCTACGGA
Conservation  ********** ********** *..*******  ********* ********** **********

              ....|....| ....|....| ....|....| ....|....| ....|....| ....|....|
                   7025       7035       7045       7055       7065       7075
2e_Brazil     CAGCGCATTT AAATTAGAAT CCATAAATAA GTTTCACGAA TTAAGCGATA ACATCAAAAC
2e_Thai       CAGCGCATTT AAATTAGAAT CCATAAATAA GTTTCACGAA TTAAGCGATA ACATCAAAAA
Conservation  ********** ********** ********** ********** ********** *********.

              ....|....| ....|....| ....|....| ....|....| ....|....| ....|....|
                   7085       7095       7105       7115       7125       7135
2e_Brazil     GAGTATAGAT GAATTGGAAA AGCTAGAACA AGTTAATCGC CAAGAACATA ATACTGTGGA
2e_Thai       GAGTATAGAT GAATTGGAAA AGCTAGAACA AGTTAATCGC CAAGAACATA ATACTGTGGA
Conservation  ********** ********** ********** ********** ********** **********

              ....|....| ....|....| ....|....| ....|....| ....|....| ....|....|
                   7145       7155       7165       7175       7185       7195
2e_Brazil     AACGCATAAA GAACGAATTA CACATTTAAT GAACAGAAGA GACAGCCTCA AAAATGGAGT
2e_Thai       AACGCATAAA GAACGAATTA CACATTTAAT GAACAGAAGA GACAGCCTCA AAAATGGAGT
Conservation  ********** ********** ********** ********** ********** **********

              ....|....| ....|....| ....|....| ....|....| ....|....| ....|....|
                   7205       7215       7225       7235       7245       7255
2e_Brazil     GAAAGAATAC GGAGAAGATG CAAATTTAAA AAAATTAAGA GGGGATATCC TAAACCAGGT
2e_Thai       GAAAGAATAC GGAGAAGATG CAAATTTAAA AAAATTAAGA GGGGATATCC TAAACCAGGT
Conservation  ********** ********** ********** ********** ********** **********

              ....|....| ....|....| ....|....| ....|....| ....|....| ....|....|
                   7265       7275       7285       7295       7305       7315
2e_Brazil     AATTAGTGAC ATAAGAAATA TTAATGGAGA ACTTAACAAC TCAGATCAGC TGTACGTCAA
2e_Thai       AATTAGTGAC ATAAGAAATA TTAATGGAGA ACTTAACAAC TCAGATCAGC TGTACGTCAA
Conservation  ********** ********** ********** ********** ********** **********
```

```
             ....|....| ....|....| ....|....| ....|....| ....|....| ....|....|
                  7325       7335       7345       7355       7365       7375
2e_Brazil    ATTAATGAAA AAAGTGGACG AAAATAATGA TTTGTGTAAA AAGAACTATA CAGAAAATTA
2e_Thai      ATTAATGAAA AAAGTGGACG AAAATAATGA TTTGTGTAAA AAGAACTATA CAGAAAATTA
Conservation ********** ********** ********** ********** ********** **********

             ....|....| ....|....| ....|....| ....|....| ....|....| ....|....|
                  7385       7395       7405       7415       7425       7435
2e_Brazil    CGTTTCTGAA GTACTGCAAA AAATCGATAA TCTGAATAAA CGCTTCAAAC AAAATTTACC
2e_Thai      CGTTTCTGAA GTACTGCAAA AAATCGATAA TCTGAATAAA CGCTTCAAAC AAAATTTACC
Conservation ********** ********** ********** ********** ********** **********

             ....|....| ....|....| ....|....| ....|....| ....|....| ....|....|
                  7445       7455       7465       7475       7485       7495
2e_Brazil    AGAAAAGGAA AAAGTTTTAC AAATTGAGAG CAACTTTAAT GAAATTAAAA CCCTATTTGG
2e_Thai      AGAAAAGAAA AAAGTTTTAC AAATTGAGAA CAACTTTAAT GAAATTAAAA CCCTATTTGG
Conservation *******.** ********** *********. ********** ********** **********

             ....|....| ....|....| ....|....| ....|....| ....|....| ....|....|
                  7505       7515       7525       7535       7545       7555
2e_Brazil    TGAAATAAAA ACCTTTTATA AGGTAGAAGA ATTTGTAACG AACATGTATA GTCAAGTTAA
2e_Thai      TGAAATAAAA ACCTTTTATA AGGTAGAAGA ATTTGTAACG AACATGTATA GTCAAGTTAA
Conservation ********** ********** ********** ********** ********** **********

             ....|....| ....|....| ....|....| ....|....| ....|....| ....|....|
                  7565       7575       7585       7595       7605       7615
2e_Brazil    TGGTGAAAAG GAACGCGTAA AAGATCAGAC AAATATTGAA AAAATAAAAT TGGCAATACA
2e_Thai      TGGTGAAAAG GAACGCGTAA AAGATCAGAC AAATATTGAA AAAATAAAAT TGGCAATACA
Conservation ********** ********** ********** ********** ********** **********

             ....|....| ....|....| ....|....| ....|....| ....|....| ....|....|
                  7625       7635       7645       7655       7665       7675
2e_Brazil    AAATATTACA AATAACAATG AAGAAGTAAA AACCTATTTA TCCAAATTTA TTAGCACCCT
2e_Thai      AAATATTACA AATAACAATG AAGAAGTAAA AACCTATTTA TCCAAATTTA TTAGCACCCT
Conservation ********** ********** ********** ********** ********** **********

             ....|....| ....|....| ....|....| ....|....| ....|....| ....|....|
                  7685       7695       7705       7715       7725       7735
2e_Brazil    AGAAAGAATG AATATCATGA AAAAAGAAAT GGACGATTTA TCCAGCTCAC TGCCCACACA
2e_Thai      AGAAAGAATG AATATCATGA AAAAAGAAAT GGACGATTTA TCCAGCTCAC TGCCCACACA
Conservation ********** ********** ********** ********** ********** **********

             ....|....| ....|....| ....|....| ....|....| ....|....| ....|....|
                  7745       7755       7765       7775       7785       7795
2e_Brazil    TAATACAAAT GCAACAGAAA ATGCAAAACG GTTTGTAAAC AGTTCGGTAG AAATCATTAA
2e_Thai      TAATACAAAT GCAACAGAAA ATGCAAAACG GTTTGTAAAC AGTTCGGTAG AAATCATTAA
Conservation ********** ********** ********** ********** ********** **********

             ....|....| ....|....| ....|....| ....|....| ....|....| ....|....|
                  7805       7815       7825       7835       7845       7855
2e_Brazil    TGAGCTAACT AGCTATATTT CTAAAATAAC CGAACTAAAG AATTATGCTG AAGGTGTGAT
2e_Thai      TGAGCTAACT AGCTATATTT CTAAAATAAC CGAACTAAAG AATTATGCTG AAGGTGTGAT
Conservation ********** ********** ********** ********** ********** **********

             ....|....| ....|....| ....|....| ....|....| ....|....| ....|....|
                  7865       7875       7885       7895       7905       7915
2e_Brazil    AACCGAATTA GAAGAAGCAT CTAAGTTAAT ACGCCCAGCT GCTAATGACT CAAATGAGAC
2e_Thai      AACCGAATTA GAAGAAGCAT CTAAGTTAAT ACGCCCAGCT GCTAATGACT CAAATGAGAC
Conservation ********** ********** ********** ********** ********** **********
```

```
               ....|....| ....|....| ....|....| ....|....| ....|....| ....|....|
                    7925       7935       7945       7955       7965       7975
2e_Brazil      GCAAATGTTG CCAACCGAAG CTGATAGCAC ACATCAAGAC GATTCACAGA GTGATAATCG
2e_Thai        GCAAATGTTG CCAACCGAAG CTGATAGCAC ACATCAAGAC GATTCACAGA GTGATAATCG
Conservation   ********** ********** ********** ********** ********** **********

               ....|....| ....|....| ....|....| ....|....| ....|....| ....|....|
                    7985       7995       8005       8015       8025       8035
2e_Brazil      TTCAAGGGCA AGAGATGCAG ATGGAAGAAC TCGTTATGCA GCAGCTATTA TTGGTTTGTC
2e_Thai        TTCAAGGGCA AGAGATGCAG ATGGAAGAAC TCGTTATGCA GCAGCTATTA TTGGTTTGTC
Conservation   ********** ********** ********** ********** ********** **********

               ....|....| ....|....| ....|....| ....|....| ....|....| ....|....|
                    8045       8055       8065       8075       8085       8095
2e_Brazil      TCTTATTACC GGAGTTGCCC TATTAAGCCG CAGGCATAAT CCTGATGAAG AAGAGGATCA
2e_Thai        TCTTATTACC GGAGTTGCCC TATTAAGCCG CAGGCATAAT CCTGATGAAG AAGAGGATCA
Conservation   ********** ********** ********** ********** ********** **********

               ....|....| ....|....| ....|....| ....|....| ....|....| ....|....|
                    8105       8115       8125       8135       8145       8155
2e_Brazil      TCATGAACAC GGCTATCATG AGGCATTTGA GGGCAACGAC GACTACACTG TGCACGATAA
2e_Thai        TCATGAACAC GGCTATCATG AGGCATTTGA GGGCAACGAC GACTACACTG TGCACGATAA
Conservation   ********** ********** ********** ********** ********** **********

               ....|....| ....|....| ....|....| ....|....|
                    8165       8175       8185       8195
2e_Brazil      GGAAGAAGTC ATCGAAGTCT GTTTCAATGA CAGTGATTAG
2e_Thai        GGAAGAAGTC ATCGAAGTCT GTTTCAATGA CAGTGATTAG
Conservation   ********** ********** ********** **********
```

Alignment: Pvrbp3

```
             ....|....| ....|....| ....|....| ....|....| ....|....| ....|....|
                 5         15         25         35         45         55
3_SalI       ------ATGA ACCAAATTAT AGCAGAAGGG AGAGG----- ---------- -AAAGAAAAG
3_Thai       ATGAAGAAAA ACGGATGGTG GGGAGTATGC TTCGGGGTTC TTCTCTTTCT TATCGCTGGG
Conservation       *..* ** .*..... .* **.*.*  ...**                 *..*....*

             ....|....| ....|....| ....|....| ....|....| ....|....| ....|....|
                 65         75         85         95        105        115
3_SalI       GAAAAGCATT ACTG----TA AAACTGCAAA GTGAAGAGGA AACGTCTTCT TCTGTTAATT
3_Thai       GAAATGCCAT AAACGAATCG AATAAACACA AAAAGGAGGA AACGACTTCT TCTGTTAATT
Conservation ****:**..* *..      . **....**.* ...*.***** ****.***** **********

             ....|....| ....|....| ....|....| ....|....| ....|....| ....|....|
                125        135        145        155        165        175
3_SalI       CGTATTGGGG TAGACAACAT GAGTCAGAAT ACCCTGGAAG GAAGAAGAAA AATTTTAAAA
3_Thai       CGTATTGGGG TAGACAACAT GAGTCAGAAT ACCCTGGAAG GAAGAAGAAA AATTTTAAAA
Conservation ********** ********** ********** ********** ********** **********

             ....|....| ....|....| ....|....| ....|....| ....|....| ....|....|
                185        195        205        215        225        235
3_SalI       AAAATAGCTT GTCCCCAGGA GAGGAGGGAT GTAGGAATGA TTGCCCCCCC AGCGGTAGTG
3_Thai       AAAATAGCTT GTCCCCAGGA GAGGAGGGAT GTAGGAATGA TTGCCCCCCC AGCGGTAGTG
Conservation ********** ********** ********** ********** ********** **********

             ....|....| ....|....| ....|....| ....|....| ....|....| ....|....|
                245        255        265        275        285        295
3_SalI       ATGGAAACGA ATCAGGGTTA TTTAAGAAGA AAGCACTTAT ACATTTAAGA GATAGCAAAT
3_Thai       ATGGAAACGA ATCAGGGTTA TTTAAGAAGA AAGCACTTAT ACATTTAAGA GATAGCAAAT
Conservation ********** ********** ********** ********** ********** **********

             ....|....| ....|....| ....|....| ....|....| ....|....| ....|....|
                305        315        325        335        345        355
3_SalI       TTAGAAGGAA GCCATCGCGG TATGCGTATA TTAAAAGAAA CGGTGAGAAC AATTTGGATA
3_Thai       TTAGAAGGAA GCCATCGCGG TATGCGTATA TTAAAAGAAA CGGTGAGAAC AATTTGGATA
Conservation ********** ********** ********** ********** ********** **********

             ....|....| ....|....| ....|....| ....|....| ....|....| ....|....|
                365        375        385        395        405        415
3_SalI       GACTTCCGAG TAGCACTGAG AAGGAGGAAA ATAATGACCG TAATGGAGGT GCCAGTTCCT
3_Thai       GACTTCCGAG TAGCACTGAG AAGGAGGAAA ATAATGACCG TAATGGAGGT GCCAGTTCCT
Conservation ********** ********** ********** ********** ********** **********

             ....|....| ....|....| ....|....| ....|....| ....|....| ....|....|
                425        435        445        455        465        475
3_SalI       TTTTACAAAA TTTGAACGTT CCCAATGTAG ACGAGATTTT AGATTATTTC GAGATTGTAG
3_Thai       TTTTACAAAA TTTGAACGTT CCCAATGTAG ACGAGATTTT AGATTATTTC GAGATTGTAG
Conservation ********** ********** ********** ********** ********** **********

             ....|....| ....|....| ....|....| ....|....| ....|....| ....|....|
                485        495        505        515        525        535
3_SalI       GTGAACAGGG AGAGTTAGTC TTTTCATTAG AATCATACTA TGTGGAGATC TACACGTGCA
3_Thai       GTGAACAGGG AGAGTTAGTC TTTTCATTAG AATCATACTA TGTGGAGATC TACACGTGCA
Conservation ********** ********** ********** ********** ********** **********

             ....|....| ....|....| ....|....| ....|....| ....|....| ....|....|
                545        555        565        575        585        595
3_SalI       AAGAAATTGT GCGTTTTATA AACCGCCAAG CGTTAACCAA CTTTAGCGGG AACTATGGGG
3_Thai       AAGAAATTGT GCGTTTTATA AACCGCCAAG CGTTAACCAA CTTTAGCGGG AACTATGGGG
Conservation ********** ********** ********** ********** ********** **********
```

```
             ....|....| ....|....| ....|....| ....|....| ....|....| ....|....|
                605        615        625        635        645        655
3_SalI       ATGATGATGG TGAGGAGGAA GAAGAAGATG AGGATAGTGA CTACAATTCT GGGTTAAGAA
3_Thai       ATGATGATGG TGAGGAGGAA GAAGAAGATG AGGATAGTGA CTACAATTCT GGGTTAAGAA
Conservation ********** ********** ********** ********** ********** **********

             ....|....| ....|....| ....|....| ....|....| ....|....| ....|....|
                665        675        685        695        705        715
3_SalI       AAGATTCCAG GATACAAAAT CTGTATCATC ATATGAAACT CTCCATGATT TCTTGCATTT
3_Thai       AAGATTCCAG GATACAAAAT CTGTATCATC ATATGAAACT CTCCATGATT TCTTGCATTT
Conservation ********** ********** ********** ********** ********** **********

             ....|....| ....|....| ....|....| ....|....| ....|....| ....|....|
                725        735        745        755        765        775
3_SalI       CTGAAAAGGT GAAGCTACTA AACGAAATAA ATAGTTTGGA ATACCCCCAG GCAGAGGATA
3_Thai       CTGAAAAGGT GAAGCTACTA AACGAAATAA ATAGTTTGGA ATACCCCCAG GCAGAGGATA
Conservation ********** ********** ********** ********** ********** **********

             ....|....| ....|....| ....|....| ....|....| ....|....| ....|....|
                785        795        805        815        825        835
3_SalI       GTGAAGAGGA CTCAAGAGCA GAGAATTCTA CCACATACAA ATTACTCATA AATGAGTATG
3_Thai       GTGAAGAGGA CTCAAGAGCA GAGAATTCTA CCACATACAA ATTACTCATA AATGAGTATG
Conservation ********** ********** ********** ********** ********** **********

             ....|....| ....|....| ....|....| ....|....| ....|....| ....|....|
                845        855        865        875        885        895
3_SalI       TAGATTGCTT AAAGAAGTAT AGCGAAAATG TTAAAGGGAA GATAGCGGAT GCTAAGAAAA
3_Thai       TAGATTGCTT AAAGAAGTAT AGCGAAAATG TTAAAGGGAA GATAGCGGAT GCTAAGAAAA
Conservation ********** ********** ********** ********** ********** **********

             ....|....| ....|....| ....|....| ....|....| ....|....| ....|....|
                905        915        925        935        945        955
3_SalI       GCATCGAAAC AGAATATGGA AATAAATTTT GTCCTAATGA GTGCAATTCG AATGTGTATT
3_Thai       GCATCGAAAC AGAATATGGA AATAAATTTT GTCCTAATGA GTGCAATTCG AATGTGTATT
Conservation ********** ********** ********** ********** ********** **********

             ....|....| ....|....| ....|....| ....|....| ....|....| ....|....|
                965        975        985        995        1005       1015
3_SalI       TTGAAAGTGT ACATTTGTAC CTCTCATATG TCAATAATAA TGCAGACATT TTGTACAATT
3_Thai       TTGAAAGTGT ACATTTGTAC CTCTCATATG TCAATAATAA TGCAGACATT TTGTACAATT
Conservation ********** ********** ********** ********** ********** **********

             ....|....| ....|....| ....|....| ....|....| ....|....| ....|....|
                1025       1035       1045       1055       1065       1075
3_SalI       CTAACATTAG CGATGCGAAG GAATTTTTAA AATCTGGGAC AAGCATTGTT AGCATTATAG
3_Thai       CTAACATTAG CGATGCGAAG GAATTTTTAA AATCTGGGAC AAGCATTGTT AGCATTATAG
Conservation ********** ********** ********** ********** ********** **********

             ....|....| ....|....| ....|....| ....|....| ....|....| ....|....|
                1085       1095       1105       1115       1125       1135
3_SalI       AGAAAGAAAT AGGGTTAAAG CATGTCATAA GTTCTATAAA GTTCCTGCAA AGTGAAATAA
3_Thai       AGAAAGAAAT AGGGTTAAAG CATGTCATAA GTTCTATAAA GTTCCTGCAA AGTGAAATAA
Conservation ********** ********** ********** ********** ********** **********

             ....|....| ....|....| ....|....| ....|....| ....|....| ....|....|
                1145       1155       1165       1175       1185       1195
3_SalI       ATAACATCAT AAATAACTAC AATTATCACA TAAAGAATAT AAAAACTGCT ATCGATAATA
3_Thai       ATAACATCAT AAATAACTAC AATTATCACA TAAAGAATAT AAAAACTGCT ATCGATAATA
Conservation ********** ********** ********** ********** ********** **********
```

```
              ....|....| ....|....| ....|....| ....|....| ....|....| ....|....|
                   1205       1215       1225       1235       1245       1255
3_SalI        TAAAGGAGCA TGAAAAAAAT GATAACTCTA TCGTGTTTGA TAAAAAAAGC TTGGTGGAGA
3_Thai        TAAAGGAGCA TGAAAAAAAT GATAACTCTA TCGTGTTTGA TAAAAAAAGC TTGGTGGAGA
Conservation  ********** ********** ********** ********** ********** **********

              ....|....| ....|....| ....|....| ....|....| ....|....| ....|....|
                   1265       1275       1285       1295       1305       1315
3_SalI        AAAGTTTCGA CTTGGCAAAA AACATGTCCG TTTACAAATT TAACAATGAG ATGTTCAATA
3_Thai        AAAGTTTCGA CTTGGCAAAA AACATGTCCG TTTACAAATT TAACAATGAG ATGTTCAATA
Conservation  ********** ********** ********** ********** ********** **********

              ....|....| ....|....| ....|....| ....|....| ....|....| ....|....|
                   1325       1335       1345       1355       1365       1375
3_SalI        GGATGAAAGT ATTGTACGAT TTGAAGAGAA CTAAATTTAA AAAACTATTT ATTTTCTTAG
3_Thai        GAATGAAAGT ATTGTACGAT TTGAAGAGAA CTAAATTTAA AAAACTATTT ATTTTCTTAG
Conservation  *.******** ********** ********** ********** ********** **********

              ....|....| ....|....| ....|....| ....|....| ....|....| ....|....|
                   1385       1395       1405       1415       1425       1435
3_SalI        CAGAGGAGTT AGAGAATAAG CTTAACTCCT TTGCAGAGTC CGCGGAATTT CAGAAAACGT
3_Thai        CAGAGGAGTT AGAGAATAAG CTTAACTCCT TTGCAGAGTC CGCGGAATTT CAGAAAACGT
Conservation  ********** ********** ********** ********** ********** **********

              ....|....| ....|....| ....|....| ....|....| ....|....| ....|....|
                   1445       1455       1465       1475       1485       1495
3_SalI        ATGACTCGAT AATTAACGAC TGGAAATTTA TCTTGGCATA CGCGAAAGAT GTATATAATA
3_Thai        ATGACTCGAT AATTAACGAC TGGAAATTTA TCTTGGCATA CGCGAAAGAT GTATATAATA
Conservation  ********** ********** ********** ********** ********** **********

              ....|....| ....|....| ....|....| ....|....| ....|....| ....|....|
                   1505       1515       1525       1535       1545       1555
3_SalI        AAAATTTGAC CAAGATAAAA AATTACGAAG GGAATGAAAG TTTAGAAGTT ATCATAGTCA
3_Thai        AAAATTTGAC CAAGATAAAA AATTACGAAG GGAATGAAAG TTTAGAAGTT ATCATAGTCA
Conservation  ********** ********** ********** ********** ********** **********

              ....|....| ....|....| ....|....| ....|....| ....|....| ....|....|
                   1565       1575       1585       1595       1605       1615
3_SalI        GAAATAAGGT GAAGGAAAAG CTAGCAACGT TGGAAGGTCT GGTAGACAAG TTAGAGGACC
3_Thai        GAAATAAGGT GAAGGAAAAG CTAGCAACGT TGGAAGGTCT GGTAGACAAG TTAGAGGACC
Conservation  ********** ********** ********** ********** ********** **********

              ....|....| ....|....| ....|....| ....|....| ....|....| ....|....|
                   1625       1635       1645       1655       1665       1675
3_SalI        TATTTAATAT AATCAAATCG AAGTATGTAA TTATCATGTC AGCGAAATCG CTCATTGGGG
3_Thai        TATTTAATAT CATCAAATCG AAGTATGTAA TTATCATGTC AGCGAAATCG CTCATTGGGG
Conservation  ********** .********* ********** ********** ********** **********

              ....|....| ....|....| ....|....| ....|....| ....|....| ....|....|
                   1685       1695       1705       1715       1725       1735
3_SalI        AACTGAAGGG GGAATTCAAA ACGGGAGAAA ATGTCGAGCA GAAATTTGAT GACCTCATAA
3_Thai        AACTGAAGGG GGAATTCAAA ACTGGAGAAA ATGTCGAGCA GAAATTTGAT GACCTCATAA
Conservation  ********** ********** ** ******* ********** ********** **********

              ....|....| ....|....| ....|....| ....|....| ....|....| ....|....|
                   1745       1755       1765       1775       1785       1795
3_SalI        GATTAATGGA AACGATAAGC AGCAAAATTA ACACGATGAA TGAAGGTATC GATACTGTAA
3_Thai        GATTAATGGA AACGATAAGC AGCAAAATTA ACACGATGAA TGAAGGTATC GATACTGTAA
Conservation  ********** ********** ********** ********** ********** **********
```

```
               ....|....| ....|....| ....|....| ....|....| ....|....| ....|....|
                   1805       1815       1825       1835       1845       1855
3_SalI         ATAAGACCTA CTCCAGCATT AAGTATGTAC AAATACAAAT CGAAAACTTA AGCACTTCTA
3_Thai         ATAAGACCTA CTCCAGCATT AAGTATGTAC AAATACAAAT CGAAAACTTA AGCACTTCTA
Conservation   ********** ********** ********** ********** ********** **********

               ....|....| ....|....| ....|....| ....|....| ....|....| ....|....|
                   1865       1875       1885       1895       1905       1915
3_SalI         TCGATGGGTA CATGAACGAA ATAGATGCCT TAAAAGCTAA GGGGTCAACG AATGATCATA
3_Thai         TCGATGGGTA CATGAACGAA ATAGATGCCT TAAAAGCTAA GGGGTCAACG AATGATCATA
Conservation   ********** ********** ********** ********** ********** **********

               ....|....| ....|....| ....|....| ....|....| ....|....| ....|....|
                   1925       1935       1945       1955       1965       1975
3_SalI         TTAGAGAAGA AATGGTGTCC AAAATGTTGT TCATTACGGA AAATATCAAC AATTTGAAAA
3_Thai         TTAGAGAAGA AATGGTGTCC AAAATGTTGT TCATTACGGA AAATATCAAC AATTTGAAAA
Conservation   ********** ********** ********** ********** ********** **********

               ....|....| ....|....| ....|....| ....|....| ....|....| ....|....|
                   1985       1995       2005       2015       2025       2035
3_SalI         AAATTCTTTC CCTGGAGGAG AAGGCAAACG AAAATATTGC GCAAATTGAT GAATTAATTA
3_Thai         AAATTTTTTC CCTGGAGGAG AAGGCAAACG AAAATATTGC GCAAATTGAT GAATTAATTA
Conservation   ***** **** ********** ********** ********** ********** **********

               ....|....| ....|....| ....|....| ....|....| ....|....| ....|....|
                   2045       2055       2065       2075       2085       2095
3_SalI         TGGGAACATC GTTAGATATG AACGAAATTA TAGATAAAAA AAGTGACGCG TATAATAAAA
3_Thai         TGGGAACATC GTTAGATATG AACGAAATTA TAGATAAAAA AAGTGACGCG TATAATAAAA
Conservation   ********** ********** ********** ********** ********** **********

               ....|....| ....|....| ....|....| ....|....| ....|....| ....|....|
                   2105       2115       2125       2135       2145       2155
3_SalI         TAAAAATGAT TATTAAAGAT GTTTATGAAG GCAATTTGAA GGACTTTCTA GACGAACTGT
3_Thai         TAAAAATGAT TATTAAAGAT GTTTATGAAG GCAATTTGAA GGACTTTCTA GACGAACTGT
Conservation   ********** ********** ********** ********** ********** **********

               ....|....| ....|....| ....|....| ....|....| ....|....| ....|....|
                   2165       2175       2185       2195       2205       2215
3_SalI         CTCTTAATGT AGACAAGAAT AGAAATTCCA TCAACCAAAG TAGTTCTACG AACGAACTGG
3_Thai         CTCTTAATGT AGACAAGAAT AGAAATTCCA TCAACCAAAG TAGTTCTACG AACGAACTGG
Conservation   ********** ********** ********** ********** ********** **********

               ....|....| ....|....| ....|....| ....|....| ....|....| ....|....|
                   2225       2235       2245       2255       2265       2275
3_SalI         ATGGCGTGTT AAATAATATA AAAGGTGATT ATGAGAAAAT GAAATCTATC AAATGTGATA
3_Thai         ATGGCGTGTT AAATAATATA AAAGGTGATT ATGAGAAAAT GAAATCTATC AAATGTGATA
Conservation   ********** ********** ********** ********** ********** **********

               ....|....| ....|....| ....|....| ....|....| ....|....| ....|....|
                   2285       2295       2305       2315       2325       2335
3_SalI         ATATATCAGA ATCTTTGCAG AACATAACGA ATGAACTCAA AATTATTTCA GACGCCAAAG
3_Thai         ATATATCAGA ATCTTTGCAG AACATAACGA ATGAACTCAA AATTATTTCA GACGCCAAAG
Conservation   ********** ********** ********** ********** ********** **********

               ....|....| ....|....| ....|....| ....|....| ....|....| ....|....|
                   2345       2355       2365       2375       2385       2395
3_SalI         AGGAAATTTT AAAAATCCAA TTCGGAGATA TCAATGAAAA ATTAACAAAC TCGTTAGAGC
3_Thai         AGGAAATTTT AAAAATCCAA TTCGGAGATA TCAATGAAAA ATTAACAAAC TCGTTAGAGC
Conservation   ********** ********** ********** ********** ********** **********
```

```
             ....|....| ....|....| ....|....| ....|....| ....|....| ....|....|
                  2405       2415       2425       2435       2445       2455
3_SalI       AACTTCAGAG TGTCTACGAC AAATTACAAG TTAGCACAAC AGAGTATACA GCGGGAAAGG
3_Thai       AACTTCAGAG TGTCTACGAC AAATTACAAG TTAGCACAAC AGAGTATACA GCGGGAAAGG
Conservation ********** ********** ********** ********** ********** **********

             ....|....| ....|....| ....|....| ....|....| ....|....| ....|....|
                  2465       2475       2485       2495       2505       2515
3_SalI       GGAAGATTGA GGAATACAAA ACGGTTATAC TAAAGGGGGA AAAAGAATTT TTTGACAAAG
3_Thai       GGAAGATTGA GGAATACAAA ACGGTTATAC TAAAGGGGGA AAAAGAATTT TTTGACAAAG
Conservation ********** ********** ********** ********** ********** **********

             ....|....| ....|....| ....|....| ....|....| ....|....| ....|....|
                  2525       2535       2545       2555       2565       2575
3_SalI       CGTATGATAA GGATGAAGAT ATGTTAGAAG TGAAAGATGC TTACGCAAAT TTTCTCAAAC
3_Thai       CGTATGATAA GGATGAAGAT ATGTTAGAAG TGAAAGATGC TTACGCAAAT TTTCTCAAAC
Conservation ********** ********** ********** ********** ********** **********

             ....|....| ....|....| ....|....| ....|....| ....|....| ....|....|
                  2585       2595       2605       2615       2625       2635
3_SalI       ATAAGGATAA TCTCCTCAAG AACAGAAGTA AAGTGTTTGA AGAATTCCAT ATGGCGAAAG
3_Thai       ATAAGGATAA TCTCCTCAAG AACAGAAGTA AAGTGTTTGA AGAATTCCAT ATGGCGAAAG
Conservation ********** ********** ********** ********** ********** **********

             ....|....| ....|....| ....|....| ....|....| ....|....| ....|....|
                  2645       2655       2665       2675       2685       2695
3_SalI       AGGCCCTAAA AACGGCTAGG ATTTACCTCC TATCTTATGT AGATGTGCCG GAAAAATATA
3_Thai       AGGCCCTAAA AACGGCTAGG ATTTACCTCC TATCTTATGT AGATGTGCCG GAAAAATATA
Conservation ********** ********** ********** ********** ********** **********

             ....|....| ....|....| ....|....| ....|....| ....|....| ....|....|
                  2705       2715       2725       2735       2745       2755
3_SalI       ACATTATCAA GGGGGAAACA AATCAAAAAT ATAAAAAGAT AATACAAGAA ATTAATGACA
3_Thai       ACATTATCAA GGGGGAAACA AATCAAAAAT ATAAAAAGAT AATACAAGAA ATTAATGACA
Conservation ********** ********** ********** ********** ********** **********

             ....|....| ....|....| ....|....| ....|....| ....|....| ....|....|
                  2765       2775       2785       2795       2805       2815
3_SalI       AAGATGTCGA TATCAAATTG GAAGAATACG AAGCAGACTT CAACAGGGAC AACCAATTAT
3_Thai       AAGATGTCGA TATCAAATTG GAAGAATACG AAGCAGACTT CAACAGGGAC AACCAATTAT
Conservation ********** ********** ********** ********** ********** **********

             ....|....| ....|....| ....|....| ....|....| ....|....| ....|....|
                  2825       2835       2845       2855       2865       2875
3_SalI       CCGATAGCAT CATAAAGGAA GTGGACCAAT CAAATAAAAA TTTGTCTAAC CTGAAAATTT
3_Thai       CCGATAGCAT CATAAAGGAA GTGGACCAAT CAAATAAAAA TTTGTCTAAC CTGAAAATTT
Conservation ********** ********** ********** ********** ********** **********

             ....|....| ....|....| ....|....| ....|....| ....|....| ....|....|
                  2885       2895       2905       2915       2925       2935
3_SalI       TAAACAAGTC TATTAAAGAT TGCTCCACGG ATAGCAACAT TATCGAAGAA TTAATTAGCA
3_Thai       TAAACAAGTC TATTAAAGAT TGCTCCACGG ATAGCAACAT TATCGAAGAA TTAATTAGCA
Conservation ********** ********** ********** ********** ********** **********

             ....|....| ....|....| ....|....| ....|....| ....|....| ....|....|
                  2945       2955       2965       2975       2985       2995
3_SalI       AAAATAATTC TCTGAAGGAA AAAATTATTT CCGAAAGGAA AGAAATAGAA GAAGATAATT
3_Thai       AAAATAATTC TCTGAAGGAA AAAATTATTT CCGAAAGGAA AGAAATAGAA GAAGATAATT
Conservation ********** ********** ********** ********** ********** **********
```

```
             ....|....| ....|....| ....|....| ....|....| ....|....| ....|....|
                  3005       3015       3025       3035       3045       3055
3_SalI       TCATGGAACA ACATGTGAAG ATAAATTTAA AAATCAAATT GAACGATGCT CGTATTACTC
3_Thai       TCATGGAACA ACATGTGAAG ATAAATTTAA AAATCAAATT GAACGATGCT CGTATTACTC
Conservation ********** ********** ********** ********** ********** **********

             ....|....| ....|....| ....|....| ....|....| ....|....| ....|....|
                  3065       3075       3085       3095       3105       3115
3_SalI       TTGAAAACAA ATTGTATGAC AACAATTTGA CGAACTTGAA GACACAAATT AAGAATATTC
3_Thai       TTGAAAACAA ATTGTATGAC AACAATTTGA CGAACTTGAA GACACAAATT AAGAATATTC
Conservation ********** ********** ********** ********** ********** **********

             ....|....| ....|....| ....|....| ....|....| ....|....| ....|....|
                  3125       3135       3145       3155       3165       3175
3_SalI       TCGATTTTCA CACAAGTTCG AAGGAAAAAT ATAAAGATTT GAACGAAACA GACCTGAAGA
3_Thai       TCGATTTTCA CACAAGTTCG AAGGAAAAAT ATAAAGATTT GAACGAAACA GACCTGAAGA
Conservation ********** ********** ********** ********** ********** **********

             ....|....| ....|....| ....|....| ....|....| ....|....| ....|....|
                  3185       3195       3205       3215       3225       3235
3_SalI       AAATAAAAGA AAATGAAGAG TGGAAAAGCG CTAAGGAATT GATATACGCT CTTAATGTCG
3_Thai       AAATAAAAGA AAATGAAGAG TGGAAAAGCG CTAAGGAATT GATATACGCT CTTAATGTCG
Conservation ********** ********** ********** ********** ********** **********

             ....|....| ....|....| ....|....| ....|....| ....|....| ....|....|
                  3245       3255       3265       3275       3285       3295
3_SalI       AATACGAAAT ATTGAAGAAG CAAGCGGATG GTCTGATCAG CAGCAAAAAT AGTGAAATTA
3_Thai       AATACGAAAT ATTGAAGAAG CAAGCGGATG GTCTGATCAG CAGCAAAAAT AGTGAAATTA
Conservation ********** ********** ********** ********** ********** **********

             ....|....| ....|....| ....|....| ....|....| ....|....| ....|....|
                  3305       3315       3325       3335       3345       3355
3_SalI       TAAAATGGAT AGGCAACAGA ATAGTGGAAA AGAATAAAGA AATAAATGAA AAAGTGGATG
3_Thai       TAAAATGGAT AGGCAACAGA ATAGTGGAAA AGAATAAAGA AATAAATGAA AAAGTGGATG
Conservation ********** ********** ********** ********** ********** **********

             ....|....| ....|....| ....|....| ....|....| ....|....| ....|....|
                  3365       3375       3385       3395       3405       3415
3_SalI       AACATGTAAA TTCGTTCGAT GAAATTGTTG TAAAATCTAA GTCGCCCAGT TTTGTCAGAG
3_Thai       AACATGTAAA TTCGTTCGAT GAAATTGTTG TAAAATCTAA GTCGCCCAGT TTTGTCAGAG
Conservation ********** ********** ********** ********** ********** **********

             ....|....| ....|....| ....|....| ....|....| ....|....| ....|....|
                  3425       3435       3445       3455       3465       3475
3_SalI       ATGTTAATCA CTACCAAAAT GGTGAGAATA AAGAAAAGGC AAGACAGTTT AATGCAGAAG
3_Thai       ATGTTAATCA CTACCAAAAT GGTGAGAATA AAGAAAAGGC AAGACAGTTT AATGCAGAAG
Conservation ********** ********** ********** ********** ********** **********

             ....|....| ....|....| ....|....| ....|....| ....|....| ....|....|
                  3485       3495       3505       3515       3525       3535
3_SalI       TGGAAGCTCT CGTTCGGAAG ATCGAAACAG AAAAGGAAAA GTTTAACAAA ATTAAAACAA
3_Thai       TGGAAGCTCT CGTTCGGAAG ATCGAAACAG AAAAGGAAAA GTTTAACAAA ATTAAAACAA
Conservation ********** ********** ********** ********** ********** **********

             ....|....| ....|....| ....|....| ....|....| ....|....| ....|....|
                  3545       3555       3565       3575       3585       3595
3_SalI       ATTCTAATGA ATACTTGGTA CAAGCAAATC GAAAGAAAAG CGAAAATGTA GATTTCAAGG
3_Thai       ATTCTAATGA ATACTTGGTA CAAGCAAATC GAAAGAAAAG CGAAAATGTA GATTTCAAGG
Conservation ********** ********** ********** ********** ********** **********
```

```
             ....|....| ....|....| ....|....| ....|....| ....|....| ....|....|
                  3605       3615       3625       3635       3645       3655
3_SalI       CCAAGGAAAA GGCCATGAAA GAAGTGTACG AACAAATTAG AAGCGCATCA GAAGAACTGA
3_Thai       CCAAGGAAAA GGCCATGAAA GAAGTGTACG AACAAATTAG AAGCGCATCA GAAGAACTGA
Conservation ********** ********** ********** ********** ********** **********

             ....|....| ....|....| ....|....| ....|....| ....|....| ....|....|
                  3665       3675       3685       3695       3705       3715
3_SalI       ATAACTCACT GAAGGAAATT CCCACACTGA ATGATTTACA GAAGATAGAA TTAAAATTTG
3_Thai       ATAACTCACT GAAGGAAATT CCCACACTGA ATGATTTACA GAAGATAGAA TTAAAATTTG
Conservation ********** ********** ********** ********** ********** **********

             ....|....| ....|....| ....|....| ....|....| ....|....| ....|....|
                  3725       3735       3745       3755       3765       3775
3_SalI       AAAAAAACGA AATTCACGAT ATGGTTAATC AGATTACCAC TGAGCAGAAA AAGTGTGAGC
3_Thai       AAAAAAACGA AATTCACGAT ATGGTTAATC AGATTACCAC TGAGCAGAAA AAGTGTGAGC
Conservation ********** ********** ********** ********** ********** **********

             ....|....| ....|....| ....|....| ....|....| ....|....| ....|....|
                  3785       3795       3805       3815       3825       3835
3_SalI       AGGAAATGGC AGAAATCAAT TCTTGTAAAC ATGATATAGA TGAAATGAAG AGGGCAAACT
3_Thai       AGGAAATGGC AGAAATCAAT TCTTGTAAAC ATGATATAGA TGAAATGAAG AGGGCAAACT
Conservation ********** ********** ********** ********** ********** **********

             ....|....| ....|....| ....|....| ....|....| ....|....| ....|....|
                  3845       3855       3865       3875       3885       3895
3_SalI       CGGGAGAAGA GACTCCCGAT TCCCCTTTAT TTGTATATAC CGAATTTTAC GAAAAAGCGG
3_Thai       CGGGAGAAGA GACTCCCGAT TCCCCTTTAT TTGTATATAC CGAATTTTAC GAAAAAGCGG
Conservation ********** ********** ********** ********** ********** **********

             ....|....| ....|....| ....|....| ....|....| ....|....| ....|....|
                  3905       3915       3925       3935       3945       3955
3_SalI       TAAGCAGTCA TGGGACTATC AAAGCCCGTT TTAACGAGGC TACCGGGTTA AATGGGAAGT
3_Thai       TAAGCAGTCA TGGGACTATC AAAGCCCGTT TTAACGAGGC TACCGGGCTA AATGGGAAGT
Conservation ********** ********** ********** ********** ******* ** **********

             ....|....| ....|....| ....|....| ....|....| ....|....| ....|....|
                  3965       3975       3985       3995       4005       4015
3_SalI       GTGAGAGTAG CGAAAATATT AACGAAATAA AGGGAATGAA AAAGCAGATT GATTGTTATT
3_Thai       GTGAGAGTAG CGAAAATATT AACGAAATAA AGGGAATGAA AAAGCAGATT GATGGTTATT
Conservation ********** ********** ********** ********** ********** *** ******

             ....|....| ....|....| ....|....| ....|....| ....|....| ....|....|
                  4025       4035       4045       4055       4065       4075
3_SalI       TGAGAGAAAT TTTAACAAAT TATGGCACCT TGCATGAAGC GTTGGGCGAT ATTAAAAATA
3_Thai       TGAGAGAAAT TTTAACAAAT TATGGCACCT TGCATGAAGC GTTGGGCGAT ATTAAAAATA
Conservation ********** ********** ********** ********** ********** **********

             ....|....| ....|....| ....|....| ....|....| ....|....| ....|....|
                  4085       4095       4105       4115       4125       4135
3_SalI       TGAAGGAAAT GCTGATGGAT GTGGATGTTC AAGAAATTGT GCGTCTGGTG GAGAAGGATG
3_Thai       TGAAGGAAAT GCTGATGGAT GTGGATGTTC AAGAAATTGT GCGTCTGGTG GAGAAGGATG
Conservation ********** ********** ********** ********** ********** **********

             ....|....| ....|....| ....|....| ....|....| ....|....| ....|....|
                  4145       4155       4165       4175       4185       4195
3_SalI       AGACAGAAGC GCAGAAATAT GCCACGTTGG TGAAAAGTGA GCAGGCCAAA TCAGATGCCC
3_Thai       AGACAGAAGC GCAGAAATAT GCCACGTTGG TGAAAAGTGA GCAGGCCAAA TCAGATGCCC
Conservation ********** ********** ********** ********** ********** **********
```

```
             ....|....| ....|....| ....|....| ....|....| ....|....| ....|....|
                  4205       4215       4225       4235       4245       4255
3_SalI       TAATTAAAGA ATTAGATAAA CATTTCACAG AAGCAACGAG GCTAAAAACG GAAATGAATA
3_Thai       TAATTAAAGA ATTAGATAAA CATTTCACAG AAGCAACGAG GCTAAAAACG GAAATGAATA
Conservation ********** ********** ********** ********** ********** **********

             ....|....| ....|....| ....|....| ....|....| ....|....| ....|....|
                  4265       4275       4285       4295       4305       4315
3_SalI       AAAATTTATC CATCCAAAGA ATCGATGAAA TTGTCAATGA AATTATGAAA TATAAGGACG
3_Thai       AAAATTTATC CATCCAAAGA ATCGATGAAA TTGTCAATGA AATTATGAAA TATAAGGACG
Conservation ********** ********** ********** ********** ********** **********

             ....|....| ....|....| ....|....| ....|....| ....|....| ....|....|
                  4325       4335       4345       4355       4365       4375
3_SalI       AAATAGCACA CCGGAAAGAA GAAATGAACA ATTATTTGAA GAACTCCAAG GAGTATAAGG
3_Thai       AAATAGCACA CCGGAAAGAA GAAATGAACA ATTATTTGAA GAACTCCAAG GAGTATAAGG
Conservation ********** ********** ********** ********** ********** **********

             ....|....| ....|....| ....|....| ....|....| ....|....| ....|....|
                  4385       4395       4405       4415       4425       4435
3_SalI       ATAAGGCTTT TCTGCATTAC CGTAACGCAG ACAGGAGAAA GAAAAAGCTA CAACATTTGA
3_Thai       ATAAGGCTTT TCTGCATTAC CGTAACGCAG ACAGGAGAAA GAAAAAGCTA CAACATTTGA
Conservation ********** ********** ********** ********** ********** **********

             ....|....| ....|....| ....|....| ....|....| ....|....| ....|....|
                  4445       4455       4465       4475       4485       4495
3_SalI       AGGAAAAGGG GGAAGAATCT GAACCGCAGA TTGATATGGA TAAAGTAAGT TTGAGTGTAA
3_Thai       AGGAAAAGGG GGAAGAATCT GAACCGCAGA TTGATATGGA TAAAGTAAGT TTGAGTGTAA
Conservation ********** ********** ********** ********** ********** **********

             ....|....| ....|....| ....|....| ....|....| ....|....| ....|....|
                  4505       4515       4525       4535       4545       4555
3_SalI       ACCAATGTAT GACTCAATCA AATGCCGCTT CAGCGAGTGA AGAAGAAATA AAAAAGCACG
3_Thai       ACCAATGTAT GACTCAATCA AATGCCGCTT CAGCGAGTGA AGAAGAAATA AAAAAGCACG
Conservation ********** ********** ********** ********** ********** **********

             ....|....| ....|....| ....|....| ....|....| ....|....| ....|....|
                  4565       4575       4585       4595       4605       4615
3_SalI       GTAATAGCTA CGCAGAATAT GAAGGAAAAA TGAATACTCT CTTAAATGAC ATTTCCGTTT
3_Thai       GTAATAGCTA CGCAGAATAT GAAGGAAAAA TGAATACTCT CTTAAATGAC ATTTCCGTTT
Conservation ********** ********** ********** ********** ********** **********

             ....|....| ....|....| ....|....| ....|....| ....|....| ....|....|
                  4625       4635       4645       4655       4665       4675
3_SalI       TGCAAATAAG GACCAAGTAT GCAAAGGGGA AGGACCAGGC CACCAGCATC ATGAGAGAAA
3_Thai       TGCAAATAAG GACCAAGTAT GCAAAGGGGA AGGACCAGGC CACCAGCATC ATGAGAGAAA
Conservation ********** ********** ********** ********** ********** **********

             ....|....| ....|....| ....|....| ....|....| ....|....| ....|....|
                  4685       4695       4705       4715       4725       4735
3_SalI       TCGAACAATT GCATGCAGGC ATCCAAGAAA AACTTAAAGA ATCTGAAAGA AAATTAGAAA
3_Thai       TCGAACAATT GCATGCAGGC ATCCNAGAAA AACTTAAAGA ATCTGAAAGA AAATTAGAAA
Conservation ********** ********** **** ***** ********** ********** **********

             ....|....| ....|....| ....|....| ....|....| ....|....| ....|....|
                  4745       4755       4765       4775       4785       4795
3_SalI       TGTGGAAGAG GAAATCCCAA ATGAATGAAA GAACAGATAT ATTGAATAAT GACAAATCGA
3_Thai       TGTGGAAGAG GAAATCCCAA ATGAATGAAA GAACAGATAT ATTGAATAAT GACAAATCGA
Conservation ********** ********** ********** ********** ********** **********
```

```
              ....|....| ....|....| ....|....| ....|....| ....|....| ....|....|
                 4805       4815       4825       4835       4845       4855
3_SalI        AGACGGCATA CATAAACGTG AAATTAAATT TAGAATTAGT CGAGTCTAAT TTAGGCCAGA
3_Thai        AGACGGCATA CATAAACGTG AAATTAAATT TAGAATTAGT CGAGTCTAAT TTAGGCCAGA
Conservation ********** ********** ********** ********** ********** **********

              ....|....| ....|....| ....|....| ....|....| ....|....| ....|....|
                 4865       4875       4885       4895       4905       4915
3_SalI        TAAAAAGTTT GAAAGAAAGT GTGAATAATA TTCTATCCAA ATCGACTAAT TTGAATAGTT
3_Thai        TAAAAAGTTT GAAAGAAAGT GTGAATAATA TTCTATCCAA ATCGACTAAT TTGAATAGTT
Conservation ********** ********** ********** ********** ********** **********

              ....|....| ....|....| ....|....| ....|....| ....|....| ....|....|
                 4925       4935       4945       4955       4965       4975
3_SalI        CCATACTAGG GGCGTATAAA ATTGAAAGTG ATAGCTCCTT AGATGTGCTG AAAAAGGAAG
3_Thai        CCATACTAGG GGCGTATAAA ATTGAAAGTG ATAGCTCCTT AGATGTGCTG AAAAAGGAAG
Conservation ********** ********** ********** ********** ********** **********

              ....|....| ....|....| ....|....| ....|....| ....|....| ....|....|
                 4985       4995       5005       5015       5025       5035
3_SalI        AAATGCATTA CATGCAGTAT TTGAAAAATA TTGAAAATGA GAAAAAGCTC ATGACGGAAG
3_Thai        AAATGCATTA CATGCAGTAT TTGAAAAATA TTGAAAATGA GAAAAAGCTC ATGACGGAAG
Conservation ********** ********** ********** ********** ********** **********

              ....|....| ....|....| ....|....| ....|....| ....|....| ....|....|
                 5045       5055       5065       5075       5085       5095
3_SalI        AGAAGAGCAA CATGGATGTG ATACACGGAA ATGTCCTTAA AATAGAAAAT GAGATGGAGA
3_Thai        AGAAGAGCAA CATGGATGTG ATACACGGAA ATGTCCTTAA AATAGAAAAT GAGATGGAGA
Conservation ********** ********** ********** ********** ********** **********

              ....|....| ....|....| ....|....| ....|....| ....|....| ....|....|
                 5105       5115       5125       5135       5145       5155
3_SalI        AATGTAAAAA AAATTACGAA GAGAGCATTT TGAAAAAAGC CAAAGAAACG GCTGATAAGA
3_Thai        AATGTAAAAA AAATTACGAA GAGAGCATTT TGAAAAAAGC CAAAGAAACG GCTGATAAGA
Conservation ********** ********** ********** ********** ********** **********

              ....|....| ....|....| ....|....| ....|....| ....|....| ....|....|
                 5165       5175       5185       5195       5205       5215
3_SalI        AGAAACAACT TATTGAGGCA ACTAGGGAGT CGCTAAACTC CCTGGAGCCA TACTTTACCG
3_Thai        AGAAACAACT TATTGAGGCA ACTAGGGAGT CGCTAAACTC CCTGGAGCCA TACTTTACCG
Conservation ********** ********** ********** ********** ********** **********

              ....|....| ....|....| ....|....| ....|....| ....|....| ....|....|
                 5225       5235       5245       5255       5265       5275
3_SalI        AGATGTTTAA TGAATCATAT TTGAAAGAAT ACAACATTAA GGAAAAATTT GTCGGTTATC
3_Thai        AGATGTTTAA TGAATCATAT TTGAAAGAAT ACAACATTAA GGAAAAATTT GTCGGTTATC
Conservation ********** ********** ********** ********** ********** **********

              ....|....| ....|....| ....|....| ....|....| ....|....| ....|....|
                 5285       5295       5305       5315       5325       5335
3_SalI        AAAAGAGCAT GAAAGGGTTA TATGACGAAT TTGGTGAGTC CTACAAAGTA ATCGAAACGA
3_Thai        AAAAGAGCAT GAAAGGGTTA TATGACGAAT TTGGTGAGTC CTACAAAGTA ATCGAAACGA
Conservation ********** ********** ********** ********** ********** **********

              ....|....| ....|....| ....|....| ....|....| ....|....| ....|....|
                 5345       5355       5365       5375       5385       5395
3_SalI        ACGCTGCAAA GGTTGCAGAG GGCACGGTAA AGTATGATGA AGCGAGAAGA CTGAGGGAGG
3_Thai        ACGCTGCAAA GGTTGCAGAG GGCACGGTAA AGTATGATGA AGCGAGAAGA CTGAGGGAGG
Conservation ********** ********** ********** ********** ********** **********
```

```
             ....|....| ....|....| ....|....| ....|....| ....|....| ....|....|
                  5405       5415       5425       5435       5445       5455
3_SalI       AAGCTCAAAA GGCAGAAATA AATATTAACA ATAAGGAAGA AGCTGCAAAA ACAAATTTGA
3_Thai       AAGCTCAAAA GGCAGAAATA AATATTAACA ATAAGGAAGA AGCTGCAAAA ACAAATTTGA
Conservation ********** ********** ********** ********** ********** **********

             ....|....| ....|....| ....|....| ....|....| ....|....| ....|....|
                  5465       5475       5485       5495       5505       5515
3_SalI       ACAAAATAAA GCAGCATGAA TTTATGAACT TTCTATTTCA TACAAAGGAA CATGTGGATA
3_Thai       ACAAAATAAA GCAGCATGAA TTTATGAACT TTCTATTTCA TACAAAGGAA CATGTGGATA
Conservation ********** ********** ********** ********** ********** **********

             ....|....| ....|....| ....|....| ....|....| ....|....| ....|....|
                  5525       5535       5545       5555       5565       5575
3_SalI       ATATTAAGAA AGCGTGTGAA CAAGAAAATA CCAAAATGGA CTAAGGACAT ACCCATATTA
3_Thai       ATATTAAGAA AGCGTGTGAA CAAGAAAATA CCAAAATGGA CGAAGGACAT ACCCATATTA
Conservation ********** ********** ********** ********** * ******** **********

             ....|....| ....|....| ....|....| ....|....| ....|....| ....|....|
                  5585       5595       5605       5615       5625       5635
3_SalI       AGCAAATCAT TATAAACATT CGAAAGTTAA CAGACCAGAA GAGCACTTTC GAAACGTTAA
3_Thai       AGCAAATCAT TATAAACATT CGAAAGTTAA CAGACCAGAA GAGCACTTTC GAAACGTTAA
Conservation ********** ********** ********** ********** ********** **********

             ....|....| ....|....| ....|....| ....|....| ....|....| ....|....|
                  5645       5655       5665       5675       5685       5695
3_SalI       AAATGGCAAA GGAGAAAAAT AACGAAATAA AAAAGAACGC ACAGCAGTGT AATAAAAATG
3_Thai       AAATGGCAAA GGAGAAAAAT AACGAAATAA AAAAGAACGC ACAGCAGTGT AATAAAAATG
Conservation ********** ********** ********** ********** ********** **********

             ....|....| ....|....| ....|....| ....|....| ....|....| ....|....|
                  5705       5715       5725       5735       5745       5755
3_SalI       AGGTACATAG CGCCTTTGGG AAGATGATAA AAGCGTCTAA TTTTATGGGG ATAAAAATAT
3_Thai       AGGCACATAG CGCCTTTGGG AAGATGATAA AAGCGTCTAA TTTTATGGGG ATAAAAATAT
Conservation *** ****** ********** ********** ********** ********** **********

             ....|....| ....|....| ....|....| ....|....| ....|....| ....|....|
                  5765       5775       5785       5795       5805       5815
3_SalI       TAACAAACTT AGGATCCGAG TTGAGCCCCG AAATGCACCT AGAAACTACT TCACGAACTA
3_Thai       TAACAAACTT AGGATCCGAG TTGAGCCCCG AAATGCACCT AGAAACTACT TCACGAACTA
Conservation ********** ********** ********** ********** ********** **********

             ....|....| ....|....| ....|....| ....|....| ....|....| ....|....|
                  5825       5835       5845       5855       5865       5875
3_SalI       ATTCAGAGTT AAAATTTGAA TCGGAGGTGG AAATAAAGTC GGAAAGTGAT GCCAAATTAG
3_Thai       ATTCAGAGTT AAAATTTGAA TCGGAGGTGG AAATAAAGTC GGAAAGTGAT GCCAAATTAG
Conservation ********** ********** ********** ********** ********** **********

             ....|....| ....|....| ....|....| ....|....| ....|....| ....|....|
                  5885       5895       5905       5915       5925       5935
3_SalI       ATGCCTACAA AAATCTACAA GTGGCCTATG GATATATTCA AAAAATATTT AAAGATTCGG
3_Thai       ATGCCTACAA AAATCTACAA GTGGCCTATG GATATATTCA AAAAATATTT AAAGATTCGG
Conservation ********** ********** ********** ********** ********** **********

             ....|....| ....|....| ....|....| ....|....| ....|....| ....|....|
                  5945       5955       5965       5975       5985       5995
3_SalI       AGGAGACAGA GAGGAAACAA GAAGAAATCG AAGGGTGGAT AAGGAAAGGG AACAATAAAT
3_Thai       AGGAGACAGA GAGGAAACAA GAAGAAATCG AAGGGTGGAT AAGGAAAGGG AACAATAAAT
Conservation ********** ********** ********** ********** ********** **********
```

```
                ....|....| ....|....| ....|....| ....|....| ....|....| ....|....|
                    6005       6015       6025       6035       6045       6055
3_SalI          GTCACGATAT TAAATCATCT AATGAATTAA AAACTAAATT GAAGTACACG AAGTATAAGG
3_Thai          GTCACGATAT TAAATCATCT AATGAATTAA AAACTAAATT GAAGTACACG AAGTATAAGG
Conservation    ********** ********** ********** ********** ********** **********

                ....|....| ....|....| ....|....| ....|....| ....|....| ....|....|
                    6065       6075       6085       6095       6105       6115
3_SalI          GAAACATTAC TTTAACTAAA ATTAATGACG CTTTGACCAA GTCTGCCCAA TTGAAAGCGA
3_Thai          GAAACATTAC TTTAACTAAA ATTAATGACG CTTTGACCAA GTCTGCCCAA TTGAAAGCGA
Conservation    ********** ********** ********** ********** ********** **********

                ....|....| ....|....| ....|....| ....|....| ....|....| ....|....|
                    6125       6135       6145       6155       6165       6175
3_SalI          TGACTTGCAG TATTGTGAAC GATAGCAAAA TTATGGAGAG CTCAGAATAT ACAAAATTGA
3_Thai          TGACTTGCAG TATTGTGAAC GATAGCAAAA TTATGGAGAG CTCAGAATAT ACAAAATTGA
Conservation    ********** ********** ********** ********** ********** **********

                ....|....| ....|....| ....|....| ....|....| ....|....| ....|....|
                    6185       6195       6205       6215       6225       6235
3_SalI          AGGAGATGAG TGATACGTAC AATCGGAAGA AGAAGGAAGA AGTATCCGAG ACGGAGGTCG
3_Thai          AGGAGATGAG TGATACGTAC AATCGGAAGA AGAAGGAAGA AGTATCCGAG ACGGAGGTCG
Conservation    ********** ********** ********** ********** ********** **********

                ....|....| ....|....| ....|....| ....|....| ....|....| ....|....|
                    6245       6255       6265       6275       6285       6295
3_SalI          ACGGAATAAA TGAAGAATTC CAAGGGATTA TGAAAAATAT AGAAAGTTTA GAAAAGCAAT
3_Thai          ACGGAATAAA TGAAGAATTC CAAGGGATTA TGAAAAATAT AGAAAGTTTA GAAAAGCAAT
Conservation    ********** ********** ********** ********** ********** **********

                ....|....| ....|....| ....|....| ....|....| ....|....| ....|....|
                    6305       6315       6325       6335       6345       6355
3_SalI          TTGAAGCAGT ACAGGAACAA GAATCACCCA GCGAAATGAT TATCGTGGAA AACTCCCTAG
3_Thai          TTGAAGCAGT ACAGGAACAA GAATCACCCA GCGAAATGAT TATCGTGGAA AACTCCCTAG
Conservation    ********** ********** ********** ********** ********** **********

                ....|....| ....|....| ....|....| ....|....| ....|....| ....|....|
                    6365       6375       6385       6395       6405       6415
3_SalI          TTACAGAAAT TAATAAAAAA ATAGACAGCA TAAATAGCAG AATTACAACG ATTATGACTA
3_Thai          TTACAGAAAT TAATAAAAAA ATAGACAGCA TAAATAGCAG AATTACAACG ATTATGACTA
Conservation    ********** ********** ********** ********** ********** **********

                ....|....| ....|....| ....|....| ....|....| ....|....| ....|....|
                    6425       6435       6445       6455       6465       6475
3_SalI          GTCTTAACGA GTTGCTAGTA ACAGGAAAGG CATGTGAGAA ATCGTCTTAT GCTTCCCTAA
3_Thai          GTCTTAACGA GTTGCTAGTA ACAGGAAAGG CATGTGAGAA ATCGTCTTAT GCTTCCCTAA
Conservation    ********** ********** ********** ********** ********** **********

                ....|....| ....|....| ....|....| ....|....| ....|....| ....|....|
                    6485       6495       6505       6515       6525       6535
3_SalI          TTGGTAATAT AAATGCGAAA ACGTCAAGTG ACTTAAGAAT AATCAGTAAC CAAAAGGAAA
3_Thai          TTGGTAATAT AAATGCGAAA ACGTCAAGTG ACTTATGAAT AATCAGTAAC CAAAAGGAAA
Conservation    ********** ********** ********** *****:**** ********** **********

                ....|....| ....|....| ....|....| ....|....| ....|....| ....|....|
                    6545       6555       6565       6575       6585       6595
3_SalI          ACGCGGAAAA GTACGTAGAA TACATTAAGA AAAATTCAAA CTTGATAAAT GATGACATTC
3_Thai          ACGCGGAAAA GTACGTAGAA TACATTAAGA AAAATTCAAA CTTGATAAAT GATGACATTC
Conservation    ********** ********** ********** ********** ********** **********
```

```
             ....|....| ....|....| ....|....| ....|....| ....|....| ....|....|
                 6605       6615       6625       6635       6645       6655
3_SalI       GTGCATTAAA TAAATACTTT GATACTAATA GGATAAATAA TTACCAGTTG AAAAATTTGG
3_Thai       GTGCATTAAA TAAATACTTT GATACTAATA GGATAAATAA TTACCAGTTG AAAAATTTGG
Conservation ********** ********** ********** ********** ********** **********

             ....|....| ....|....| ....|....| ....|....| ....|....| ....|....|
                 6665       6675       6685       6695       6705       6715
3_SalI       AAGAAGTAAT TAAACACGCT AATGATTTTA ACGCAAAGGA AAAAGAAGCA ACGGGAATAG
3_Thai       AAGAAGTAAT TAAACACGCT AATGATTTTA ACGTAAAGGA AAAAGAAGCA ACGGGAATAG
Conservation ********** ********** ********** *** ****** ********** **********

             ....|....| ....|....| ....|....| ....|....| ....|....| ....|....|
                 6725       6735       6745       6755       6765       6775
3_SalI       TTAACGAAAT TAAAAAAGAA TTCCTAGACC TAAGTCGGGA GGTAGAAATG AATTTCTTAA
3_Thai       TTAACGAAAT TAAAAAAGAA TTCCTAGACC TAAGTCGGGA GGTAGAAATG AATTTCTTAA
Conservation ********** ********** ********** ********** ********** **********

             ....|....| ....|....| ....|....| ....|....| ....|....| ....|....|
                 6785       6795       6805       6815       6825       6835
3_SalI       ATAGCAGCAA AGAAAAAATT ATGGAACATT ACAAAAATCT GAAAGATAAA ATAAAGAGCA
3_Thai       ATAGCAGCAA AGAAAAAATT ATGGAACATT ACAAAAATCT GAAAGATAAA ATAAAGAGCA
Conservation ********** ********** ********** ********** ********** **********

             ....|....| ....|....| ....|....| ....|....| ....|....| ....|....|
                 6845       6855       6865       6875       6885       6895
3_SalI       TTAACGAAGT TTATAAAAAT ATCAACTTGG TAAAATTGAA AGAAATGGAA AGTAGCTCAG
3_Thai       TTAACGAAGT TTATAAAAAT ATCAACTTGG TAAAATTGAA AGAAATGGAA AGTAGCTCAG
Conservation ********** ********** ********** ********** ********** **********

             ....|....| ....|....| ....|....| ....|....| ....|....| ....|....|
                 6905       6915       6925       6935       6945       6955
3_SalI       ATAAGTACTT AGAAATAGCA GGGAAATTTA AAAATGTGCT AGATACTCAA ATAACAAGGC
3_Thai       ATAAGTACTT AGAAATAGCA GGGAAATTTA AAAATGTGCT AGATACTCAA ATAACAAGGC
Conservation ********** ********** ********** ********** ********** **********

             ....|....| ....|....| ....|....| ....|....| ....|....| ....|....|
                 6965       6975       6985       6995       7005       7015
3_SalI       TGTTGGATAA TCATATGGTG CTACAAGATA TCGAAAAAAA TATAACAGAA AATGAAGGGG
3_Thai       TGTTGGATAA TCATATGGTG CTACAAGATA TCGAAAAAAA TATAACAGAA AATGAAGGGG
Conservation ********** ********** ********** ********** ********** **********

             ....|....| ....|....| ....|....| ....|....| ....|....| ....|....|
                 7025       7035       7045       7055       7065       7075
3_SalI       AACTGAAAGG TATTAGTAAT ACATACACAC TACAGTCCAT CCAAAAGTTT AACAATGTGT
3_Thai       AACTGAAAGG TATTAGTAAT ACATACACAC TACAGTCCAT CCAAAAGTTT AACAATGTGT
Conservation ********** ********** ********** ********** ********** **********

             ....|....| ....|....| ....|....| ....|....| ....|....| ....|....|
                 7085       7095       7105       7115       7125       7135
3_SalI       GTAAGAATAT TGAGACCAAT ATGCAGAAGC TGCATGAAGT AGAGCAGTAG AATAACAGTG
3_Thai       GTAAGAATAT TGAGACCAAT ATGCAGAAGC TGCATGAAGT AGAGCAGTAG AATAACAGTG
Conservation ********** ********** ********** ********** ********** **********

             ....|....| ....|....| ....|....| ....|....| ....|....| ....|....|
                 7145       7155       7165       7175       7185       7195
3_SalI       AGGAAAAACA GGTGAAAGCC TGTATAGAAA ATGTGTCCCA TCTTATAAAC AGAGGAAACA
3_Thai       AGGAAAAACA GGTGAAAGCC TGTATAGAAA ATGTGTCCCA TCTTATAAAC AGAGGAAACA
Conservation ********** ********** ********** ********** ********** **********
```

```
             ....|....| ....|....| ....|....| ....|....| ....|....| ....|....|
                 7205       7215       7225       7235       7245       7255
3_SalI       ATTTGTTGAC CGATTTAAAT GATTACGATG TAGTAAGCCA TAGTGCGGCG AAAGAAATAA
3_Thai       ATTTGTTGAC CGATTTAAAT GATTACGATG TAGTAAGCCA TAGTGCGGCG AAAGAAATAA
Conservation ********** ********** ********** ********** ********** **********

             ....|....| ....|....| ....|....| ....|....| ....|....| ....|....|
                 7265       7275       7285       7295       7305       7315
3_SalI       CTGATGAGAG TACAAAAGAG TACATTACAA AGATTAAAGG AAAGGTAAAT AATACCATAG
3_Thai       CTGATGAGAG TACAAAAGAG TACATTACAA AGATTAAAGG AAAGGTAAAT AATACCATAG
Conservation ********** ********** ********** ********** ********** **********

             ....|....| ....|....| ....|....| ....|....| ....|....| ....|....|
                 7325       7335       7345       7355       7365       7375
3_SalI       AGGCATTTCA AAAGGTGTTA GAAAACATTC AGGAAAATAA ATCGCACACA CAGAACAATG
3_Thai       AGGCATTTCA AAAGGTGTTA GAAAACATTC AGGAAAATAA ATCGCACACA CAGAACAATG
Conservation ********** ********** ********** ********** ********** **********

             ....|....| ....|....| ....|....| ....|....| ....|....| ....|....|
                 7385       7395       7405       7415       7425       7435
3_SalI       GAGATTTAAA TAAAGGCATA TACGAAATAT GGAAAAGGGC TAACGAAATT AAGGCAAACT
3_Thai       GAGATTTAAA TAAAGGCATA TACGAAATAT GGAAAAGGGC TAACGAAATT AAGGCAAACT
Conservation ********** ********** ********** ********** ********** **********

             ....|....| ....|....| ....|....| ....|....| ....|....| ....|....|
                 7445       7455       7465       7475       7485       7495
3_SalI       TCACGGAAAA TTTTCCAGAA AATGATAACT ACTTTCAAGT GGGGGATTAT CTAAAGGATA
3_Thai       TCACGGAAAA TTTTCCAGAA AATGATAACT ACTTTCAAGT GGGGGATTAT CTAAAGGATA
Conservation ********** ********** ********** ********** ********** **********

             ....|....| ....|....| ....|....| ....|....| ....|....| ....|....|
                 7505       7515       7525       7535       7545       7555
3_SalI       TAAAAAACAT TTTGAACGAA ACTGTTGGAG GTGCCAACAT AGAGGAATAT ATCAAAAGCG
3_Thai       TAAAAAACAT TTTGAACGAA ACTGTTGGAG GTGCCAACAT AGAGGAATAT ATCAAAAGCG
Conservation ********** ********** ********** ********** ********** **********

             ....|....| ....|....| ....|....| ....|....| ....|....| ....|....|
                 7565       7575       7585       7595       7605       7615
3_SalI       TTTCTCAAAA TATTGAAGAA CAAATAAAGG GTACACAAAA TCATCGTAAT GTGGAATCCA
3_Thai       TTTCTCAAAA TATTGAAGAA CAAATAAAGG GTACACAAAA TCATCGTAAT GTGGAATCCA
Conservation ********** ********** ********** ********** ********** **********

             ....|....| ....|....| ....|....| ....|....| ....|....| ....|....|
                 7625       7635       7645       7655       7665       7675
3_SalI       TTTTAAAAGC CAAAAAAAAG ATTCAATCTT ATAATGAAGA GGCAAACAAA ACATTACAAT
3_Thai       TTTTAAAAGC CAAAAAAAAG ATTCAATCTT ATAATGAAGA GGCAAACAAA ACATTACAAT
Conservation ********** ********** ********** ********** ********** **********

             ....|....| ....|....| ....|....| ....|....| ....|....| ....|....|
                 7685       7695       7705       7715       7725       7735
3_SalI       CTATGAACAC TGCTCAGGAT AAAATTTTGT TAAAGAAAAA AGACATGGAT AATATATTTA
3_Thai       CTATGAACAC TGCTCAGGAT AAAATTTTGT TAAAGAAAAA AGACATGGAT AATATATTTA
Conservation ********** ********** ********** ********** ********** **********

             ....|....| ....|....| ....|....| ....|....| ....|....| ....|....|
                 7745       7755       7765       7775       7785       7795
3_SalI       GTATTCTATC CGTAAATATG AAGAATAGTG TGTACATAAA TACGAAAAAA TATATTAACG
3_Thai       GTATTCTATC CGTAAATATG AAGAATAGTG TGTACATAAA TACGAAAAAA TATATTAACG
Conservation ********** ********** ********** ********** ********** **********
```

```
             ....|....| ....|....| ....|....| ....|....| ....|....| ....|....|
                  7805       7815       7825       7835       7845       7855
3_SalI       AGGTGGATGA TTTATTTAAC AAGTTAAAAG TGGATGTACA TAAGTTGGAA AATTTTATAA
3_Thai       AGGTGGATGA TTTATTTAAC AAGTTAAAAG TGGATGTACA TAAGTTGGAA AATTTTATAA
Conservation ********** ********** ********** ********** ********** **********

             ....|....| ....|....| ....|....| ....|....| ....|....| ....|....|
                  7865       7875       7885       7895       7905       7915
3_SalI       ATGATACTGA ACTGAGAATA AAGCAGTTAG AGGAAGAGGA AGCTAAGCTC AAAATTAAGA
3_Thai       ATGATACTGA ACTGAGAATA AAGCAGTTAG AGGAAGAGGA AGCTAAGCTC AAAATTAAGA
Conservation ********** ********** ********** ********** ********** **********

             ....|....| ....|....| ....|....| ....|....| ....|....| ....|....|
                  7925       7935       7945       7955       7965       7975
3_SalI       ATGCAGTTAA TGGTGATTTG AATGAGCATT CTGAGGATGC TTCTCGTGAT GAGGACGACG
3_Thai       ATGCAGTTAA TGGTGATTTG AATGAGCATT CTGAGGATGC TTCTCGTGAT GAGGACGACG
Conservation ********** ********** ********** ********** ********** **********

             ....|....| ....|....| ....|....| ....|....| ....|....| ....|....|
                  7985       7995       8005       8015       8025       8035
3_SalI       AACCATTAGG TGAGGTTACG CAAAATTCAG ATTCGGAAGG GTCAAGTGAT AACCAAAATA
3_Thai       AACCATTAGG TGAGGTTACG CAAAATTCAG ATTCGGAAGG GTCAAGTGAT AACCAAAATA
Conservation ********** ********** ********** ********** ********** **********

             ....|....| ....|....| ....|....| ....|....| ....|....| ....|....|
                  8045       8055       8065       8075       8085       8095
3_SalI       GGCGTAAACG AAATGGTGCA AGTAGCCATT CAAAAGGAAA TTCTTCTAAT AACTCTTTGA
3_Thai       GGCGTAAACG AAATGGTGCA AGTAGCCATT CAAAAGGAAA TTCTTCTAAT AACTCTTTGA
Conservation ********** ********** ********** ********** ********** **********

             ....|....| ....|....| ....|....| ....|....| ....|....| ....|....|
                  8105       8115       8125       8135       8145       8155
3_SalI       ATAATGTGCA TGGATTAAAA GAAGATAATA AAACTGGTGA CAACCATTCT GATGACAATC
3_Thai       ATAATGTGCA TGGATTAAAA GAAGATAATA AAACTGGTGA CAACCATTCT GATGACAATC
Conservation ********** ********** ********** ********** ********** **********

             ....|....| ....|....| ....|....| ....|....| ....|....| ....|....|
                  8165       8175       8185       8195       8205       8215
3_SalI       AGAAAGAAGA ATCTCATAAT GGAAGTAATA ATTCGGGTGG AAAAGGCTCA CGGGAGAGGT
3_Thai       AGAAAGAAGA ATCTCATAAT GGAAGTAATA ATTCGGGTGG AAAAGGCTCA CGGGAGAGGT
Conservation ********** ********** ********** ********** ********** **********

             ....|....| ....|....| ....|....| ....|....| ....|....| ....|....|
                  8225       8235       8245       8255       8265       8275
3_SalI       TCAGATATGC TGGAGGAATT ACGCTAGCAT TTTTTATTTG CTCAAGTGCT GGATTCGCAA
3_Thai       TCAGATATGC TGGAGGAATT ACGCTAGCAT TTTTTATTTG CTCAAGTGCT GGATTCGCAA
Conservation ********** ********** ********** ********** ********** **********

             ....|....| ....|....| ....|....| ....|....| ....|....| ....|....|
                  8285       8295       8305       8315       8325       8335
3_SalI       TCATTAAATA TAAAAATCAT GAATCACAAG AAGCCGATTT CGGAACAGAC AAGGAGCACT
3_Thai       TCATTAAATA TAAAAATCAT GAATCACAAG AAGCCGATTT CGGAACAGAC AAGGAGCACT
Conservation ********** ********** ********** ********** ********** **********

             ....|....| ....|....| ....|....| ....|....| ....|....| ....|....|
                  8345       8355       8365       8375       8385       8395
3_SalI       TTGAGGGGGA TAAAGATTTT AATAAACGGG AAAAGGAGGA AATTATTGAA GTTTCTTTTA
3_Thai       TTGAGGGGGA TAAAGATTTT AATAAACGGG AAAAGGAGGA AATTATTGAA GTTTCTTTTA
Conservation ********** ********** ********** ********** ********** **********
```

```
             ....|....| ....|....| ...
                8405       8415
3_SalI       ACGAAAATGA ACACTATGAA TGA
3_Thai       ACGAAAATGA ACACTATGAA TGA
Conservation ********** ********** ***
```
